# Copper‐Mediated *N*‐Trifluoromethylation of *O*‐Benzoylhydroxylamines

**DOI:** 10.1002/chem.202303314

**Published:** 2023-12-07

**Authors:** Thomas D. Fleetwood, William J. Kerr, Joseph Mason

**Affiliations:** ^1^ Medicinal Chemistry GSK Medicines Research Centre Gunnels Wood Road SG1 2NY Stevenage England U.K.; ^2^ Department of Pure and Applied Chemistry University of Strathclyde G1 1XL Glasgow Scotland U.K.

**Keywords:** Copper, Amination, Trifluoromethyl, Catalysis, *N*-Trifluoromethylamines

## Abstract

The use of trifluoromethyl containing compounds is well established within medicinal chemistry, with a range of approved drugs containing *C*−CF_3_ and *O*−CF_3_ moieties. However, the utilisation of the *N*−CF_3_ functional group remains relatively unexplored. This may be attributed to the challenging synthesis of this unit, with many current methods employing harsh conditions or less accessible reagents. A robust methodology for the *N*‐trifluoromethylation of secondary amines has been developed, which employs an umpolung strategy in the form of a copper‐catalysed electrophilic amination. The method is operationally simple, uses mild, inexpensive reagents, and has been used to synthesise a range of novel, structurally complex *N*−CF_3_ containing compounds.

## Graphical abstract

A copper‐mediated electrophilic amination methodology has been developed to provide access to a range of structurally diverse *N*−CF_3_ amines in good to moderate yields. The method proceeds under mild reaction conditions utilizing inexpensive reagents in an operationally simple manner.

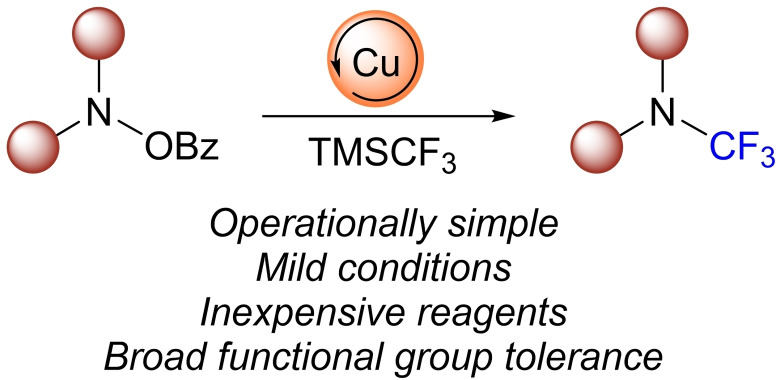

## Introduction

The trifluoromethyl group is a powerful tool within medicinal chemistry and chemical biology due to its ability to modulate the pharmaceutically relevant properties of bioactive molecules.^[1]^ The influences of this unit can range from protecting a drug from metabolism,^[1]^ to increasing the overall potency of a drug *via* modulation of protein‐ligand interactions.^[1]^ The high number of CF_3_‐containing drugs that have progressed to market (72, as of August 2020)^[2]^ provides further evidence of the applicability and potential of this functional group within medicinal chemistry programmes and chemical biology studies. In a more specific sense, Schiesser *et al*. demonstrated that when compared to the respective *N*‐methyl analogue, select *N*−CF_3_ amines and azoles displayed increased lipophilicity as well as a decrease in their p*K*_a_H values.^[2]^ The ability to modulate these two properties can be desirable within drug discovery, in which the former can lead to the enhancement of a drug's binding affinity and permeability,^[3]^ whilst the latter can be beneficial in controlling DMPK properties.^[4]^

Despite these advantages, the incorporation of *N*−CF_3_ amines into pharmaceutical compounds remains relatively unexplored. With regards to alkyl *N*−CF_3_ amines, a key factor may be related to their potential conversion to carbamoyl fluorides under aqueous conditions.^[2]^ Whilst this transformation has some published precedent for alkyl *N*−CF_3_ amines,[^2^, ^5^] there are only a limited number of examples where this is reported. To facilitate further investigation, a preparatively convenient and accessible method to allow access to more elaborate sets of alkyl *N*−CF_3_ compounds would be beneficial. This is especially so in a drug discovery and chemical biology context, where new methods for synthesis would provide a platform to facilitate an enhanced understanding of the stability and utility of such species.

**Scheme 1:**
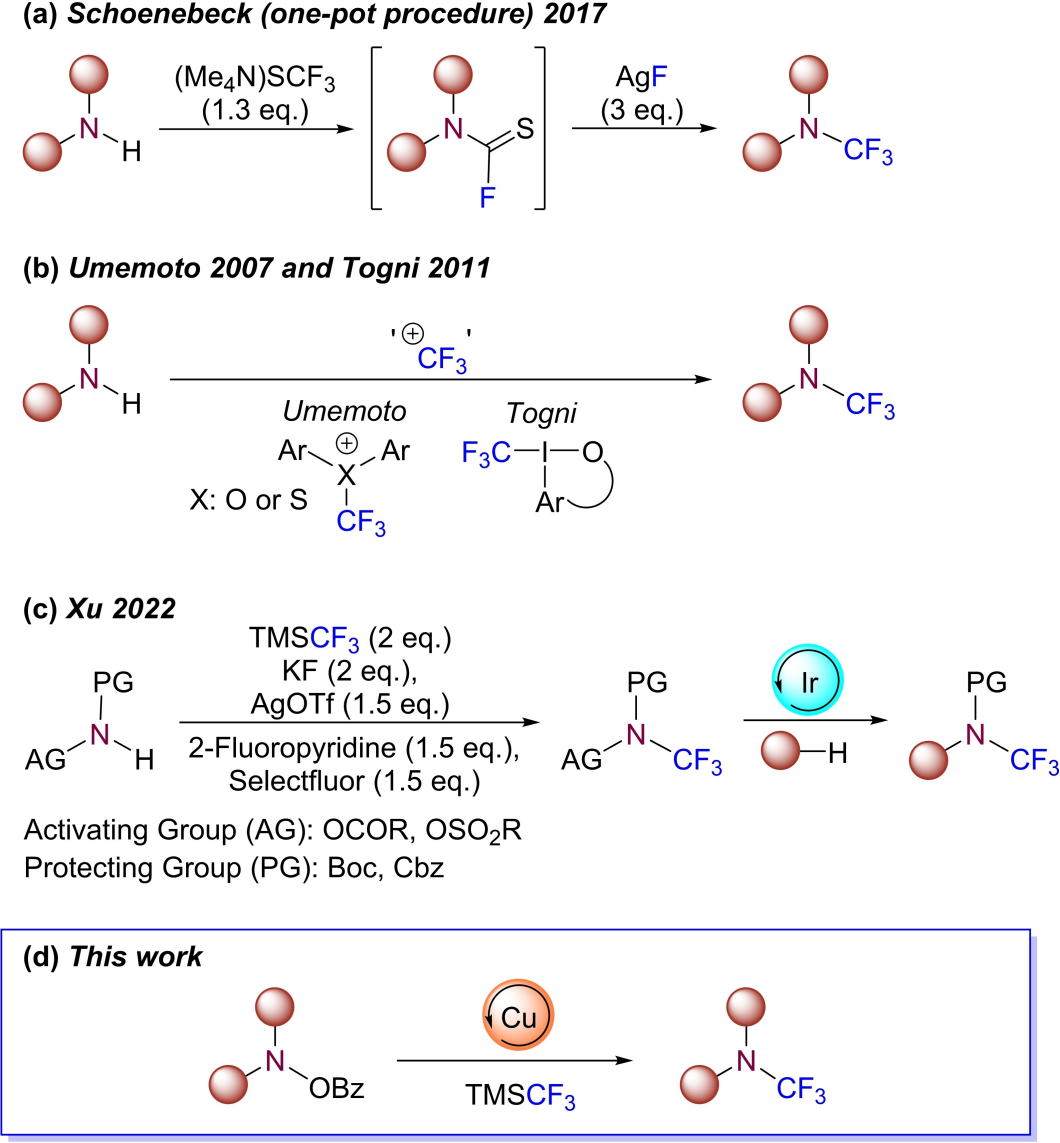
(a) One‐pot process via thiocarbamoyl fluorides; (b) Electrophilic *N*‐trifluoromethylation; (c) Synthesis of protected *N*−CF_3_ hydroxylamines and their subsequent photo‐catalysed cross‐coupling to unsaturated compounds; (d) Copper‐catalyzed electrophilic amination of *O*‐benzoylhydroxylamines to deliver *N*−CF_3_ amines (*this work*).

A range of classical methods for the synthesis of *N*−CF_3_ amines has been previously reported,^[6]^ focusing on functional group transformations such as fluorine‐halogen exchange,^[7]^ or oxidative‐desulfurisation fluorination from dithiocarbamates.[^5c^, ^5d^, ^8^] However, these methods are often accompanied with hazardous reagents, harsh reaction conditions, and a limited substrate scope. The latest advances in the synthesis of *N*−CF_3_ amines are largely associated with the fluorination of thiocarbamoyl fluorides (Scheme 1a). Based on the pioneering work by Schoenebeck *et. al*.,^[9]^ this approach has been used to synthesise a wide range of *N*−CF_3_ amines in good yields under mild reaction conditions.[^5a^, ^9^, ^10^] Despite the Schoenebeck method employing the bench‐stable (Me_4_N)SCF_3_ in a one‐pot protocol, the drawback of this method is the use, and associated relative expense, of super‐stoichiometric quantities of AgF.^[11]^ Alternatively, *N*−CF_3_ amines may be synthesised by the direct trifluoromethylation of amines, using electrophilic reagents developed by Umemoto and Togni *et al*. (Scheme 1b).^[12]^ However, the required pre‐synthesis and cost of these reagents is a limitation of their use. A recent publication by Xu and co‐workers exemplified an alternate approach to *N*‐trifluoromethylation whereby the entire *N*−CF_3_ unit is cross‐coupled to a range of substrates (Scheme 1c).^[13]^ Despite this, in the core and direct C−H activation pathway as described, only unsaturated compounds could be used as coupling partners.

Recognising the constraints within the established pathways for accessing *N*−CF_3_ compounds, we set out to develop a methodology that would utilise mild, inexpensive reagents and be operationally simple. Copper‐mediated electrophilic amination has been employed widely for the creation of C−N bonds^[14]^ and, whilst effective in the creation of *N*‐alkyl and *N*‐aryl compounds, such approaches have yet to be used for the creation of *N*−CF_3_ compounds. Conceptually, the replacement of an alkyl/aryl nucleophilic coupling partner with a practicable nucleophilic source of CF_3_, alongside the concomitant formulation of an effective catalytic manifold, would provide access to *N*−CF_3_ amines. Herein, we report the development of a robust protocol for the synthesis of *N*−CF_3_ amines from *O*‐benzoylhydroxylamines under a copper‐catalyzed electrophilic amination process (Scheme 1d).

## Results and Discussion

**Scheme 2:**
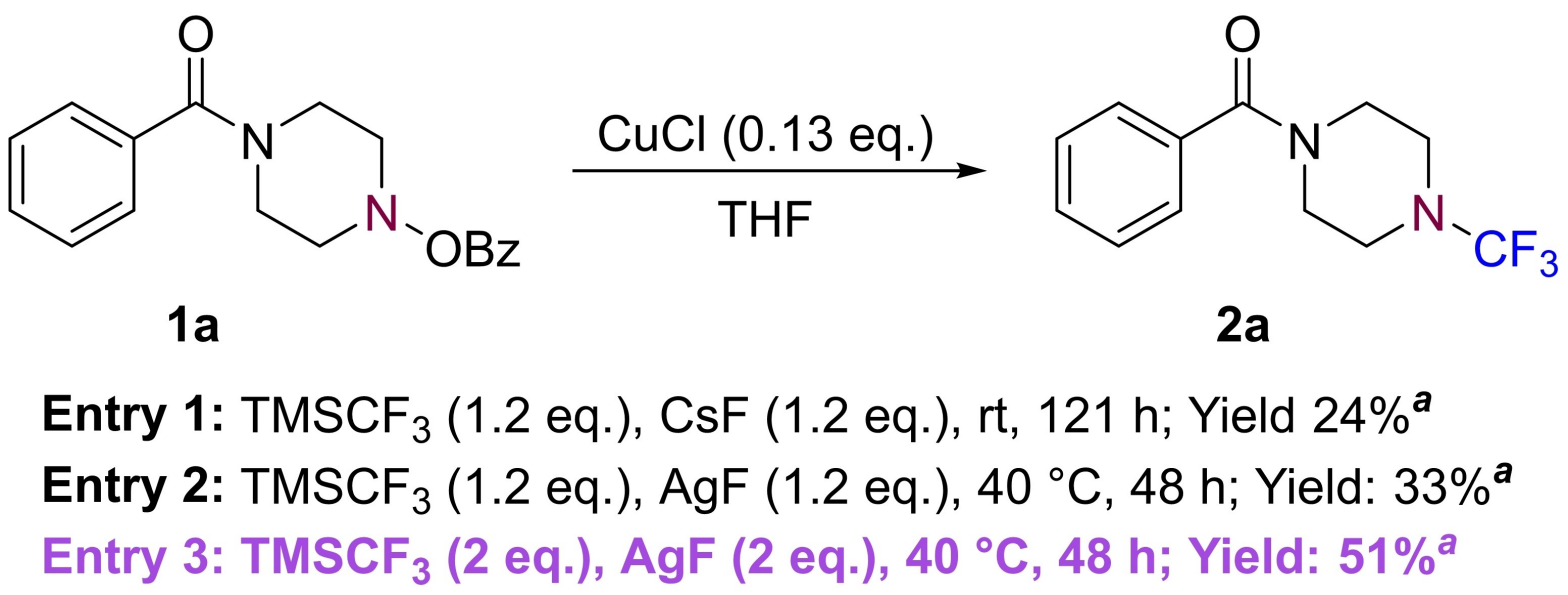
Initial *N*‐trifluoromethylation reactions of **1 a**. ^*a*^ Isolated yield.

Our investigations began with the identification of a suitable nucleophilic CF_3_ source. The Ruppert‐Prakash (RP) reagent,^[15]^ trifluoromethyltrimethylsilane (TMSCF_3_), was selected due to its precedented use in *C*−CF_3_ trifluoromethylation processes.^[16]^ Additionally, and of particular relevance to this investigation, it has been reported that Cu−CF_3_ can be generated *in situ* (based on solution NMR analysis) from the combination of TMSCF_3_, a copper halide, and a fluoride activator.[^17^, ^18^] It was, therefore, envisaged that the desired *N‐*trifluoromethylation could be achieved by incorporating this practical approach, in combination with the use of an electrophilic *O*‐benzoylhydroxylamine coupling partner. As shown in Scheme 2, our initial test of this hypothesis proved to be successful, with the isolation of trifluoromethylamine **2 a** in a 24 % yield, which was later improved to 51 % upon switching to AgF as the fluoride activator and increasing the equivalents of TMSCF_3_.

**Figure 1:**
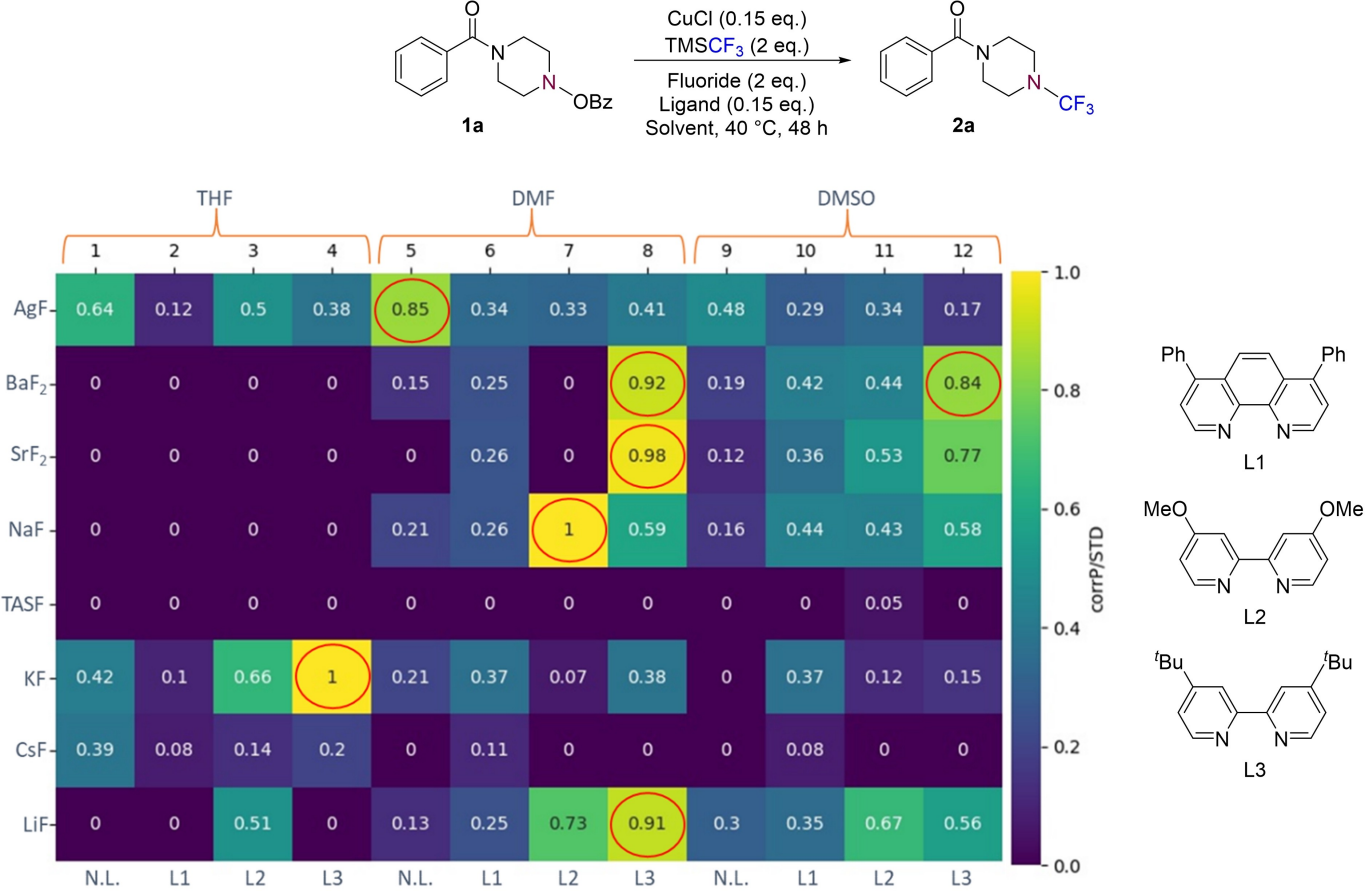
High‐throughput screening (HTS) for the optimisation of the desired reaction. CorrP/STD=ratio of the product to the internal standard (IS), relative to the maximum observed product : IS ratio over the whole plate. N.L.=No ligand.

We envisaged that the effectiveness of the process could be further enhanced by investigating a range of reaction conditions as part of a high‐throughput screening (HTS) approach (each performed on 10 μmol scale).^[19]^ Switching from CsF to AgF had previously been observed to increase the yield of the *N*−CF_3_ product **2 a** in our benchmark process and so it was decided that a range of commercially available fluoride sources should be screened. Three polar aprotic solvents were chosen based on their precedented use in copper‐mediated trifluoromethylation processes,^[20]^ and three electron‐rich diamine ligands were employed in order to modulate the stereoelectronics of the copper catalyst. Following this HTS approach, seven hits were identified (outlined in red in Figure 1);^[21]^ each of these conditions were then validated by repeating the reaction on a 0.322 mmol scale and obtaining an isolated yield (Table 1, entries A–G). Yields of ≥60 % were obtained for all of these enhanced scale processes. Further, after testing a number of copper sources (see SI for full optimisation details), it was found that replacement of CuCl with CuBr led to improved yields of up to 88 % (Table 1, entry H; mean yield from a triplicate dataset).

**Table 1:** Validation of HTS hits.

Entry	Fluoride source	Ligand	Solvent	Isolated yield of **2 a**/%
A	AgF	No ligand	DMF	63
B	BaF_2_	^*t*^Bu‐Bipy (L3)	DMF	73
C	BaF_2_	^*t*^Bu‐Bipy (L3)	DMSO	67
D	SrF_2_	^*t*^Bu‐Bipy (L3)	DMF	75
E	NaF	MeO‐Bipy (L2)	DMF	77
F	KF	^*t*^Bu‐Bipy (L3)	THF	60
G	LiF	^*t*^Bu‐Bipy (L3)	DMF	71
H^[b]^	SrF_2_	^*t*^Bu‐Bipy (L3)	DMF	88^[a]^
I^[b]^	NaF	^*t*^Bu‐Bipy (L3)	DMF	81

[a] Mean yield; reaction run in triplicate. [b] CuCl replaced with CuBr.

**Scheme 3:**
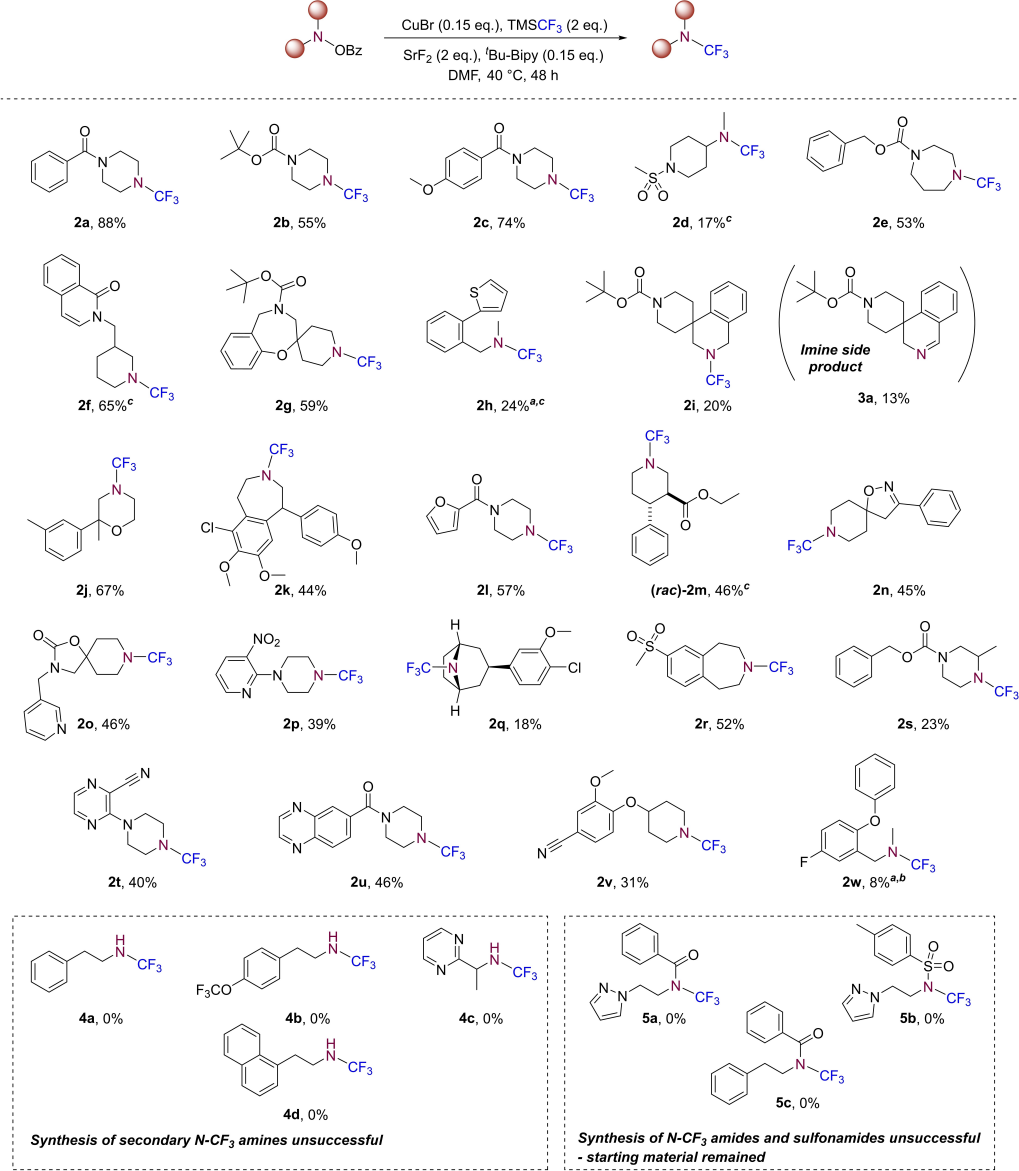
Substrate scope for the *N*‐trifluoromethylation of *O*‐benzoylhydroxylamines. All yields reported are isolated. N=2 obtained for each reaction and average yields reported unless stated otherwise. ^*a*^ N=1 obtained. ^*b*^ Tentative assignment as only ^1^H, ^19^F NMR and LCMS data could be acquired. ^*c*^ Tentative yield due to traces of impurities or residual solvent present.

With an optimised set of conditions in hand, the substrate scope was investigated to test the applicability of the optimised reaction conditions to other secondary amines bearing a range of functional groups (Scheme 3). Accordingly, a set of structurally diverse tertiary *N*−CF_3_ amines were synthesised in good to moderate yields, the majority of which have not previously been prepared. In each case, the isolated yield reported is an average of two runs, each performed separately. The established protocol was found to tolerate a wide array of diverse functional groups, including amides (**2 a**, **2 c**, **2 f**), carbamates (**2 b**, **2 e**, **2 g**, **2 i**, **2 s**), sulfonamides (**2 d**), aromatic and (partially) saturated heterocycles (**2 j**, **2 l**, **2 n**, **2 o**, **2 u**), sulfones (**2 r**), halogens (**2 k**, **2 q**, **2 w**), esters (**2 m**), nitro (**2 p**) and nitrile (**2 t**, **2 v**) groups. Whilst it is acknowledged that some of the yields obtained were moderate to low, further investigations and forthcoming discussion provides some explanation for these cases.

As also detailed in Scheme 3, attempts to apply this methodology to the synthesis of secondary *N*−CF_3_ amines was unsuccessful, which may be attributed to their instability,[^13^, ^22^] likely during the reaction as no product was observed via LCMS analysis of the reaction mixture. Application to the direct synthesis of *N*−CF_3_ amides and sulfonamides was also not possible, where an inherent lack of reactivity of the *O*‐benzoylhydroxylamines of these substrates was observed. Additionally, unsuccessful attempts to form other tertiary *N*−CF_3_ amines, including those that were more sterically hindered, are also provided in the supplementary information.

**Scheme 4:**
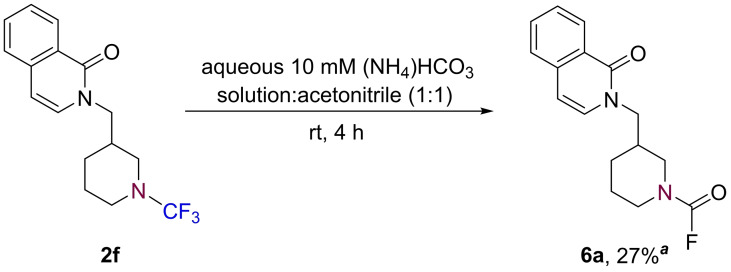
Transformation of alkyl *N*−CF_3_ product **2 f** to carbamoyl fluoride **6 a**. ^*a*^ Isolated yield.

In the course of our investigations, we observed that, when exposed to the aqueous conditions of the LCMS analysis, a sub‐set of products transformed into the corresponding carbamoyl fluoride species. It has been previously reported that alkyl *N*−CF_3_ amines, particularly those without electron‐withdrawing groups attached, are susceptible to fluorine elimination;[^2^, ^5^] furthermore, the groups of Schindler, Yi, and Xiao each reported independently that degradation can occur rapidly upon flash column chromatography purification.[^5a^, ^10a^, ^10d^] To confirm that the aqueous conditions of the LCMS were the cause of the observed transformation, compound **2 f** was subjected to the relevant aqueous ammonium bicarbonate conditions used in the LCMS analysis, which resulted in the formation of carbamoyl fluoride **6 a** in 27 % isolated yield (Scheme 4).

Whilst the inherent instability of certain *N*−CF_3_ products will be undesirable for certain applications, Schoenebeck *et al*. have shown that the resultant carbamoyl fluorides can be used as effective electrophilic handles for further derivatisation.^[23]^ With the developed Cu‐catalyzed method now providing more ready accessibility to *N*−CF_3_ compounds, alongside the potentially controlled generation of carbamoyl fluorides, we also hypothesize that the electrophilic nature of these latter species may find application within chemical biology as suitable reactive probes,^[24]^ similar to sulfonyl fluorides.^[25]^ The feasibility of this proposal would, in due course, require further investigation.

**Scheme 5:**
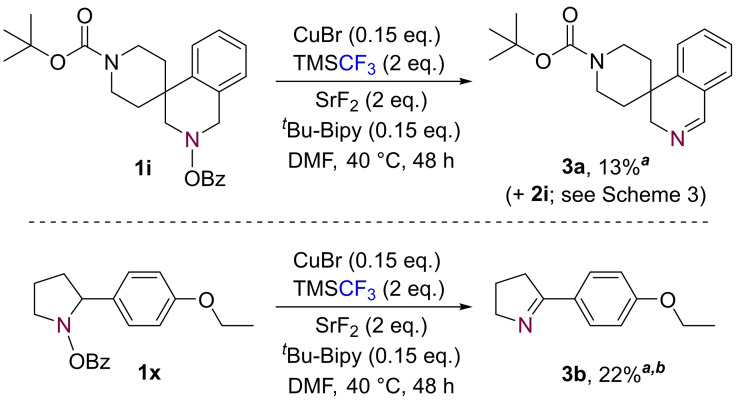
Imine formation side reaction. ^*a*^ Isolated yield. ^*b*^ N=2 obtained for product **3 b** and average yield reported.

In the course of our investigations, we were also able to isolate elimination side‐products (Scheme 5), suggesting that this may be an alternative reaction pathway for our substrates. More specifically, when using substrate **1 i** the imine by‐product **3 a** was formed in 13 % yield alongside desired product **2 i** (see Scheme 3). Subjecting compound **1 x** to the same optimised trifluoromethylation conditions yielded imine **3 b** (22 %), with no evidence for the formation of the *N*−CF_3_ species.

**Scheme 6:**
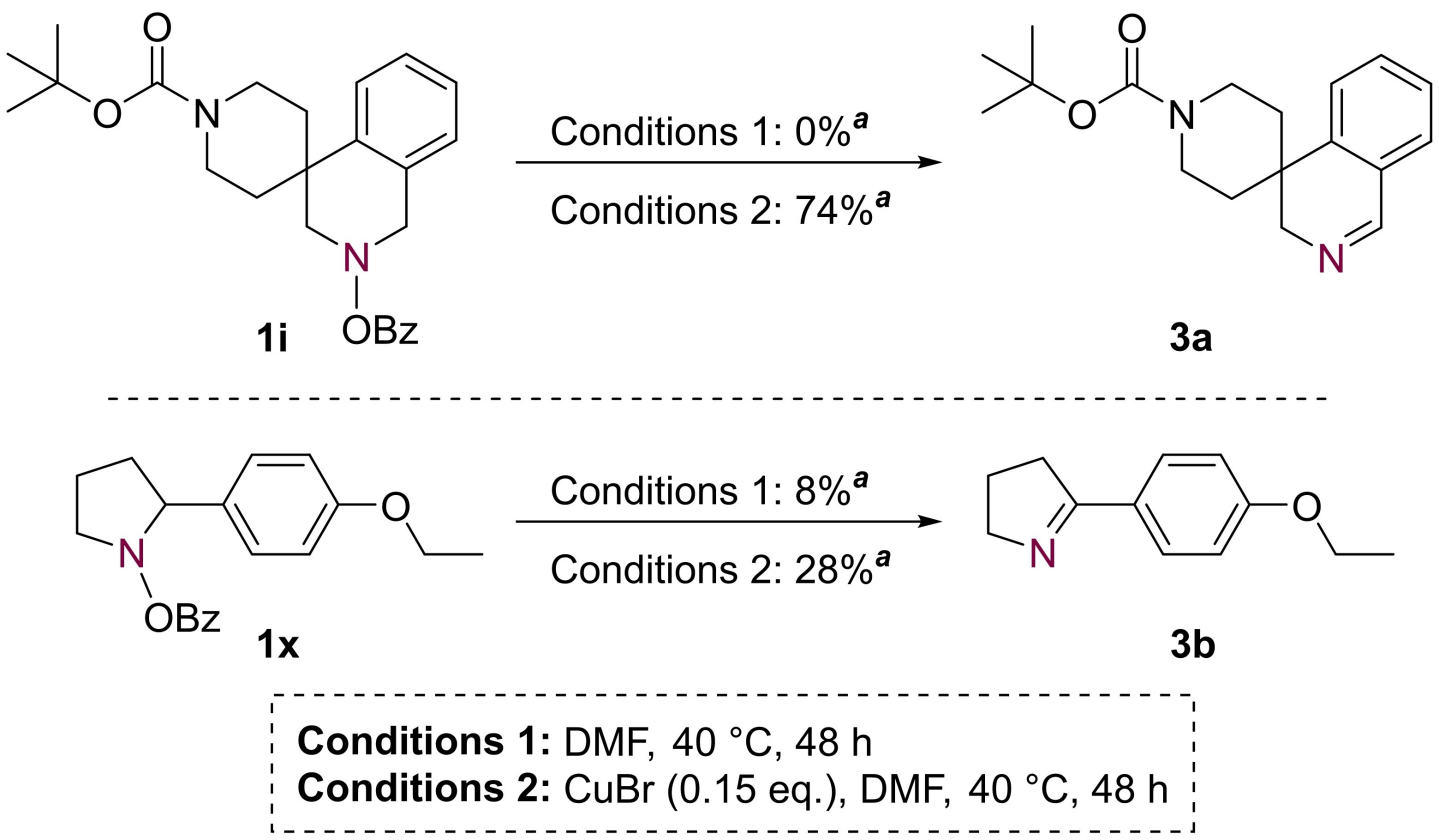
Imine formation control reactions. ^*a*^ Isolated yield.

A series of control experiments determined that the imines may be generated in low yield in the absence of other reagents (for substrate **1 x**) or, more effectively, in the presence of CuBr (Scheme 6). We tentatively propose that in the absence of a copper catalyst, imine formation is the result of an intramolecular *retro*‐ene‐type reaction;^[26]^ the presence of the copper for each substrate may catalyze this elimination pathway, or instead may mediate a β‐hydride elimination process from a Cu intermediate (*vide infra*).^[27]^

**Scheme 7:**
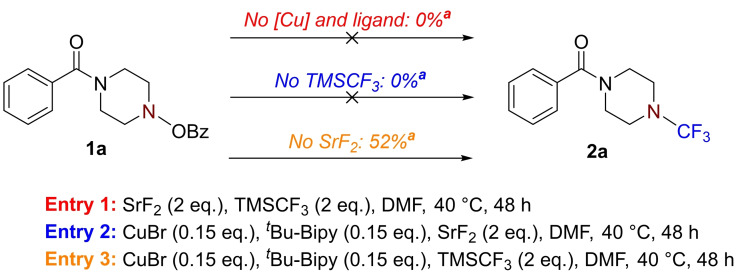
Mechanistic control experiments. ^*a*^ Isolated yield.

In order to gain further insight into the possible reaction pathway for the developed *N*‐trifluoromethylation, a further series of control experiments was conducted to evaluate the role of each reagent (Scheme 7). When the copper and ligand were removed from the reaction mixture, no reaction occurred, indicating that the desired *N*−CF_3_ forming process is, indeed, copper‐mediated. When the Ruppert‐Prakash reagent, TMSCF_3_, was removed, the starting material was consumed and a complex mixture was formed; however, no *N*‐trifluoromethyl product was observed. This highlights that the source of CF_3_ is derived from the RP reagent. Interestingly, when the fluoride source (SrF_2_) was removed, the desired product was observed and isolated in 52 % yield. Accordingly, it was concluded that such a fluoride activator is not required for reaction success, but is beneficial; this also suggests that TMSCF_3_ can be activated by an alternative nucleophilic species, although, an appreciable increase in yield is observed when fluoride is present (*cf*. the 88 % yield for **2 a** as described in Table 1 and Scheme 3).

**Scheme 8:**
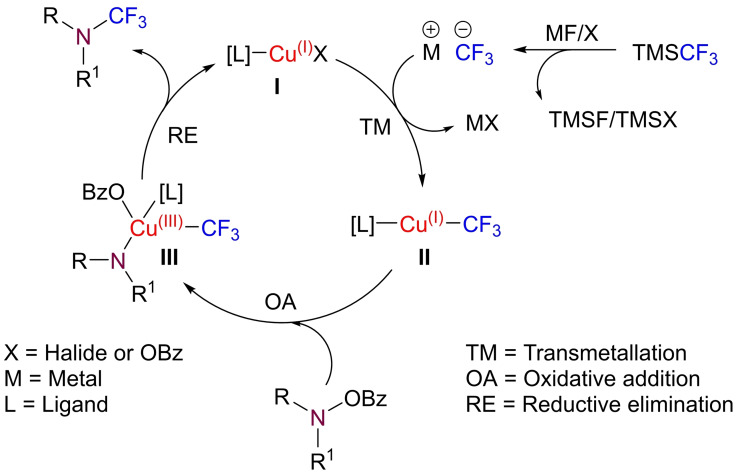
Proposed mechanistic cycle.

Based on these results, as well as previous mechanistic reports within the fields of transition metal‐catalyzed electrophilic amination^[14a]^ and copper‐mediated trifluoromethylation reactions,[^20^, ^28^] the following mechanistic cycle is proposed (Scheme 8). Firstly, TMSCF_3_ is activated by the fluoride or other nucleophilic group (e. g. Br^−^, BzO^−^) to generate a reactive M−CF_3_ species. M−CF_3_ then undergoes transmetallation (TM) onto Cu(I)X (**I**) to form a Cu(I)CF_3_ species (**II**). Oxidative addition (OA) across the N−O bond of the *O*‐benzoylhydroxylamine then results in a Cu(III) intermediate (**III**), which can undergo reductive elimination (RE) to regenerate Cu(I) (**I**) and afford the desired *N*‐trifluoromethyl product.

## Conclusions

In summary, facilitated by the application of a high‐throughput screening approach, we have established a direct and practicable copper‐mediated electrophilic amination method for the synthesis of tertiary *N*‐trifluoromethylamines from *O*‐benzoylhydroxylamine substrates. Good to moderate yields were obtained with a range of structurally diverse molecular frameworks, with the developed method also tolerating a broad spectrum of additional functionality. Moreover, the requisite *N*−OBz substrates can be synthesised in a mild and operationally simple fashion. Based on this methodology now enabling more facile access to *N*‐trifluoromethylamines (and, indeed, carbamoyl fluorides), we envisage that the established protocols have the potential to inspire and facilitate future investigations into the properties of such *N*−CF_3_ compounds.

## Experimental Section

Full experimental details and compound characterization data are provided in the Supporting Information.

**General procedure for the preparation of** ***O*** **‐benzolyhydroxylamines**: Benzoic peroxyanhydride (Luperox A75, 75 %) (2 eq.) and cesium carbonate (3 eq.) were added to a round bottomed flask and stirred in DCM for 2 h at room temperature under a nitrogen atmosphere. To the reaction mixture was added amine (1 eq.) pre‐dissolved in DCM and the reaction mixture was stirred at room temperature for 21 h (reaction molarity=0.071–0.077 M). [Work‐up A: Water was added to the reaction mixture and this was stirred for 5 min and then extracted with DCM. The organic layer was washed with brine, dried through a hydrophobic frit, and concentrated *in vacuo* to give the crude product]. [Work up B: The reaction mixture was filtered through celite]. The crude product was preabsorbed onto celite (or dissolved in DCM) and purified by normal phase chromatography using a silica cartridge over 14 CV. The desired fractions were combined and evaporated *in vacuo* to give the *O*‐benzoylhydroxylamine products.

**General procedure for the preparation of** ***N*** **‐trifluoromethylamines 2 a–2 w**: *O*‐Benzoylhydroxylamine (0.322 mmol, 1 eq.), copper (I) bromide (7 mg, 0.049 mmol, 0.15 eq.), 4,4’‐di‐*tert*‐butyl‐2,2’‐bipyridine (13 mg, 0.048 mmol, 0.15 eq.) and strontium fluoride (81 mg, 0.644 mmol, 2 eq.) were added to a microwave vial and stirred in DMF (3 mL) at room temperature under a nitrogen atmosphere. To the reaction mixture was added trimethyl(trifluoromethyl)silane (95 μL, 0.644 mmol, 2 eq.) dropwise and the reaction mixture was stirred at 40 °C for 48 h.

Purification A: The reaction mixture was diluted with EtOAc (10 mL) and washed with 1 M Na_2_CO_3_ (or 1 M NaOH) aqueous solution (2×20 mL). The combined organics were then dried through a hydrophobic frit, absorbed onto celite (or evaporated *in vacuo* and then dissolved in DCM) and purified by normal phase chromatography using a silica cartridge over 14 CV. The desired fractions were combined and evaporated *in vacuo* to give the *N*‐trifluoromethyl products.

Purification B: The reaction mixture was blown‐down under a flow of nitrogen and then dissolved in EtOAc. The reaction mixture was preabsorbed onto celite and purified by normal phase chromatography using a 12 g silica cartridge over 14 CV. The desired fractions were combined and evaporated *in vacuo* to give the *N*‐trifluoromethyl products.

## Supporting Information

The authors have cited additional references within the Supporting Information.[^29^, ^30^]

## Conflict of interest

The authors declare no conflict of interest.

1

## Supporting information

As a service to our authors and readers, this journal provides supporting information supplied by the authors. Such materials are peer reviewed and may be re‐organized for online delivery, but are not copy‐edited or typeset. Technical support issues arising from supporting information (other than missing files) should be addressed to the authors.

Supporting Information

## Data Availability

The data that support the findings of this study are available in the supplementary material of this article.
