# Supplementary material for: Copper‐Mediated N‐Trifluoromethylation of O‐Benzoylhydroxylamines
Source: Chemistry. 2023 Dec 7;30(6):e202303314. doi: 10.1002/chem.202303314 (PMC10952365; doi:10.1002/chem.202303314)

Chemistry–A European Journal

Supporting Information

Copper-Mediated *N*-Trifluoromethylation of *O*-Benzoylhydroxylamines

Thomas D. Fleetwood, William J. Kerr,* and Joseph Mason*

Contents

1. General Information 2

2. Preparation of *O*-Benzoylhydroxylamines 5

3. Optimization Studies 44

4. General Procedure for the Trifluoromethylation of *O*-Benzoylhydroxylamines 55

5. Side Products and Unsuccessful Substrates 80

6. Mechanistic Control Reactions 86

7. Mechanistic Proposal 90

8. References 91

9. NMR Spectra of *O*-Benzoylhydroxylamines 92

10. NMR Spectra of *N*-Trifluoromethylamines 133

11. NMR Spectra of Isolated Side Products 169

1. General Information

All reagents were obtained from commercial suppliers and were used without further purification unless otherwise stated. Anhydrous solvents were obtained from Sigma-Aldrich and transferred from the Sure Seal™ bottles by syringe, under an atmosphere of nitrogen using standard Schlenk techniques. All reported compounds are at least 90% pure. These purity assessments are made using the UV absorption spectra collected as part of LCMS analysis and/or NMR.

^{1}H, ^{13}C{^{1}H} and ^{19}F/^{19}F{^{1}H} **NMR spectra** were recorded in either $CDCl_3$ or DMSO-d_6 on a Bruker AV-400 spectrometer (400 MHz, 101 MHz, and 376 MHz, respectively). Selected ^{1}H and ^{13}C{^{1}H} spectra were recorded using a Bruker AV-600 spectrometer (600 MHz and 151 MHz, respectively). Chemical shifts are reported in ppm and coupling constants (*J*) are reported in Hz. The internal standard used was either tetramethylsilane or the residual protonated solvent. The following abbreviations were used to explain the multiplicities: s = singlet, d = doublet, t = triplet, q = quartet, m = multiplet, br = broad, quint = quintet. **Infrared (IR) spectra** were obtained using a Perkin Elmer Spectrum Two™ FT-IR Spectrometer either from solids or by allowing a thin film of compound as a solution in $CDCl_3$ to evaporate. The absorption values are listed in units of wavenumbers (cm^{-1}) and the following abbreviations were used to explain the peak intensities: br = broad, w = weak, m = medium, s = strong. **LCMS** data were recorded using solutions in acetonitrile, methanol, or DMSO using either System A or B:

System A

Column: 50 mm x 2.1 mm ID, Acquity UPLC CSH C_{18} column.
Flow Rate: 1 mL/min
Temp: 40 °C
UV detection range: 210 to 350 nm
Mass spectrum: Recorded on a mass spectrometer using alternate-scan positive and negative mode electrospray ionisation.

Solvents: A: 0.1 % v/v solution of formic acid in water
B: 0.1 % v/v solution of formic acid in acetonitrile

Gradient:	*Time (min.)*	*A%*	*B%*
	0	97	3
	1.5	3	97
	1.9	3	97
	2.0	98	2

System B

Column: 50 mm x 2.1 mm ID, Acquity UPLC CSH C_{18} column.
Flow Rate: 1 mL/min
Temp: 40 °C
UV detection range: 210 to 350 nm
Mass spectrum: Recorded on a mass spectrometer using alternate-scan positive and negative mode electrospray ionisation.

Solvents: A: 10 mM solution of ammonium bicarbonate in water adjusted to pH 10 with ammonia solution.
B: Acetonitrile

Gradient:	*Time (min.)*	*A%*	*B%*
	0	97	3
	1.5	3	95
	1.9	3	95
	2.0	97	3

HRMS data were recorded using solutions in acetonitrile, methanol, or DMSO using a Waters XEVO® G2-XS QTof mass spectrometer using positive electrospray ionisation with a scan range of 100 to 1200 atomic mass units (AMU). **Thin layer chromatography (TLC)** was carried out using POLYGRAM® SIL G/UV_{254} plates with 0.2 mm silica gel and fluorescent indicator UV_{254} (available from Macherey-Nagel, Germany). TLC plates were analysed using UV irradiation and/or developed using potassium permanganate solution as appropriate. **Chromatographic purification** was performed using pre-packed silica gel cartridges (2 g to 220 g) on a Teledyne ISCO Combi*Flash*® Rf+ instrument. **Mass directed autopreparative (MDAP)** HPLC was undertaken using the conditions given below. The UV detection was an averaged signal from wavelength of 210 nm to 350 nm and mass spectra were recorded on a mass spectrometer using alternate-scan positive and negative mode electrospray ionisation:

Method A

Method A was conducted on an Xselect CSH C_{18} column (typically 150 mm x 30 mm i.d. 5 µm packing diameter) at ambient temperature. The solvents employed were:

A = 10 mM aqueous ammonium bicarbonate adjusted to pH 10 with ammonia solution.
B = Acetonitrile

Method B

Method B was conducted on a Xselect CSH C_{18} column (typically 150 mm x 30 mm i.d. 5 µm packing diameter) at ambient temperature. The solvents employed were:

A = 0.1 % v/v solution of formic acid in water
B = 0.1 % v/v solution of formic acid in acetonitrile.

GCMS data were recorded using solutions in acetonitrile, methanol, or DMSO on an Agilent Intuvo 9000 GC, 5977B GC/MSD using the following conditions: Total run time 14.4 min; Inlet temp 200 °C; Injection volume 0.5 µL; Flow rate 1.0 mL/min (He); Split ratio 50:1; Initial Oven temp 40 °C; Initial hold 2 min; Ramp rate 25 °C/min; Final Temp 300 °C; Final Hold 2 min; Column: 30 m x 0.25 mm ID x 0.25 micron; Column phase: HP5 MS UltraInert. **Melting points (M.pt.)** of solid compounds were determined using a Gallenkamp Melting Point Apparatus. **[α_D]** values were obtained using a Jasco P-1030 polarimeter. The light source was a sodium lamp (Na) with all measurements made at a wavelength of 589 nm.

2. Preparation of *O*-Benzoylhydroxylamines

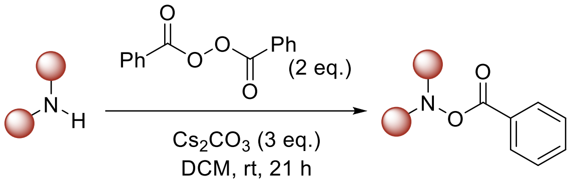

General procedure 2.1:[1]

Benzoic peroxyanhydride (Luperox A75, 75%) (2 eq.) and cesium carbonate (3 eq.) were added to a round bottomed flask and stirred in DCM for 2 h at room temperature under a nitrogen atmosphere. To the reaction mixture was added amine (1 eq.) pre-dissolved in DCM and the reaction mixture was stirred at room temperature for 21 h (reaction molarity = 0.071-0.077 M). [Work-up A : Water was added to the reaction mixture and the reaction mixture was stirred for 5 min and then extracted with DCM. The organic layer was washed with brine, dried through a hydrophobic frit and concentrated *in vacuo* to give the crude product]. [Work up B: The reaction mixture was filtered through celite]. The crude product was preabsorbed onto celite (or dissolved in DCM) and purified by normal phase chromatography using a silica cartridge over 14 CV. The desired fractions were combined and evaporated *in vacuo* to give the *O*-benzoylhydroxylamine products.

4-Benzoylpiperazin-1-yl benzoate (1a)

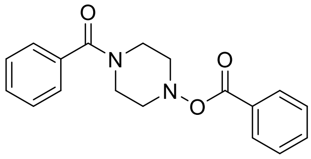

Compound synthesised following general procedure 2.1.

Amounts of each reagent used in this reaction:

Benzoic peroxyanhydride (Luperox A75, 75%) (3.40 g, 10.51 mmol, 2 eq.)

Cesium carbonate (5.14 g, 15.77 mmol, 3 eq.)

4-Benzoylpiperazin-1-yl benzoate (1 g, 5.26 mmol, 1 eq.)

DCM (initial mixture: 50 mL; Amine solution: 20 mL)

Work-up A; The product mixture was preabsorbed onto celite and purified by normal phase chromatography (50-100% EtOAc in cyclohexane) using a 80 g silica cartridge over 14 CV. The desired fractions were combined and evaporated *in vacuo* to give the desired product (**1a**) (1.52 g, 93%) as a white solid. **^{1}H NMR (400 MHz, DMSO-*d_6*):** δ 7.95-7.93 (m, 2H), 7.70-7.65 (m, 1H), 7.56-7.52 (m, 2H), 7.48-7.44 (m, 5H), 4.48-4.14 (m, 1H), 3.81-3.55 (m, 1H), 3.52-3.32 (m, 4H), 3.09-2.82 (m, 2H); **^{13}C NMR (151 MHz, DMSO-*d_6*):** δ 169.0, 163.6, 135.4, 133.5, 129.7, 129.0, 128.80, 128.76, 128.4, 126.9, 55.2, 45.2; **LCMS (System B):** t_R = 1.00 min. No m/z in keeping with the structure of the desired product observed; **HRMS (ESI$^+$):** $C_{18}H_{18}N_2NaO_3$ [M+Na]$^+$ requires 333.1210 found 333.1213; **IR (cm^{-1}):** 3063 (w, C-H), 2857 (w, C-H), 1732 (s, C=O), 1632 (s, C=O); **M.pt. (EtOAc/cyclohexane):** 134-136 °C.

***Tert*-Butyl 4-(benzoyloxy)piperazine-1-carboxylate (1b)**

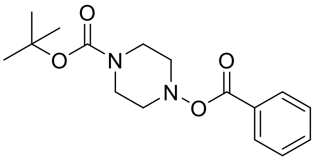

Compound synthesised following general procedure 2.1.

Amounts of each reagent used in this reaction:

Benzoic peroxyanhydride (Luperox A75, 75%) (3.47 g, 10.74 mmol, 2 eq.)

Cesium carbonate (5.25 g, 16.11 mmol, 3 eq.)

Tert-butyl piperazine-1-carboxylate (1 g, 5.37 mmol, 1 eq.)

DCM (initial mixture: 50 mL; Amine solution: 20 mL)

Work-up A; The product mixture was preabsorbed onto celite and purified by normal phase chromatography (0-100% EtOAc in cyclohexane) using a 80 g silica cartridge over 14 CV. The desired fractions were combined and evaporated *in vacuo* to give the desired product (**1b**) (1.10 g, 67%) as a white solid. **^{1}H NMR (600 MHz, DMSO-d_6):** δ 7.91-7.94 (m, 2H), 7.65-7.68 (m, 1H), 7.51-7.54 (m, 2H), 3.88 (s, 2H), 3.36 (br s, 2H), 3.16 (s, 2H), 2.82 (s, 2H), 1.42 (s, 9H); **^{13}C NMR (151 MHz, DMSO-d_6) (restricted rotation at the N-CO bond leads to separate signals observed for the piperazine carbons adjacent to this bond. This is observed for other similar compounds containing the N-CO bond):**[2] δ 163.5, 153.7, 133.4, 129.0, 128.8, 128.5, 79.2, 55.2, 42.0, 41.3, 28.0; **LCMS (System B):** t_R = 1.16 min. No m/z in keeping with the structure of the desired product observed; **HRMS (ESI$^+$):** $C_{16}H_{22}N_2NaO_4$ $[M+Na]^+$ requires 329.1472 found 329.1487; **IR (cm^{-1}):** 2977 (w, C-H), 1738 (s, C=O), 1691 (s, C=O), 1248.

4-(4-Methoxybenzoyl)piperazin-1-yl benzoate (1c)

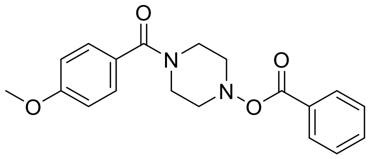

Compound synthesised following general procedure 2.1.

Amounts of each reagent used in this reaction:

Benzoic peroxyanhydride (Luperox A75, 75%) (5.86 g, 18.16 mmol, 2 eq.)

Cesium carbonate (8.87 g, 27.20 mmol, 3 eq.)

(4-Methoxyphenyl)(piperazin-1-yl)methanone (2 g, 9.08 mmol, 1 eq.)

DCM (initial mixture: 81 mL; Amine solution: 40 mL)

Work-up A; The product mixture was dissolved in DCM and purified by normal phase chromatography (50-100% EtOAc in cyclohexane) using a 80 g silica cartridge over 14 CV. The desired fractions were combined and evaporated *in vacuo* to give the desired product (**1c**) (1.94 g, 63%) as a colourless oil. **^{1}H NMR (600 MHz, DMSO-*d*$_6$):** δ 7.95-7.93 (m, 2H), 7.69-7.66 (m, 1H), 7.55-7.52 (m, 2H), 7.44-7.41 (m, 2H), 7.01-6.99 (m, 2H), 4.47-3.81 (m, 2H), 3.80 (s, 3H), 3.59-3.31 (m, 4H), 3.07-2.77 (m, 2H); **^{13}C NMR (151 MHz, DMSO-*d*$_6$):** δ 169.0, 163.5, 160.3, 133.5, 129.1, 129.0, 128.79, 128.76, 127.3, 113.7, 55.3, 55.2, 45.3; **LCMS (System B):** t_R = 1.00 min, m/z = No m/z in keeping with structure of desired product observed; **HRMS (ESI$^+$):** $C_{19}H_{21}N_2O_4$ [M+H]$^+$ requires 341.1496 found 341.1501; **IR (cm^{-1}):** 2848 (w, C-H), 1735 (s, C=O), 1631 (s, C=O), 1245.

_O_-Benzoyl-_N_-methyl-_N_-(1-(methylsulfonyl)piperidin-4-yl)hydroxylamine (1d)

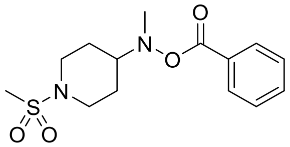

Compound synthesised following general procedure 2.1.

Amounts of each reagent used in this reaction:

Benzoic peroxyanhydride (Luperox A75, 75%) (3.36 g, 10.40 mmol, 2 eq.)

Cesium carbonate (5.08 g, 15.60 mmol, 3 eq.)

N-Methyl-1-(methylsulfonyl)piperidin-4-amine (1 g, 5.20 mmol, 1 eq.)

DCM (initial mixture: 50 mL; Amine solution: 20 mL)

Work-up A; The product mixture was preabsorbed onto celite and purified by normal phase chromatography (50-100% EtOAc in cyclohexane) using a 80 g silica cartridge over 14 CV. The desired fractions were combined and evaporated *in vacuo* to give the desired product (**1d**) (610 mg, 38%) as a white solid. **^{1}H NMR (600 MHz, DMSO-*d*$_6$):** δ 7.94-7.92 (m, 2H), 7.68-7.66 (m, 1H), 7.55-7.52 (m, 2H), 3.56 (dt, *J* = 12.1, 3.3 Hz, 2H), 2.96 (tt, *J* = 10.3, 4.0 Hz, 1H), 2.87 (s, 3H), 2.84-2.80 (m, 2H), 2.81 (s, 3H), 1.99-1.96 (m, 2H), 1.57-1.51 (m, 2H); **^{13}C NMR (151 MHz, DMSO-*d*$_6$):** δ 164.0, 133.4, 128.93, 128.85, 128.8, 63.1, 44.0, 42.9, 34.3, 27.6; **LCMS (System B):** t_R = 0.88 min, m/z = 313 (MH+); **HRMS (ESI$^+$):** $C_{14}H_{21}N_2O_4S$ $[M+H]^+$ requires 313.1217 found 313.1221; **IR (cm^{-1}):** 3007 (w, C-H), 1731 (s, C=O), 1332 (s, S=O), 1148; **M.pt. (EtOAc/cyclohexane):** 139-140 °C.

Benzyl 4-(benzoyloxy)-1,4-diazepane-1-carboxylate (1e)

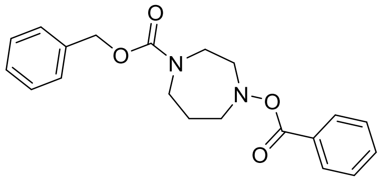

Compound synthesised following general procedure 2.1.

Amounts of each reagent used in this reaction:

Benzoic peroxyanhydride (Luperox A75, 75%) (5.51 g, 17.07 mmol, 2 eq.)

Cesium carbonate (8.34 g, 25.6 mmol, 3 eq.)

Benzyl 1,4-diazepane-1-carboxylate (2 g, 8.54 mmol, 1 eq.)

DCM (initial mixture: 76 mL; Amine solution: 38 mL)

Work-up A; The product mixture was dissolved in DCM and purified by normal phase chromatography (20-100% EtOAc in cyclohexane) using a 120 g silica cartridge over 14 CV. The desired fractions were combined and evaporated *in vacuo* to give the desired product (**1e**) (2.49 g, 82%) as a colourless oil. **^{1}H NMR (600 MHz, DMSO-*d*$_6$)**: δ 7.93-7.91 (m, 2H), 7.67-7.64 (m, 1H), 7.53-7.51 (m, 2H), 7.39-7.34 (m, 4H), 7.33-7.29 (m, 1H), 5.11-5.10 (m, 2H), 3.63-3.61 (m, 2H), 3.52 (dt, *J* = 14.1, 6.2 Hz, 2H), 3.31-3.29 (m, 2H), 3.26-3.23 (m, 2H), 1.96-1.92 (m, 2H); **^{13}C NMR (151 MHz, DMSO-*d*$_6$)** (rotameric signals observed): δ 163.57 (rot. A), 163.55 (rot. B), 155.3 (rot. A), 155.0 (rot. B), 137.0 (rot. A), 136.9 (rot. B), 133.3, 128.94, 128.92, 128.8, 128.4, 127.8, 127.44 (rot. A), 127.41 (rot. B), 66.23 (rot. A), 66.21 (rot. B), 58.2 (rot. A), 58.1 (rot. B), 57.2 (rot. A), 56.9 (rot. B), 44.7, 42.11 (rot. A), 42.09 (rot. B), 23.5 (rot. A), 23.4 (rot. B); **LCMS (System B):** t_R = 1.18 min, m/z = 355 (MH+); **HRMS (ESI$^+$):** $C_{20}H_{23}N_2O_4$ $[M+H]^+$ requires 355.1652 found 355.1655; **IR (cm^{-1}):** 2954 (w, C-H), 1736 (s, C=O), 1693 (s, C=O), 1241.

3-((1-Oxoisoquinolin-2(1*H*)-yl)methyl)piperidin-1-yl benzoate (1f)

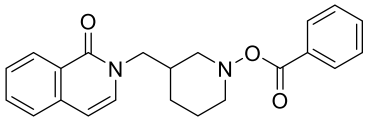

Compound synthesised following general procedure 2.1.

Amounts of each reagent used in this reaction:

Benzoic peroxyanhydride (Luperox A75, 75%) (4.63 g, 14.35 mmol, 2 eq.)

Cesium carbonate (7.01 g, 21.52 mmol, 3 eq.)

2-(Piperidin-3-ylmethyl)isoquinolin-1(2*H*)-one, hydrochloride (2 g, 7.17 mmol, 1 eq.)

DCM (initial mixture: 65 mL; Amine solution: 31 mL)

Work-up A; The product mixture was dissolved in DCM and purified by normal phase chromatography (50-100% EtOAc in cyclohexane) using a 120 g silica cartridge over 14 CV. The desired fractions were combined and evaporated *in vacuo* to give the desired product (**1f**) (1.99 g, 77%) as a white solid. **^{1}H NMR (600 MHz, DMSO-d_6):** δ 8.24-8.20 (m, 1H), 7.91-7.84 (m, 2H), 7.70-7.68 (m, 1H), 7.65-7.63 (m, 2H), 7.52-7.44 (m, 4H), 7.34 (d, *J* = 7.3 Hz, 1H), 4.21-3.81 (br m, 2H), 3.48-3.37 (br m, 1H), 3.36-2.99 (br m, 1H), 2.73-2.58 (br m, 1H), 2.57-2.44 (br m, 1H), 2.38-2.21 (br m, 1H), 1.83-1.77 (br m, 1H), 1.67-1.53 (br m, 2H), 1.46-0.97 (br m, 1H); **^{13}C NMR (151 MHz, DMSO-d_6):** δ 163.6, 161.1, 136.9, 133.3, 133.3, 132.2, 128.94, 128.87, 128.7, 127.0, 126.6, 126.1, 125.3, 104.9, 60.0, 56.6, 51.1, 36.5, 26.9, 23.8; **LCMS (System B):** t_R = 1.15 min, m/z = 363 (MH+); **HRMS (ESI$^+$):** $C_{22}H_{23}N_2O_3$ $[M+H]^+$ requires 363.1703 found 363.1710; **IR (cm^{-1}):** 2952 (w, C-H), 1733 (s, C=O), 1646 (s, C=O), 1250.

***Tert*-butyl 1'-(benzoyloxy)-3*H*-spiro[benzo[*f*][1,4]oxazepine-2,4'-piperidine]-4(5*H*)-carboxylate (1g)**

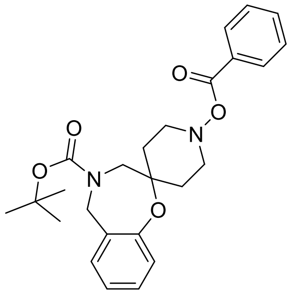

Compound synthesised following general procedure 2.1.

Amounts of each reagent used in this reaction:

Benzoic peroxyanhydride (Luperox A75, 75%) (4.06 g, 12.56 mmol, 2 eq.)

Cesium carbonate (6.14 g, 18.84 mmol, 3 eq.)

Tert-butyl 3*H*-spiro[benzo[f][1,4]oxazepine-2,4'-piperidine]-4(5*H*)-carboxylate (2 g, 6.28 mmol, 1 eq.)

DCM (initial mixture: 57 mL; Amine solution: 27 mL)

Work-up A; The product mixture was preabsorbed onto celite and purified by normal phase chromatography (0-100% EtOAc in cyclohexane) using a 80 g silica cartridge over 14 CV. The desired fractions were combined and evaporated *in vacuo* to give the desired product (**1g**) (2.32 g, 84%) as a white solid. **^{1}H NMR (500 MHz, 380 K, DMSO-*d*$_6$):** δ 7.95-7.93 (m, 2H), 7.66-7.63 (m, 1H), 7.54-7.51 (m, 2H), 7.28-7.24 (m, 2H), 7.14 (d, *J* = 7.3 Hz, 1H), 7.09-7.06 (m, 1H), 4.42 (s, 2H), 3.65 (s, 2H), 3.30-3.28 (m, 4H), 1.87-1.81 (m, 2H), 1.77-1.74 (m, 2H), 1.42 (s, 9H); **^{13}C NMR (126 MHz, 380 K, DMSO-*d*$_6$):** δ 163.2, 153.6, 153.4, 132.4, 131.2, 129.0, 128.6, 128.2, 128.0, 127.9, 122.9, 122.4, 78.8, 76.6, 55.0, 50.7, 49.2, 29.8, 27.5; **LCMS (System B):** t_R = 1.38 min, m/z = 439 (MH+); **HRMS (ESI$^+$):** $C_{25}H_{31}N_2O_5$ $[M+H]^+$ requires 439.2227 found 439.2230; **IR (cm^{-1}):** 2928 (w, C-H), 1738 (s, C=O), 1692 (s, C=O), 1241.

O-Benzoyl-N-methyl-N-(2-(thiophen-2-yl)benzyl)hydroxylamine (1h)

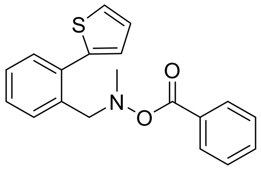

Compound synthesised following general procedure 2.1.

Amounts of each reagent used in this reaction:

Benzoic peroxyanhydride (Luperox A75, 75%) (794 mg, 2.459 mmol, 2 eq.)

Cesium carbonate (1202 mg, 3.69 mmol, 3 eq.)

N-Methyl-1-(2-(thiophen-2-yl)phenyl)methanamine (250 mg, 1.230 mmol, 1 eq.)

DCM (initial mixture: 11 mL; Amine solution: 5.5 mL)

Work-up A; The product mixture was dissolved in DCM and purified by normal phase chromatography (0-10% EtOAc in cyclohexane) using a 24 g silica cartridge over 14 CV. The desired fractions were combined and evaporated *in vacuo* to give the desired product (**1h**) (146 mg, 37%) as a colourless oil. **^{1}H NMR (600 MHz, DMSO-*d*$_6$):** δ 7.79-7.78 (m, 2H), 7.65-7.60 (m, 3H), 7.48-7.46 (m, 2H), 7.42-7.39 (m, 2H), 7.32-7.28 (m, 2H), 7.19-7.17 (m, 1H), 4.20 (s, 2H), 2.88 (s, 3H); **^{13}C NMR (151 MHz, DMSO-*d*$_6$):** δ 163.9, 141.1, 134.1, 134.0, 133.3, 130.5, 130.3, 128.8, 128.72, 128.69, 128.0, 127.68, 127.65, 127.6, 126.6, 61.6, 46.2; **LCMS (System B):** t_R = 1.38 min, m/z = 324 (MH+); **HRMS (ESI$^+$):** $C_{19}H_{18}NO_2S$ $[M+H]^+$ requires 324.1053 found 324.1063; **IR (cm^{-1}):** 3066 (w, C-H), 2876 (w, C-H), 1734 (s, C=O), 1247.

***Tert*-butyl 2-(benzoyloxy)-2,3-dihydro-1*H*-spiro[isoquinoline-4,4'-piperidine]-1'-carboxylate (1i)**

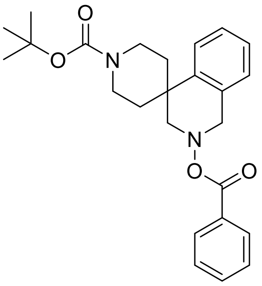

Compound synthesised following general procedure 2.1.

Amounts of each reagent used in this reaction:

Benzoic peroxyanhydride (Luperox A75, 75%) (3.81 g, 11.80 mmol, 2 eq.)

Cesium carbonate (5.77 g, 17.71 mmol, 3 eq.)

Tert-butyl 2,3-dihydro-1*H*-spiro[isoquinoline-4,4'-piperidine]-1'-carboxylate, hydrochloride (2 g, 5.90 mmol, 1 eq.)

DCM (initial mixture: 53 mL; Amine solution: 26 mL)

Work-up A; The product mixture was dissolved in DCM and purified by normal phase chromatography (0-100% EtOAc in cyclohexane) using a 120 g silica cartridge over 14 CV. The desired fractions were combined and evaporated *in vacuo* to give the desired product (**1i**) (2.04 g, 82%) as a white solid. **^{1}H NMR (400 MHz, $CDCl_3$):** δ 8.07-8.04 (m, 2H), 7.62-7.58 (m, 1H), 7.50-7.45 (m, 2H), 7.36-7.34 (m, 1H), 7.30-7.26 (m, 1H), 7.22-7.18 (m, 1H), 7.09-7.07 (m, 1H), 4.52-4.31 (br m, 2H), 4.25-4.03 (br m, 2H), 3.25-2.84 (br m, 2H), 2.29-1.75 (br m, 4H), 1.50 (s, 9H), 1.44 (br s, 2H); **^{13}C NMR (101 MHz, $CDCl_3$):** δ 164.7, 155.0, 141.4, 133.1, 132.2, 129.4, 129.3, 128.5, 127.4, 127.0, 126.6, 125.7, 79.5, 60.2, 59.8, 40.0 (br), 39.7, 36.2, 28.5, 26.9; **LCMS (System B):** t_R = 1.46 min, m/z = No m/z in keeping with structure of desired product observed; **HRMS (ESI$^+$):** $C_{25}H_{30}N_2NaO_4$ $[M+Na]^+$ requires 445.2098 found 445.2102; **IR (cm^{-1}):** 2924 (w, C-H), 1737 (s, C=O), 1687 (s, C=O), 1242.

2-Methyl-2-(*m*-tolyl)morpholino benzoate (1j)

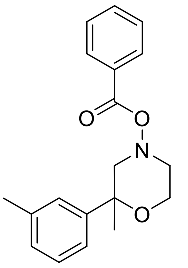

Compound synthesised following general procedure 2.1.

Amounts of each reagent used in this reaction:

Benzoic peroxyanhydride (Luperox A75, 75%) (5.67 g, 17.6 mmol, 2 eq.)

Cesium carbonate (8.58 g, 26.3 mmol, 3 eq.)

2-Methyl-2-(*m*-tolyl)morpholine, hydrochloride (2 g, 8.78 mmol, 1 eq.)

DCM (initial mixture: 78 mL; Amine solution: 39 mL)

Work-up A; The product mixture was dissolved in DCM and purified by normal phase chromatography (0-10% TBME in cyclohexane) using a 120 g silica cartridge over 14 CV. The desired fractions were combined and evaporated *in vacuo* to give a colourless oil. LCMS showed that there was benzoyl peroxide still present. The sample was dissolved in DCM and purified by normal phase chromatography (0-10% TBME in cyclohexane) using an 80 g silica cartridge over 14 CV. The desired fractions were combined and evaporated *in vacuo* to give the desired product (**1j**) (1.90 g, 70%) as a colourless oil. **^{1}H NMR (400 MHz, 393 K, DMSO-*d*$_6$):** δ 7.91-7.89 (m, 2H), 7.66-7.62 (m, 1H), 7.53-7.49 (m, 2H), 7.42-7.37 (m, 2H), 7.25 (t, J = 7.6 Hz, 1H), 7.08 (d, J = 7.6 Hz, 1H), 3.90-3.85 (m, 1H), 3.83-3.75 (br m, 1H), 3.74-3.66 (m, 1H), 3.20-3.13 (m, 3H), 2.34 (br s, 3H), 1.48 (br s, 3H); **^{13}C NMR (101 MHz, DMSO-*d*$_6$):** δ 163.7, 142.9, 137.1, 133.4, 128.9, 128.8, 128.7, 128.0, 127.5, 127.1, 123.6, 77.8, 62.7, 60.8, 56.1, 30.8, 21.2; **LCMS (System B):** t_R = 1.35 min, m/z = 312 (MH+); **HRMS (ESI$^+$):** $C_{19}H_{21}NNaO_3$ [M+Na]$^+$ requires 334.1414 found 334.1426; **IR (cm^{-1}):** 2974 (w, C-H), 2847 (w, C-H), 1734 (s, C=O), 1243.

6-Chloro-7,8-dimethoxy-1-(4-methoxyphenyl)-1,2,4,5-tetrahydro-3*H*-benzo[*d*]azepin-3-yl benzoate (1k)

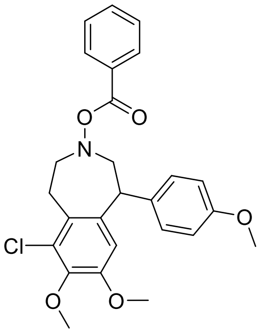

Compound synthesised following general procedure 2.1.

Amounts of each reagent used in this reaction:

Benzoic peroxyanhydride (Luperox A75, 75%) (3.71 g, 11.5 mmol, 2 eq.)

Cesium carbonate (5.62 g, 17.2 mmol, 3 eq.)

6-Chloro-7,8-dimethoxy-1-(4-methoxyphenyl)-2,3,4,5-tetrahydro-1*H*-benzo[*d*]azepine (2 g, 5.75 mmol, 1 eq.)

DCM (initial mixture: 52 mL; Amine solution: 25 mL)

Work-up A; The product mixture was dissolved in DCM and purified by normal phase chromatography (0-50% EtOAc in cyclohexane) using a 120 g silica cartridge over 14 CV. The desired fractions were combined and evaporated *in vacuo* to give the desired product (**1k**) (2.55 g, 95%) as a white solid. **^{1}H NMR (400 MHz, 393 K, DMSO-*d*$_6$):** δ 7.92-7.90 (m, 2H), 7.65-7.61 (m, 1H), 7.52-7.48 (m, 2H), 7.30-7.27 (m, 2H), 6.95-6.91 (m, 2H), 6.54 (s, 1H), 4.62 (br d, *J* = 7.8, 1H), 3.86 (dd, *J* = 12.0, 7.8 Hz, 1H), 3.79-3.78 (m, 6H), 3.68 (s, 3H), 3.59 (br dd, *J* = 12.0, 1.7 Hz, 1H), 3.41-3.34 (m, 2H), 3.32-3.26 (m, 1H), 3.13-3.07 (m, 1H); **^{13}C NMR (101 MHz, DMSO-*d*$_6$):** δ 163.5, 157.7, 150.9, 143.0, 140.6, 133.3, 133.1, 130.1, 129.2, 129.0, 128.9, 128.7, 127.3, 113.7, 112.8 (br), 61.2 (br), 59.9, 56.7, 55.8, 55.0, 45.4 (br), 25.6; **LCMS (System B):** t_R = 1.46 min, m/z = 468 (MH+); **HRMS (ESI$^+$):** $C_{26}H_{27}ClNO_5$ $[M+H]^+$ requires 468.1572 found 468.1579; **IR (cm^{-1}):** 2933 (w, C-H), 2835 (w, C-H), 1735 (s, C=O), 1598; **M.pt. (EtOAc/cyclohexane):** 79-83 °C.

4-(Furan-2-carbonyl)piperazin-1-yl benzoate (1l)

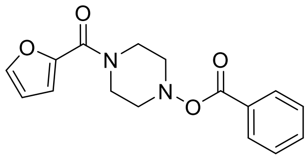

Compound synthesised following general procedure 2.1.

Amounts of each reagent used in this reaction:

Benzoic peroxyanhydride (Luperox A75, 75%) (7.17 g, 22.2 mmol, 2 eq.)

Cesium carbonate (10.8 g, 33.3 mmol, 3 eq.)

Furan-2-yl(piperazin-1-yl)methanone (2 g, 11.1 mmol, 1 eq.)

DCM (initial mixture: 99 mL; Amine solution: 49 mL)

Work-up A; The product mixture was dissolved in DCM and purified by normal phase chromatography (50-100% EtOAc in cyclohexane) using a 120 g silica cartridge over 14 CV. The desired fractions were combined and evaporated *in vacuo* to give the desired product (**1l**) (2.50 g, 75%) as an off-white solid. **^{1}H NMR (400 MHz, DMSO-d_6):** δ 7.96-7.94 (m, 2H), 7.86 (dd, *J* = 1.8, 0.7 Hz, 1H), 7.70-7.65 (m, 1H), 7.56-7.51 (m, 2H), 3.42 (dd, *J* = 3.5, 0.7 Hz, 1H), 6.64 (dd, *J* = 3.5, 1.8 Hz 1H), 4.49-4.08 (br m, 2H), 3.72-3.33 (br m, 4H), 3.17-2.76 (br m, 2H); **^{13}C NMR (151 MHz, DMSO-d_6):** δ 163.6, 158.3, 146.7, 144.9, 133.5, 129.0, 128.80, 128.75, 116.0, 111.4, 55.4, 42.3 (br); **LCMS (System B):** t_R = 0.94 min, m/z = No m/z in keeping with structure of desired product observed; **HRMS (ESI$^+$):** $C_{16}H_{16}N_2NaO_4$ $[M+Na]^+$ requires 323.1002 found 323.1013; **IR (cm^{-1}):** 2854 (w, C-H), 1732 (s, C=O), 1611 (s, C=O), 1252; **M.pt. (EtOAc/cyclohexane):** 88-91 °C.

Ethyl 1-(benzoyloxy)-4-phenylpiperidine-3-carboxylate; *trans*:*cis* 80:20 (1m)

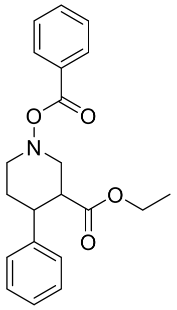

Compound synthesised following general procedure 2.1.

Amounts of each reagent used in this reaction:

Benzoic peroxyanhydride (Luperox A75, 75%) (5.54 g, 17.1 mmol, 2 eq.)

Cesium carbonate (8.38 g, 25.7 mmol, 3 eq.)

Ethyl 4-phenylpiperidine-3-carboxylate; *trans*:*cis* 81:19 (2 g, 8.57 mmol, 1 eq.)

DCM (initial mixture: 76 mL; Amine solution: 38 mL)

Work-up A; The product mixture was dissolved in DCM and purified by normal phase chromatography (0-50% TBME in cyclohexane) using an 80 g silica cartridge over 14 CV. The desired fractions were combined and evaporated *in vacuo* to give a colourless oil. LCMS showed that there was benzoyl peroxide still present. The sample was dissolved in DCM and purified by normal phase chromatography (0-50% TBME in cyclohexane) using an 80 g silica cartridge over 14 CV. The desired fractions were combined and evaporated *in vacuo* to give the desired product (**1m**) (80:20 *trans*:*cis* mixture, 1.47 g, 49%) as a colourless oil. **^{1}H NMR (400 MHz, DMSO-*d*$_6$):** δ 8.07-7.90 (br m, 2H), 7.70-7.64 (m, 1H), 7.58-7.51 (m, 2H), 7.35-7.16 (m, 5H), 3.87-3.80 (m, 2H), 3.70-3.68 (m, 1H), 3.57-3.54 (m, 1H), 3.25-2.62 (br m, 4H), 2.19-1.54 (br, m, 2H), 0.87 (t, *J* = 6.9 Hz, 3H); **^{13}C NMR (151 MHz, DMSO-*d*$_6$)** (Diastereomeric signals observed): δ 172.4 (br), 171.0 (br), 163.6, 163.5, 143.2, 142.3, 133.43, 133.36, 129.0, 128.9, 128.8, 128.7, 128.3, 127.8, 127.3, 126.6, 125.9, 59.9, 59.4, 58.2 (br), 56.4 (br), 47.9 (br), 43.8, 32.1 (br), 13.7; **LCMS (System B):** t_R = 1.29 min, m/z = 354 (MH+); **HRMS (ESI$^+$):** $C_{21}H_{24}NO_4$ [M+H]$^+$ requires 354.1700 found 354.1705; **IR (cm^{-1}):** 2978 (w, C-H), 2845 (w, C-H), 1728 (s, C=O), 1240.

3-Phenyl-1-oxa-2,8-diazaspiro[4.5]dec-2-en-8-yl benzoate (1n)

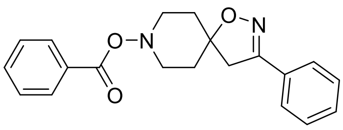

Compound synthesised following general procedure 2.1.

Amounts of each reagent used in this reaction:

Benzoic peroxyanhydride (Luperox A75, 75%) (5.97 g, 18.5 mmol, 2 eq.)

Cesium carbonate (9.04 g, 27.7 mmol, 3 eq.)

3-Phenyl-1-oxa-2,8-diazaspiro[4.5]dec-2-ene (2 g, 9.25 mmol, 1 eq.)

DCM (initial mixture: 82 mL; Amine solution: 41 mL)

Work-up A; The product mixture was dissolved in DCM and purified by normal phase chromatography (0-100% EtOAc in cyclohexane) using a 120 g silica cartridge over 14 CV. The desired fractions were combined and evaporated *in vacuo* to give the desired product (**1n**) (2.14 g, 69%) as a white solid. **^{1}H NMR (600 MHz, DMSO-*d*$_6$):** δ 7.95-7.94 (m, 2H), 7.68-7.66 (m, 3H), 7.55-7.53 (m, 2H), 7.47-7.44 (m, 3H), 3.49-3.34 (br m, 2H), 3.28 (br s, 2H), 3.21-3.01 (br m, 2H), 2.12-1.86 (br m, 4H); **^{13}C NMR (151 MHz, DMSO-*d*$_6$))**: δ 163.7, 156.6 (br), 133.3, 129.9, 129.7, 129.0, 128.9, 128.8, 128.7, 126.4, 84.1 (br), 82.6 (br) (duplicated signal caused by slow conformational interconversion of the 6-membered ring), 52.8, 43.8 (br), 33.9 (br), 32.9 (br); **LCMS (System B):** t_R = 1.23 min, m/z = 337 (MH+); **HRMS (ESI$^+$):** $C_{20}H_{21}N_2O_3$ [M+H]$^+$ requires 337.1547 found 337.1555; **IR (cm^{-1}):** 3063 (w, C-H), 2846 (w, C-H), 1733 (s, C=O), 1250; **M.pt. (EtOAc/cyclohexane):** 131-133 °C.

2-Oxo-3-(pyridin-3-ylmethyl)-1-oxa-3,8-diazaspiro[4.5]decan-8-yl benzoate (1o)

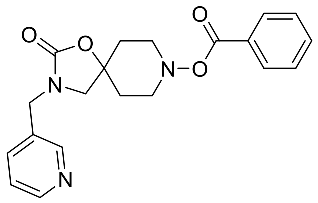

Compound synthesised following general procedure 2.1.

Amounts of each reagent used in this reaction:

Benzoic peroxyanhydride (Luperox A75, 75%) (5.22 g, 16.2 mmol, 2 eq.)

Cesium carbonate (7.91 g, 24.3 mmol, 3 eq.)

3-(Pyridin-3-ylmethyl)-1-oxa-3,8-diazaspiro[4.5]decan-2-one (2 g, 8.09 mmol, 1 eq.)

DCM (initial mixture: 72 mL; Amine solution: 36 mL)

Work-up A; The product mixture was preabsorbed onto celite and purified by normal phase chromatography (50-100% 3:1 EtOAc:EtOH in cyclohexane) using an 80 g silica cartridge over 14 CV. The desired fractions were combined and evaporated *in vacuo* to give the desired product (**1o**) (676 mg, 23%) as a white solid. **^{1}H NMR (400 MHz, 393 K, DMSO-d_6):** δ 8.53-8.52 (m, 2H), 7.93-7.91 (m, 2H), 7.71-7.69 (m, 1H), 7.66-7.62 (m, 1H), 7.53-7.49 (m, 2H), 7.38 (dd, J = 7.8, 4.7 Hz, 1H), 4.43 (s, 2H), 3.35 (s, 2H), 3.28-3.19 (m, 4H), 2.05-1.95 (m, 4H); **^{13}C NMR (101 MHz, DMSO-d_6):** δ 163.6, 156.3, 149.0, 148.8, 135.5, 133.3, 132.0, 128.9, 128.7, 128.5, 123.7, 75.4 (br), 53.4 (br), 51.9 (br), 44.8, 33.9 (br); **LCMS (System B):** t_R = 0.85 min, m/z = 368 (MH+); **HRMS (ESI$^+$):** $C_{20}H_{22}N_3O_4$ [M+H]$^+$ requires 368.1605 found 368.1612; **IR (cm^{-1}):** 2932 (w, C-H), 2865 (w, C-H), 1740 (s, C=O), 1728 (s, C=O); **M.pt. (3:1 EtOAc:EtOH/cyclohexane):** 167-170 °C.

4-(3-Nitropyridin-2-yl)piperazin-1-yl benzoate (1p)

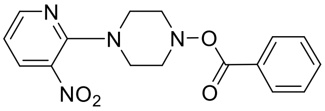

Compound synthesised following general procedure 2.1.

Amounts of each reagent used in this reaction:

Benzoic peroxyanhydride (Luperox A75, 75%) (6.20 g, 19.2 mmol, 2 eq.)

Cesium carbonate (9.39 g, 28.8 mmol, 3 eq.)

1-(3-Nitropyridin-2-yl)piperazine (2 g, 9.61 mmol, 1 eq.)

DCM (initial mixture: 86 mL; Amine solution: 42 mL)

Work-up A; The product mixture was dissolved in DCM and purified by normal phase chromatography (0-100% EtOAc in cyclohexane) using an 80 g silica cartridge over 14 CV. The desired fractions were combined and evaporated *in vacuo* to give the desired product (**1p**) (246 mg, 8%) as a yellow solid. **^{1}H NMR (400 MHz, DMSO-*d*$_6$):** δ 8.45 (dd, *J* = 4.7, 2.0 Hz, 1H), 8.30 (dd, *J* = 7.9, 2.0 Hz, 1H), 7.96-7.93 (m, 2H), 7.69-7.65 (m, 1H), 7.55-7.51 (m, 2H), 6.98 (dd, *J* = 7.9, 4.7 Hz, 1H), 3.93-3.66 (br m, 2H), 3.54-3.34 (br m, 4H), 3.19-2.89 (br m, 2H); **^{13}C NMR (101 MHz, DMSO-*d*$_6$):** δ 163.5, 152.1, 151.6, 135.9, 133.4, 132.9, 129.2, 129.0, 128.8, 114.6, 55.1, 45.7; **LCMS (System B):** t_R = 1.13 min, m/z = 329 (MH+); **HRMS (ESI$^+$):** $C_{16}H_{16}N_4NaO_4$ $[M+Na]^+$ requires 351.1064 found 351.1074; **IR (cm^{-1}):** 3062 (w, C-H), 2848 (w, C-H), 1737 (s, C=O), 1594 (s, N-O); **M.pt. (EtOAc/cyclohexane):** 119-121 °C.

(1*R*,3*s*,5*S*)-3-(4-Chloro-3-methoxyphenyl)-8-azabicyclo[3.2.1]octan-8-yl benzoate (1q)

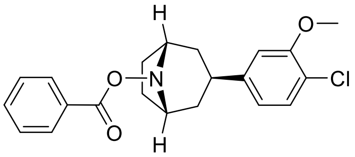

Compound synthesised following general procedure 2.1.

Amounts of each reagent used in this reaction:

Benzoic peroxyanhydride (Luperox A75, 75%) (4.48 g, 13.9 mmol, 2 eq.)

Cesium carbonate (6.78 g, 20.8 mmol, 3 eq.)

(1*R*,3*s*,5*S*)-3-(4-Chloro-3-methoxyphenyl)-8-azabicyclo[3.2.1]octane, hydrochloride (2 g, 6.94 mmol, 1 eq.)

DCM (initial mixture: 62 mL; Amine solution: 31 mL)

Work-up A; The product mixture was dissolved in DCM and purified by normal phase chromatography (0-20% EtOAc in cyclohexane) using an 80 g silica cartridge over 14 CV. The desired fractions were combined and evaporated *in vacuo* to give the desired product (**1q**) (2.26 g, 88%) as a white solid. **^{1}H NMR (400 MHz, DMSO-*d*$_6$):** δ 7.89-7.84 (m, 2H), 7.66-7.62 (m, 1H), 7.54-7.49 (m, 2H), 7.32 (d, *J* = 8.2 Hz, 1H), 7.14 (d, *J* = 1.6 Hz, 1H), 7.02 (dd, *J* = 8.2, 1.6 Hz, 1H), 3.89-3.83 (br m, 5H), 3.06 (quin, *J* = 6.9 Hz, 1H), 2.48-2.45 (m, 2H) (overlap with DMSO-*d*$_6$ signal), 2.14-2.03 (m, 4H), 1.64-1.59 (m, 2H); **^{13}C NMR (101 MHz, DMSO-*d*$_6$):** δ 163.7, 154.1, 145.6, 133.1, 129.4, 129.1, 128.8, 128.7, 119.5, 118.3, 111.8, 62.6, 56.0, 36.8, 30.4, 27.8; **LCMS (System B):** t_R = 1.40 min, m/z = 372 (MH+); **HRMS (ESI$^+$):** $C_{21}H_{23}ClNO_3$ $[M+H]^+$ requires 372.1361 found 372.1371; **IR (cm^{-1}):** 2970 (w, C-H), 2942 (w, C-H), 1733 (s, C=O), 1248; **M.pt. (EtOAc/cyclohexane):** 120-122 °C; **$[\alpha_D]^{16\ °C}$ (*c* 1, CDCl3):** -1.2 °.

7-(Methylsulfonyl)-1,2,4,5-tetrahydro-3*H*-benzo[*d*]azepin-3-yl benzoate (1r)

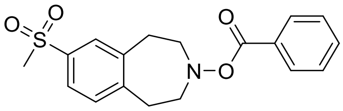

Compound synthesised following general procedure 2.1.

Amounts of each reagent used in this reaction:

Benzoic peroxyanhydride (Luperox A75, 75%) (5.73 g, 17.8 mmol, 2 eq.)

Cesium carbonate (8.68 g, 26.6 mmol, 3 eq.)

7-(Methylsulfonyl)-2,3,4,5-tetrahydro-1*H*-benzo[*d*]azepine (2 g, 8.88 mmol, 1 eq.)

DCM (initial mixture: 79 mL; Amine solution: 39 mL)

Work-up A; The product mixture was dissolved in DCM and purified by normal phase chromatography (0-100% EtOAc in cyclohexane) using an 80 g silica cartridge over 14 CV. The desired fractions were combined and evaporated *in vacuo* to give the desired product (**1r**) (2.44 g, 80%) as an off-white solid. **^{1}H NMR (400 MHz, DMSO-*d*$_6$):** δ 7.98-7.95 (m, 2H), 7.77 (d, *J* = 1.7 Hz, 1H), 7.71 (dd, *J* = 7.9, 1.7 Hz, 1H), 7.69-7.64 (m, 1H), 7.55-7.51 (m, 2H), 7.47 (d, *J* = 7.9 Hz, 1H), 3.40-3.24 (br m, 4H) (overlap with water signal), 3.23-3.06 (br m, 7H); **^{13}C NMR (101 MHz, DMSO-*d*$_6$):** δ 163.4, 147.1, 142.3, 138.9, 133.3, 129.9, 129.02, 128.96, 128.7, 127.1, 125.1, 57.1, 56.8, 43.6, 31.3, 31.2; **LCMS (System B):** t_R = 1.00 min, m/z = 346 (MH+); **HRMS (ESI$^+$):** $C_{18}H_{20}NO_4S$ $[M+H]^+$ requires 346.1108 found 346.1120; **IR (cm^{-1}):** 2965 (w, C-H), 2926 (w, C-H), 1725 (s, C=O), 1289 (s, S=O); **M.pt. (DCM):** 151-154 °C.

Benzyl 4-(benzoyloxy)-3-methylpiperazine-1-carboxylate (1s)

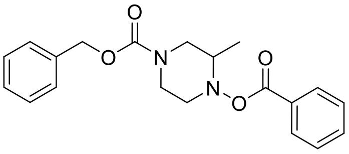

Compound synthesised following general procedure 2.1.

Amounts of each reagent used in this reaction:

Benzoic peroxyanhydride (Luperox A75, 75%) (5.51 g, 17.1 mmol, 2 eq.)

Cesium carbonate (8.34 g, 25.6 mmol, 3 eq.)

Benzyl 3-methylpiperazine-1-carboxylate (2 g, 8.54 mmol, 1 eq.)

DCM (initial mixture: 76 mL; Amine solution: 38 mL)

Work-up A; The product mixture was dissolved in DCM and purified by normal phase chromatography (0-50% EtOAc in cyclohexane) using an 80 g silica cartridge over 14 CV. The desired fractions were combined and evaporated *in vacuo* to give the desired product (**1s**) (2.42 g, 80%) as a light yellow oil. **^{1}H NMR (400 MHz, DMSO-d_6):** δ 7.95-7.94 (br m, 2H), 7.69-7.65 (m, 1H), 7.56-7.51 (m, 2H), 7.40-7.36 (m, 4H), 7.35-7.30 (m, 1H), 5.15-5.08 (m, 2H), 4.13-3.56 (br m, 2H), 3.41 (br d, J = 9.8 Hz, 1H), 3.28-3.09 (br m, 1H), 3.06-2.70 (br m, 3H), 1.06 (d, J = 5.6 Hz, 3H); **^{13}C NMR (101 MHz, DMSO-d_6):** δ 163.9, 154.1, 136.7, 133.4, 129.0, 128.8, 128.6, 128.4, 127.8, 127.6, 66.4, 60.0, 55.5, 48.4, 42.6, 16.0; **LCMS (System B):** t_R = 1.20 min, m/z = 355 (MH+); **HRMS (ESI$^+$):** $C_{20}H_{22}N_2NaO_4$ $[M+Na]^+$ requires 377.1472 found 377.1468; **IR (cm^{-1}):** 3064 (w, C-H), 2859 (w, C-H), 1741 (s, C=O), 1698 (s, C=O); **M.pt. (DCM):** 151-154 °C.

4-(3-Cyanopyrazin-2-yl)piperazin-1-yl benzoate (1t)

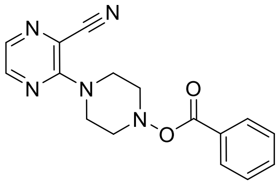

Compound synthesised following general procedure 2.1.

Amounts of each reagent used in this reaction:

Benzoic peroxyanhydride (Luperox A75, 75%) (6.83 g, 21.1 mmol, 2 eq.)

Cesium carbonate (10.3 g, 31.7 mmol, 3 eq.)

3-(Piperazin-1-yl)pyrazine-2-carbonitrile (2 g, 10.6 mmol, 1 eq.)

DCM (initial mixture: 94 mL; Amine solution: 47 mL)

Work-up A; The product mixture was dissolved in DCM and purified by normal phase chromatography (0-80% EtOAc in cyclohexane) using an 120 g silica cartridge over 14 CV. The desired fractions were combined and evaporated *in vacuo* to give the desired product (**1t**) (2.04 g, 62%) as a brown solid. **^{1}H NMR (400 MHz, DMSO-*d*$_6$):** δ 8.48 (d, *J* = 2.0 Hz, 1H), 8.17 (d, *J* = 2.0 Hz, 1H), 7.97-7.94 (m, 2H), 7.69-7.65 (m, 1H), 7.55-7.51 (m, 2H), 4.52-4.12 (br m, 2H), 3.85-3.35 (br m, 4H), 3.25-2.92 (br m, 2H); **^{13}C NMR (151 MHz, DMSO-*d*$_6$):** δ 163.6, 156.1, 145.7, 135.5, 133.5, 129.0, 128.78, 128.76, 117.0, 114.9, 55.0, 45.0; **LCMS (System B):** t_R = 1.03 min, m/z = 310 (MH+); **HRMS (ESI$^+$):** $C_{16}H_{15}N_5NaO_2$ [M+Na]$^+$ requires 332.1118 found 332.1115; **IR (cm^{-1}):** 2904 (w, C-H), 2854 (w, C-H), 2223 (w, C≡N), 1720 (s, C=O); **M.pt. (EtOAc/cyclohexane):** 127-130 °C.

4-(Quinoxaline-6-carbonyl)piperazin-1-yl benzoate (1u)

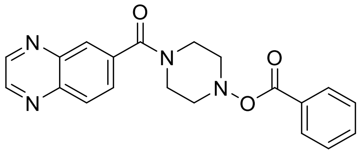

Compound synthesised following general procedure 2.1.

Amounts of each reagent used in this reaction:

Benzoic peroxyanhydride (Luperox A75, 75%) (1.41 g, 4.37 mmol, 2 eq.)

Cesium carbonate (2.14 g, 6.55 mmol, 3 eq.)

Piperazin-1-yl(quinoxalin-6-yl)methanone, hydrochloride (609 mg, 2.18 mmol, 1 eq.)

DCM (initial mixture: 19 mL; Amine solution: 10 mL)

Work-up A; The product mixture was dissolved in DCM and purified by normal phase chromatography (80-100% EtOAc in cyclohexane) using a 40 g silica cartridge over 14 CV. The desired fractions were combined and evaporated *in vacuo* to give the desired product (**1u**) (613 mg, 78%) as a white solid. **^{1}H NMR (400 MHz, DMSO-*d*$_6$):** δ 9.03-9.00 (m, 2H), 8.20-8.17 (m, 2H), 7.96-7.90 (m, 3H), 7.69-7.64 (m, 1H), 7.55-7.51 (m, 2H), 4.66-4.10 (br m, 1H), 3.91-3.36 (br m, 5H), 3.20-2.92 (br m, 2H); **^{13}C NMR (151 MHz, DMSO-*d*$_6$) (restricted rotation at the N-CO bond leads to separate signals observed for the piperazine carbons adjacent to this bond - see 1b, pg. 7):** δ 167.7, 163.5, 146.63, 146.57, 142.3, 141.6, 137.0, 133.5, 129.7, 129.0, 128.78, 128.75, 128.70, 127.4, 55.2 (br), 54.9 (br), 45.1 (br); **LCMS (System B):** t_R = 0.87 min, m/z = 363 (MH+); **HRMS (ESI$^+$):** $C_{40}H_{37}N_8O_6$ [2M+H]$^+$ requires 725.2831 found 725.2827; **IR (cm^{-1}):** 2842 (w, C-H), 1736 (s, C=O), 1626 (s, C=O), 1613 (s, C=C); **M.pt. (DCM):** 163-166 °C.

4-(4-Cyano-2-methoxyphenoxy)piperidin-1-yl benzoate (1v)

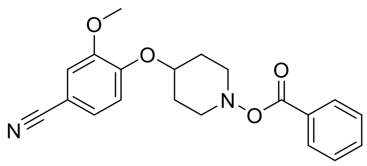

Compound synthesised following general procedure 2.1.

Amounts of each reagent used in this reaction:

Benzoic peroxyanhydride (Luperox A75, 75%) (5.56 g, 17.2 mmol, 2 eq.)

Cesium carbonate (8.42 g, 25.8 mmol, 3 eq.)

3-Methoxy-4-(piperidin-4-yloxy)benzonitrile (2 g, 8.61 mmol, 1 eq.)

DCM (initial mixture: 77 mL; Amine solution: 38 mL)

Work-up A; The product mixture was dissolved in DCM and purified by normal phase chromatography (0-80% EtOAc in cyclohexane) using an 80 g silica cartridge over 14 CV. The desired fractions were combined and evaporated *in vacuo* to give the desired product (**1v**) (2.00 g, 66%) as an orange solid. **^{1}H NMR (400 MHz, DMSO-*d*$_6$):** δ 7.94-7.93 (m, 2H), 7.69-7.64 (m, 1H), 7.55-7.52 (m, 2H), 7.43-7.38 (m, 2H), 7.24 (br d, *J* = 8.4 Hz, 1H), 4.78-4.57 (br m, 1H), 3.84 (s, 3H), 3.58-2.88 (br m, 4H) (overlap with water signal), 2.30-1.67 (br m, 4H); **^{13}C NMR (101 MHz, DMSO-*d*$_6$):** δ 163.6, 150.4, 149.8, 133.3, 129.0, 128.9, 128.7, 126.1, 119.1, 115.2, 115.0, 103.0, 70.4 (br), 56.0, 53.1 (br), 51.8 (br) (duplicated signal caused by slow conformational interconversion of the 6-membered ring), 29.0 (br), 28.0 (br) (duplicated signal caused by slow conformational interconversion of the 6-membered ring); **LCMS (System B):** t_R = 1.15 min, m/z = 353 (MH+); **HRMS (ESI$^+$):** $C_{20}H_{21}N_2O_4$ [M+H]$^+$ requires 353.1496 found 353.1501; **IR (cm^{-1}):** 2935 (w, C-H), 2859 (w, C-H), 2228 (m, C≡N), 1738 (s, C=O).

O-Benzoyl-N-(5-fluoro-2-phenoxybenzyl)-N-methylhydroxylamine (1w)

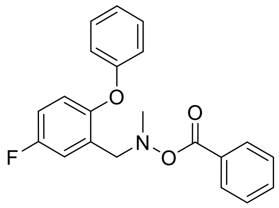

Compound synthesised following general procedure 2.1.

Amounts of each reagent used in this reaction:

Benzoic peroxyanhydride (Luperox A75, 75%) (5.59 g, 17.3 mmol, 2 eq.)

Cesium carbonate (8.45 g, 25.9 mmol, 3 eq.)

1-(5-Fluoro-2-phenoxyphenyl)-*N*-methylmethanamine (2 g, 8.65 mmol, 1 eq.)

DCM (initial mixture: 77 mL; Amine solution: 38 mL)

Work-up A; The product mixture was dissolved in DCM and purified by normal phase chromatography (0-10% TBME in cyclohexane) using an 80 g silica cartridge over 14 CV. The desired fractions were combined and evaporated *in vacuo* to give a colourless oil. LCMS showed that there was benzoyl peroxide still present. The sample was dissolved in DCM and purified by normal phase chromatography (5-20% TBME in cyclohexane) using a 40 g silica cartridge over 14 CV. The desired fractions were combined and evaporated *in vacuo* to give the desired product (**1w**) (1.41 g, 46%) as a colourless oil. **^{1}H NMR (400 MHz, DMSO-d_6):** δ 7.84-7.81 (m, 2H), 7.65-7.61 (m, 1H), 7.51-7.47 (m, 2H), 7.40-7.32 (m, 3H), 7.12-7.07 (m, 2H), 6.95-6.93 (m, 1H), 6.91-6.88 (m, 2H), 4.17 (s, 2H), 2.89 (s, 3H); **^{13}C NMR (101 MHz, DMSO-d_6):** δ 163.9, 158.1 (d, *J* = 239.6 Hz), 157.4, 150.0 (d, *J* = 3.1 Hz), 133.3, 130.3 (d, *J* = 7.6 Hz), 129.9, 128.8, 128.7, 128.6, 122.9, 121.3 (d, *J* = 9.2 Hz), 117.2, 116.7 (d, *J* = 24.4 Hz), 115.5 (d, *J* = 22.9 Hz), 57.2, 46.3; **^{19}F{^{1}H} NMR (376 MHz, DMSO-d_6):** δ -118.82 (s, 1F); **LCMS (System B):** t_R = 1.38 min, m/z = 352 (MH+); **HRMS (ESI$^+$):** $C_{21}H_{19}FNO_3$ $[M+H]^+$ requires 352.1343 found 352.1344; **IR (cm^{-1}):** 3065 (w, C-H), 2881 (w, C-H), 1738 (s, C=O), 1483.

2-(4-Ethoxyphenyl)pyrrolidin-1-yl benzoate (1x)

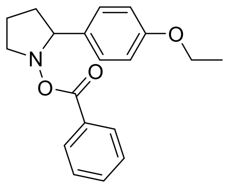

Compound synthesised following general procedure 2.1

Amounts of each reagent used in this reaction:

Benzoic peroxyanhydride (Luperox A75, 75%) (6.75 g, 20.9 mmol, 2 eq.)

Cesium carbonate (10.22 g, 31.37 mmol, 3 eq.)

2-(4-Ethoxyphenyl)pyrrolidine (2 g, 10 mmol, 1 eq.)

DCM (initial mixture: 94 mL; Amine solution: 46 mL)

Work-up B; The product mixture was preabsorbed onto celite and purified by normal phase chromatography (0-50% EtOAc in cyclohexane) using a 120 g silica cartridge over 14 CV. The desired fractions were combined and evaporated *in vacuo* to give the desired product (**1x**) (2.76 g, 89%) as a colourless oil. **^{1}H NMR (400 MHz, DMSO-*d*$_6$):** δ 7.78-7.76 (m, 2H), 7.62-7.58 (m, 1H), 7.48-7.44 (m, 2H), 7.35-7.31 (m, 2H), 6.85-6.81 (m, 2H), 4.24 (dd, *J* = 9.8, 7.9 Hz, 1H), 3.95 (q, *J* = 7.0 Hz, 2H), 3.66-3.60 (m, 1H), 3.01 (br q, *J* = 9.4 Hz, 1H), 2.30-2.21 (m, 1H), 2.05-1.89 (m, 2H), 1.79-1.69 (m, 1H), 1.27 (t, *J* = 7.0 Hz, 3H); **^{13}C NMR (101 MHz, DMSO-*d*$_6$):** δ 164.1, 157.6, 133.2, 132.1, 128.8, 128.71, 128.66, 128.1, 114.0, 69.6, 62.8, 55.2, 29.1, 19.9, 14.6; **LCMS (System B):** t_R = 1.30 min, m/z = 312 (MH+); **HRMS (ESI$^+$):** $C_{19}H_{21}NNaO_3$ $[M+Na]^+$ requires 334.1414 found 334.1411; **IR (cm^{-1}):** 2984 (w, C-H), 2943 (w, C-H), 1724 (s, C=O), 1246.

O-Benzoylhydroxylamines of the Unsuccessful Compounds

O-Benzoyl-N-benzyl-N-methylhydroxylamine (S1a)

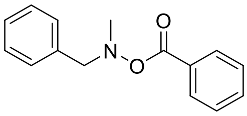

Compound synthesised following general procedure 2.1

Amounts of each reagent used in this reaction:

Benzoic peroxyanhydride (Luperox A75, 75%) (5.36 g, 16.58 mmol, 2 eq.)

Cesium carbonate (8.1 g, 24.86 mmol, 3 eq.)

N-Methyl-1-phenylmethanamine (1.07 mL, 8.29 mmol, 1 eq.)

DCM (initial mixture: 50 mL; Amine solution: 20 mL) (reaction molarity 0.118 M).

Work-up A; The product mixture was purified by normal phase chromatography (0-100% TBME in cyclohexane) using an 80 g silica cartridge over 14 CV. The desired fractions were combined and evaporated *in vacuo* to give a colourless liquid. LCMS showed that there was a large amount of benzoyl peroxide present. The sample was dissolved in DCM and purified by normal phase chromatography (0-100% EtOAc in cyclohexane) using an 80 g silica cartridge over 14 CV. The desired fractions were combined and evaporated *in vacuo* to give the desired product (**S1a**) (1.11 g, 56%) as a colourless liquid. **^{1}H NMR (600 MHz, DMSO-d_6):** δ 7.79-7.78 (m, 2H), 7.63-7.60 (m, 1H), 7.48-7.45 (m, 2H), 7.41-7.39 (m, 2H), 7.32-7.29 (m, 2H), 7.25-7.22 (m, 1H), 4.15 (s, 2H), 2.87 (s, 3H); **^{13}C NMR (151 MHz, DMSO-d_6):** δ 163.9, 136.4, 133.2, 128.89, 128.86, 128.73, 128.69, 128.1, 127.3, 63.8, 46.0; **LCMS (System B):** t_R = 1.15 min, m/z = 242 (MH+); **HRMS (ESI$^+$):** $C_{15}H_{15}NNaO_2$ $[M+Na]^+$ requires 264.0995 found 264.1013; **IR (cm^{-1}):** 3031 (w, C-H), 2853 (w, C-H), 1733 (s, C=O), 1258.

O-Benzoyl-N-benzyl-N-(but-3-en-1-yl)hydroxylamine (S1b)

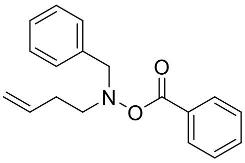

Compound synthesised following general procedure 2.1

Amounts of each reagent used in this reaction:

Benzoic peroxyanhydride (Luperox A75, 75%) (4.01 g, 12.40 mmol, 2 eq.)

Cesium carbonate (6.06 g, 18.60 mmol, 3 eq.)

N-Benzylbut-3-en-1-amine (1 g, 6.20 mmol, 1 eq.)

DCM (initial mixture: 56 mL; Amine solution: 27 mL)

Work-up A; The product mixture was purified by normal phase chromatography (0-100% DCM in cyclohexane) using a 80 g silica cartridge over 14 CV. The desired fractions were combined and evaporated *in vacuo* to give a white solid. LCMS showed that there were impurities present. The sample was dissolved in DCM and purified by normal phase chromatography (0-20% EtOAc in cyclohexane) using a 80 g silica cartridge over 14 CV. The desired fractions were combined and evaporated *in vacuo* to give the desired product (**S1b**) (669 mg, 38%) as a colourless oil **^{1}H NMR (400 MHz, DMSO-*d*$_6$):** δ 7.83-7.80 (m, 2H), 7.63-7.59 (m, 1H), 7.49-7.38 (m, 2H), 7.41-7.38 (m, 2H), 7.30-7.26 (m, 2H), 7.24-7.20 (m, 1H), 5.91-5.81 (m, 1H), 5.08-4.94 (m, 2H), 4.18 (s, 2H), 3.09 (t, *J* = 6.9 Hz, 2H), 2.33-2.27 (m, 2H); **^{13}C NMR (101 MHz, DMSO-*d*$_6$):** δ 164.1, 136.2, 135.9, 133.1, 129.1, 128.78, 128.75, 128.6, 128.0, 127.3, 115.9, 62.3, 57.3, 30.9; **LCMS (System B):** t_R = 1.32 min, m/z = 282 (MH+); **HRMS (ESI$^+$):** $C_{18}H_{20}NO_2$ [M+H]$^+$ requires 282.1489 found 282.1504; **IR (cm^{-1}):** 3064 (w, C-H), 2841 (w, C-H), 1739 (s, C=O), 1241.

***O*-Benzoyl-*N*-benzyl-*N*-(cyclopropylmethyl)hydroxylamine (S1c)**

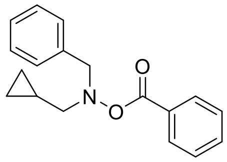

Compound synthesised following general procedure 2.1

Amounts of each reagent used in this reaction:

Benzoic peroxyanhydride (Luperox A75, 75%) (4.01 g, 12.40 mmol, 2 eq.)

Cesium carbonate (6.06 g, 18.60 mmol, 3 eq.)

N-Benzyl-1-cyclopropylmethanamine (1 g, 6.20 mmol, 1 eq.)

DCM (initial mixture: 56 mL; Amine solution: 27 mL)

Work-up A; The product mixture was purified by normal phase chromatography (0-20% EtOAc in cyclohexane) using a 80 g silica cartridge over 14 CV. The desired fractions were combined and evaporated *in vacuo* to give the desired product (**S1c**) (1.27 g, 73%) as a colourless oil. **^{1}H NMR (400 MHz, DMSO-*d*$_6$):** δ 7.83-7.80 (m, 2H), 7.63-7.58 (m, 1H), 7.49-7.45 (m, 2H), 7.42-7.39 (m, 2H), 7.30-7.26 (m, 2H), 7.23-7.19 (m, 1H), 4.19 (s, 2H), 2.92 (d, *J* = 6.4 Hz, 2H), 1.06-0.96 (m, 1H), 0.44-0.39 (m, 2H), 0.16-0.12 (m, 2H); **^{13}C NMR (101 MHz, DMSO-*d*$_6$):** δ 164.1, 136.6, 133.0, 129.1, 128.8, 128.69, 128.65, 128.0, 127.2, 62.7, 61.9, 8.14, 3.33; **LCMS (System B):** t_R = 1.29 min, m/z = 282 (MH+); **HRMS (ESI$^+$):** $C_{18}H_{20}NO_2$ $[M+H]^+$ requires 282.1489 found 282.1502; **IR (cm^{-1}):** 3005 (w, C-H), 2837 (w, C-H), 1738 (s, C=O), 1242.

4-((6-Chloropyridazin-3-yl)oxy)-2,2,6,6-tetramethylpiperidin-1-yl benzoate (S1d)

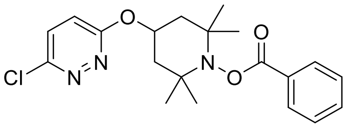

Compound synthesised following general procedure 2.1 (reaction stirred at rt for 96 h then heated to 30 °C for 20 h).

Amounts of each reagent used in this reaction:

Benzoic peroxyanhydride (Luperox A75, 75%) (**2.2**) (4.79 g, 14.8 mmol, 2 eq.)

Cesium carbonate (7.25 g, 22.2 mmol, 3 eq.)

3-Chloro-6-((2,2,6,6-tetramethylpiperidin-4-yl)oxy)pyridazine (2 g, 7.41 mmol, 1 eq.)

DCM (initial mixture: 66 mL; Amine solution: 33 mL)

Work-up A; The product mixture was dissolved in DCM and purified by normal phase chromatography (0-50% EtOAc in cyclohexane) using an 80 g silica cartridge over 14 CV. The desired fractions were combined and evaporated *in vacuo* to give the desired product (**S1d**) (1.30 g, 45%) as a white solid. **^{1}H NMR (400 MHz, DMSO-*d*$_6$):** δ 8.03-8.01 (m, 2H), 7.80 (d, *J* = 9.2 Hz, 1H), 7.72-7.68 (m, 1H), 7.59-7.55 (m, 2H), 7.33 (d, *J* = 9.2 Hz, 1H), 5.61-5.53 (m, 1H), 2.28-2.24 (m, 2H), 1.76 (br t, *J* = 11.9 Hz, 2H), 1.38 (s, 6H), 1.08 (s, 6H); **^{13}C NMR (101 MHz, DMSO-*d*$_6$):** δ 165.3, 163.9, 150.4, 133.4, 131.8, 129.01, 128.96, 128.8, 121.3, 68.6, 60.2, 43.1, 31.5, 21.2; **LCMS (System B):** t_R = 1.33 min, m/z = 390 (MH+); **HRMS (ESI$^+$):** $C_{20}H_{25}ClN_3O_3$ [M+H]$^+$ requires 390.1579 found 390.1587; **IR (cm^{-1}):** 3058 (w, C-H), 2977 (w, C-H), 1746 (s, C=O), 1411; **M.pt. (EtOAc/cyclohexane):** 200-203 °C.

N-(1-(Benzofuran-2-yl)ethyl)-O-benzoyl-N-cyclopentylhydroxylamine (S1e)

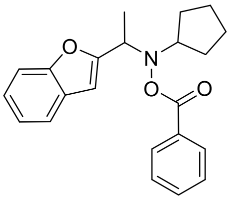

Compound synthesised following general procedure 2.1 (reaction stirred at rt for 43 h then heated to 30 °C for 24 h and then 40 °C for 4 h).

Amounts of each reagent used in this reaction:

Benzoic peroxyanhydride (Luperox A75, 75%) (5.63 g, 17.4 mmol, 2 eq.)

Cesium carbonate (8.52 g, 26.2 mmol, 3 eq.)

N-(1-(Benzofuran-2-yl)ethyl)cyclopentanamine (2 g, 8.72 mmol, 1 eq.)

DCM (initial mixture: 78 mL; Amine solution: 38 mL)

Work-up A; The product mixture was dissolved in DCM and purified by normal phase chromatography (0-15% TBME in cyclohexane) using an 80 g silica cartridge over 14 CV. The desired fractions were combined and evaporated *in vacuo* to give a yellow solid. LCMS showed that there was benzoyl peroxide still present. The sample was dissolved in DCM and purified by normal phase chromatography (0-10% TBME in cyclohexane) using a 40 g silica cartridge over 14 CV. The desired fractions were combined and evaporated *in vacuo* to give the desired product (**S1e**) (1.38 g, 45%) as a yellow oil. **^{1}H NMR (600 MHz, DMSO-d_6):** δ 7.95 (br d, J = 7.7 Hz, 2H), 7.67-7.64 (m, 1H), 7.61-7.58 (m, 1H), 7.56-7.52 (m, 3H), 7.28 (t, J = 7.4 Hz, 1H), 7.21 (d, J = 7.4 Hz, 1H), 6.88 (s, 1H), 4.54 (q, J = 6.9 Hz, 1H), 3.48-3.36 (br m, 1H), 1.96-1.81 (br m, 1H), 1.69-1.56 (m, 4H), 1.55-1.40 (br m, 6H); **^{13}C NMR (151 MHz, DMSO-d_6):** δ 164.9, 156.3 (br), 154.1, 133.3, 129.0, 128.84, 128.76, 127.7, 124.1, 122.7, 121.0, 111.0, 105.1 (br), 65.1, 57.1, 30.0, 29.4 (diastereomeric signal), 24.1, 23.8; **LCMS (System A):** t_R = 1.47 min, m/z = 350 (MH+); **HRMS (ESI$^+$):** $C_{22}H_{23}NNaO_3$ $[M+Na]^+$ requires 372.1570 found 372.1583; **IR (cm^{-1}):** 2957 (m, C-H), 2870 (w, C-H), 1742 (s, C=O), 1451.

Ethyl 5-(benzoyloxy)-3-(4-fluorobenzamido)-6,6-dimethyl-5,6-dihydropyrrolo[3,4-*c*]pyrazole-1(4*H*)-carboxylate (S1f)

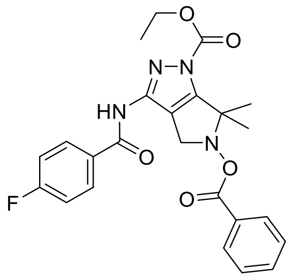

Compound synthesised following general procedure 2.1 (reaction stirred at rt for 43 h then heated to 30 °C for 24 h and then 40 °C for 4 h).

Amounts of each reagent used in this reaction:

Benzoic peroxyanhydride (Luperox A75, 75%) (3.73 g, 11.5 mmol, 2 eq.)

Cesium carbonate (5.64 g, 17.3 mmol, 3 eq.)

Ethyl 3-(4-fluorobenzamido)-6,6-dimethyl-5,6-dihydropyrrolo[3,4-*c*]pyrazole-1(4*H*)-carboxylate (2 g, 5.77 mmol, 1 eq.)

DCM (initial mixture: 52 mL; Amine solution: 25 mL)

Work-up A; The product mixture was dissolved in DCM and purified by normal phase chromatography (0-100% EtOAc in cyclohexane) using an 80 g silica cartridge over 14 CV. The desired fractions were combined and evaporated *in vacuo* to give the desired product (**S1f**) (1.03 g, 38%) as a white solid. **^{1}H NMR (400 MHz, DMSO-*d*$_6$):** δ 11.5 (s, 1H), 8.14-8.08 (m, 2H), 7.97-7.95 (m, 2H), 7.69-7.65 (m, 1H), 7.56-7.52 (m, 2H), 7.35-7.29 (m, 2H), 4.49 (s, 2H), 4.45 (q, *J* = 7.1 Hz, 2H), 1.59 (s, 6H), 1.36 (t, *J* = 7.1 Hz, 3H); **^{13}C NMR (101 MHz, DMSO-*d*$_6$):** δ 164.4 (d, *J* = 250.2 Hz), 164.2, 164.1, 153.6, 148.3, 144.5, 133.4, 130.8 (d, *J* = 9.2 Hz), 129.3 (d, *J* = 3.1 Hz), 129.0, 128.8, 128.6, 115.6 (d, *J* = 82.4 Hz), 115.4, 68.5, 64.3, 55.3, 22.3, 14.0; **^{19}F{^{1}H} NMR (376 MHz, DMSO-*d*$_6$):** δ -107.68 (s, 1F); **LCMS (System B):** t_R = 1.23 min, m/z = 467 (MH+); **HRMS (ESI$^+$):** $C_{24}H_{24}FN_4O_5$ $[M+H]^+$ requires 467.1725 found 467.1727; **IR (cm^{-1}):** 3252 (w, N-H), 1748 (s, C=O), 1725 (s, C=O), 1671 (s, C=O); **M.pt. (EtOAc/cyclohexane):** 180-183 °C.

***O*-Benzoyl-*N*-phenethylhydroxylamine (S2a)**

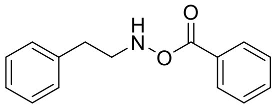

Compound synthesised following general procedure 2.1 (total reaction time was 14 h).

Amounts of each reagent used in this reaction:

Benzoic peroxyanhydride (Luperox A75, 75%) (323 mg, 1 mmol, 2 eq.)

Cesium carbonate (489 mg, 1.5 mmol, 3 eq.)

2-Phenylethan-1-amine (63 µL, 0.5 mmol, 1 eq.)

DCM (initial mixture: 5 mL; Amine solution: 2 mL)

Work-up A; The product mixture was dissolved in DCM and purified by normal phase chromatography (0-50% TBME in cyclohexane) using a 12 g silica cartridge over 14 CV. The desired fractions were combined and evaporated *in vacuo* to give the desired product (**S2a**) (98 mg, 81%) as a colourless oil. **^{1}H NMR (600 MHz, DMSO-*d*$_6$):** δ 8.30 (t, *J* = 5.8 Hz, 1H), 7.91-7.89 (m, 2H), 7.68-7.65 (m, 1H), 7.54-7.52 (m, 2H), 7.30-7.26 (m, 4H), 7.21-7.18 (m, 1H), 3.33-3.30 (m, 2H), 2.87 (t, *J* = 7.3 Hz, 2H); **^{13}C NMR (151 MHz, DMSO-*d*$_6$):** δ 165.3, 139.5, 133.3, 128.79, 128.77, 128.6, 128.5, 128.3, 126.0, 52.8, 33.4; **LCMS (System B):** t_R = 1.20 min, m/z = 242 (MH+); **HRMS (ESI$^+$):** $C_{15}H_{16}NO_2$ $[M+H]^+$ requires 242.1176 found 242.1185; **IR (cm^{-1}):** 3234 (w, N-H), 3028 (w, C-H), 1716 (s, C=O), 1266.

***O*-Benzoyl-*N*-(4-(trifluoromethoxy)phenethyl)hydroxylamine (S2b)**

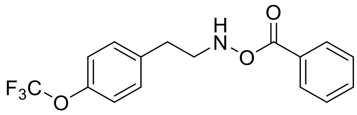

Compound synthesised following general procedure 2.1

Amounts of each reagent used in this reaction:

Benzoic peroxyanhydride (Luperox A75, 75%) (2.67 g, 8.28 mmol, 2 eq.)

Cesium carbonate (4.05 g, 12.42 mmol, 3 eq.)

2-(4-(Trifluoromethoxy)phenyl)ethan-1-amine, hydrochloride (1 g, 4.14 mmol, 1 eq.)

DCM (initial mixture: 35 mL; Amine solution: 20 mL)

Work-up A; The product mixture was preabsorbed onto celite and purified by normal phase chromatography (0-20% EtOAc in cyclohexane) using a 80 g silica cartridge over 14 CV. The desired fractions were combined and evaporated *in vacuo* to give the desired product (**S2b**) (558 mg, 42%) as a colourless oil. **^{1}H NMR (600 MHz, DMSO-*d*$_6$):** δ 8.34 (t, *J* = 5.5 Hz, 1H), 7.90-7.88 (m, 2H), 7.67-7.65 (m, 1H), 7.52 (t, *J* = 7.7 Hz, 2H), 7.41-7.39 (m, 2H), 7.26 (d, *J* = 8.6 Hz, 2H), 3.34-3.31 (m, 2H), 2.91 (t, *J* = 6.6 Hz, 2H); **^{13}C NMR (151 MHz, DMSO-*d*$_6$):** δ 165.3, 146.7 (q, *J* = 1.7 Hz), 139.2, 133.3, 130.5, 128.753, 128.745, 128.5, 120.8, 120.1 (q, *J* = 255.4 Hz), 52.5, 32.7; **^{19}F NMR (376 MHz, DMSO-*d*$_6$):** δ -58.87 (s, 3F); **LCMS (System B):** t_R = 1.31 min, m/z = 326 (MH+); **HRMS (ESI$^+$):** $C_{16}H_{15}F_3NO_3$ $[M+H]^+$ requires 326.0999 found 326.1014; **IR (cm^{-1}):** 3237 (br, w, N-H), 2937 (w, C-H), 1718 (s, C=O), 1253.

O-Benzoyl-N-(1-(pyrimidin-2-yl)ethyl)hydroxylamine (S2c)

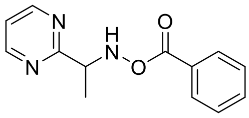

Compound synthesised following general procedure 2.1

Amounts of each reagent used in this reaction:

Benzoic peroxyanhydride (Luperox A75, 75%) (3.29 g, 10.20 mmol, 2 eq.)

Cesium carbonate (4.98 g, 15.30 mmol, 3 eq.)

1-(Pyrimidin-2-yl)ethan-1-amine, di-hydrochloride (1 g, 5.10 mmol, 1 eq.)

DCM (initial mixture: 46 mL; Amine solution: 22 mL)

Work-up A; The product mixture was preabsorbed onto celite and purified by normal phase chromatography (50-100% EtOAc in cyclohexane) using a 80 g silica cartridge over 14 CV. The desired fractions were combined and evaporated *in vacuo* to give the desired product (**S2c**) (634 mg, 51%) as a white solid. **^{1}H NMR (400 MHz, DMSO-d_6):** δ 8.80 (d, *J* = 4.9 Hz, 2H), 8.56 (d, *J* = 8.9 Hz, 1H), 7.76-7.73 (m, 2H), 7.63-7.59 (m, 1H), 7.48-7.44 (m, 2H), 7.40 (t, *J* = 4.9 Hz, 1H), 4.64-4.67 (m, 1H), 1.46 (d, *J* = 6.9 Hz, 3H); **^{13}C NMR (101 MHz, DMSO-d_6):** δ 169.3, 164.8, 157.4, 133.3, 128.8, 128.5, 128.1, 120.1, 61.3, 17.1; **LCMS (System B):** t_R = 0.82 min, m/z = 244 (MH+); **HRMS (ESI$^+$):** $C_{13}H_{14}N_3O_2$ $[M+H]^+$ requires 244.1081 found 244.1094; **IR (cm^{-1}):** 3214 (w, N-H), 2978 (w, C-H), 1714 (s, C=O), 1563.

O-Benzoyl-N-(2-(naphthalen-1-yl)ethyl)hydroxylamine (S2d)

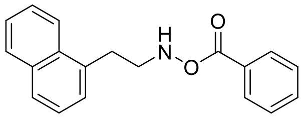

Compound synthesised following general procedure 2.1

Amounts of each reagent used in this reaction:

Benzoic peroxyanhydride (Luperox A75, 75%) (3.77 g, 11.7 mmol, 2 eq.)

Cesium carbonate (5.71 g, 17.5 mmol, 3 eq.)

2-(Naphthalen-1-yl)ethan-1-amine (1 g, 5.84 mmol, 1 eq.)

DCM (initial mixture: 52 mL; Amine solution: 26 mL)

Work-up A; The product mixture was dissolved in DCM and purified by normal phase chromatography (0-30% EtOAc in cyclohexane) using a 80 g silica cartridge over 14 CV. The desired fractions were combined and evaporated *in vacuo* to give a colourless oil. LCMS showed that there was benzoyl peroxide present. The product mixture was dissolved in DCM and purified by normal phase chromatography (0-25% EtOAc in cyclohexane) using a 40 g silica cartridge over 14 CV. The desired fractions were combined and evaporated *in vacuo* to give the desired product (**S2d**) (534 mg, 31%) as colourless oil. **^{1}H NMR (600 MHz, DMSO-d_6):** δ 8.44 (t, *J* = 5.7 Hz, 1H), 8.14 (d, *J* = 8.4 Hz, 1H), 7.93-7.90 (m, 3H), 7.81-7.78 (m, 1H), 7.69-7.66 (m, 1H), 7.55-7.50 (m, 4H), 7.45-7.42 (m, 2H), 3.43-3.40 (m, 2H), 3.38-3.34 (m, 2H); **^{13}C NMR (151 MHz, DMSO-d_6):** δ 165.3, 135.5, 133.44, 133.35, 131.5, 128.82, 128.76, 128.6, 128.5, 126.72, 126.68, 126.0, 125.59, 125.56, 123.6, 52.4, 30.5; **LCMS (System B):** t_R = 1.31 min, m/z = 292; **HRMS (ESI$^+$):** $C_{19}H_{18}NO_2$ $[M+H]^+$ requires 292.1332 found 292.1331; **IR (cm^{-1}): 3233 (**br w, N-H), 3060 (w, C-H), 1715 (s, C=O), 1266.

***N*-(2-(1*H*-Pyrazol-1-yl)ethyl)-*O*-benzoylhydroxylamine (S2e)**

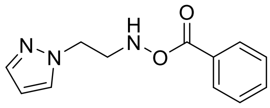

Compound synthesised following general procedure 2.1

Amounts of each reagent used in this reaction:

Benzoic peroxyanhydride (Luperox A75, 75%) (5.81 g, 17.99 mmol, 2 eq.)

Cesium carbonate (8.79 g, 27 mmol, 3 eq.)

2-(1*H*-Pyrazol-1-yl)ethan-1-amine (1 g, 9 mmol, 1 eq.)

DCM (initial mixture: 80 mL; Amine solution: 40 mL)

Work-up A; The product mixture was preabsorbed onto celite and purified by normal phase chromatography (50-100% EtOAc in cyclohexane) using a 80 g silica cartridge over 14 CV. The desired fractions were combined and evaporated *in vacuo* to give the desired product (**S2e**) (886 mg, 43%) as a colourless oil. **^{1}H NMR (400 MHz, DMSO-*d*$_6$):** δ 8.41 (t, *J* = 6.0 Hz, 1H), 7.93-7.90 (m, 2H), 7.77 (d, *J* = 2.2 Hz, 1H), 7.69-7.65 (m, 1H), 7.55-7.51 (m, 2H), 7.45 (d, *J* = 2.2 Hz, 1H), 6.23 (t, *J* = 2.2 Hz, 1H), 4.36 (t, *J* = 6.0 Hz, 2H), 3.47 (q, *J* = 6.0 Hz, 2H); **^{13}C NMR (101 MHz, DMSO-*d*$_6$):** δ 165.2, 138.7, 133.4, 130.3, 128.8, 128.7, 128.3, 104.9, 51.4, 49.1; **LCMS (System B):** t_R = 0.83 min, m/z = 232 (MH+); **HRMS (ESI$^+$):** $C_{12}H_{14}N_3O_2$ $[M+H]^+$ requires 232.1081 found 232.1091; **IR (cm^{-1}):** 3229 (m, N-H), 2946 (w, C-H), 1717 (s, C=O), 1265.

Preparation of Amides and Sulfonamides:

N-(2-(1H-pyrazol-1-yl)ethyl)-N-(benzoyloxy)benzamide (S3a)

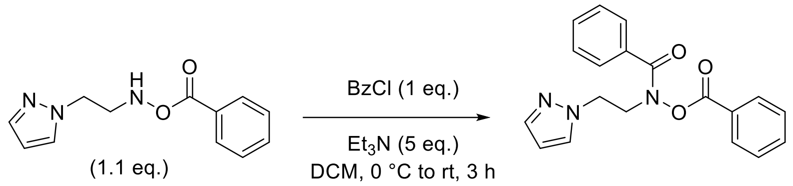

N-(2-(1*H*-Pyrazol-1-yl)ethyl)-*O*-benzoylhydroxylamine (**S2e**) (54 mg, 0.234 mmol, 1.1 eq.) and triethylamine (149 µL, 1.067 mmol, 5 eq.) were added to DCM (0.9 mL) and cooled to 0 °C. A solution of benzoyl chloride (25 µL, 0.213 mmol, 1 eq.) in DCM (0.9 mL) was added dropwise to the reaction mixture. The reaction mixture was then stirred at room temperature under a nitrogen atmosphere for 3 h. The reaction mixture was washed with water (2 x 10 mL) and the layers separated each time. The aqueous layer was extracted with DCM (2 x 10 mL) and the combined organics were washed with brine (20 mL), dried through a hydrophobic frit and evaporated *in vacuo* to give the crude product as a brown oil. The crude product was dissolved in DMSO (1 mL) and purified by MDAP (Method A) and the desired fractions were combined and evaporated *in vacuo* to afford **S3a** (64 mg, 89%) as a yellow oil. **^{1}H NMR (600 MHz, DMSO-d_6):** δ 7.90-7.89 (m, 2H), 7.84 (d, *J* = 2.1 Hz, 1H), 7.43 (br t, *J* = 7.6 Hz, 1H), 7.55 (br t, *J* = 7.6 Hz, 2H), 7.46-7.43 (m, 2H), 7.43-7.30 (m, 4H), 6.27 (t, *J* = 2.1 Hz, 1H), 4.48 (t, *J* = 5.6 Hz, 2H), 4.28-4.13 (br m, 2H); **^{13}C NMR (151 MHz, DMSO-d_6):** δ 169.1, 163.7, 139.1, 134.6, 132.8, 131.0, 130.7, 129.6, 129.0, 128.2, 127.4, 126.3, 105.2, 50.0 (br), 48.4; **LCMS (System B):** t_R = 1.02 min, m/z = 336; **HRMS (ESI$^+$):** $C_{19}H_{18}N_3O_3$ [M+H]$^+$ requires 336.1343 found 336.1349; **IR (cm^{-1}):** 3063 (w, C-H), 1759 (s, C=O), 1666 (s, C=O), 1237.

***N*-(2-(1*H*-Pyrazol-1-yl)ethyl)-*N*-(benzoyloxy)-4-methylbenzenesulfonamide (S3b)**

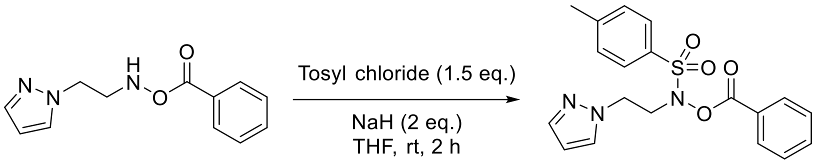

N-(2-(1*H*-Pyrazol-1-yl)ethyl)-*O*-benzoylhydroxylamine (**S2e**) (150 mg, 0.649 mmol, 1 eq.) was dissolved in THF (6 mL) and placed under a nitrogen atmosphere. To this solution was added NaH (60% dispersion in mineral oil; 52 mg, 1.3 mmol, 2 eq.) and the solution was stirred for 5 min. To this solution was added tosyl chloride (185 mg, 0.973 mmol, 1.5 eq.) and the reaction mixture was stirred at room temperature for 2 h. The reaction mixture was evaporated *in vacuo* to give the crude product as a white solid. The sample was preabsorbed onto celite and purified by normal phase chromatography (0-100% EtOAc in cyclohexane) using a 13 g silica cartridge over 14 CV. The desired fractions were combined and evaporated *in vacuo* to give a colourless oil. LCMS showed that there were large amounts of unknown impurities present. The sample was dissolved in DMSO (1 mL) and purified by MDAP (Method A) and the desired fractions were combined and evaporated *in vacuo* to afford **S3b** (77 mg, 31%) as a colourless oil. **^{1}H NMR (400 MHz, DMSO-*d*$_6$):** δ 7.73-7.71 (m, 2H), 7.63 (d, *J* = 2.0 Hz, 1H), 7.48-7.43 (m, 1H), 7.38-7.36 (m, 2H), 7.34-7.28 (m, 3H), 7.08-7.05 (m, 2H), 6.20 (t, *J* = 2.0 Hz, 1H), 4.38 (br t, *J* = 5.4 Hz, 2H), 4.20-4.00 (br m, 2H), 2.38 (s, 3H); **^{13}C NMR (101 MHz, DMSO-*d*$_6$):** δ 169.8, 146.5, 139.1, 131.6, 131.4, 130.7, 130.0, 129.5, 128.9, 127.93, 127.89, 105.3, 52.8, 47.0, 21.1; **LCMS (System B):** t_R = 1.07 min, m/z = 386; **HRMS (ESI$^+$):** $C_{19}H_{20}N_3O_4S$ [M+H]$^+$ requires 386.1169 found 386.1169; **IR (cm^{-1}):** 2956 (w, C-H), 1699 (s, C=O), 1373 (s, S=O), 1176.

***N*-(Benzoyloxy)-*N*-phenethylbenzamide (S3c)**

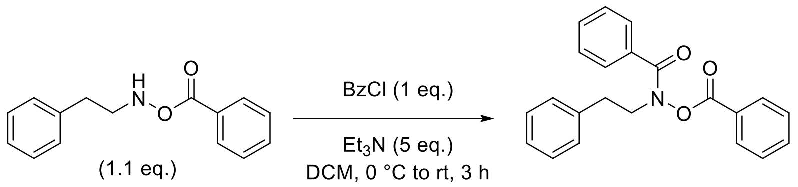

O-Benzoyl-*N*-phenethylhydroxylamine (**S2a**) (190 mg, 787 µmol, 1.1 eq.) and triethylamine (362 mg, 498 µL, 3.58 mmol, 5 eq.) were added in DCM (3 mL) and cooled to 0 °C under a nitrogen atmosphere. A solution of benzoyl chloride (101 mg, 83.0 µL, 715 µmol, 1 eq.) in DCM (3 mL) was added dropwise to the reaction mixture. The reaction mixture was then stirred at room temperature for 3 h. The reaction mixture was washed with water (2 x 20 mL) and the layers separated each time. The aqueous layer was extracted with DCM (2 x 20 mL) and the combined organics were washed with brine (40 mL), dried through a hydrophobic frit and evaporated *in vacuo* to give the crude product as an orange oil. The crude product was dissolved in DMSO (2 x 1 mL) and purified by MDAP (Method A) and the desired fractions were combined and evaporated *in vacuo* to afford **S3c** (213 mg, 86%) as a yellow oil. **^{1}H NMR (400 MHz, DMSO-*d*$_6$):** δ 7.88 (br d, *J* = 7.9 Hz, 2H), 7.74-7.70 (m, 1H), 7.54 (br t, *J* = 7.9 Hz, 2H), 7.46-7.40 (m, 1H), 7.39-7.34 (m, 4H), 7.32-7.26 (m, 4H), 7.25-7.20 (m, 1H), 4.05 (br t, *J* = 6.8 Hz, 2H), 3.01 (t, *J* = 6.8 Hz, 2H); **^{13}C NMR (101 MHz, DMSO-*d*$_6$):** δ 169.0, 163.7, 138.3, 134.5, 133.2, 130.8, 129.4, 129.0, 128.9, 128.3, 128.2, 127.2, 126.4, 126.3, 51.0 (br), 32.9; **LCMS (System B):** t_R = 1.30 min, m/z = 346; **HRMS (ESI$^+$):** $C_{22}H_{20}NO_3$ $[M+H]^+$ requires 346.1438 found 346.1441; **IR (cm^{-1}):** 3062 (w, C-H), 2932 (w, C-H), 1759 (s, C=O), 1666 (s, C=O).

3. Optimization Studies

Preliminary Reaction Optimization:

General procedure 3.1:

4-Benzoylpiperazin-1-yl benzoate (**1a**) (100 mg, 0.322 mmol, 1 eq.), copper (I) chloride (4 mg, 0.040 mmol, 0.13 eq.), and silver (I) fluoride (or cesium fluoride, 59 mg, 0.388 mmol, 1.2 eq., for Entry A) were added to a microwave vial and stirred in THF (3 mL) at room temperature under a nitrogen atmosphere. To the reaction mixture was added trimethyl(trifluoromethyl)silane dropwise and the reaction mixture was stirred at 40 °C for 48 h or 50 °C for 24 h (see Table S1 Entry A and B for their respective temperature and time). The reaction mixture was diluted with EtOAc (10 mL) and washed with 1 M Na_2CO_3 aqueous solution (2 x 20 mL). The combined organics were then dried through a hydrophobic frit, absorbed onto celite and purified by normal phase chromatography (20-100% EtOAc in cyclohexane) using a 12 g silica cartridge over 14 CV. The desired fractions were combined and evaporated *in vacuo* to give the desired product (**2a**) as a colourless oil.

Table S1: Testing the effects of temperature, time, and equivalents of $TMSCF_3$ and AgF on reaction yield, following general procedure 3.1. [a] CsF used instead of AgF. [b] rt for 119 h then heated to 40 °C for 24 h. [c] Reaction run in duplicate.

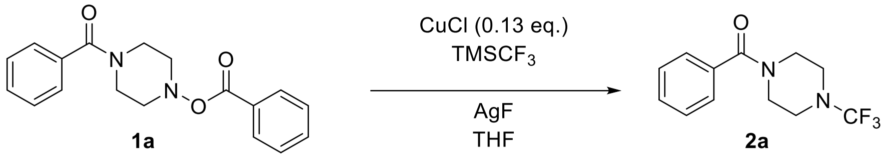

Entry	Quantity of $TMSCF_3$ used	Quantity of AgF used	Temperature / °C	Time / h	Percentage isolated yield of **2a** / %
A	57 µL, 0.387 mmol, 1.2 eq.	59 mg, 0.388 mmol, 1.2 eq. [a]	rt	121	20 mg, 0.077 mmol, 24%
B	57 µL, 0.387 mmol, 1.2 eq.	49 mg, 0.386 mmol, 1.2 eq.	rt[b]	143	28 mg, 0.108 mmol, 34%
C	57 µL, 0.387 mmol, 1.2 eq.	49 mg, 0.386 mmol, 1.2 eq.	40	48	26 mg, 0.101 mmol, 31%, 29 mg, 0.112 mmol, 35%[c]
D	57 µL, 0.387 mmol, 1.2 eq.	49 mg, 0.386 mmol, 1.2 eq.	50	24	23 mg, 0.089 mmol, 28%
E	95 µL, 0.644 mmol, 2 eq.	82 mg, 0.644 mmol, 2 eq.	40	48	42 mg, 0.163 mmol, 51%
F	191 µL, 1.289 mmol, 4 eq.	164 mg, 1.289 mmol, 4 eq.	40	48	42 mg, 0.163 mmol, 51%
G	286 µL, 1.933 mmol, 6 eq.	245 mg, 1.933 mmol, 6 eq.	40	48	42 mg, 0.163 mmol, 51%

As shown in Table S1, a 51% yield was recorded when the number of equivalents of $TMSCF_3$ and AgF were increased to 2, 4 or 6 at 40 °C for 48 h. The increase in temperature allowed for a reduction in reaction time from 143 h to 48 h, with the increase in yield arising from the equivalents of $TMSCF_3$ and AgF. As the yield was not improved upon increasing the equivalents from 2 to 4 to 6, the conditions highlighted in entry E (Table S1) were used as the seed conditions for the high throughput screen (HTS).

High throughput screen:

General procedure 3.2:

Within a nitrogen filled glovebox, CuCl (21.4 mg, 0.216 mmol) was dissolved in methanol (7179 µL) and dispensed across the 96 well plate as a suspension (50 µL per well, 1.5 µmol per well). Each ligand (0.054 mmol) was dissolved in methanol (1782-1785 µL per ligand) and dispensed as per the plate design (50 µL per well, 1.5 µmol per well). The solvent was then evaporated using the Genevac* and then each fluoride source (20 µmol, 2 eq.) was added by solid addition as per the plate design. Stock solutions of 4-benzoylpiperazin-1-yl benzoate in each of the three solvents were prepared by dissolving 4-benzoylpiperazin-1-yl benzoate (**1a**) (149 mg, 0.480 mmol) in each solvent (2251 µL per solvent) and these were dispensed as per plate design (50 µL per well, 10 µmol per well). Stock solutions of $TMSCF_3$ in each of the three solvents were prepared by dissolving $TMSCF_3$ (142 µL, 0.961 mmol) in each solvent (2258 µL per solvent) and these were dispensed as per plate design (50 µL per well, 20 µmol per well). The plate was then sealed, removed from the glovebox and stirred at 40 °C for 48 h. The reaction mixtures were quenched with standard (*N,N*-dibenzylaniline (1 µmol per well)) and DMSO (20 µmol per well) and the volume of each well was topped up to 500 µL using acetonitrile. Aliquots of each reaction mixture (50 µL per well) were dispensed into corresponding wells on an LCMS analysis plate and 150 µL of acetonitrile was added to each well. These solutions were analysed by LCMS (High pH, 2 mins).

* Vials F6 and F7 were cracked during evaporation of methanol in the Genevac. For these vials, the required reaction solution of $TMSCF_3$, *N*-oxide ester, CuCl and the ligand was made up in DMF, and this was transferred to a new vial containing the fluoride source.

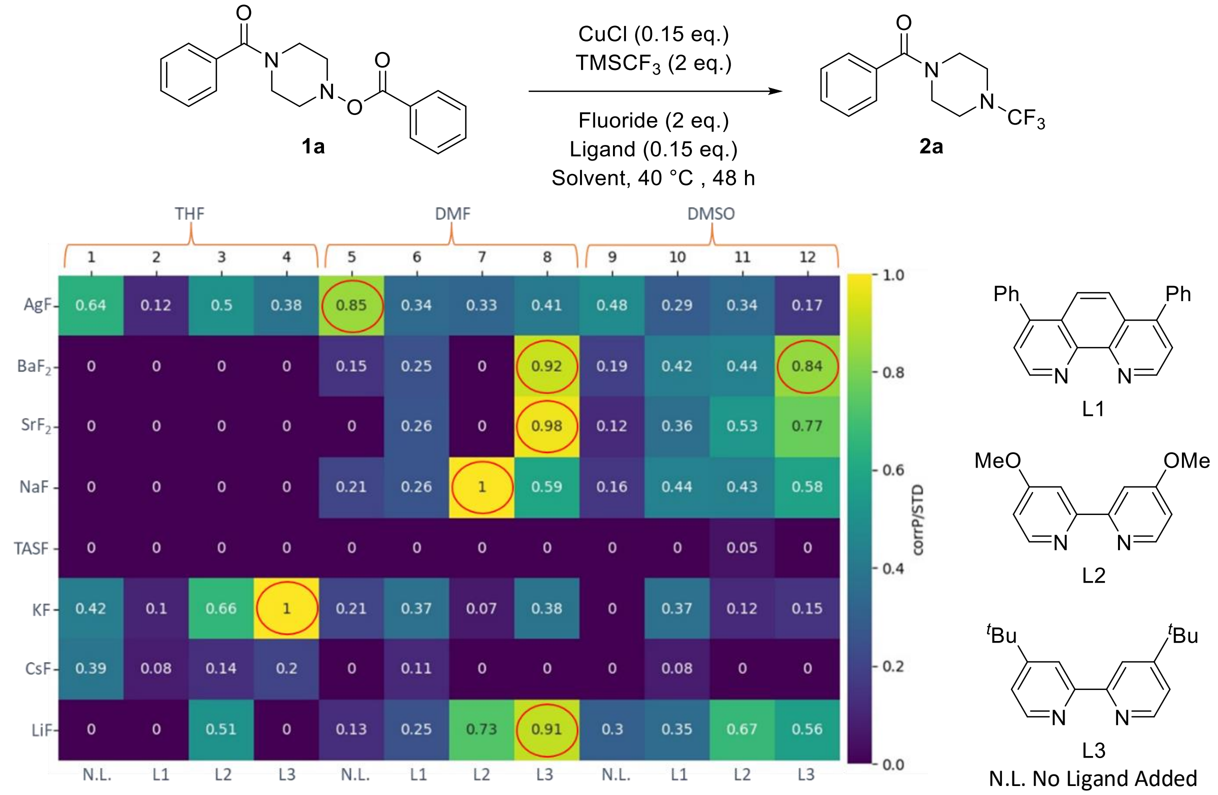

Figure S1: HTS for the optimisation of the desired reaction in which the effects of fluoride source, ligands, and solvent were tested. CorrP/STD = ratio of the product to the internal standard (IS) (*N*,*N*-dibenzylaniline), relative to the maximum observed product:IS ratio over the whole plate.

LCMS analysis was performed on each well of the plate which revealed a total of seven hits (hit identified as having a corrP/STD >0.8) and these were subsequently validated on a 100 mg scale and yields were isolated (Table S2).

General procedure 3.3:

4-Benzoylpiperazin-1-yl benzoate (**1a**) (100 mg, 0.322 mmol, 1 eq.), copper (I) chloride (4.8 mg, 0.048 mmol, 0.15 eq.), ligand (0.15 eq.) (if applicable) and fluoride source (2 eq.) were added to a microwave vial and stirred in solvent (3 mL) at room temperature under a nitrogen atmosphere. To the reaction mixture was added trimethyl(trifluoromethyl)silane (95 µL, 0.644 mmol, 2 eq.) dropwise and the reaction mixture was stirred at 40 °C for 48 h. The reaction mixture was diluted with EtOAc (10 mL) and washed with 1 M Na_2CO_3 aqueous solution (2 x 20 mL). The combined organics were then dried through a hydrophobic frit, absorbed onto celite and purified by normal phase chromatography (20-100% EtOAc in cyclohexane) using a 12 g silica cartridge over 14 CV. The desired fractions were combined and evaporated *in vacuo* to give the desired product (**2a**) as a colourless oil (or a white solid).

Table S2: Validation of hits identified from the HTS following general procedure 3.3.

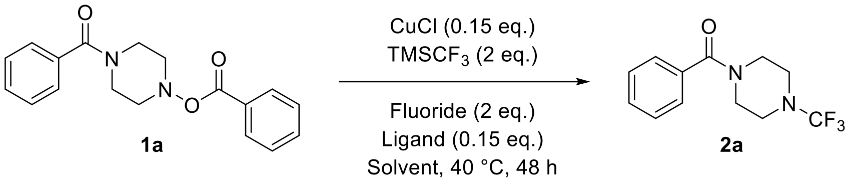

Entry	Fluoride source	Ligand	Solvent	Percentage isolated yield of **2a** / %
A	AgF - 82 mg, 0.644 mmol, 2 eq.	No ligand	DMF	52 mg, 0.2013 mmol, 63%
B	BaF_2 - 113 mg, 0.644 mmol, 2 eq.	^{t}Bu-Bipy - 13 mg, 0.048 mmol, 0.15 eq.	DMF	61 mg, 0.236 mmol, 73%
C	BaF_2 - 113 mg, 0.644 mmol, 2 eq.	^{t}Bu-Bipy - 13 mg, 0.048 mmol, 0.15 eq.	DMSO	56 mg, 0.217 mmol, 67%
D	SrF_2 - 81 mg, 0.644 mmol, 2 eq.	^{t}Bu-Bipy - 13 mg, 0.048 mmol, 0.15 eq.	DMF	62 mg, 0.240 mmol, 75%
E	NaF - 27 mg, 0.643 mmol, 2 eq.	MeO-Bipy - 10.5 mg, 0.049 mmol, 0.15 eq.	DMF	64 mg, 0.248 mmol, 77%
F	KF - 37 mg, 0.637 mmol, 2 eq.	^{t}Bu-Bipy - 13 mg, 0.048 mmol, 0.15 eq.	THF	50 mg, 0.194 mmol, 60%
G	LiF - 17 mg, 0.655 mmol, 2 eq.	^{t}Bu-Bipy - 13 mg, 0.048 mmol, 0.15 eq.	DMF	59 mg, 0.228 mmol, 71%

As elucidated from Table S2, each of the validated hits afforded yields ≥60% with entries D and E performing the best with yields ≥75%. Consequently, these two sets of reaction conditions were selected for use in the next stage of reaction optimisation.

Testing Alternate Copper Sources:

General procedure 3.4:

4-Benzoylpiperazin-1-yl benzoate (**1a**) (100 mg, 0.322 mmol, 1 eq.), copper source (0.15 eq.) ligand (0.15 eq.) and fluoride source (2 eq.) were added to a microwave vial and stirred in DMF (3 mL) at room temperature under a nitrogen atmosphere. To the reaction mixture was added trimethyl(trifluoromethyl)silane (95 µL, 0.644 mmol, 2 eq.) dropwise and the reaction mixture was stirred at 40 °C for 48 h. The reaction mixture was diluted with EtOAc (10 mL) and washed with 1 M Na_2CO_3 aqueous solution (2 x 20 mL) (For entry B3, yield 3, work-up was skipped and the reaction mixture was directly absorbed onto celite). The combined organics were then dried through a hydrophobic frit, absorbed onto celite and purified by normal phase chromatography (20-100% EtOAc in cyclohexane) (0-100% EtOAc in cyclohexane for entry B3, yield 3) using a 12 g silica cartridge over 14 CV. The desired fractions were combined and evaporated *in vacuo* to give the desired product (**2a**) as a colourless oil (or a white solid).

Figure S2: Focused screen testing the effects of different copper sources.

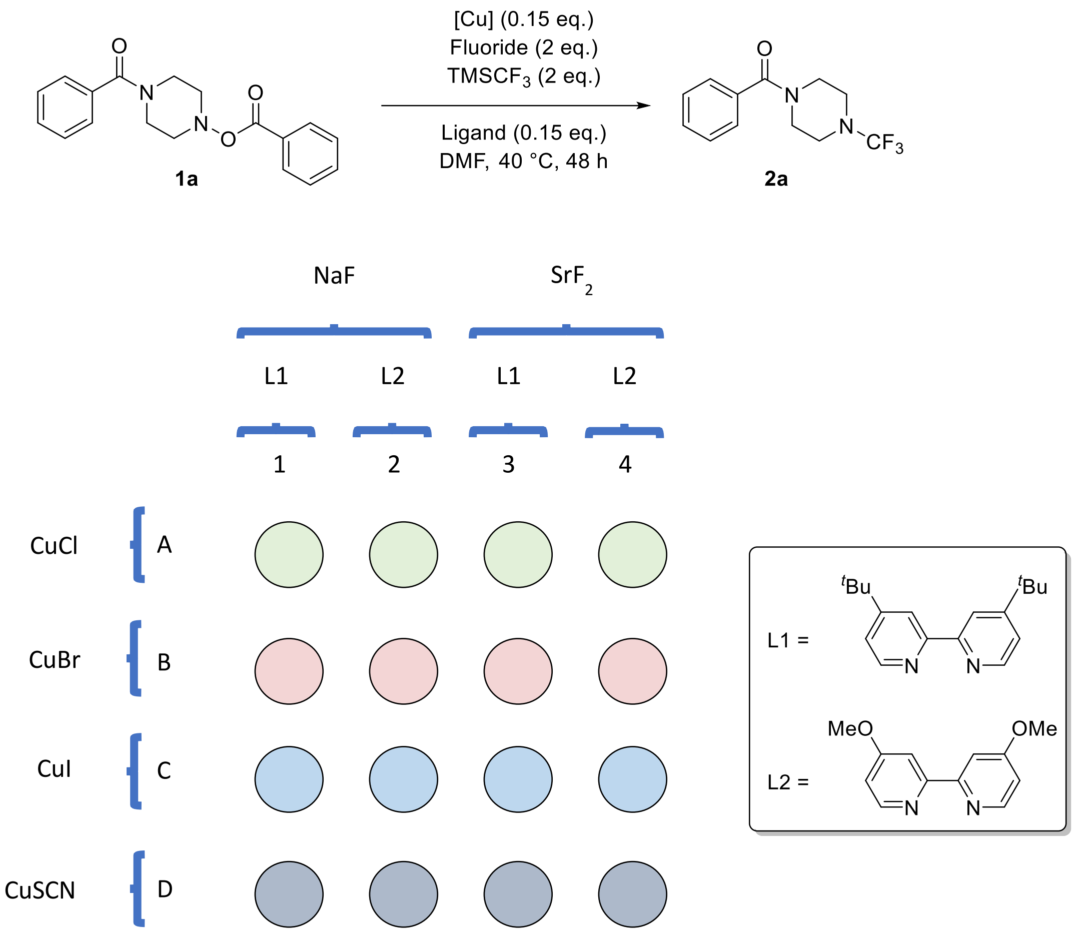

Table S3: Optimisation results upon changing the copper source, following general procedure 3.4. The entry codes correspond to the letter-number codes shown in Figure S2. [a] Reaction run in triplicate.

Entry	Copper source	Fluoride source	Ligand	Percentage isolated yield of **2a** / %
A1	CuCl - 4.8 mg, 0.048 mmol, 0.15 eq.	NaF - 27 mg, 0.643 mmol, 2 eq.	L1 - 13 mg, 0.048 mmol, 0.15 eq.	58 mg, 0.225 mmol, 70%
A2	CuCl - 4.8 mg, 0.048 mmol, 0.15 eq.	NaF - 27 mg, 0.643 mmol, 2 eq.	L2 - 10.5 mg, 0.049 mmol, 0.15 eq.	53 mg, 0.205 mmol, 64%
A3	CuCl - 4.8 mg, 0.048 mmol, 0.15 eq.	SrF_2 - 81 mg, 0.644 mmol, 2 eq.	L1 - 13 mg, 0.048 mmol, 0.15 eq.	62 mg, 0.240 mmol, 75%
A4	CuCl - 4.8 mg, 0.048 mmol, 0.15 eq.	SrF_2 - 81 mg, 0.644 mmol, 2 eq.	L2 - 10.5 mg, 0.049 mmol, 0.15 eq.	55 mg, 0.213 mmol, 66%
B1	CuBr - 7 mg, 0.049 mmol, 0.15 eq.	NaF - 27 mg, 0.643 mmol, 2 eq.	L1 - 13 mg, 0.048 mmol, 0.15 eq.	67 mg, 0.259 mmol, 81%
B2	CuBr - 7 mg, 0.049 mmol, 0.15 eq.	NaF - 27 mg, 0.643 mmol, 2 eq.	L2 - 10.5 mg, 0.049 mmol, 0.15 eq.	62 mg, 0.240 mmol, 75%
B3[a]	CuBr - 7 mg, 0.049 mmol, 0.15 eq.	SrF_2 - 81 mg, 0.644 mmol, 2 eq.	L1 - 13 mg, 0.048 mmol, 0.15 eq.	72 mg, 0.279 mmol, 87%; 69 mg, 0.267 mmol, 83%; 78 mg, 0.302 mmol, 94%
B4	CuBr - 7 mg, 0.049 mmol, 0.15 eq.	SrF_2 - 81 mg, 0.644 mmol, 2 eq.	L2 - 10.5 mg, 0.049 mmol, 0.15 eq.	55 mg, 0.213 mmol, 66%
C1	CuI - 9.2 mg, 0.048 mmol, 0.15 eq.	NaF - 27 mg, 0.643 mmol, 2 eq.	L1 - 13 mg, 0.048 mmol, 0.15 eq.	60 mg, 0.232 mmol, 72%
C2	CuI - 9.2 mg, 0.048 mmol, 0.15 eq.	NaF - 27 mg, 0.643 mmol, 2 eq.	L2 - 10.5 mg, 0.049 mmol, 0.15 eq.	65 mg, 0.252 mmol, 78%
C3	CuI - 9.2 mg, 0.048 mmol, 0.15 eq.	SrF_2 - 81 mg, 0.644 mmol, 2 eq.	L1 - 13 mg, 0.048 mmol, 0.15 eq.	50 mg, 0.194 mmol, 60%
C4	CuI - 9.2 mg, 0.048 mmol, 0.15 eq.	SrF_2 - 81 mg, 0.644 mmol, 2 eq.	L2 - 10.5 mg, 0.049 mmol, 0.15 eq.	64 mg, 0.248 mmol, 77%
D1	CuSCN - 5.9 mg, 0.049 mmol, 0.15 eq.	NaF - 27 mg, 0.643 mmol, 2 eq.	L1 - 13 mg, 0.048 mmol, 0.15 eq.	62 mg, 0.240 mmol, 75%

D2	CuSCN - 5.9 mg, 0.049 mmol, 0.15 eq.	NaF - 27 mg, 0.643 mmol, 2 eq.	L2 - 10.5 mg, 0.049 mmol, 0.15 eq.	62 mg, 0.240 mmol, 75%
D3	CuSCN - 5.9 mg, 0.049 mmol, 0.15 eq.	SrF_2 - 81 mg, 0.644 mmol, 2 eq.	L1 - 13 mg, 0.048 mmol, 0.15 eq.	49 mg, 0.190 mmol, 59%
D4	CuSCN - 5.9 mg, 0.049 mmol, 0.15 eq.	SrF_2 - 81 mg, 0.644 mmol, 2 eq.	L2 - 10.5 mg, 0.049 mmol, 0.15 eq.	53 mg, 0.205 mmol, 64%

All of the reactions performed to a high standard, in particular reaction B3 where >85% yield was observed. Upon repeating reaction B3 to ensure it was reproducible, yields of 83 and 94% were obtained thus validating this hit (average yield of 88%).

Optimization of Copper/Ligand Loading:

General procedure 3.5:

4-Benzoylpiperazin-1-yl benzoate (**1a**) (100 mg, 0.322 mmol, 1 eq.), copper (I) bromide (0.3-0.05 eq.) 4,4'-di-*tert*-butyl-2,2'-bipyridine (0.3-0.05 eq.) and strontium fluoride (81 mg, 0.644 mmol, 2 eq.) were added to a microwave vial and stirred in DMF (3 mL) at room temperature under a nitrogen atmosphere. To the reaction mixture was added trimethyl(trifluoromethyl)silane (95 µL, 0.644 mmol) dropwise and the reaction mixture was stirred at 40 °C for 48 h. The reaction mixture was diluted with EtOAc (10 mL) and washed with 1 M Na_2CO_3 aqueous solution (2 x 20 mL). The combined organics were then dried through a hydrophobic frit, absorbed onto celite and purified by normal phase chromatography (20-100% EtOAc in cyclohexane) using a 12 g silica cartridge over 14 CV. The desired fractions were combined and evaporated *in vacuo* to give the desired product (**2a**) as a colourless oil (or a white solid).

Table S4: Testing the effects of copper/ligand loading on the *N*-trifluoromethylation reaction, following general procedure 3.5.[a] Average yield taken from those shown in Table S3, entry B3.

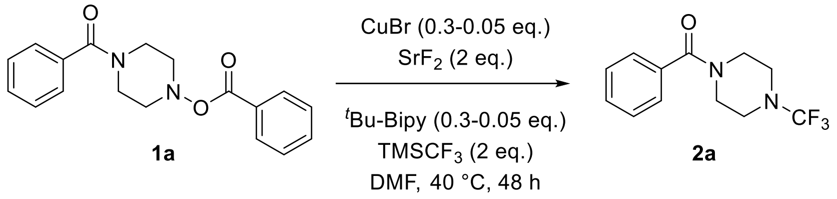

Entry	Quantity of CuBr used	Quantity of ^{t}Bu-Bipy used	Percentage isolated yield of **2a** / %
Initial	7 mg, 0.049 mmol, 0.15 eq.	13 mg, 0.048 mmol 0.15 eq.	88%[a]
A	14 mg, 0.098 mmol, 0.3 eq.	26 mg, 0.097 mmol, 0.3 eq.	54 mg, 0.209 mmol, 65%
B	4.6 mg, 0.032 mmol, 0.1 eq.	8.7 mg, 0.032 mmol, 0.1 eq.	52 mg, 0.2014 mmol, 63%
C	2.3 mg, 0.016 mmol, 0.05 eq.	4.3 mg, 0.016 mmol, 0.05 eq.	52 mg, 0.2014 mmol 63%

It was found that increasing or decreasing the copper/ligand loading from 0.15 eq. was detrimental to yield, however, good yields were still recorded. Consequently, the equivalents of CuBr and ^{t}Bu-Bipy used remained at 0.15.

Optimization of Reaction Concentration:

General procedure 3.6:

4-Benzoylpiperazin-1-yl benzoate (**1a**) (100 mg, 0.322 mmol, 1 eq.), copper (I) bromide (7 mg, 0.049 mmol, 0.15 eq.), 4,4'-di-*tert*-butyl-2,2'-bipyridine (13 mg, 0.048 mmol, 0.15 eq.) and strontium fluoride (81 mg, 0.644 mmol, 2 eq.) were added to a microwave vial and stirred in DMF (1.5-12 mL) at room temperature under a nitrogen atmosphere. To the reaction mixture was added trimethyl(trifluoromethyl)silane (95 µL, 0.644 mmol, 2 eq.) dropwise and the reaction mixture was stirred at 40 °C for 48 h. The reaction mixture was diluted with EtOAc (10 mL) and washed with 1 M Na_2CO_3 aqueous solution (2 x 20 mL). The combined organics were then dried through a hydrophobic frit, absorbed onto celite and purified by normal phase chromatography (20-100% EtOAc in cyclohexane) using a 12 g silica cartridge over 14 CV. The desired fractions were combined and evaporated *in vacuo* to give the desired product (**2a**) as a colourless oil.

Table S5: Testing the effects of concentration on the *N*-trifluoromethylation reaction, following general procedure 3.6.

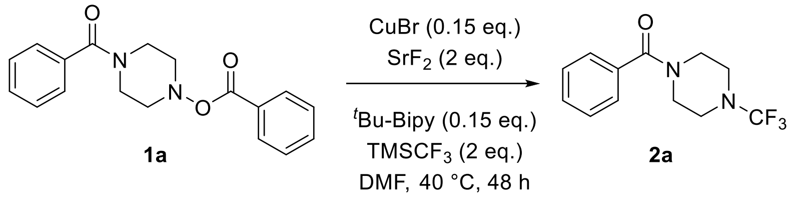

Entry	Volume of DMF used / mL	Percentage isolated yield of **2a** / %
Initial	3 (0.1 M)	88
A	1.5 (0.2 M)	52 mg, 0.2014 mmol, 63%
B	6 (0.05 M)	51 mg, 0.197 mmol, 61%
C	12 (0.025 M)	45 mg, 0.174 mmol, 54%

Both an increase and decrease in the volume of DMF used gave poorer yields, with 12 mL of DMF dropping the yield below 60%. Consequently, as alternative copper/ligand loadings and reaction concentration had only compromised the yield, the conditions highlighted in entry B3 (Table S3) were established as the optimised reaction conditions for the *N*-trifluoromethylation of model substrate **1a**.

4. General Procedure for the Trifluoromethylation of *O*-Benzoylhydroxylamines

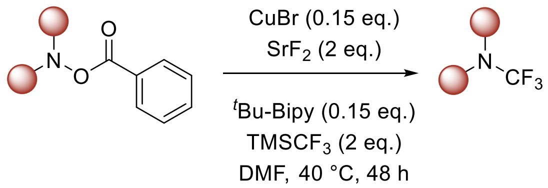

General procedure 4.1:

O-Benzoylhydroxylamine (0.322 mmol, 1 eq.), copper (I) bromide (7 mg, 0.049 mmol, 0.15 eq.), 4,4'-di-*tert*-butyl-2,2'-bipyridine (13 mg, 0.048 mmol, 0.15 eq.) and strontium fluoride (81 mg, 0.644 mmol, 2 eq.) were added to a microwave vial and stirred in DMF (3 mL) at room temperature under a nitrogen atmosphere. To the reaction mixture was added trimethyl(trifluoromethyl)silane (95 µL, 0.644 mmol, 2 eq.) dropwise and the reaction mixture was stirred at 40 °C for 48 h.

Purification A:

The reaction mixture was diluted with EtOAc (10 mL) and washed with 1 M Na_2CO_3 (or 1M NaOH) aqueous solution (2 x 20 mL). The combined organics were then dried through a hydrophobic frit, absorbed onto celite (or evaporated *in vacuo* and then dissolved in DCM) and purified by normal phase chromatography using a silica cartridge over 14 CV. The desired fractions were combined and evaporated *in vacuo* to give the *N*-trifluoromethyl products.

Purification B:

The reaction mixture was blown-down under a flow of nitrogen and then dissolved in EtOAc. The reaction mixture was preabsorbed onto celite and purified by normal phase chromatography using a 12 g silica cartridge over 14 CV. The desired fractions were combined and evaporated *in vacuo* to give the *N*-trifluoromethyl products.

Note: Some of the *N*-CF_3 products were unstable on the LCMS which resulted in multiple peaks seen. Purity was then determined by NMR. In some cases restricted rotation has led to broadening of signals in ^1H and ^{13}C NMR as well as duplicated signals (caused by rotamers). TLC data and GCMS data are reported in some cases for clarity. Where HRMS cannot detect the mass of the desired product, the mass of the carbamoyl fluoride is often found and reported. As reported by Schindler *et. al.*, *N*-CF_3 compounds are not entirely stable under ESI-conditions and unavoidable degradation can be observed.[3] Where the amounts of reagents used differ from general procedure 4.1, the quantities used are detailed.

Phenyl(4-(trifluoromethyl)piperazin-1-yl)methanone (2a)

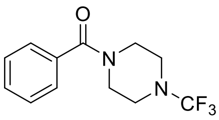

Compound synthesised following general procedure 4.1.

Amount of *O*-benzoylhydroxylamine used:

4-Benzoylpiperazin-1-yl benzoate (**1a**) (100 mg, 0.322 mmol, 1 eq.)

Purification A; The product mixture was preabsorbed onto celite and purified by normal phase chromatography (20-100% EtOAc in cyclohexane) using a 12 g silica cartridge over 14 CV to afford **2a** (72 mg, 87%) as a white solid. **^{1}H NMR (400 MHz, DMSO-*d*$_6$):** δ 7.48-7.40 (m, 5H), 3.80-3.33 (m, 4H), 3.03-2.83 (m, 4H); **^{13}C NMR (151 MHz, DMSO-*d*$_6$) (restricted rotation at the N-CO bond leads to separate signals observed for the piperazine carbons adjacent to this bond):** δ 169.2, 135.4, 129.7, 128.4, 127.0, 124.1 (q, *J* = 255.4 Hz), 46.0, 43.8, 40.4; **^{19}F NMR (376 MHz, DMSO-*d*$_6$):** δ -65.88 (s, 3F); **LCMS (System A):** t_R = 0.99 min, m/z = 259 (MH+); **HRMS (ESI$^+$):** $C_{12}H_{14}F_3N_2O$ $[M+H]^+$ requires 259.1053 found 259.1064; **IR (cm^{-1}):** 2857 (w, C-H), 1625 (s, C=O), 1360.

The reaction was repeated using Purification B to afford the desired product (**2a**) (78 mg, 94%) as a colourless oil and repeated again using purification A to afford the desired product (**2a**) (69 mg, 83%) as a colourless oil, with data matching that recorded, above.

***Tert*-Butyl 4-(trifluoromethyl)piperazine-1-carboxylate (2b)**[4]

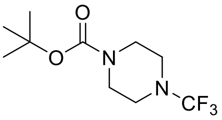

Compound synthesised following general procedure 4.1.

Amounts of *O*-benzoylhydroxylamine, strontium fluoride and $TMSCF_3$ used:

Tert-butyl 4-(benzoyloxy)piperazine-1-carboxylate (**1b**) (100 mg, 0.326 mmol, 1 eq.)

Strontium fluoride (82 mg, 0.653 mmol, 2 eq.)

Trimethyl(trifluoromethyl)silane (96 μL, 0.649 mmol, 2 eq.)

Purification A; The product mixture was dissolved in DCM and purified by normal phase chromatography (0-100% EtOAc in cyclohexane) using a 12 g silica cartridge over 14 CV. The desired fractions were combined and evaporated *in vacuo* to give a colourless oil. NMR showed that benzoic acid was still present. The sample was dissolved in EtOAc (10 mL) and washed with 1 M Na_2CO_3 aqueous solution (2 x 20 mL). The combined organics were then dried through a hydrophobic frit and evaporated *in vacuo* to afford **2b** (44 mg, 53%) as a white solid. **^{1}H NMR (600 MHz, DMSO-*d*$_6$):** δ 3.39 (t, *J* = 5.1 Hz, 4H), 2.83 (t, *J* = 5.1 Hz, 4H), 1.40 (s, 9H); **^{13}C NMR (151 MHz, DMSO-*d*$_6$) (restricted rotation at the N-CO bond leads to separate signals observed for the piperazine carbons adjacent to this bond):** δ 153.6, 123.9 (q, *J* = 254.9 Hz), 79.3, 43.7 (q, *J* = 2.8 Hz), 42.6 (br), 41.7 (br), 28.0; **^{19}F NMR (376 MHz, DMSO-*d*$_6$):** δ -66.02 (s, 3F); **LCMS (System A):** t_R = 1.18 min, m/z = No m/z in keeping with structure of desired product observed; **GCMS (EI):** t_R = 7.57 min, m/z 254.1 (M^+), 198.1 (77.5%), 183.1 (21.5%), 181.1 (56.9%), 154.1 (9.9%), 112 (25.9%), 70.1 (10.2%), 57.1 (100%), 56.1 (14.6%), 41.1 (20.5%); **IR (cm^{-1}):** 2979 (w, C-H), 2869 (w, C-H), 1683 (s, C=O), 1238; **R_f:** 0.4 (20:80 EtOAc/Cyclohexane). (Potassium permanganate stain used to visualise compound). For published data for this product, see ref. 4.

The reaction was repeated using Purification A to afford the desired product (**2b**) (46 mg, 56%) as a white solid, with data matching that recorded, above.

(4-Methoxyphenyl)(4-(trifluoromethyl)piperazin-1-yl)methanone (2c)

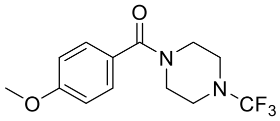

Compound synthesised following general procedure 4.1.

Amount of *O*-benzoylhydroxylamine used:

4-(4-Methoxybenzoyl)piperazin-1-yl benzoate (**1c**) (110 mg, 0.322 mmol, 1 eq.)

Purification A; The product mixture was dissolved in DCM and purified by normal phase chromatography (0-100% EtOAc in cyclohexane) using a 12 g silica cartridge over 14 CV to afford **2c** (67 mg, 72%) as a colourless oil. **^{1}H NMR (400 MHz, DMSO-*d*$_6$):** δ 7.42-7.38 (m, 2H), 7.01-6.97 (m, 2H), 3.80 (s, 3H), 3.64-3.49 (br m, 4H), 2.92 (br t, *J* = 4.9 Hz, 4H); **^{13}C NMR (151 MHz, DMSO-*d*$_6$) (restricted rotation at the N-CO bond leads to separate signals observed for the piperazine carbons adjacent to this bond):** δ 169.2, 160.3, 129.1, 127.3, 124.1 (q, *J* = 255.4 Hz), 113.7, 55.2, 45.8 (br) (restricted rotation of N-CO bond leads to severe broadening), 43.9, 40.8 (br) (restricted rotation of N-CO bond leads to severe broadening); **^{19}F NMR (376 MHz, DMSO-*d*$_6$):** δ -65.93 (s, 3F); **LCMS (System B):** t_R = 0.99 min, m/z = 289 (MH+); **HRMS (ESI$^+$):** $C_{13}H_{16}F_3N_2O_2$ $[M+H]^+$ requires 289.1158 found 289.1158; **IR (cm^{-1}):** 2862 (w, C-H), 1634 (s, C=O), 1608, 1238.

The reaction was repeated using Purification B to afford the desired product (**2c**) (70 mg, 75%) as a colourless oil, with data matching that recorded, above.

***N*-Methyl-1-(methylsulfonyl)-*N*-(trifluoromethyl)piperidin-4-amine (2d)**

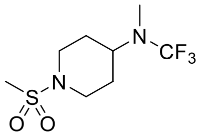

Compound synthesised following general procedure 4.1.

Amounts of *O*-benzoylhydroxylamine and strontium fluoride used:

O-Benzoyl-*N*-methyl-*N*-(1-(methylsulfonyl)piperidin-4-yl)hydroxylamine (**1d**) (100 mg, 0.320 mmol, 1 eq.)

Strontium fluoride (80 mg, 0.640 mmol, 2 eq.)

Purification A; The product mixture was dissolved in DCM and purified by normal phase chromatography (0-100% EtOAc in cyclohexane) using a 12 g silica cartridge over 14 CV to afford **2d** (15 mg, 18%) as a white solid. Tentative yield due to the presence of trace impurities. **^{1}H NMR (400 MHz, $CDCl_3$):** δ 3.93-3.87 (m, 2H), 3.26-3.18 (m, 1H), 2.79 (s, 3H), 2.74-2.68 (m, 2H), 2.51 (q, *J* = 1.5 Hz, 3H), 1.87-1.81 (m, 4H); **^{13}C NMR (101 MHz, $CDCl_3$)**: δ 124.9 (q, *J* = 254.8 Hz), 53.1, 45.7, 35.1, 28.7, 27.6 (q, *J* = 3.1 Hz); **^{19}F NMR (376 MHz, $CDCl_3$):** δ -61.14 (s, 3F); **LCMS (System B):** t_R = 0.79 min, m/z = 261 (MH+); **GCMS (EI):** t_R = 9.34 min**,** m/z 181.1 ([M]-[SO_2Me]$^+$, 100%), 161.1 (35.5%), 138.0 (47.7%), 125.0 (45.6%), 107.0 (45%), 82.0 (98.6%), 79.0 (29.8%), 56.0 (93.6%), 55.0 (53.3%), 42.0 (31.7%); **HRMS (ESI$^+$):** $C_8H_{16}F_3N_2O_2S$ [M+H]$^+$ requires 261.0879 found 261.0885; **IR (cm^{-1}):** 2855 (w, C-H), 1314 (s, S=O), 1294, 1151; **R*f*:** 0.3 (50:50 EtOAc/Cyclohexane). (Potassium permanganate stain used to visualise compound).

The reaction was repeated using Purification A to afford the desired product (**2d**) (13 mg, 16%) as a white solid, with data matching that recorded, above.

Benzyl 4-(trifluoromethyl)-1,4-diazepane-1-carboxylate (2e)

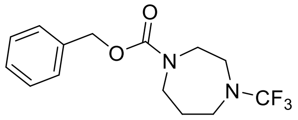

Compound synthesised following general procedure 4.1.

Amount of *O*-benzoylhydroxylamine used:

Benzyl 4-(benzoyloxy)-1,4-diazepane-1-carboxylate (**1e**) (114 mg, 0.322 mmol, 1 eq.)

Purification A; The product mixture was dissolved in DCM and purified by normal phase chromatography (0-100% EtOAc in cyclohexane) using a 12 g silica cartridge over 14 CV. The desired fractions were combined and evaporated *in vacuo* to afford **2e** (48 mg, 49%) as a colourless oil. **^{1}H NMR (400 MHz, DMSO-*d*$_6$)**: δ 7.39-7.29 (m, 5H), 5.09 (m, 2H), 3.54-3.45 (m, 4H), 3.16-3.10 (m, 2H), 3.06 (br t, *J* = 5.4 Hz, 2H), 1.74-1.68 (m, 2H); **^{13}C NMR (151 MHz, DMSO-*d*$_6$)** (rotameric signals observed): δ 155.0 (rot. A), 154.9 (rot. B), 137.0 (rot. A), 136.9 (rot. B), 128.4, 127.8, 127.38 (rot. A), 127.36 (rot. B), 124.4 (q, *J* = 253.2 Hz), 66.18 (rot. A), 66.16 (rot. B), 46.8 (rot. A), 46.4 (rot. B), 46.1 (rot. A), 45.9 (rot. B), 45.4 (rot. A), 45.3 (rot. A), 45.2 (rot. B), 45.0 (rot. B), 28.2 (rot. A), 27.8 (rot. B); **^{19}F NMR (376 MHz, DMSO-*d*$_6$):** δ -59.51 (s, 3F); **LCMS (System B):** t_R = 1.22 min, m/z = No m/z in keeping with structure of desired product observed; **HRMS (ESI$^+$):** Desired product mass not found. Mass of the carbamoyl fluoride decomposition product was found, $C_{14}H_{18}FN_2O_3$ $[M+H]^+$ requires 281.1296 found 281.1295; **GCMS (EI):** t_R = 10.58 min, m/z 282.1 (24.1%), 211.1 ($[M]-[PhCH_2]^+$, 22.8%), 191.1 (21.1%), 148.1 (8.9%), 147.1 (23.1%), 124.1 (11.8%), 92.1 (10.1%), 91.1 ($[PhCH_2]^+$, 100%), 65.1 (10.7%), 42.1 (8.4%); **IR (cm^{-1}):** 2954 (w, C-H), 2871 (w, C-H), 1697 (s, C=O), 1217.

The reaction was repeated using Purification B to afford the desired product (**2e**) (55 mg, 57%) as a colourless oil, with data matching that recorded, above.

2-((1-(Trifluoromethyl)piperidin-3-yl)methyl)isoquinolin-1(2*H*)-one (2f)

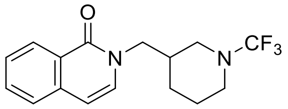

Compound synthesised following general procedure 4.1.

Amount of *O*-benzoylhydroxylamine used:

3-((1-Oxoisoquinolin-2(1*H*)-yl)methyl)piperidin-1-yl benzoate (**1f**) (117 mg, 0.322 mmol, 1 eq.)

Purification A; The product mixture was dissolved in DCM and purified by normal phase chromatography (0-100% EtOAc in cyclohexane) using a 12 g silica cartridge over 14 CV. The desired fractions were combined and evaporated *in vacuo* to afford **2f** (65 mg, 65%) as a colourless oil. Tentative yield due to traces of residual solvent. **^{1}H NMR (400 MHz, DMSO-*d*$_6$):** δ 8.24-8.21 (m, 1H), 7.72-7.68 (m, 1H), 7.66-7.64 (m, 1H), 7.52-7.48 (m, 1H), 7.43 (d, *J* = 7.1 Hz, 1H), 6.63 (d, *J* = 7.1 Hz, 1H), 3.94 (dd, *J* = 13.3, 7.4 Hz, 1H), 3.90 (dd, *J* = 13.3, 7.4 Hz, 1H), 3.12-3.07 (m, 1H), 3.06-3.02 (m, 1H), 2.59-2.53 (m, 1H), 2.44 (t, *J* = 10.6 Hz, 1H), 2.15-2.04 (m, 1H), 1.78-1.71 (m, 1H), 1.68-1.62 (m, 1H), 1.49-1.39 (m, 1H), 1.22-1.12 (m, 1H); **^{13}C NMR (101 MHz, DMSO-*d*$_6$):** δ 161.1, 136.8, 133.2, 132.2, 127.0, 126.6, 126.1, 125.3, 124.4 (q, *J* = 253.3 Hz), 104.9, 50.7, 47.3, 44.4-44.3 (m), 34.7, 26.9, 22.9; **^{19}F NMR (376 MHz, DMSO-*d*$_6$):** δ -65.40 (s, 3F); **LCMS (System B):** t_R = 1.16 min, m/z = No m/z in keeping with structure of desired product observed; **HRMS (ESI$^+$):** Desired product mass not found. Mass of the carbamoyl fluoride decomposition product was found, $C_{16}H_{18}FN_2O_2$ $[M+H]^+$ requires 289.1347 found 289.1342; **GCMS (EI):** t_R = 12.05 min, m/z 310.2 (M^+, 14.7%), 291.2 (7.2%), 224.2 (9.1%), 160.1 (13.9%), 159.1 (100%), 158.2 (9.4%), 150.1 (10.2%), 145.1 (16.8%), 128.1 (21.2%), 118.1 (7.7%); **IR (cm^{-1}):** 2940 (w, C-H), 1649 (s, C=O), 1625, 1248; **R*$_f$*:** 0.4 (50:50 EtOAc/Cyclohexane).

The reaction was repeated using Purification B to afford the desired product (**2f**) (65 mg, 65%) as a yellow oil, with data matching that recorded, above.

***Tert*-butyl 1'-(trifluoromethyl)-3*H*-spiro[benzo[*f*][1,4]oxazepine-2,4'-piperidine]-4(5*H*)-carboxylate (2g)**

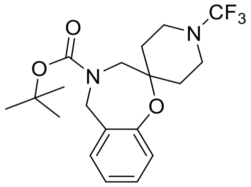

Compound synthesised following general procedure 4.1.

Amount of *O*-benzoylhydroxylamine used:

Tert-butyl 1'-(benzoyloxy)-3*H*-spiro[benzo[*f*][1,4]oxazepine-2,4'-piperidine]-4(5*H*)-carboxylate (**1g**) (141 mg, 0.322 mmol, 1 eq.)

Purification A; The product mixture was dissolved in DCM and purified by normal phase chromatography (0-50% EtOAc in cyclohexane) using a 12 g silica cartridge over 14 CV. The desired fractions were combined and evaporated *in vacuo* to afford **2g** (77 mg, 62%) as a colourless oil. **^{1}H NMR (400 MHz, DMSO-*d*$_6$):** δ 7.27-7.22 (m, 2H), 7.09-7.05 (m, 2H), 4.36 (s, 2H), 3.60 (s, 2H), 3.09-3.00 (br m, 2H), 3.00-2.90 (br m, 2H), 1.69-1.54 (br m, 4H), 1.37 (s, 9H); **^{13}C NMR (151 MHz, DMSO-*d*$_6$)** (rotameric signals observed): δ 154.3 (rot. A), 153.7 (rot. A), 153.6 (rot. B), 153.5 (rot. B), 132.1 (rot. A), 131.7 (rot. B), 129.6 (rot. A), 129.1 (rot. B), 128.7, 124.6 (q, *J* = 256.7 Hz), 123.60 (rot. A), 123.57 (rot. B), 123.0 (rot. A), 122.9 (rot. B), 79.3 (rot. A), 79.1 (rot. B), 77.4 (rot. A), 77.1 (rot. B), 56.2 (rot. A), 55.5 (rot. B), 49.9 (rot. A), 49.4 (rot. B), 39.74 (rot. A), 39.73 (rot. B) (overlap with DMSO-*d*$_6$ signal), 30.9 (rot. A), 30.8 (rot. B), 27.93 (rot. A), 27.87 (rot. B); **^{19}F NMR (376 MHz, DMSO-*d*$_6$)** (rotameric signals observed): δ -64.96 (rot. A), -65.26 (rot. B); **LCMS (System B):** t_R = 1.45 min, m/z = No m/z in keeping with structure of desired product observed; **HRMS (ESI$^+$):** Desired product mass not found. Mass of the carbamoyl fluoride decomposition product was found, $C_{19}H_{26}FN_2O_4$ $[M+H]^+$ requires 365.1871 found 365.1865; **GCMS (EI):** t_R = 11.77 min, m/z 330.1 ([M] -[^{t}Bu]$^+$, 96.4%), 310.1 (39.2%), 286.1 (24.9%), 285.1 (43.2%), 265.1 (27.2%), 188.1 (30.8%), 164.1 (27.1%), 163.1 (39.3%), 107.1 (100%), 57.1 ([^{t}Bu]$^+$, 81.4%); **IR (cm^{-1}):** 2979 (w, C-H), 2930 (w, C-H), 1693 (s, C=O), 1226; **R$_f$:** 0.5 (20:80 EtOAc/Cyclohexane).

The reaction repeated using Purification B to afford the desired product (**2g**) (70 mg, 56%) as a colourless oil, with data matching that recorded, above.

1,1,1-Trifluoro-*N*-methyl-*N*-(2-(thiophen-2-yl)benzyl)methanamine (2h)

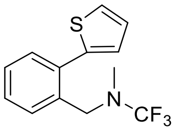

Compound synthesised following general procedure 4.1.

Amount of *O*-benzoylhydroxylamine used:

O-Benzoyl-*N*-methyl-*N*-(2-(thiophen-2-yl)benzyl)hydroxylamine (**1h**) (104 mg, 0.322 mmol, 1 eq.)

Purification A; The product mixture was dissolved in DCM and purified by normal phase chromatography (0-50% EtOAc in cyclohexane) using a 12 g silica cartridge over 14 CV. The desired fractions were combined and evaporated *in vacuo* to afford **2h** (21 mg, 24%) as a colourless oil. Tentative yield due to traces of residual solvent. **^{1}H NMR (600 MHz, DMSO-*d*$_6$):** δ 7.63 (dd, *J* = 5.0, 1.3 Hz, 1H), 7.53-7.52 (m, 1H), 7.45-7.42 (m, 1H), 7.41-7.36 (m, 2H), 7.17 (dd, *J* = 3.7, 1.3 Hz, 1H), 7.16-7.15 (m, 1H), 4.08 (s, 2H), 2.37 (s, 3H); **^{13}C NMR (151 MHz, DMSO-*d*$_6$):** δ 140.4, 134.2, 133.8, 130.8, 128.8, 128.3, 127.6, 127.533, 127.526, 126.6, 125.2 (q, *J* = 254.9 Hz), 49.6 (q, *J* = 2.2 Hz), 33.3 (q, *J* = 2.8 Hz); **^{19}F NMR (376 MHz, DMSO-*d*$_6$):** δ -63.71 (s, 3F); **LCMS (System B):** t_R = 1.42 min, m/z = No m/z in keeping with structure of desired product observed; **GCMS (EI):** t_R = 9.29 min, m/z 271.1 (M^+, 100%), 256.1 (19.1%), 202.1 (92.4%), 174.1 (17.6%), 173.1 (81.5%), 172.1 (24.3%), 171 (70.5%), 129.1 (33.8%), 128.1 (24.9%), 115.1 (17.7%); **IR (cm^{-1}):** 3070 (w, C-H), 2911 (w, C-H), 1242, 1058. *HRMS data could not be obtained for this compound, presumably due to the instability of this species under ESI conditions.*[3]

***Tert*-butyl 2-(trifluoromethyl)-2,3-dihydro-1*H*-spiro[isoquinoline-4,4'-piperidine]-1'-carboxylate (2i)**

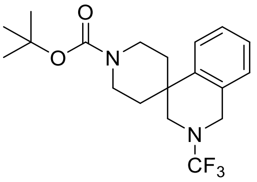

Compound synthesised following general procedure 4.1.

Amount of *O*-benzoylhydroxylamine used:

Tert-butyl 2-(benzoyloxy)-2,3-dihydro-1*H*-spiro[isoquinoline-4,4'-piperidine]-1'-carboxylate (**1i**) (136 mg, 0.322 mmol, 1 eq.)

Purification B; The product mixture was preabsorbed onto celite and purified by normal phase chromatography (0-50% EtOAc in cyclohexane) using a 12 g silica cartridge over 14 CV. The desired fractions were combined and evaporated *in vacuo* to afford **2i** (26 mg, 22%) as a colourless oil. **^{1}H NMR (600 MHz, DMSO-*d*$_6$):** δ 7.42 (br d, *J* = 7.4 Hz, 1H), 7.26-7.23 (m, 1H), 7.20-7.17 (m, 1H), 7.16-7.15 (m, 1H), 4.14 (s, 2H), 3.96-3.79 (br m, 2H), 3.22 (s, 2H), 3.09-2.84 (br m, 2H), 1.85 (td, *J* = 13.5, 5.0 Hz, 2H), 1.63 (br d, *J* = 13.5 Hz, 2H), 1.43 (s, 9H); **^{13}C NMR (151 MHz, DMSO-*d*$_6$):** δ 154.1, 141.6, 131.4, 127.1, 126.5, 126.1, 125.9, 124.6 (q, *J* = 254.3 Hz), 78.6, 46.9 (signals overlap, as determined by HSQC), 38.7 (br), 36.0, 34.4 (br), 28.1; **^{19}F NMR (376 MHz, DMSO-*d*$_6$):** δ -65.20 (s, 3F); **LCMS (System B):** t_R = 1.49 min, m/z = No m/z in keeping with structure of desired product observed; **HRMS (ESI$^+$):** $C_{19}H_{26}F_3N_2O_2$ [M+H]$^+$ requires 371.1941 found 371.1932; **IR (cm^{-1}):** 2979 (w, C-H), 2923 (w, C-H), 1683 (s, C=O), 1242; **R*$_f$*:** 0.5 (20:80 EtOAc/Cyclohexane). (Potassium permanganate stain used to visualise compound).

The reaction was repeated using Purification A to afford the desired product (**2i**) (20 mg, 17%) as a colourless oil, with data matching that recorded, above. For the isolation of the by-product **3a** within the same reaction, see the *Isolated side-products* section (pages 81-82).

2-Methyl-2-(*m*-tolyl)-4-(trifluoromethyl)morpholine (2j)

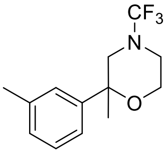

Compound synthesised following general procedure 4.1.

Amount of *O*-benzoylhydroxylamine used:

2-Methyl-2-(*m*-tolyl)morpholino benzoate (**1j**) (100 mg, 0.322 mmol, 1 eq.)

Purification A; The product mixture was dissolved in DCM and purified by normal phase chromatography (0-20% EtOAc in cyclohexane) using a 12 g silica cartridge over 14 CV. The desired fractions were combined and evaporated *in vacuo* to afford **2j** (61 mg, 73%) as a colourless oil. **^{1}H NMR (600 MHz, $CDCl_3$):** δ 7.29 (t, *J* = 7.6 Hz, 1H), 7.26 (s, 1H), 7.23 (d, *J* = 7.6 Hz, 1H), 7.12 (d, *J* = 7.6 Hz, 1H), 3.80 (dt, *J* = 11.9, 3.9 Hz, 1H), 3.71-3.67 (m, 1H), 3.54 (d, *J* = 12.1 Hz, 1H), 2.93-2.90 (m, 3H), 2.40 (s, 3H), 1.49 (s, 3H); **^{13}C NMR (151 MHz, $CDCl_3$):** δ 143.2, 138.1, 128.4, 127.9, 126.4, 124.1 (q, *J* = 255.4 Hz), 122.8, 74.6, 60.6, 51.3 (q, *J* = 2.6 Hz), 44.2 (q, *J* = 2.8 Hz), 28.1, 21.6; **^{19}F NMR (376 MHz, $CDCl_3$):** δ -68.93 (s, 3F); **LCMS (System B):** t_R = 1.32 min, m/z = 260 (MH+); **HRMS (ESI$^+$):** $C_{13}H_{17}F_3NO$ $[M+H]^+$ requires 260.1257 found 260.1267; **IR (cm^{-1}):** 2978 (w, C-H), 2863 (w, C-H), 1360, 1241.

The reaction was repeated using Purification A to afford the desired product (**2j**) (51 mg, 61%) as a colourless oil, with data matching that recorded, above.

6-Chloro-7,8-dimethoxy-1-(4-methoxyphenyl)-3-(trifluoromethyl)-2,3,4,5-tetrahydro-1*H*-benzo[*d*]azepine (2k)

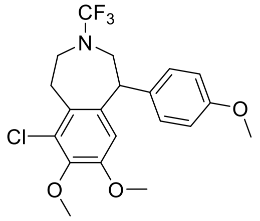

Compound synthesised following general procedure 4.1.

Amount of *O*-benzoylhydroxylamine used:

6-Chloro-7,8-dimethoxy-1-(4-methoxyphenyl)-1,2,4,5-tetrahydro-3*H*-benzo[*d*]azepin-3-yl benzoate (**1k**) (151 mg, 0.322 mmol, 1 eq.)

Purification A; The product mixture was dissolved in DCM and purified by normal phase chromatography (0-100% EtOAc in cyclohexane) using a 12 g silica cartridge over 14 CV. The desired fractions were combined and evaporated *in vacuo* to give a colourless oil. LCMS showed that a benzoate impurity was present. The sample was dissolved in DCM (10 mL) and was washed with 1 M NaOH aqueous solution (2 x 20 mL). The combined organics were then dried through a hydrophobic frit and evaporated *in vacuo* to give a colourless oil. LCMS showed that the benzoate impurity was still present. The sample was dissolved in MeOH and purified by solid-phase extraction using a 2 g aminopropyl (NH_2) cartridge, eluting with 4 CV MeOH. The filtrate was evaporated *in vacuo* to afford **2k** (55 mg, 41%) as a white solid. **^{1}H NMR (400 MHz, DMSO-*d*$_6$):** δ 7.05-7.03 (m, 2H), 6.92-6.88 (m, 2H), 6.70 (s, 1H), 4.47 (br dd, *J* = 7.6, 3.7 Hz, 1H), 3.74 (s, 3H), 3.73 (s, 3H), 3.69 (s, 3H), 3.68-3.62 (br m, 1H), 3.34-3.31 (br m, 1H), 3.27-3.24 (br m, 1H), 3.20-3.16 (br m, 1H), 2.97-2.85 (br m, 2H); **^{13}C NMR (151 MHz, DMSO-*d*$_6$):** δ 157.7, 151.2, 143.1, 139.2, 132.9, 129.0, 128.6, 127.5, 124.4 (q, *J* = 254.3 Hz), 113.9, 113.7, 59.9, 55.8, 55.0, 49.3, 48.8, 45.2, 28.3; **^{19}F NMR (376 MHz, DMSO-*d*$_6$):** δ -62.28 (s, 3F); **LCMS (System B):** t_R = 1.51 min, m/z = 416 (MH+); **HRMS (ESI$^+$):** $C_{20}H_{22}ClF_3NO_3$ $[M+H]^+$ requires 416.1235 found 416.1239; **IR (cm^{-1}):** 2939 (w, C-H), 2840 (w, C-H), 1513, 1213; **M.pt. (MeOH):** 116-118 °C.

The reaction was repeated using Purification A to afford the desired product (**2k**) (63 mg, 47%) as a white solid, with data matching that recorded, above.

Furan-2-yl(4-(trifluoromethyl)piperazin-1-yl)methanone (2l)

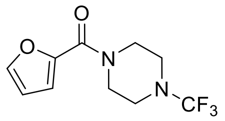

Compound synthesised following general procedure 4.1.

Amount of *O*-benzoylhydroxylamine used:

4-(Furan-2-carbonyl)piperazin-1-yl benzoate (**1l**) (96.7 mg, 0.322 mmol, 1 eq.)

Purification A; The product mixture was dissolved in DCM and purified by normal phase chromatography (0-80% EtOAc in cyclohexane) using a 12 g silica cartridge over 14 CV. The desired fractions were combined and evaporated *in vacuo* to afford **2l** (42 mg, 53%) as a yellow oil. **^{1}H NMR (400 MHz, DMSO-*d*$_6$):** δ 7.85 (m, 1H), 7.04 (d, *J* = 3.5 Hz, 1H), 6.64 (dd, *J* = 3.5, 2.0 Hz, 1H), 3.83-3.66 (br m, 4H), 2.95 (t, *J* = 4.9 Hz, 4H); **^{13}C NMR (101 MHz, DMSO-*d*$_6$):** δ 158.4, 146.6, 144.9, 124.0 (q, *J* = 254.8 Hz), 115.9, 111.3, 43.9, 42.9 (br); **^{19}F NMR (376 MHz, DMSO-*d*$_6$):** δ -66.02 (s, 3F); **LCMS (System B):** t_R = 0.92 min, m/z = 249 (MH+); **HRMS (ESI$^+$):** $C_{10}H_{12}F_3N_2O_2$ $[M+H]^+$ requires 249.0845 found 249.0857; **IR (cm^{-1}):** 2911 (w, C-H), 2865 (w, C-H), 1627 (s, C=O), 1244.

The reaction was repeated using Purification A to afford the desired product (**2l**) (49 mg, 61%) as a colourless oil, with data matching that recorded, above.

(±)-Ethyl *trans*-4-phenyl-1-(trifluoromethyl)piperidine-3-carboxylate (2m)

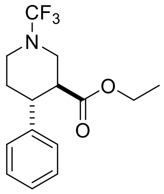

Compound synthesised following general procedure 4.1.

Amount of *O*-benzoylhydroxylamine used:

Ethyl 1-(benzoyloxy)-4-phenylpiperidine-3-carboxylate; *trans*:*cis* 80:20 (**1m**) (114 mg, 0.322 mmol, 1 eq.)

Purification A; The product mixture was dissolved in DCM and purified by normal phase chromatography (0-20% EtOAc in cyclohexane) using a 12 g silica cartridge over 14 CV. The desired fractions were combined and evaporated *in vacuo* to afford **2m** (45 mg, 46%) as a colourless oil. Tentative yield due to the presence of trace impurities. **^{1}H NMR (600 MHz, $CDCl_3$):** δ 7.32-7.29 (m, 2H), 7.24-7.20 (m, 3H), 3.94-3.86 (m, 2H), 3.58-3.56 (m, 1H), 3.45 (dquin, J = 11.9, 2.2 Hz, 1H), 2.89-2.79 (m, 3H), 2.73 (td, J = 11.9, 2.2 Hz, 1H), 1.96-1.82 (m, 2H), 0.95 (t, J = 7.0 Hz, 3H); **^{13}C NMR (151 MHz, $CDCl_3$):** δ 172.2, 142.3, 128.5, 127.3, 127.0, 124.3 (q, J = 256.0 Hz), 60.4, 48.0, 46.9 (q, J = 2.8 Hz), 45.2, 44.8 (q, J = 2.8 Hz), 31.9, 13.8; **^{19}F NMR (376 MHz, DMSO-d_6):** δ -66.67 (s, 3F); **LCMS (System B):** t_R = 1.35 min, m/z = No m/z in keeping with structure of desired product observed; **HRMS (ESI$^+$):** $C_{15}H_{19}F_3NO_2$ [M+H]$^+$ requires 302.1362 found 302.1374; **IR (cm^{-1}):** 2983 (w, C-H), 2857 (w, C-H), 1729 (s, C=O), 1226.

The reaction was repeated using Purification A to afford the desired product (**2m**) (44 mg, 45%) as a colourless oil, with data matching that recorded, above.

3-Phenyl-8-(trifluoromethyl)-1-oxa-2,8-diazaspiro[4.5]dec-2-ene (2n)

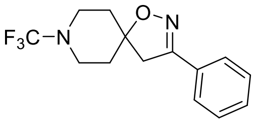

Compound synthesised following general procedure 4.1.

Amount of *O*-benzoylhydroxylamine used:

3-Phenyl-1-oxa-2,8-diazaspiro[4.5]dec-2-en-8-yl benzoate (**1n**) (114 mg, 0.322 mmol, 1 eq.)

Purification A; The product mixture was dissolved in DCM and purified by normal phase chromatography (0-10% EtOAc in cyclohexane) using a 12 g silica cartridge over 14 CV. The desired fractions were combined and evaporated *in vacuo* to afford **2n** (45 mg, 49%) as a white solid. **^{1}H NMR (600 MHz, $CDCl_3$):** δ 7.68-7.64 (m, 2H), 7.44-7.40 (m, 3H), 3.13-3.11 (m, 6H), 2.04-2.01 (m, 2H), 1.90-1.85 (m, 2H); **^{13}C NMR (151 MHz, $CDCl_3$):** δ 156.2, 130.1, 129.7, 128.7, 126.5, 124.5 (q, *J* = 256.0 Hz), 83.4, 45.4, 41.6 (q, *J* = 2.8 Hz), 34.9; **^{19}F NMR (376 MHz, $CDCl_3$):** δ -66.90 (s, 3F); **LCMS (System A):** t_R = 1.23 min, m/z = 285 (MH+); **HRMS (ESI$^+$):** $C_{14}H_{16}F_3N_2O$ $[M+H]^+$ requires 285.1209 found 285.1222; **IR (cm^{-1}):** 2951 (w, C-H), 2869 (w, C-H), 1354, 1250; **M.pt. (EtOAc/cyclohexane):** 121-124 °C.

The reaction was repeated using Purification A to afford the desired product (**2n**) (37 mg, 40%) as a white solid, with data matching that recorded, above.

3-(Pyridin-3-ylmethyl)-8-(trifluoromethyl)-1-oxa-3,8-diazaspiro[4.5]decan-2-one (2o)

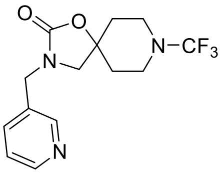

Compound synthesised following general procedure 4.1.

Amount of *O*-benzoylhydroxylamine used:

2-oxo-3-(pyridin-3-ylmethyl)-1-oxa-3,8-diazaspiro[4.5]decan-8-yl benzoate (**1o**) (118 mg, 0.322 mmol, 1 eq.)

Purification A; The product mixture was dissolved in DCM and purified by normal phase chromatography (50-100% 3:1 EtOAc:EtOH in cyclohexane) using a 12 g silica cartridge over 14 CV. The desired fractions were combined and evaporated *in vacuo* to afford **2o** (45 mg, 44%) as a yellow oil. **^{1}H NMR (600 MHz, $CDCl_3$):** δ 8.60 (br d, *J* = 4.3 Hz, 1H), 8.54 (s, 1H), 7.67 (br dt, *J* = 8.0, 1.8 Hz, 1H), 7.33 (dd, *J* = 8.0, 4.3 Hz, 1H), 4.46 (s, 2H), 3.19 (s, 2H), 3.11 (dt, *J* = 12.1, 4.0 Hz, 2H), 3.04-3.00 (m, 2H), 1.95-1.93 (m, 2H), 1.77-1.72 (m, 2H); **^{13}C NMR (151 MHz, $CDCl_3$):** δ 156.8, 149.6, 149.2, 136.0, 131.3, 124.3 (q, *J* = 256.0 Hz), 124.0, 76.1, 54.7, 45.8, 40.5 (q, *J* = 2.8 Hz), 35.0; **^{19}F NMR (376 MHz, $CDCl_3$):** δ -66.99 (s, 3F); **LCMS (System B):** t_R = 0.87 min, m/z = 316 (MH+); **HRMS (ESI$^+$):** $C_{14}H_{17}F_3N_3O_2$ $[M+H]^+$ requires 316.1267 found 316.1272; **IR (cm^{-1}):** 2928 (w, C-H), 2870 (w, C-H), 1740 (s, C=O), 1244.

The reaction was repeated using Purification A to afford the desired product (**2o**) (49 mg, 48%) as a yellow oil, with data matching that recorded, above.

1-(3-Nitropyridin-2-yl)-4-(trifluoromethyl)piperazine (2p)

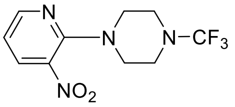

Compound synthesised following general procedure 4.1.

Amount of *O*-benzoylhydroxylamine used:

4-(3-nitropyridin-2-yl)piperazin-1-yl benzoate (**1p**) (106 mg, 0.322 mmol, 1 eq.)

Purification A; The product mixture was dissolved in DCM and purified by normal phase chromatography (0-100% EtOAc in cyclohexane) using a 12 g silica cartridge over 14 CV. The desired fractions were combined and evaporated *in vacuo* to give a yellow solid. LCMS showed that there was ligand present. The sample was dissolved in DCM and purified by normal phase chromatography (0-50% EtOAc in cyclohexane) using a 12 g silica cartridge over 14 CV. The desired fractions were combined and evaporated *in vacuo* to afford **2p** (34 mg, 38%) as a yellow oil. **^{1}H NMR (400 MHz, $CDCl_3$):** δ 8.37 (dd, *J* = 4.4, 1.8 Hz, 1H), 8.17 (dd, *J* = 8.0, 1.8 Hz, 1H), 6.84 (dd, *J* = 8.0, 4.4 Hz, 1H), 3.52 (t, *J* = 5.2 Hz, 4H), 3.06 (t, *J* = 5.2 Hz, 4H); **^{13}C NMR (101 MHz, $CDCl_3$):** δ 152.6, 151.8, 135.6, 133.6, 124.2 (q, *J* = 256.4 Hz), 114.4, 47.2, 44.0; **^{19}F NMR (376 MHz, $CDCl_3$):** δ -68.00 (s, 3F); **LCMS (System B):** t_R = 1.17 min, m/z = No m/z in keeping with structure of desired product observed; **HRMS (ESI$^+$):** $C_{10}H_{12}F_3N_4O_2$ $[M+H]^+$ requires 277.0907 found 277.0908; **IR (cm^{-1}):** 2908 (w, C-H), 2864 (w, C-H), 1596 (s, N-O), 1234.

The reaction was repeated using Purification A to afford the desired product (**2p**) (36 mg, 40%) as a yellow oil, with data matching that recorded, above.

(1*R*,3*s*,5*S*)-3-(4-Chloro-3-methoxyphenyl)-8-(trifluoromethyl)-8-azabicyclo[3.2.1]octane (2q)

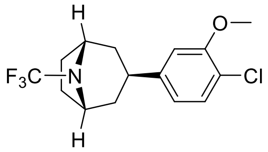

Compound synthesised following general procedure 4.1.

Amount of *O*-benzoylhydroxylamine used:

(1*R*,3*s*,5*S*)-3-(4-chloro-3-methoxyphenyl)-8-azabicyclo[3.2.1]octan-8-yl benzoate (**1q**) (120 mg, 0.322 mmol, 1 eq.)

Purification A; The product mixture was dissolved in DCM and purified by normal phase chromatography (0-15% EtOAc in cyclohexane) using a 12 g silica cartridge over 14 CV. The desired fractions were combined and evaporated *in vacuo* afford **2q** (19 mg, 18%) as a white solid. **^{1}H NMR (400 MHz, $CDCl_3$):** δ 7.28 (d, *J* = 7.9 Hz, 1H), 6.82-6.79 (m, 2H), 3.94-3.91 (m, 5H), 3.06-2.98 (m, 1H), 2.54-2.47 (m, 2H), 2.08-2.01 (m, 2H), 1.63-1.48 (m, 4H); **^{13}C NMR (151 MHz, $CDCl_3$):** δ 154.7, 145.2, 129.9, 124.9 (q, *J* = 255.4 Hz), 120.0, 119.9, 111.7, 56.1, 52.9 (q, *J* = 1.7 Hz), 39.2, 33.5, 31.4; **^{19}F NMR (376 MHz, $CDCl_3$):** δ -60.40 (s, 3F); **LCMS (System B):** t_R = 1.47 min, m/z = 320 (MH+); **HRMS (ESI$^+$):** Desired product mass not found. Mass of the carbamoyl fluoride decomposition product was found, $C_{15}H_{18}ClFNO_2$ $[M+H]^+$ requires 298.1005 found 298.1007; **IR (cm^{-1}):** 2978 (w, C-H), 2954 (w, C-H), 1584, 1204; **M.pt. ($CDCl_3$):** 84-87 °C; **$[\alpha_D]^{17\,°C}$ (*c* 1, CDCl3):** -3.3 °.

The reaction was repeated using Purification A to afford the desired product (**2q**) (19 mg, 18%) as a white solid, with data matching that recorded, above.

7-(Methylsulfonyl)-3-(trifluoromethyl)-2,3,4,5-tetrahydro-1*H*-benzo[*d*]azepine (2r)

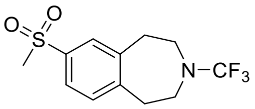

Compound synthesised following general procedure 4.1.

Amount of *O*-benzoylhydroxylamine used:

7-(Methylsulfonyl)-1,2,4,5-tetrahydro-3*H*-benzo[*d*]azepin-3-yl benzoate (**1r**) (111 mg, 0.322 mmol, 1 eq.)

Purification A; The product mixture was dissolved in DCM and purified by normal phase chromatography (0-100% EtOAc in cyclohexane) using a 12 g silica cartridge over 14 CV. The desired fractions were combined and evaporated *in vacuo* afford **2r** (53 mg, 56%) as a white solid. **^{1}H NMR (400 MHz, $CDCl_3$):** δ 7.73 (dd, *J* = 7.4, 2.0 Hz, 1H), 7.70 (d, *J* = 2.0 Hz, 1H), 7.32 (d, *J* = 7.4 Hz, 1H), 3.16-3.13 (m, 4H), 3.09-3.06 (m, 4H), 3.05 (s, 3H); **^{13}C NMR (101 MHz, $CDCl_3$):** δ 147.3, 142.5, 138.8, 130.4, 128.0, 125.8, 124.3 (q, *J* = 256.4 Hz), 46.3 (m), 46.1 (m), 44.5, 36.6, 36.5; **^{19}F NMR (376 MHz, $CDCl_3$):** δ -63.81 (s, 3F); **LCMS (System B):** t_R = 1.04 min, m/z = No m/z in keeping with structure of desired product observed; **HRMS (ESI^+):** Desired product mass not found. Mass of the carbamoyl fluoride decomposition product was found, $C_{12}H_{18}FN_2O_3S$ $[M+NH_4]^+$ requires 289.1016 found 289.1018; **IR (cm^{-1}):** 2913 (w, C-H), 2860 (w, C-H), 1385, 1299 (s, S=O); **M.pt. (EtOAc/cyclohexane):** 115-118 °C.

The reaction was repeated using Purification A to afford the desired product (**2r**) (45 mg, 48%) as a white solid, with data matching that recorded, above.

Benzyl 3-methyl-4-(trifluoromethyl)piperazine-1-carboxylate (2s)

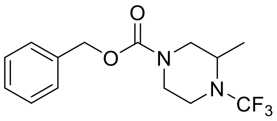

Compound synthesised following general procedure 4.1.

Amount of *O*-benzoylhydroxylamine used:

Benzyl 4-(benzoyloxy)-3-methylpiperazine-1-carboxylate (**1s**) (114 mg, 0.322 mmol, 1 eq.)

Purification A; The product mixture was dissolved in DCM and purified by normal phase chromatography (0-50% EtOAc in cyclohexane) using a 12 g silica cartridge over 14 CV. The desired fractions were combined and evaporated *in vacuo* afford **2s** (23 mg, 24%) as a colourless oil. **^{1}H NMR (400 MHz, $CDCl_3$):** δ 7.40-7.32 (m, 5H), 5.18 (d, *J* = 12.3 Hz, 1H), 5.14 (d, *J* = 12.3 Hz, 1H), 4.10-3.85 (br m, 1H), 3.75 (br d, *J* = 13.3 Hz, 1H), 3.56-3.42 (br m, 1H), 3.28 (br dd, *J* = 13.3, 3.5 Hz, 1H), 3.23-3.03 (br m, 2H), 3.02-2.90 (br m, 1H), 1.19 (br d, *J* = 4.9 Hz, 3H); **^{13}C NMR (151 MHz, $CDCl_3$)** (rotameric signals observed): δ 155.5, 136.5, 128.5, 128.1, 127.9, 124.1 (q, *J* = 256.0 Hz), 67.4, 48.8 (rot. A, br), 48.5 (rot. B, br), 48.0 (rot. A, br), 47.7 (rot. B, br), 43.1 (rot. A, br), 42.9 (rot. B, br), 39.0 (rot. A, br), 38.8 (rot. B, br), 13.9 (rot. A, br), 13.7 (rot. B, br); **^{19}F NMR (376 MHz, $CDCl_3$):** δ -61.34 (s, 3F); **LCMS (System A):** t_R = 1.23 min, m/z = 303 (MH+); **HRMS (ESI$^+$):** $C_{14}H_{17}F_2N_2O_2$ $[M-F]^+$ requires 283.1253 found 283.1255; **IR (cm^{-1}):** 2980 (w, C-H), 2875 (w, C-H), 1699 (s, C=O), 1223.

The reaction was repeated using Purification A to afford the desired product (**2s**) (21 mg, 22%) as a colourless oil, with data matching that recorded, above.

3-(4-(Trifluoromethyl)piperazin-1-yl)pyrazine-2-carbonitrile (2t)

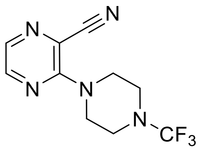

Compound synthesised following general procedure 4.1.

Amount of *O*-benzoylhydroxylamine used:

4-(3-Cyanopyrazin-2-yl)piperazin-1-yl benzoate (**1t**) (99.6 mg, 0.322 mmol, 1 eq.)

Purification A; The product mixture was dissolved in DCM and purified by normal phase chromatography (0-50% EtOAc in cyclohexane) using a 12 g silica cartridge over 14 CV. The desired fractions were combined and evaporated *in vacuo* to give a yellow oil. LCMS showed that a significant amount of dtbbpy ligand was still present. The sample was dissolved in Et_2O (10 mL) and was washed with 20% $CuSO_4$ aqueous solution (2 x 20 mL). The combined organics were then dried through a hydrophobic frit and evaporated *in vacuo* to afford **2t** (33 mg, 40%) as a yellow oil. **^{1}H NMR (400 MHz, $CDCl_3$):** δ 8.30 (d, *J* = 2.3 Hz, 1H), 8.09 (d, *J* = 2.3 Hz, 1H), 3.87 (br t, *J* = 5.0 Hz, 4H), 3.10 (br t, *J* = 5.0 Hz, 4H); **^{13}C NMR (101 MHz, $CDCl_3$):** δ 156.9, 144.9, 135.7, 124.1 (q, *J* = 256.8 Hz), 116.5, 116.4, 46.4, 44.0 (q, *J* = 2.7 Hz); **^{19}F NMR (376 MHz, $CDCl_3$):** δ -68.11 (s, 3F); **LCMS (System A):** t_R = 1.08 min, m/z = 258 (MH+); **HRMS (ESI$^+$):** $C_{10}H_{11}F_3N_5$ $[M+H]^+$ requires 258.0961 found 258.0966; **IR (cm^{-1}):** 2912 (w, C-H), 2864 (w, C-H), 2223 (w, C≡N), 1556.

The reaction was repeated using Purification A to afford the desired product (**2t**) (33 mg, 40%) as a yellow oil, with data matching that recorded, above.

Quinoxalin-6-yl(4-(trifluoromethyl)piperazin-1-yl)methanone (2u)

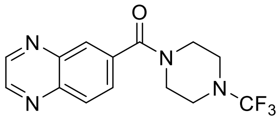

Compound synthesised following general procedure 4.1.

Amount of *O*-benzoylhydroxylamine used:

4-(Quinoxaline-6-carbonyl)piperazin-1-yl benzoate (**1u**) (117 mg, 0.322 mmol, 1 eq.)

Purification A; The product mixture was dissolved in DCM and purified by normal phase chromatography (80-100% EtOAc in cyclohexane) using a 12 g silica cartridge over 14 CV. The desired fractions were combined and evaporated *in vacuo* to afford **2u** (43 mg, 43%) as a white solid. **^{1}H NMR (400 MHz, $CDCl_3$):** δ 8.90-8.88 (m, 2H), 8.17 (d, *J* = 8.6 Hz, 1H), 8.12 (d, *J* = 1.7 Hz, 1H), 7.80 (dd, *J* = 8.6, 1.7 Hz, 1H), 4.13-3.40 (br m, 4H), 3.20-2.74 (br m, 4H); **^{13}C NMR (101 MHz, $CDCl_3$) (restricted rotation at the N-CO bond leads to separate signals observed for the piperazine carbons adjacent to this bond):** δ 168.9, 146.0, 145.9, 143.3, 142.3, 136.5, 130.4, 128.5, 128.1, 123.9 (q, *J* = 256.9 Hz), 46.5 (br), 44.3 (br), 41.2 (br); **^{19}F NMR (376 MHz, $CDCl_3$):** δ -68.02 (s, 3F); **LCMS (System B):** t_R = 0.86 min, m/z = 311 (MH+); **HRMS (ESI$^+$):** $C_{14}H_{14}F_3N_4O$ $[M+H]^+$ requires 311.1114 found 311.1121; **IR (cm^{-1}):** 2937 (w, C-H), 2863 (w, C-H), 1631 (s, C=O), 1234; **M.pt. (EtOAc/cyclohexane):** 98-100 °C.

The reaction was repeated using Purification A to afford the desired product (**2u**) (48 mg, 48%) as a white solid, with data matching that recorded, above.

3-Methoxy-4-((1-(trifluoromethyl)piperidin-4-yl)oxy)benzonitrile (2v)

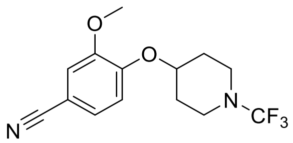

Compound synthesised following general procedure 4.1.

Amount of *O*-benzoylhydroxylamine used:

4-(4-Cyano-2-methoxyphenoxy)piperidin-1-yl benzoate (**1v**) (113 mg, 0.322 mmol, 1 eq.)

Purification A; The product mixture was dissolved in DCM and purified by normal phase chromatography (0-20% EtOAc in cyclohexane) using a 12 g silica cartridge over 14 CV. The desired fractions were combined and evaporated *in vacuo* to give a colourless oil. LCMS showed that a significant amount of dtbbpy ligand was still present. The sample was dissolved in Et_2O (10 mL) and was washed with 20% $CuSO_4$ aqueous solution (2 x 20 mL). The combined organics were then dried through a hydrophobic frit and evaporated *in vacuo* give a colourless oil. LCMS showed that there was an unknown impurity present. The sample was dissolved in DCM and purified by normal phase chromatography (0-10% EtOAc in cyclohexane) using a 12 g silica cartridge over 14 CV. The desired fractions were combined and evaporated *in vacuo* to afford **2v** (28 mg, 29%) as a colourless oil. **^{1}H NMR (400 MHz, $CDCl_3$):** δ 7.24 (dd, *J* = 8.4, 2.0 Hz, 1H), 7.11 (d, *J* = 2.0 Hz, 1H), 6.93 (d, *J* = 8.4 Hz, 1H), 4.50 (tt, *J* = 6.9, 3.6 Hz, 1H), 3.88 (s, 3H), 3.24-3.18 (m, 2H), 2.92-2.86 (m, 2H), 2.06-1.99 (m, 2H), 1.97-1.89 (m, 2H); **^{13}C NMR (101 MHz, $CDCl_3$):** δ 150.7, 150.6, 126.1, 124.5 (q, *J* = 256.0 Hz), 119.0, 115.7, 115.1, 104.8, 73.1, 56.2, 41.2 (q, *J* = 2.9 Hz), 29.5; **^{19}F NMR (376 MHz, $CDCl_3$):** δ -67.12 (s, 3F); **LCMS (System A):** t_R = 1.20 min, m/z = 301 (MH+); **HRMS (ESI$^+$):** $C_{14}H_{15}F_2N_2O_2$ $[M-F]^+$ requires 281.1096 found 281.1089; **IR (cm^{-1}):** 3085 (w, C-H), 2929 (w, C-H), 2223 (m, C≡N), 1596.

The reaction was repeated using Purification A to afford the desired product (**2v**) (32 mg, 33%) as a colourless oil, with data matching that recorded, above.

1,1,1-Trifluoro-*N*-(5-fluoro-2-phenoxybenzyl)-*N*-methylmethanamine (2w)

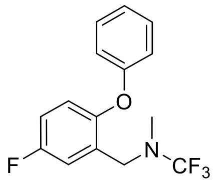

Compound synthesised following general procedure 4.1.

Amount of *O*-benzoylhydroxylamine used:

O-Benzoyl-*N*-(5-fluoro-2-phenoxybenzyl)-*N*-methylhydroxylamine (**1w**) (113 mg, 0.322 mmol, 1 eq.)

Purification A; The product mixture was dissolved in DCM and purified by normal phase chromatography (0-15% EtOAc in cyclohexane) using a 12 g silica cartridge over 14 CV. The desired fractions were combined and evaporated *in vacuo* to afford **2w** (8 mg, 8%) as a colourless oil. Due to rapid decomposition of this product, ^{13}C NMR and HRMS could not be obtained. Therefore, the product structure proposed has been tentatively assigned. **^{1}H NMR (400 MHz, $CDCl_3$):** δ 7.35-7.30 (m, 2H), 7.23 (dd, *J* = 9.1 Hz, 1H,), 7.11-7.07 (m, 1H), 6.99-6.94 (m, 1H), 6.92-6.89 (m, 3H,), 4.00 (s, 2H), 2.55-2.53 (m, 3H); **^{19}F {^{1}H} NMR (376 MHz, $CDCl_3$):** δ -65.47 (s, 3F), -118.44 (s, 1F); **LCMS (System B):** t_R = 1.43 min, m/z = No m/z in keeping with structure of desired product observed.

Scale up reaction:

Gram Scale Synthesis of Phenyl(4-(trifluoromethyl)piperazin-1-yl)methanone (2a)

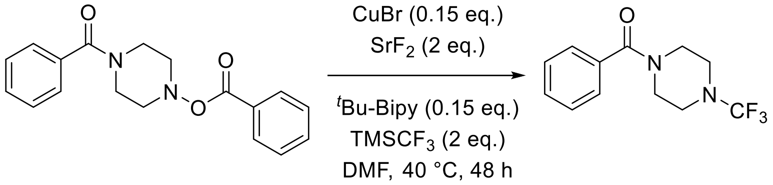

4-Benzoylpiperazin-1-yl benzoate (**1a**) (1 g, 3.22 mmol, 1 eq.), copper (I) bromide (70 mg, 0.49 mmol, 0.15 eq.), 4,4'-di-*tert*-butyl-2,2'-bipyridine (130 mg, 0.483 mmol, 0.15 eq.) and strontium fluoride (810 mg, 6.44 mmol, 2 eq.) were added to a microwave vial and stirred in DMF (30 mL) at room temperature under a nitrogen atmosphere. To the reaction mixture was added trimethyl(trifluoromethyl)silane (953 µL, 6.44 mmol, 2 eq.) dropwise and the reaction mixture was stirred at 40 °C for 48 h. The reaction mixture was diluted with EtOAc (100 mL) and washed with 1 M Na_2CO_3 aqueous solution (2 x 200 mL). The combined organics were then dried through a hydrophobic frit, absorbed onto celite and purified by normal phase chromatography (0-100% EtOAc in cyclohexane) using a 40 g silica cartridge over 14 CV. The desired fractions were combined and evaporated *in vacuo* to afford **2a** (384 mg, 46%) as a white solid.

^{1}H NMR and LCMS data for **2a** were consistent with that recorded for the same compound, above.

5. Side Products and Unsuccessful Substrates

The following side products and unsuccessful substrates were synthesised/were attempted to be synthesised from their corresponding *O*-benzoylhydroxylamines, according to general procedure 4.1.

Secondary N-trifluoromethylamines:

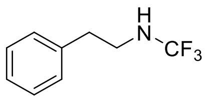

4a, 0%

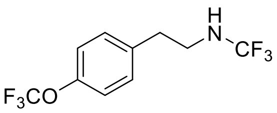

4b, 0%

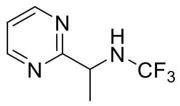

4c, 0%

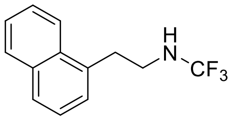

4d, 0%

Synthesis of each of the above substrates was unsuccessful, with no indication that the desired *N*-trifluoromethylation had taken place. Whilst it appeared that the starting material had reacted in these reactions, no side products could be isolated.

Tertiary N-trifluoromethylamines:

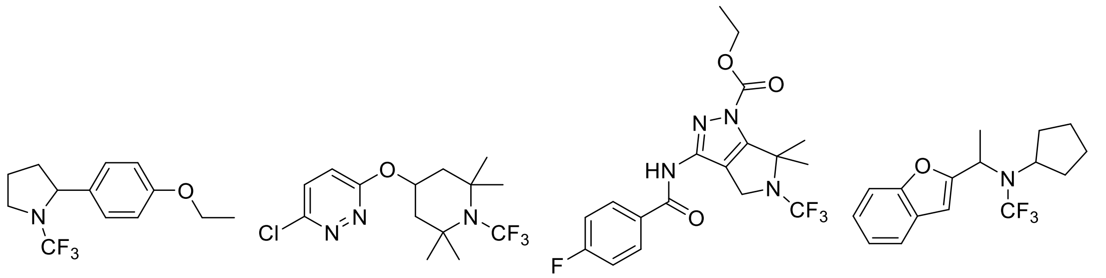

S4a, 0% **S4b**, 0% **S4c**, 0% **S4d**, 0%

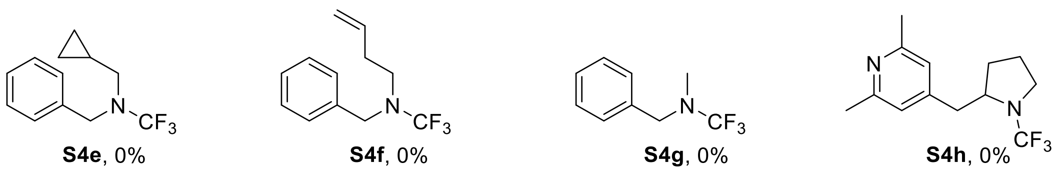

S4e, 0% **S4f**, 0% **S4g**, 0% **S4h**, 0%

In general, benzylic amine substrates were less successful, with a greater tendency to form imine side products (see below). Sterically hindered substrates (**S4b**, **S4c**), as well as 5-membered ring substrates were also unsuccessful.

Isolated side products:

***Tert*-butyl 3*H*-spiro[isoquinoline-4,4'-piperidine]-1'-carboxylate (3a)**

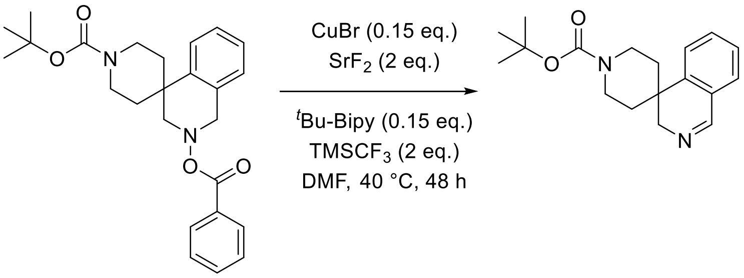

Compound synthesised following general procedure 4.1.

Amount of *O*-benzoylhydroxylamine used:

Tert-butyl 2-(benzoyloxy)-2,3-dihydro-1*H*-spiro[isoquinoline-4,4'-piperidine]-1'-carboxylate (**1i**) (136 mg, 0.322 mmol, 1 eq.)

Purification A; The product mixture was dissolved in DCM and purified by normal phase chromatography (0-50% EtOAc in cyclohexane) using a 12 g silica cartridge over 14 CV. The desired fractions were combined and evaporated *in vacuo* to give a colourless oil. LCMS

suggested that there was an unknown impurity present. The product mixture was purified by MDAP (Method A) and the desired fractions were combined and evaporated *in vacuo* to afford **3a** (13 mg, 13%) as a yellow oil. **^{1}H NMR (600 MHz, DMSO-*d*$_6$):** δ 8.80-8.20 (br m, 1H), 7.50-7.46 (m, 2H), 7.42 (br d, *J* = 7.3 Hz, 1H), 7.38-7.34 (m, 1H), 3.94-3.65 (br m, 4H), 3.22-2.97 (br m, 2H), 1.68 (td, *J* = 13.0, 4.4 Hz, 2H), 1.55 (br d, *J* = 13.0 Hz, 2H), 1.41 (s, 9H); **^{13}C NMR (151 MHz, DMSO-*d*$_6$):** δ 160.4, 153.9, 143.8, 131.8, 127.6, 127.0, 126.9, 123.1, 78.6, 52.3, 38.8 (br), 32.3, 32.0, 28.1; **LCMS (System B):** t_R = 1.07 min, m/z = 301 (MH+); **HRMS (ESI$^+$):** $C_{18}H_{25}N_2O_2$ $[M+H]^+$ requires 301.1911 found 301.1924; **IR (cm^{-1}):** 2973 (w, C-H), 2932 (w, C-H), 1683 (s, C=O), 1633 (m, C=N).

For the isolation of **2i** within the same reaction, see page 64.

5-(4-Ethoxyphenyl)-3,4-dihydro-2*H*-pyrrole (3b)

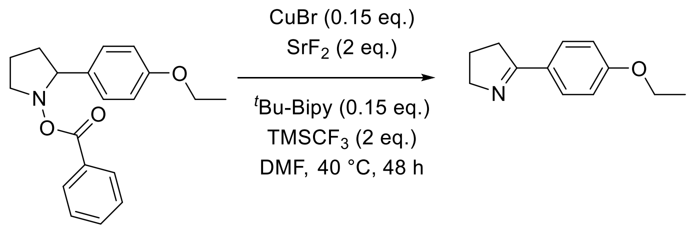

Compound synthesised following general procedure 4.1.

Amount of *O*-benzoylhydroxylamine used:

2-(4-Ethoxyphenyl)pyrrolidin-1-yl benzoate (**1x**) (100 mg, 0.322 mmol, 1 eq.)

Purification B; The product mixture was preabsorbed onto celite and purified by normal phase chromatography (0-100% EtOAc in cyclohexane) using a 12 g silica cartridge over 14 CV. The desired fractions were combined and evaporated *in vacuo* to give a brown solid. NMR and LCMS showed that unknown impurities were present. The product mixture was purified by MDAP (Method A) and the desired fractions were combined and evaporated *in vacuo* to afford **3b** (11 mg, 18%) as a white solid. **^{1}H NMR (400 MHz, DMSO-*d*$_6$):** δ 7.77-7.73 (m, 2H), 6.97-6.94 (m, 2H), 4.06 (q, *J* = 7.0 Hz, 2H), 3.91-3.87 (m, 2H), 2.88-2.83 (m, 2H), 1.95-1.87 (m, 2H), 1.33 (t, *J* = 7.0 Hz, 3H); **^{13}C NMR (101 MHz, DMSO-*d*$_6$):** δ 171.3, 160.0, 129.0, 127.0, 114.1, 63.1, 60.6, 34.4, 22.3, 14.5; **LCMS (System B):** t_R = 1.00 min, m/z = 190; **HRMS (ESI$^+$):** $C_{12}H_{16}NO$ $[M+H]^+$ requires 190.1226 found 190.1233; **IR (cm^{-1}):** 2978 (w, C-H), 2926 (w, C-H), 1602 (s, C=N), 1247.

The reaction was repeated using Purification B to afford the desired product (**2.36**) (16 mg, 26%) as a white solid, with data matching that recorded, above.

3-Chloro-6-((2,2,6,6-tetramethylpiperidin-4-yl)oxy)pyridazine (S5a)

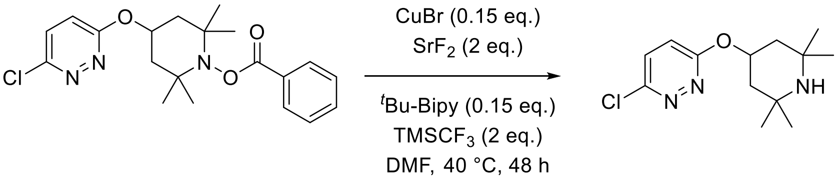

Compound synthesised following general procedure 4.1.

Amount of *O*-benzoylhydroxylamine used:

4-((6-Chloropyridazin-3-yl)oxy)-2,2,6,6-tetramethylpiperidin-1-yl benzoate (**S1d**) (126 mg, 0.322 mmol, 1 eq.)

Purification A; The product mixture was purified by MDAP (Method A) and the desired fractions were combined and evaporated *in vacuo* to afford **S5a** (54 mg, 62%) as a white solid. **^{1}H NMR (400 MHz, DMSO-d_6):** δ 7.76 (d, *J* = 9.1 Hz, 1H), 7.26 (d, *J* = 9.1 Hz, 1H), 5.57 (tt, *J* = 11.3, 4.1 Hz, 1H), 2.03 (dd, *J* = 12.1, 4.1 Hz, 2H), 1.35 (br s, 1H), 1.26-1.23 (m, 2H), 1.19 (s, 6H), 1.09 (s, 6H); **^{13}C NMR (101 MHz, DMSO-d_6):** δ 163.9, 150.1, 131.6, 121.2, 71.6, 50.9, 43.0, 34.3, 29.1; **LCMS (System B):** t_R = 0.92 min, m/z = 270 (MH+); **HRMS (ESI$^+$):** $C_{13}H_{21}ClN_3O$ $[M+H]^+$ requires 270.1368 found 270.1377; **IR (cm^{-1}):** 2969 (br w, N-H), 2954 (w, C-H), 1629, 1417; **M.pt (MeCN/H_2O/$(NH_4)_2CO_3$):** 67-71 °C.

N-Trifluoromethyl amides/sulfonamides

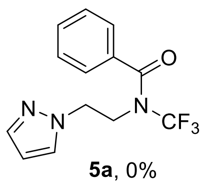

5a, 0%

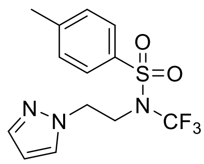

5b, 0%

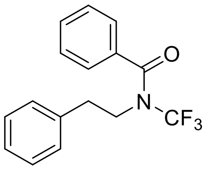

5c, 0%

The above amides and sulfonamides could not be synthesised using the developed protocol. It appeared that the starting materials within each of these processes did not react under the optimised reaction conditions.

Carbamoyl Fluoride Formation:

3-((1-Oxoisoquinolin-2(1*H*)-yl)methyl)piperidine-1-carbonyl fluoride (6a)

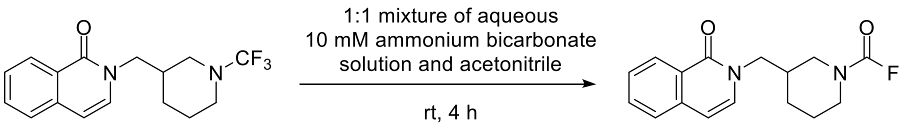

2f **6a**

2-((1-(Trifluoromethyl)piperidin-3-yl)methyl)isoquinolin-1(2*H*)-one (**2f**) (10 mg, 32 µmol, 1 eq.) was dissolved in a 1:1 mixture of ammonium bicarbonate in water (adjusted to pH 10 with ammonia solution; 0.8 mg, 1 mL, 10 mM, 0.01 mmol, 0.3 eq.) and acetonitrile (1 mL) and was stirred at room temperature for 4 h. The reaction mixture was evaporated under a flow of nitrogen to give the crude product as a white solid. The crude product was dissolved in DCM and washed with water (2 x 1 mL). The organic layer was dried through a hydrophobic frit and evaporated under a flow of nitrogen to afford **6a** (2.5 mg, 27%) as a white solid. **^{1}H NMR (400 MHz, DMSO-d_6):** δ 8.22 (d, *J* = 7.9 Hz, 1H), 7.73-7.69 (m, 1H), 7.66 (d, *J* = 7.9 Hz, 1H), 7.53-7.49 (m, 1H), 7.46-7.44 (m, 1H), 6.64 (d, *J* = 6.9 Hz, 1H), 3.96-3.86 (m, 2H), 3.69-3.60 (br m, 2H), 3.07-2.99 (br m, 1H), 2.96-2.85 (br m, 1H), 2.12-2.02 (m, 1H), 1.76-1.68 (br m, 2H), 1.48-1.36 (m, 1H), 1.33-1.23 (m, 1H); **^{13}C NMR (151 MHz, DMSO-d_6)** (rotameric signals observed): δ 161.17 (rot. A), 161.15 (rot. B), 145.6 (rot. A, d, *J* = 283.6 Hz), 145.4 (rot. B, d, *J* = 283.6 Hz), 136.9 (rot. A), 136.8 (rot. B), 133.2 (rot. A), 133.1 (rot. B), 132.3, 127.03 (rot. A), 127.02 (rot. B), 126.7, 126.1, 125.29 (rot. A), 125.28 (rot. B), 104.98 (rot. A), 104.95 (rot. B), 50.5 (rot. A), 50.2 (rot. B), 47.9 (rot. A), 47.6 (rot. B, d, *J* = 3.9 Hz), 45.2 (rot. A), 44.9 (rot. B, d, *J* = 3.9 Hz), 35.6 (rot. A), 35.3 (rot. B), 27.0 (rot. A), 26.9 (rot. B), 23.9 (rot. A), 23.3

(rot. B); **^{19}F NMR (376 MHz, DMSO-*d_6*)** (rotameric signals observed): δ -24.17 (rot. A), -24.39 (rot. B); **LCMS (System B):** t_R = 0.96 min, m/z = 289; **HRMS (ESI$^+$):** $C_{16}H_{18}FN_2O_2$ [M+H]$^+$ requires 289.1347 found 289.1365; **IR (cm^{-1}):** 2937 (w, C-H), 1776 (s, C=O), 1648 (s, C=O), 1623.

6. Mechanistic Control Reactions

Imine formation control reactions:

Reaction of 2-(4-ethoxyphenyl)pyrrolidin-1-yl benzoate (1x) in DMF

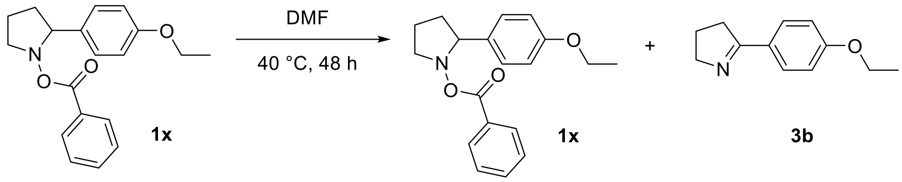

2-(4-Ethoxyphenyl)pyrrolidin-1-yl benzoate (**1x**) (100 mg, 322 µmol, 1 eq.) was stirred in DMF (3 mL) at 40 °C for 48 h under a nitrogen atmosphere. The reaction mixture was blown-down under a flow of nitrogen to give the crude product as a yellow oil. The crude product was dissolved in DMSO (1 mL) and purified by MDAP (Method A) and the desired fractions were combined and evaporated *in vacuo* to afford **1x** (79 mg, 79%) as a colourless oil and **3b** (5 mg, 8%) as a white solid.

^{1}H NMR and LCMS data for both **1x** and **3b** were consistent with that recorded for the same compounds, above.

Reaction of 2-(4-ethoxyphenyl)pyrrolidin-1-yl benzoate (1x) in DMF and CuBr

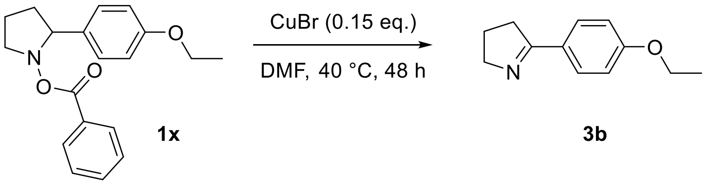

2-(4-Ethoxyphenyl)pyrrolidin-1-yl benzoate (**1x**) (100 mg, 322 µmol, 1 eq.) and copper (I) bromide (7 mg, 49 µmol, 0.15 eq.) were stirred in DMF (3 mL) at 40 °C for 48 h under a nitrogen atmosphere. The reaction mixture was blown-down under a flow of nitrogen to give the crude product as a brown oil. The crude product was dissolved in DMSO (1 mL) and purified by MDAP (Method A) and the desired fractions were combined and evaporated *in vacuo* to afford **3b** (17 mg, 28%) as a white solid.

^{1}H NMR and LCMS data for **3b** were consistent with that recorded for the same compound, above.

Reaction of *tert*-butyl 2-(benzoyloxy)-2,3-dihydro-1*H*-spiro[isoquinoline-4,4'-piperidine]-1'-carboxylate (1i) in DMF

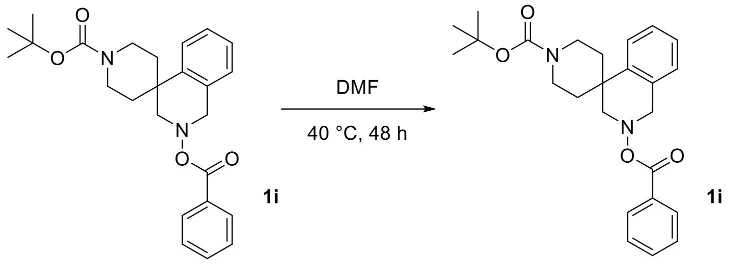

Tert-butyl 2-(benzoyloxy)-2,3-dihydro-1*H*-spiro[isoquinoline-4,4'-piperidine]-1'-carboxylate (**1l**) (136 mg, 0.322 mmol, 1 eq.) was stirred in DMF (3 mL) at 40 °C for 48 h under a nitrogen atmosphere. The reaction mixture was blown-down under a flow of nitrogen to give the crude product as a colourless oil. The crude product was dissolved in DCM and purified by normal phase chromatography (0-50% EtOAc in cyclohexane) using a 12 g silica cartridge over 14 CV. The desired fractions were combined and evaporated *in vacuo* to afford **1i** (118 mg, 87%) as a white solid.

^{1}H NMR and LCMS data for **1i** matched the structure of **1i** previously reported.

Reaction of *tert*-butyl 2-(benzoyloxy)-2,3-dihydro-1*H*-spiro[isoquinoline-4,4'-piperidine]-1'-carboxylate (1i) in DMF and CuBr

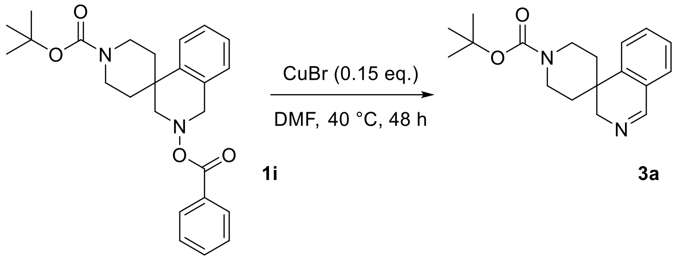

Tert-butyl 2-(benzoyloxy)-2,3-dihydro-1*H*-spiro[isoquinoline-4,4'-piperidine]-1'-carboxylate (136 mg, 0.322 mmol, 1 eq.) (**1i**) and copper (I) bromide (7 mg, 49 µmol, 0.15 eq.) were stirred in DMF (3 mL) at 40 °C for 48 h under a nitrogen atmosphere. The reaction mixture was blown-down under a flow of nitrogen to give the crude product as a green oil. The crude product was dissolved in DMSO (1 mL) and purified by MDAP (Method A) and the desired fractions were combined and evaporated *in vacuo* to afford **3a** (72 mg, 74%) as a yellow oil.

^1H NMR and LCMS data for **3a** were consistent with that recorded for the same compound, above.

The reaction was repeated to afford **3a** (72 mg, 74%) as a yellow oil, with data matching that recorded, above.

Core mechanism control reactions:

Removing copper/ligand

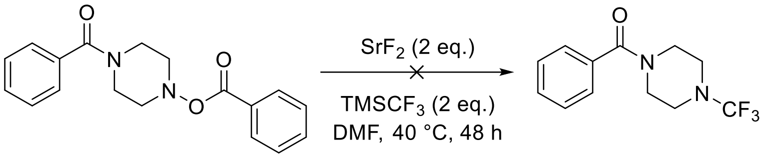

4-Benzoylpiperazin-1-yl benzoate (**1a**) (100 mg, 0.322 mmol, 1 eq.) and strontium fluoride (81 mg, 0.64 mmol, 2 eq.) were added to a microwave vial and stirred in DMF (3 mL) at room temperature under a nitrogen atmosphere. To the reaction mixture was added trimethyl(trifluoromethyl)silane (95 µL, 0.64 mmol, 2 eq.) dropwise and the reaction mixture was stirred at 40 °C for 48 h. An LCMS of the reaction mixture showed that the starting material had not reacted.

Removing TMSCF$_3$

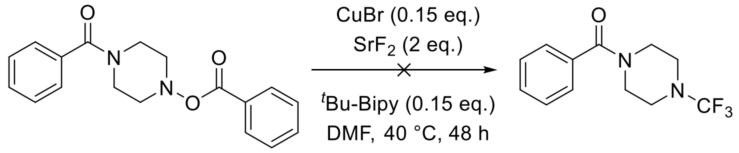

4-Benzoylpiperazin-1-yl benzoate (**1a**) (100 mg, 0.322 mmol, 1 eq.), copper (I) bromide (7 mg, 0.049 mmol, 0.15 eq.), 4,4'-di-*tert*-butyl-2,2'-bipyridine (13 mg, 0.048 mmol, 0.15 eq.) and strontium fluoride (81 mg, 0.64 mmol, 2 eq.) were added to a microwave vial and stirred in DMF (3 mL) at 40 °C for 48 h under a nitrogen atmosphere. An LCMS of the reaction mixture showed that some of the starting material had reacted but the desired product (**2a**) was not formed.

Removing SrF_2

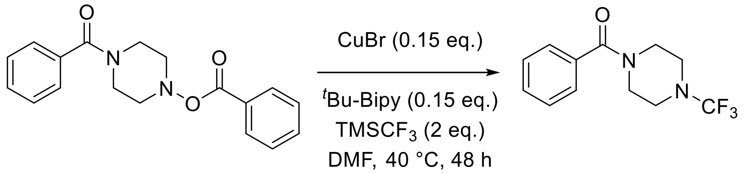

4-Benzoylpiperazin-1-yl benzoate (**1a**) (0.322 mmol, 1 eq.), copper (I) bromide (7 mg, 0.049 mmol, 0.15 eq.) and 4,4'-di-*tert*-butyl-2,2'-bipyridine (13 mg, 0.048 mmol, 0.15 eq.) were added to a microwave vial and stirred in DMF (3 mL) at room temperature under a nitrogen atmosphere. To the reaction mixture was added trimethyl(trifluoromethyl)silane (95 µL, 0.64 mmol, 2 eq.) dropwise and the reaction mixture was stirred at 40 °C for 48 h. The reaction mixture was diluted with EtOAc (10 mL) and washed with 1 M NaOH aqueous solution (2 x 20 mL). The combined organics were then dried through a hydrophobic frit and evaporated *in vacuo* to give the crude product as an orange oil. The crude product was dissolved in DCM and purified by normal phase chromatography (0-100% EtOAc in cyclohexane) using a 12 g silica cartridge over 14 CV. The desired fractions were combined and evaporated *in vacuo* to afford **2a** (43 mg, 52%) as a white solid.

^1H NMR and LCMS data for **2a** were consistent with that recorded for the same compound, above.

7. Mechanistic Proposal

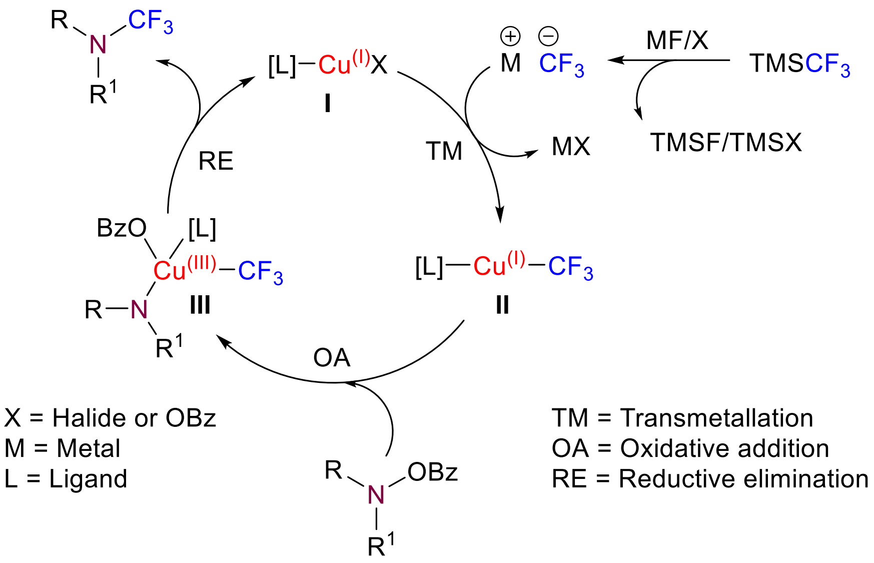

8. References

[1] A. Banerjee, H. Yamamoto, *Chem. Sci.* **2019**, *10*, 2124-2129.

[2] a) R. Wodtke, J. Steinberg, M. Köckerling, R. Löser, C. Mamat, *RSC Adv.* **2018**, *8*, 40921-40933; b) C. Mamat, M. Pretze, M. Gott, M. Köckerling, *Beilstein J. Org. Chem.* **2016**, *12*, 2478-2489.

[3] S. Schaub, J. Becker, S. Schindler, *ChemistrySelect* **2022**, *7*, e202201803.

[4] T. Scattolin, K. Deckers, F. Schoenebeck, *Angew. Chem. Int. Ed.* **2017**, *56*, 221-224.

9. NMR Spectra of *O*-Benzoylhydroxylamines

^{1}H NMR (400 MHz, DMSO-d_6) of Compound **1a**

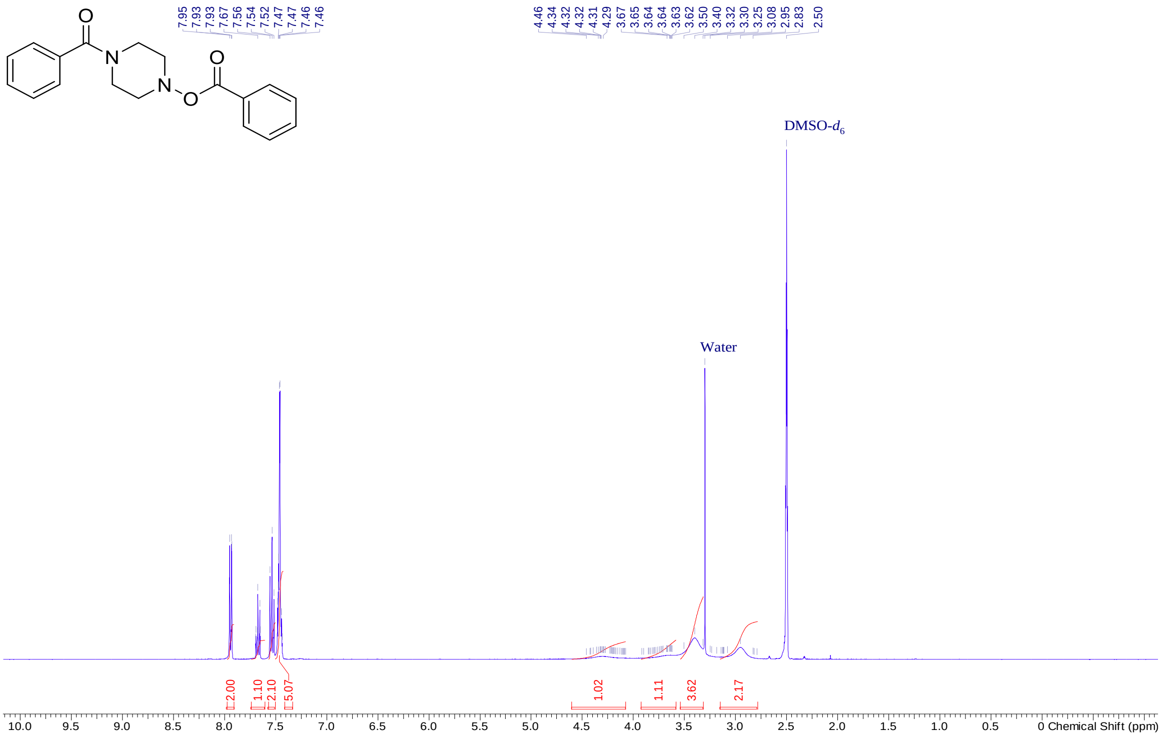

^{13}C NMR (151 MHz, DMSO-d_6) of Compound **1a**

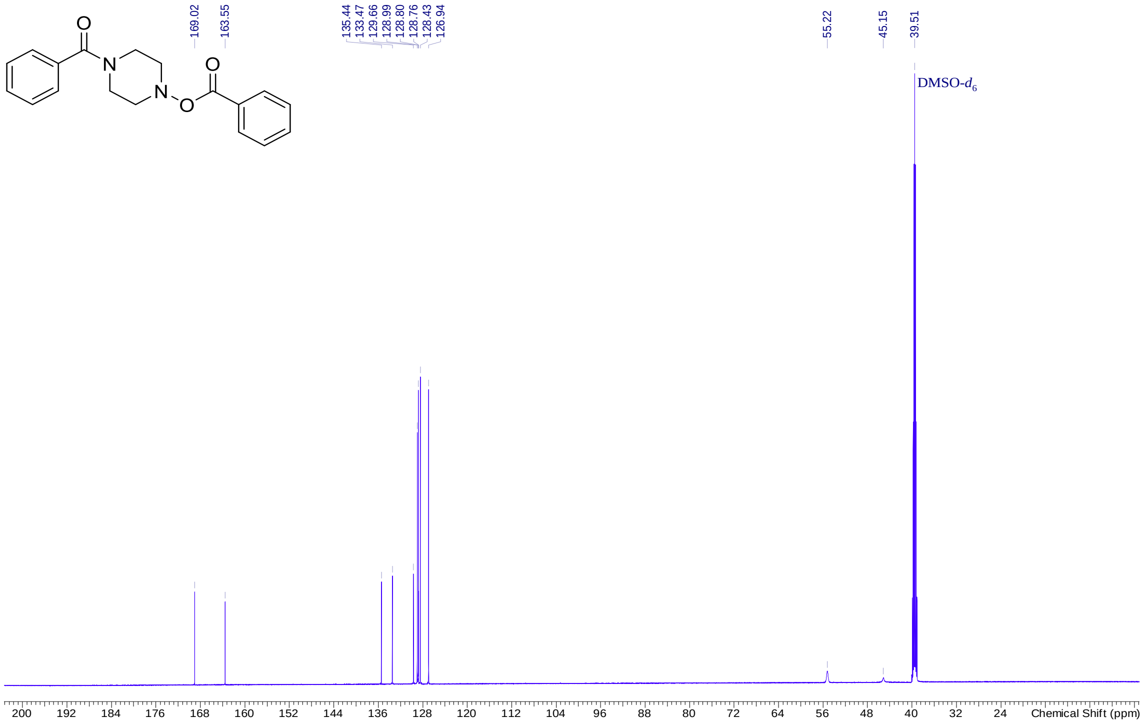

^{1}H NMR (600 MHz, DMSO-d_6) of Compound **1b**

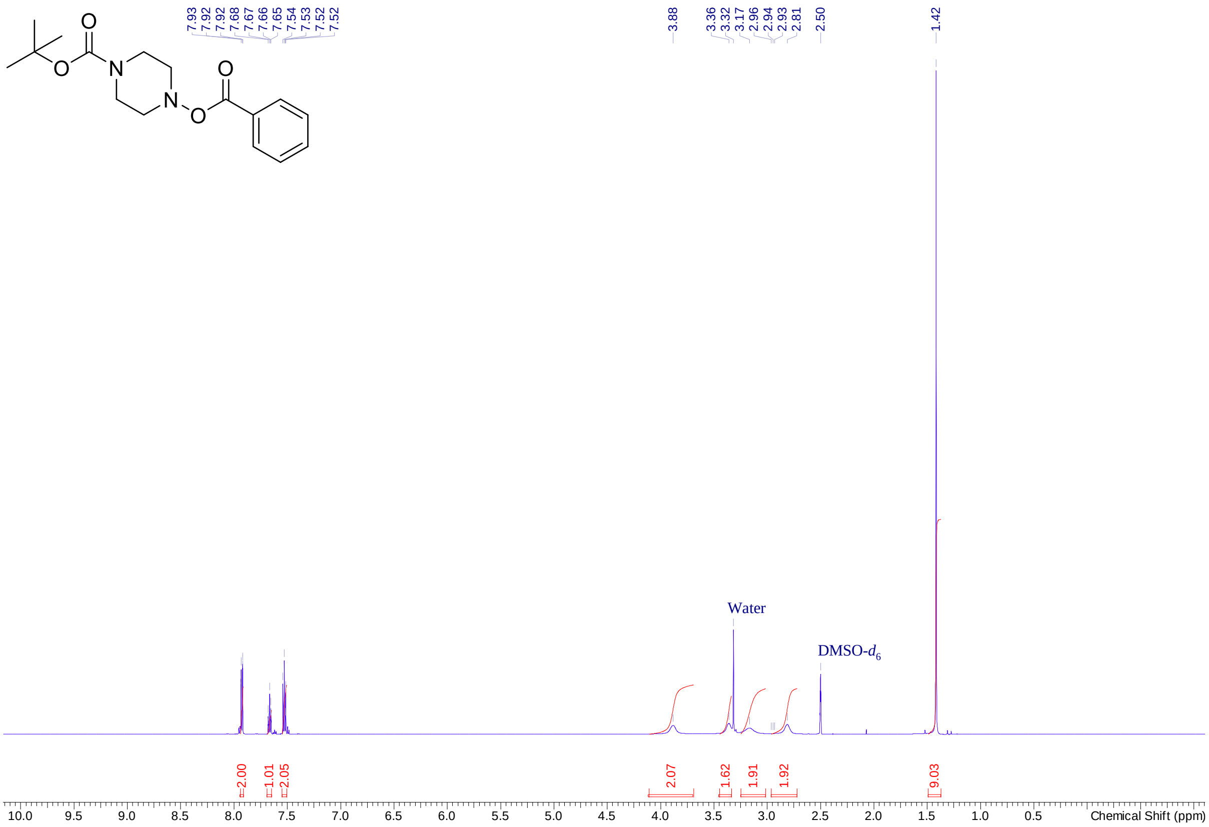

^{13}C NMR (151 MHz, DMSO-d_6) of Compound **1b**

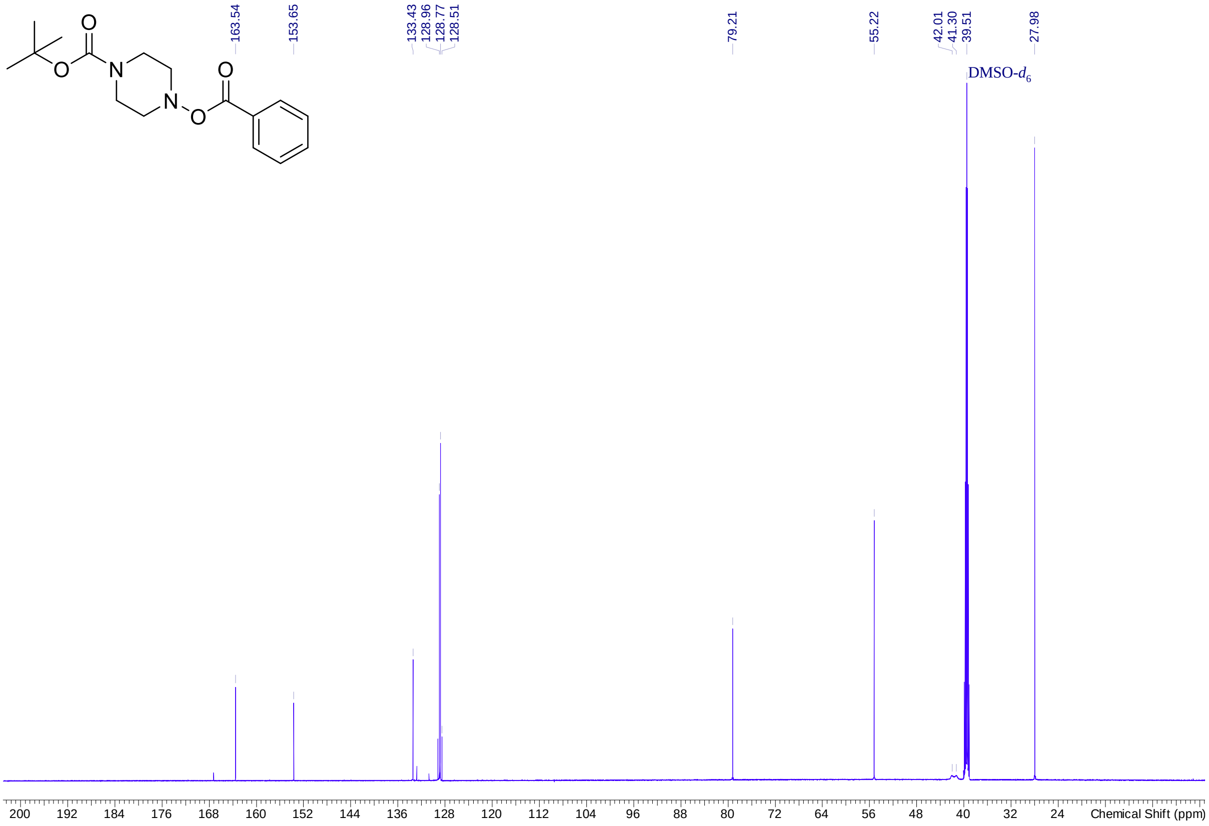

^{1}H NMR (600 MHz, DMSO-d_6) of Compound **1c**

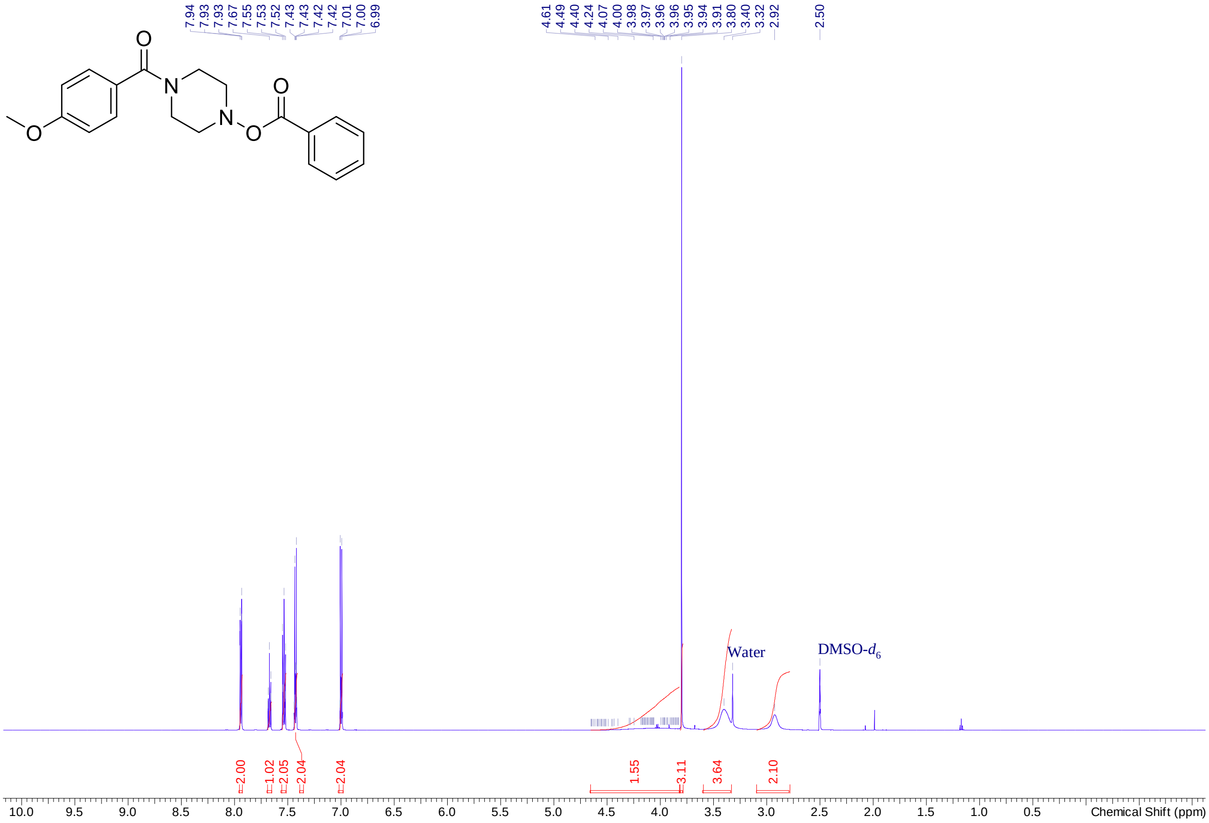

^{13}C NMR (151 MHz, DMSO-d_6) of Compound **1c**

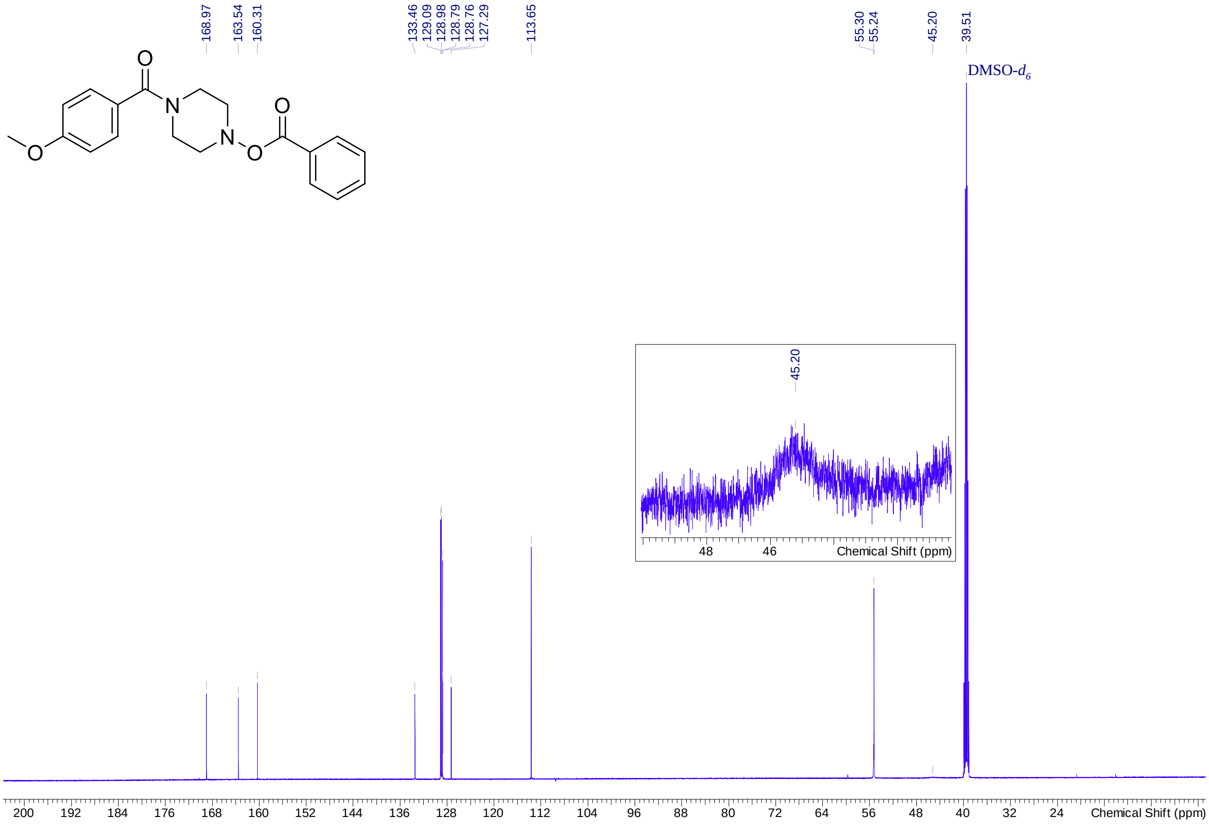

^{1}H NMR (600 MHz, DMSO-d_6) of Compound **1d**

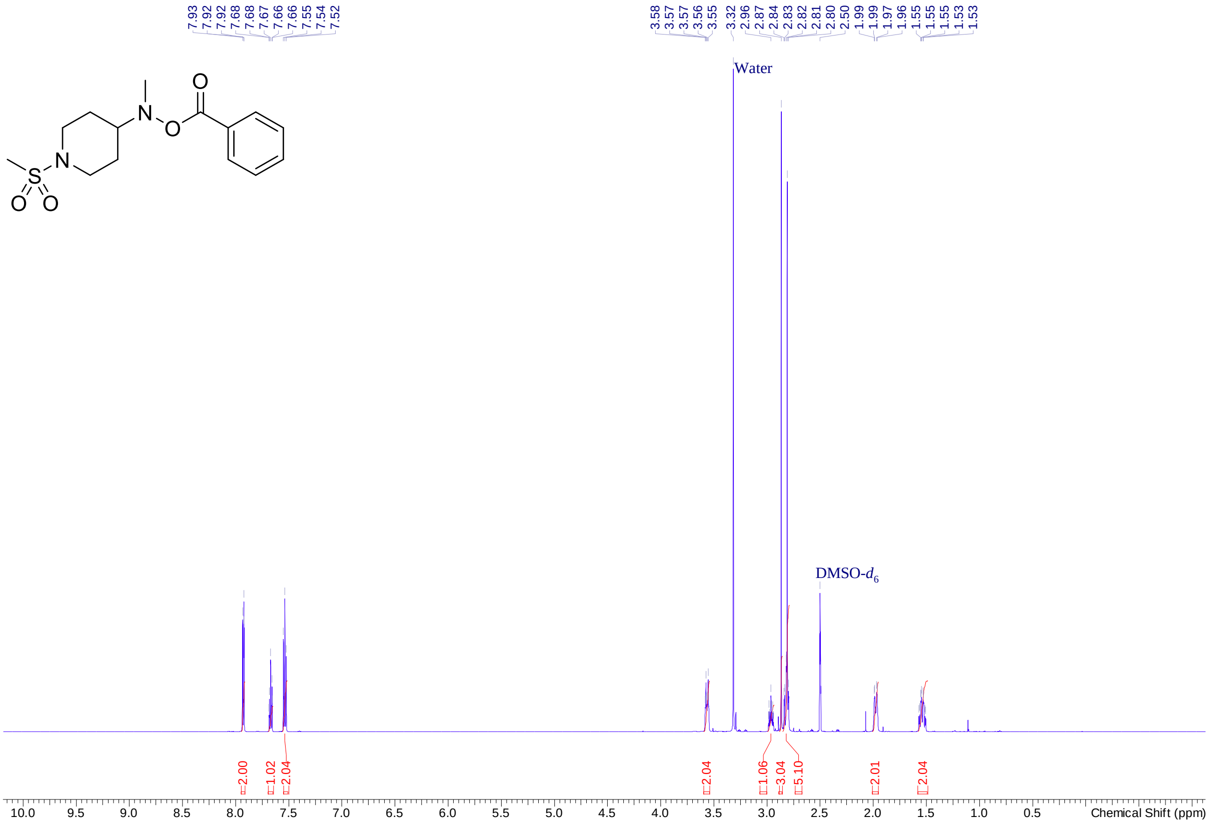

^{13}C NMR (151 MHz, DMSO-d_6) of Compound **1d**

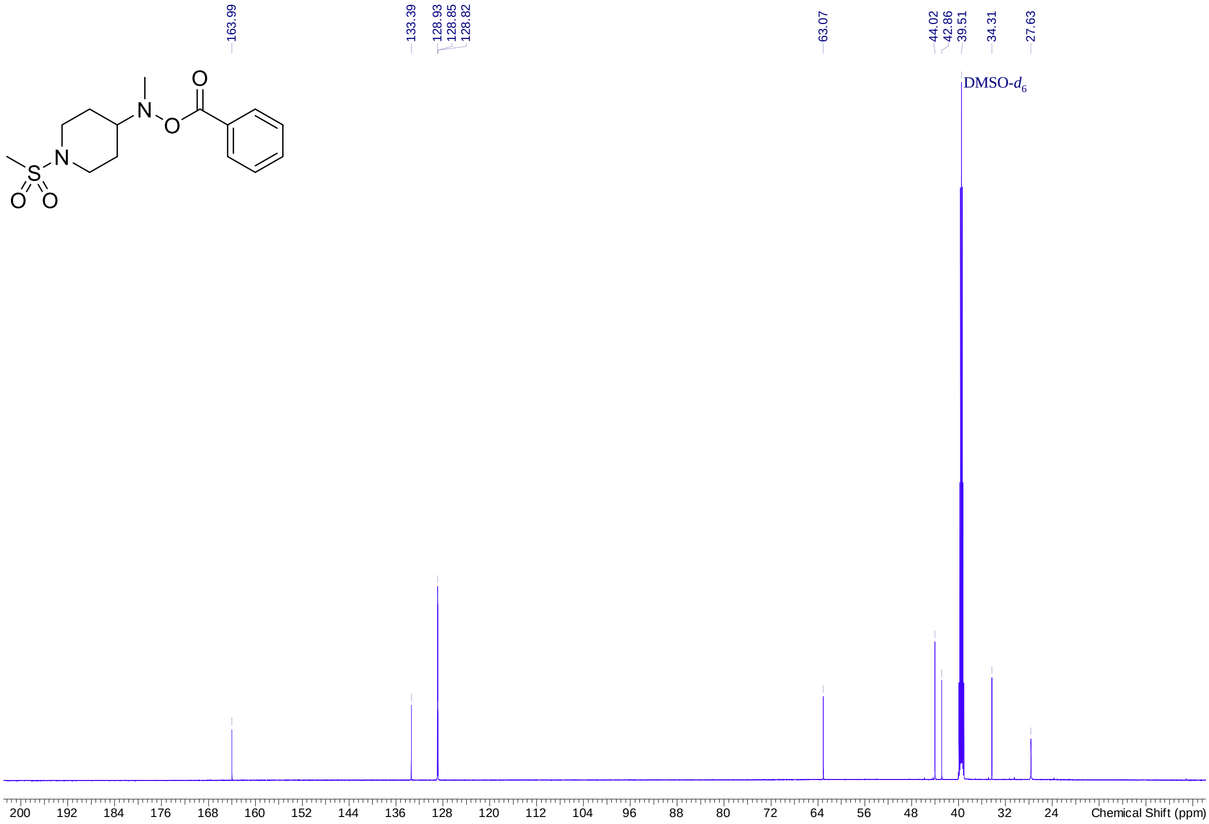

^{1}H NMR (600 MHz, DMSO-d_6) of Compound **1e**

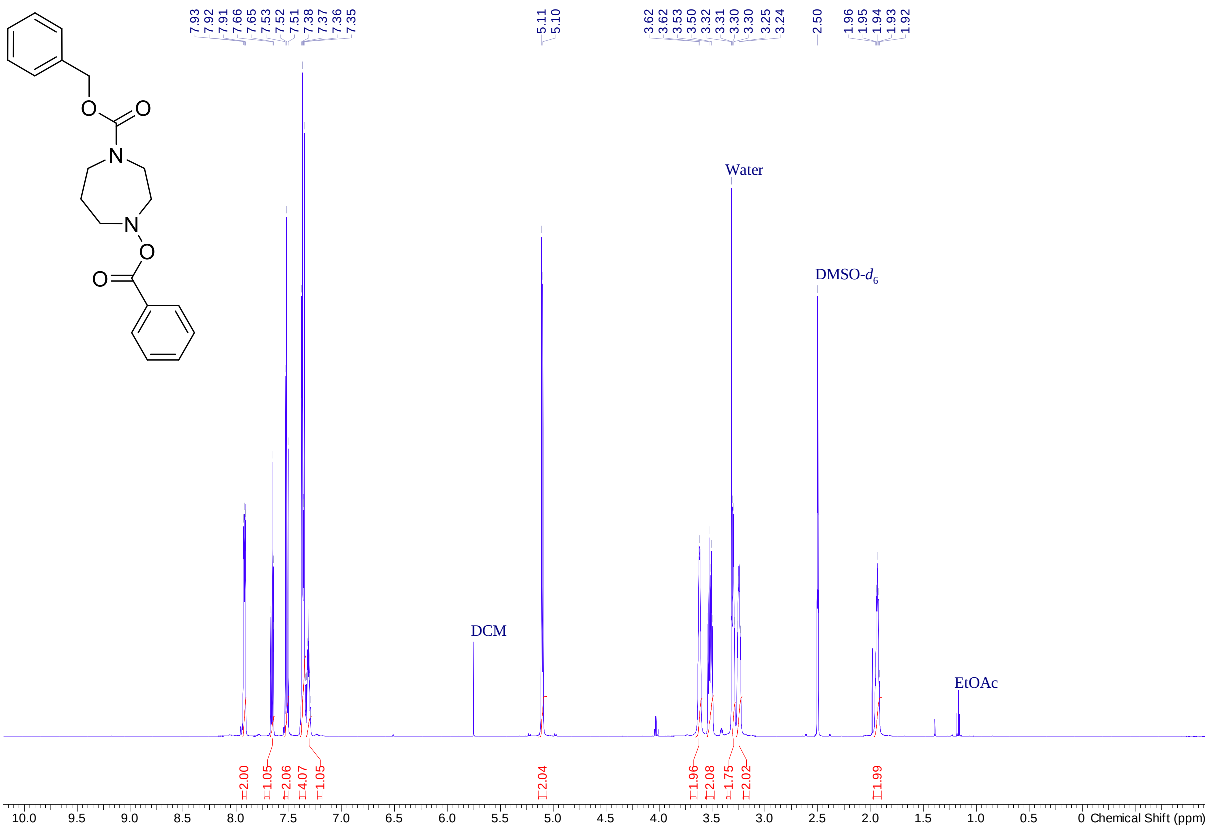

^{13}C NMR (151 MHz, DMSO-d_6) of Compound **1e**

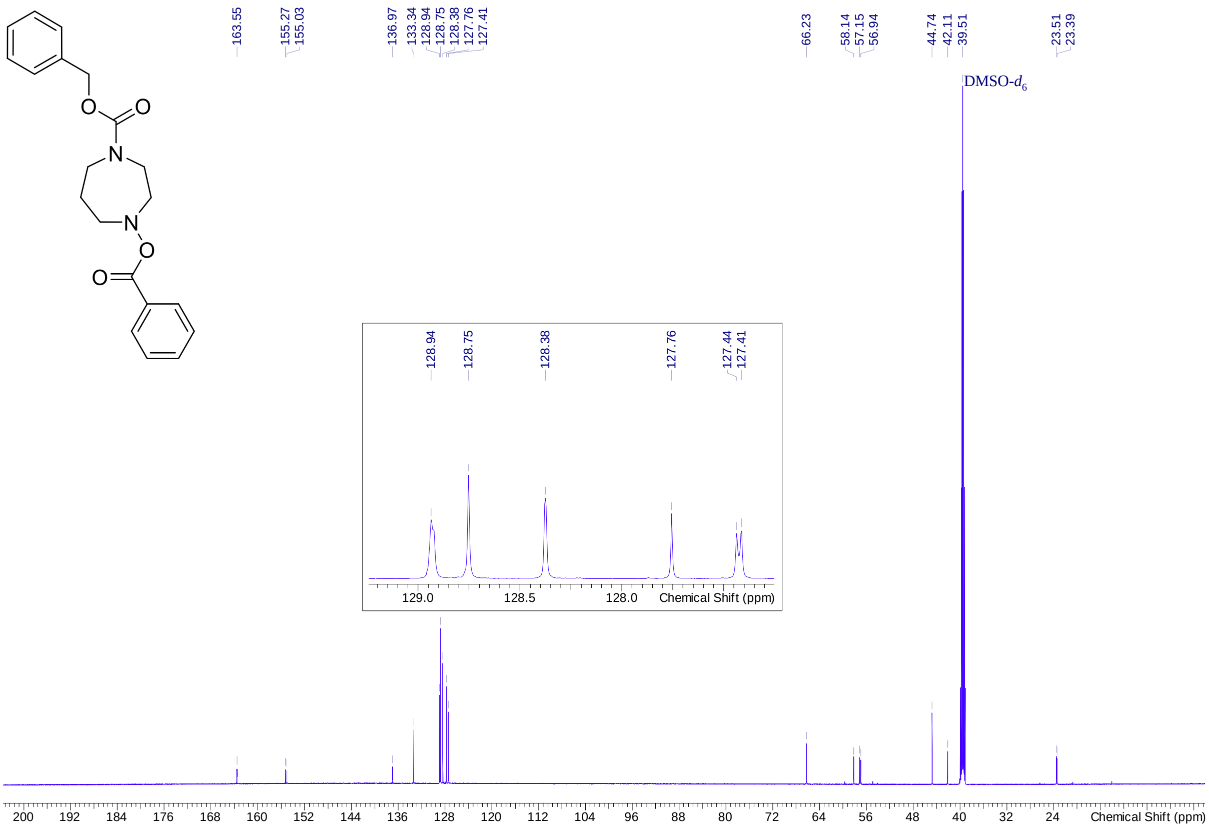

^{1}H NMR (600 MHz, DMSO-d_6) of Compound **1f**

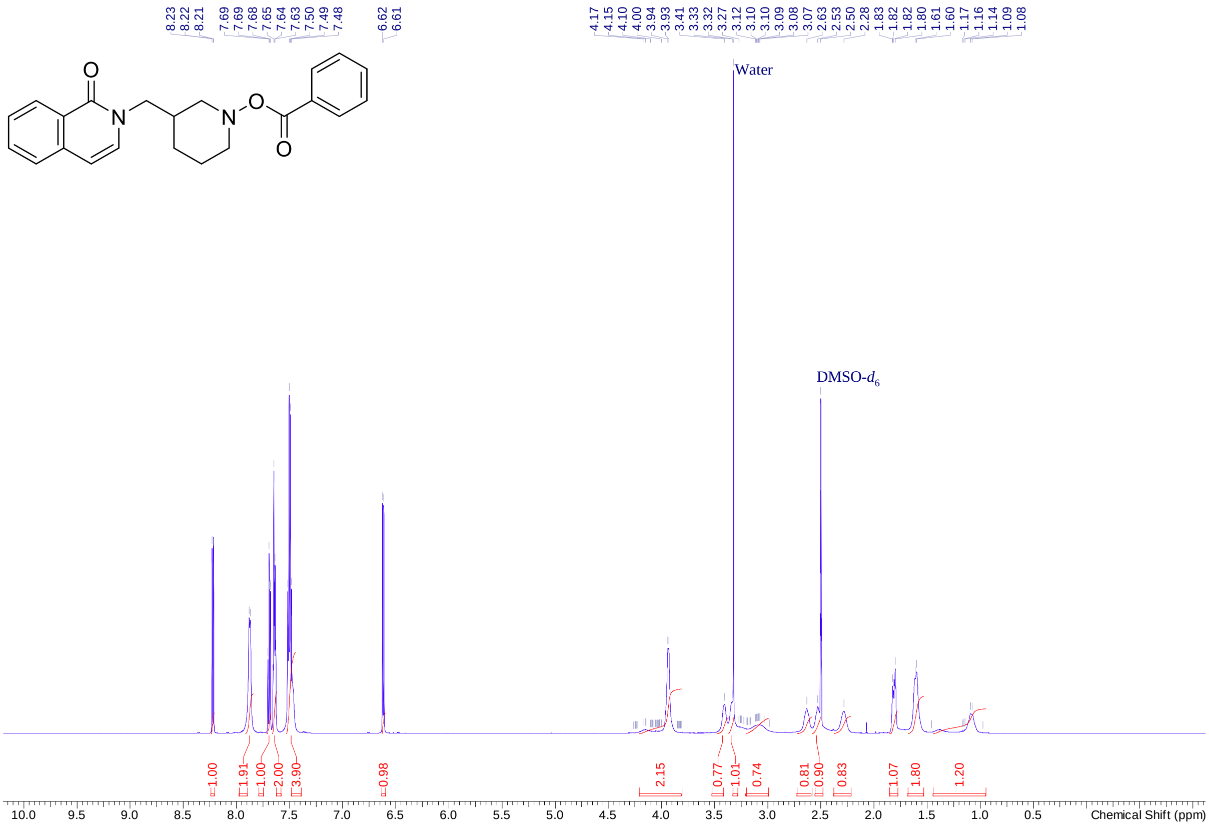

^{13}C NMR (151 MHz, DMSO-d_6) of Compound **1f**

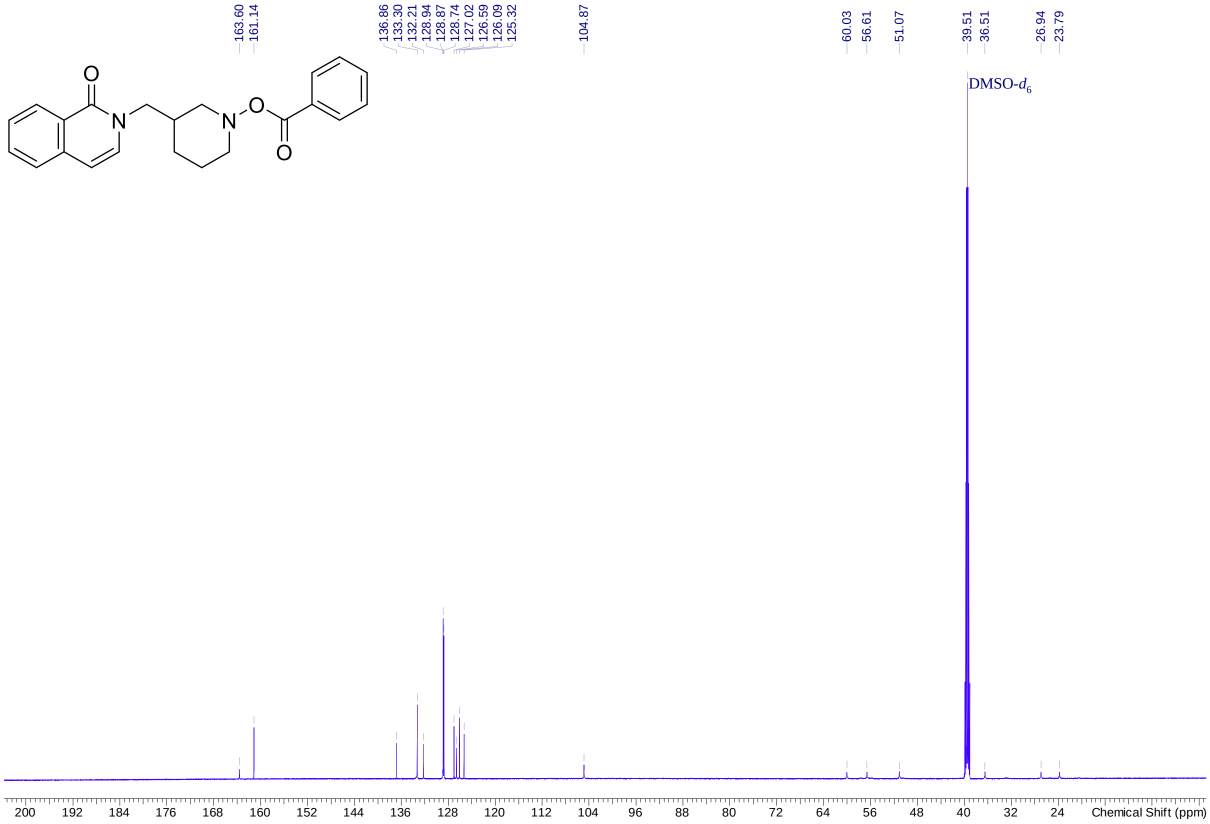

^{1}H NMR (500 MHz, 380 K, DMSO-d_6) of Compound **1g**

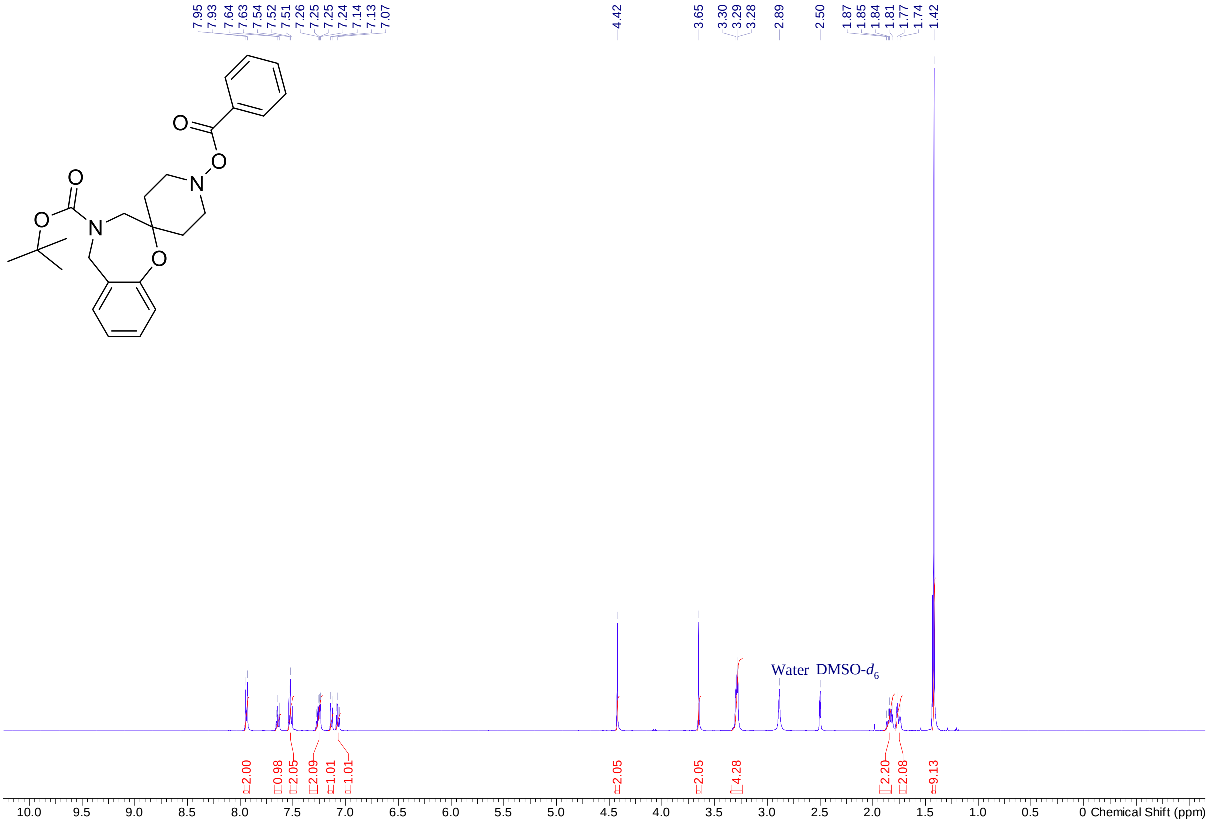

^{13}C NMR (126 MHz, 380 K, DMSO-d_6) of Compound **1g**

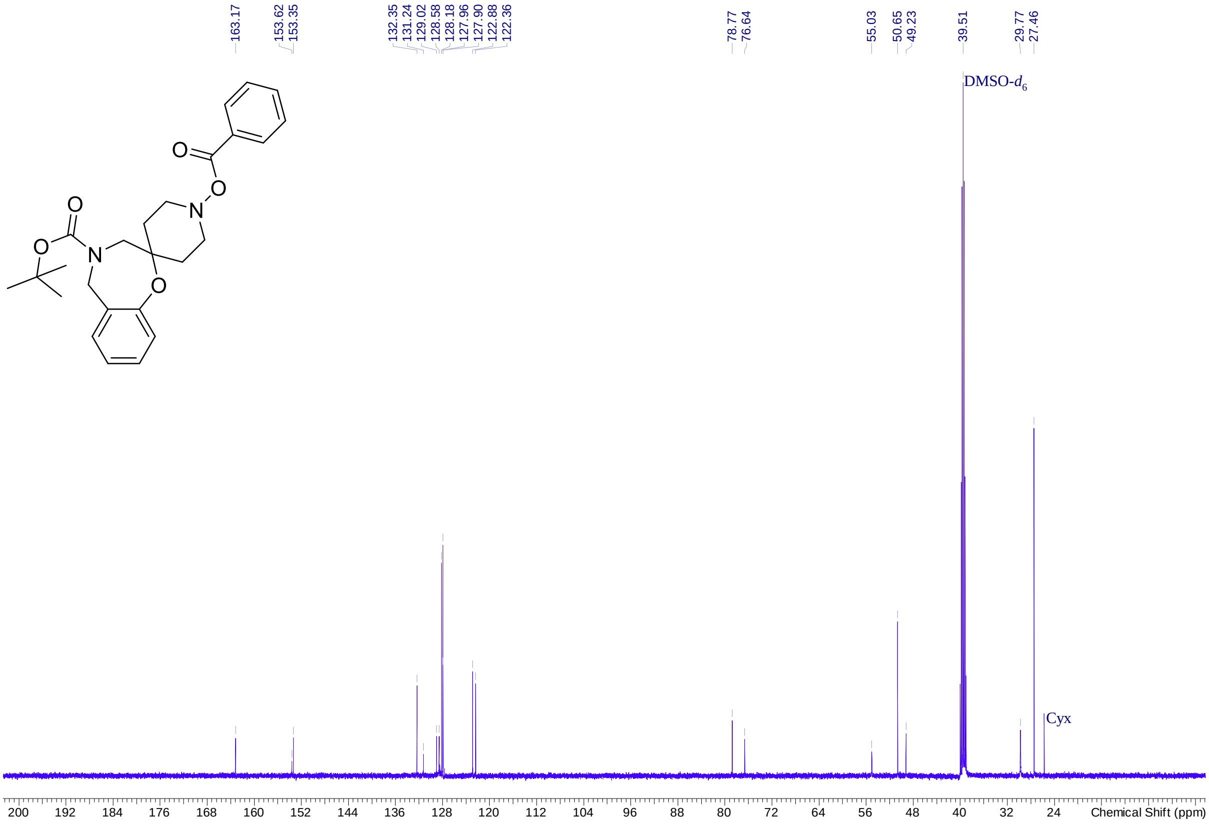

^{1}H NMR (600 MHz, DMSO-d_6) of Compound **1h**

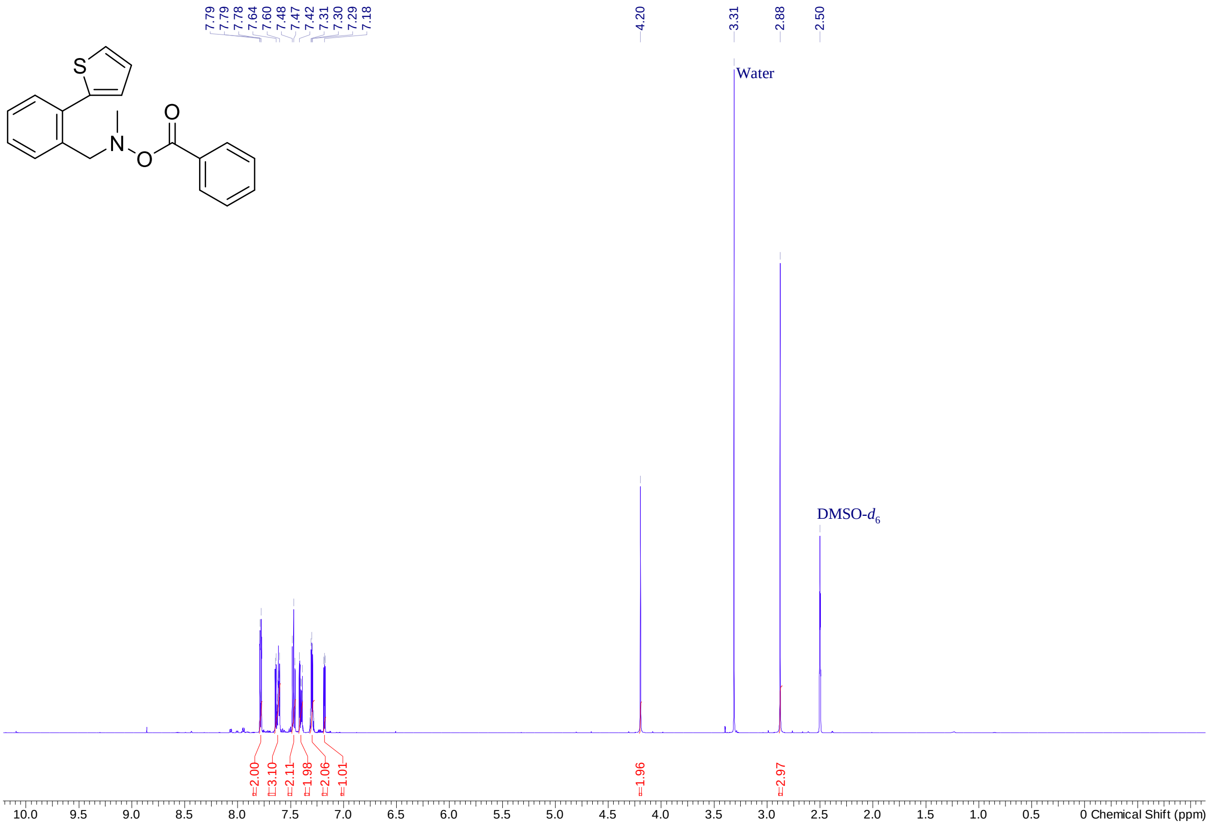

^{13}C NMR (151 MHz, DMSO-d_6) of Compound **1h**

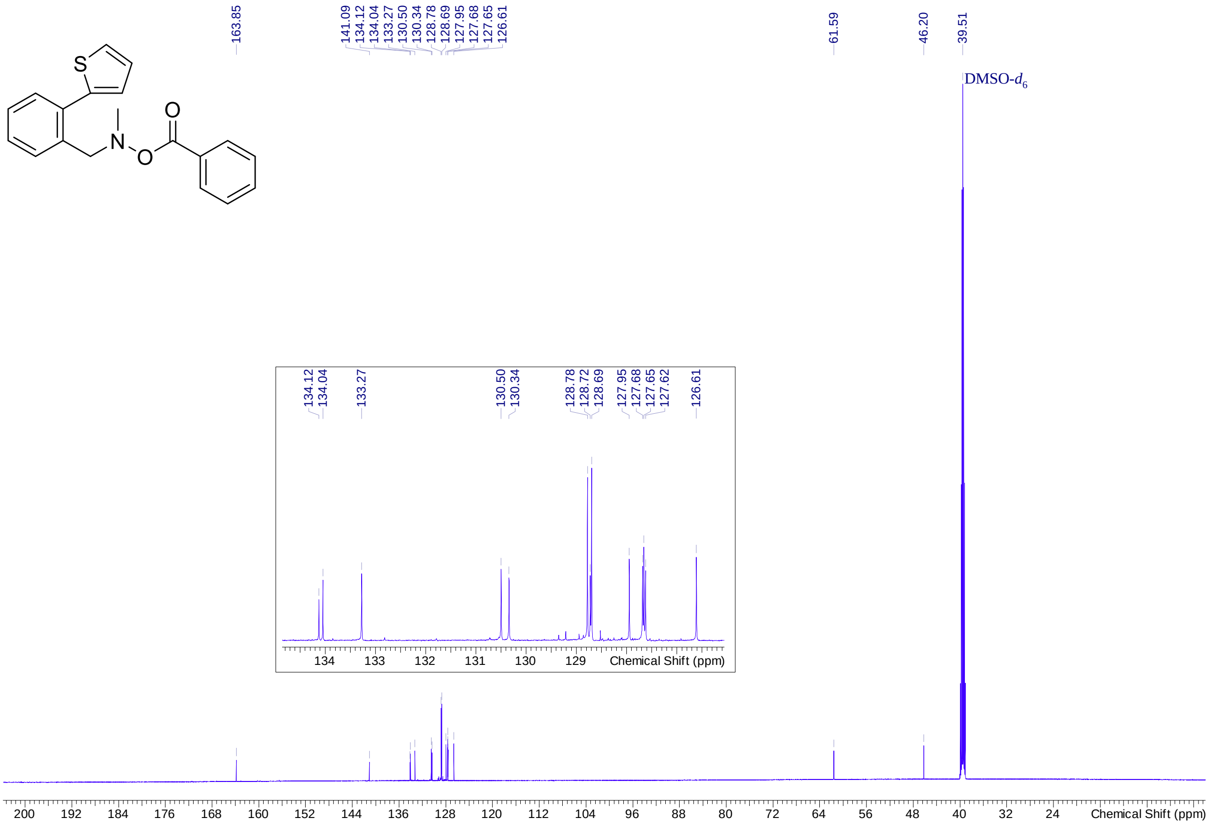

^{1}H NMR (400 MHz, $CDCl_3$) of Compound **1i**

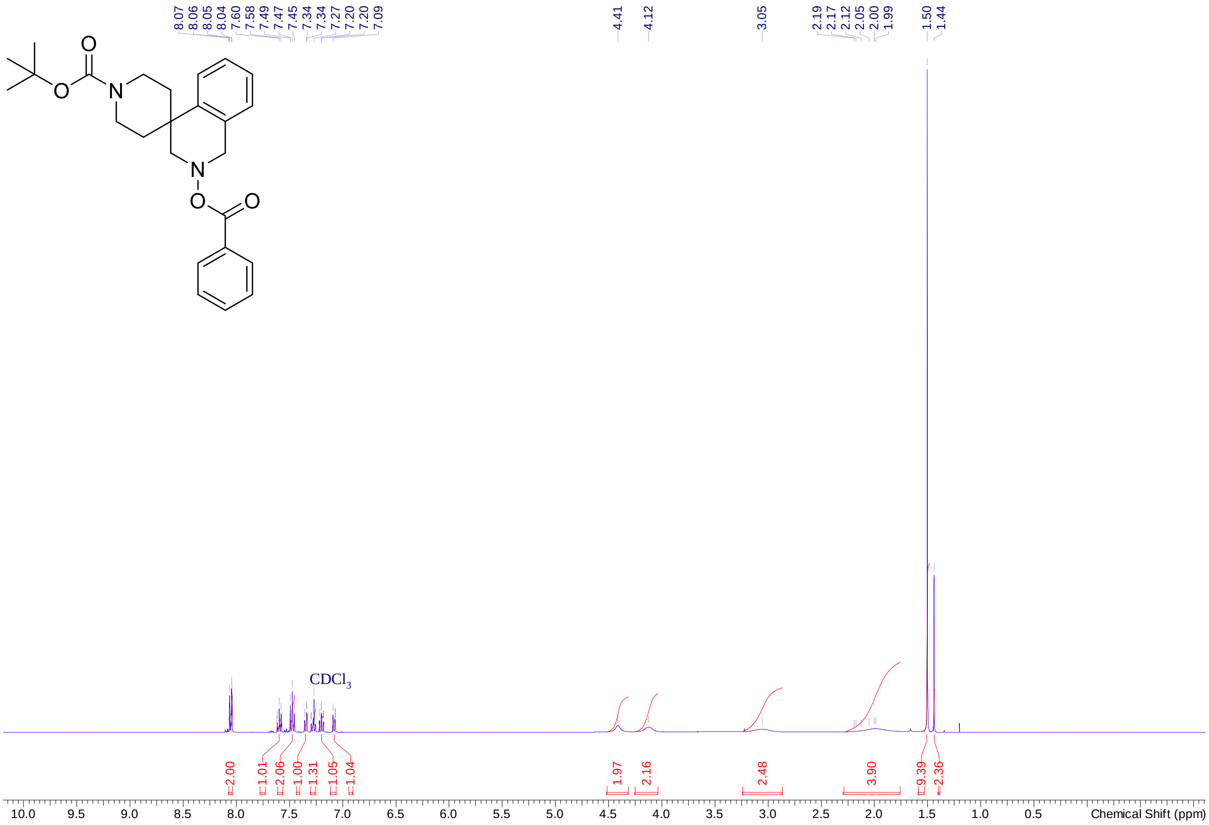

^{13}C NMR (101 MHz, $CDCl_3$) of Compound **1i**

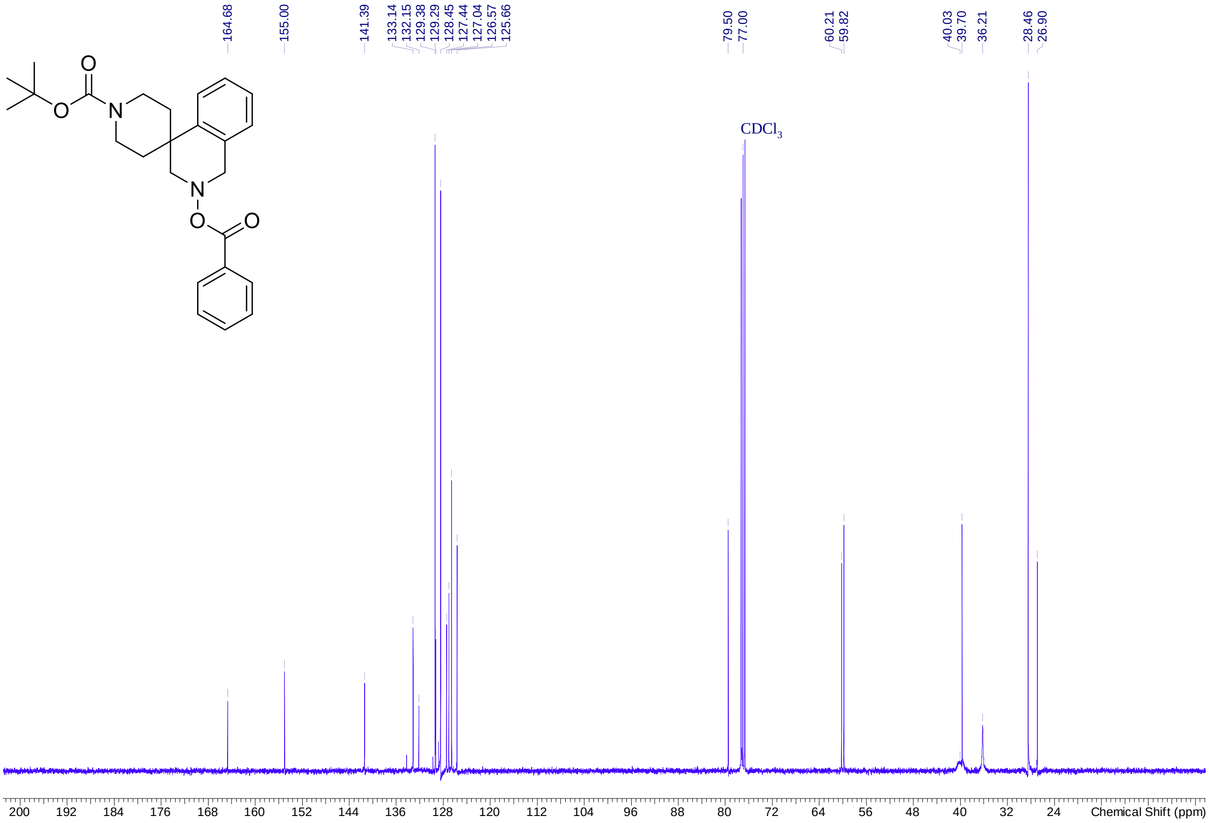

^{1}H NMR (400 MHz, 393 K, DMSO-d_6) of Compound **1j**

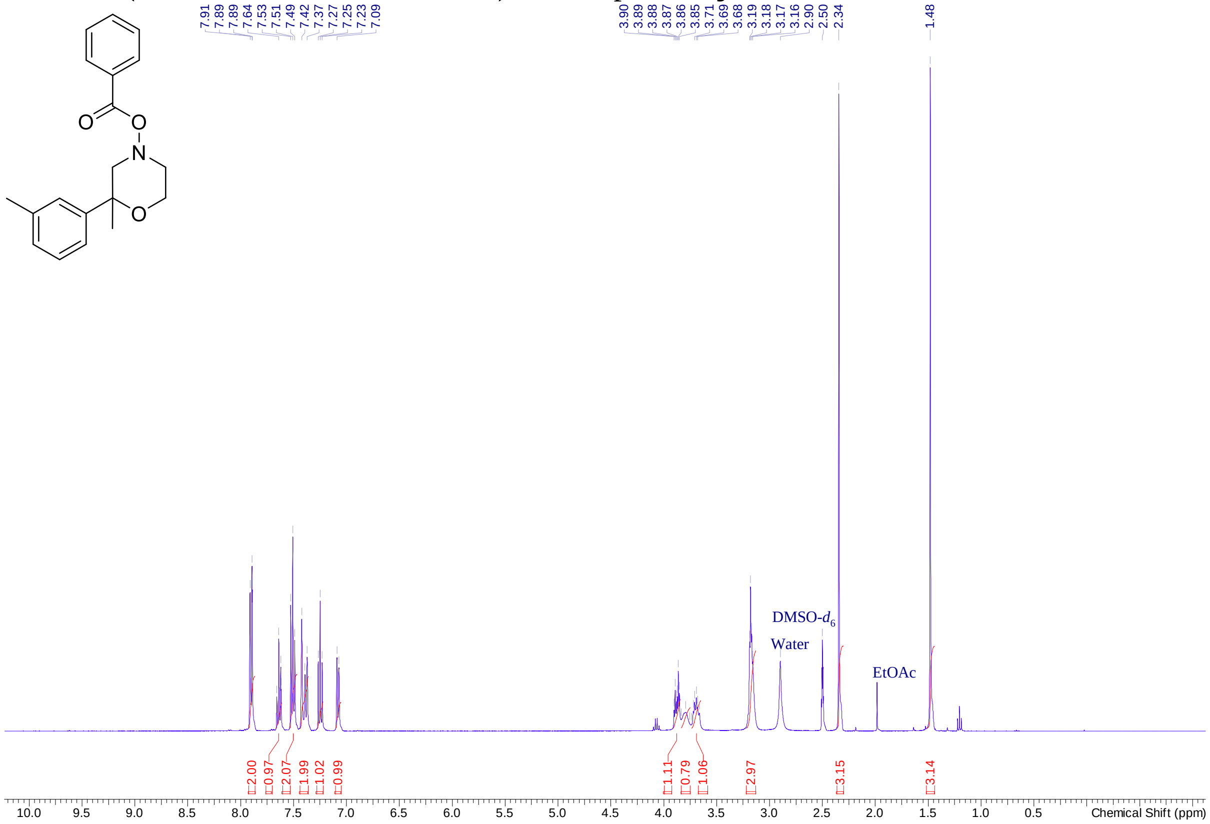

^{13}C NMR (101 MHz, DMSO-d_6) of Compound **1**j

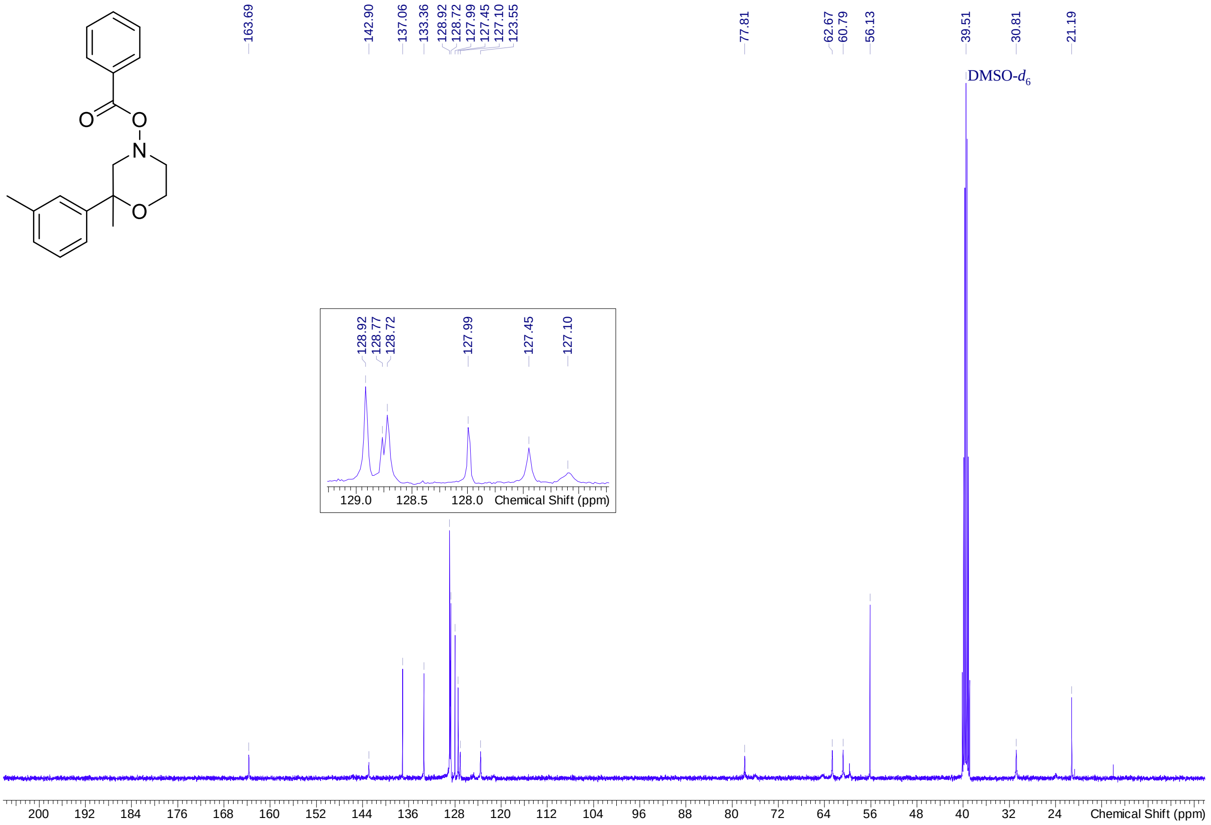

^{1}H NMR (400 MHz, 393 K, DMSO-d_6) of Compound **1k**

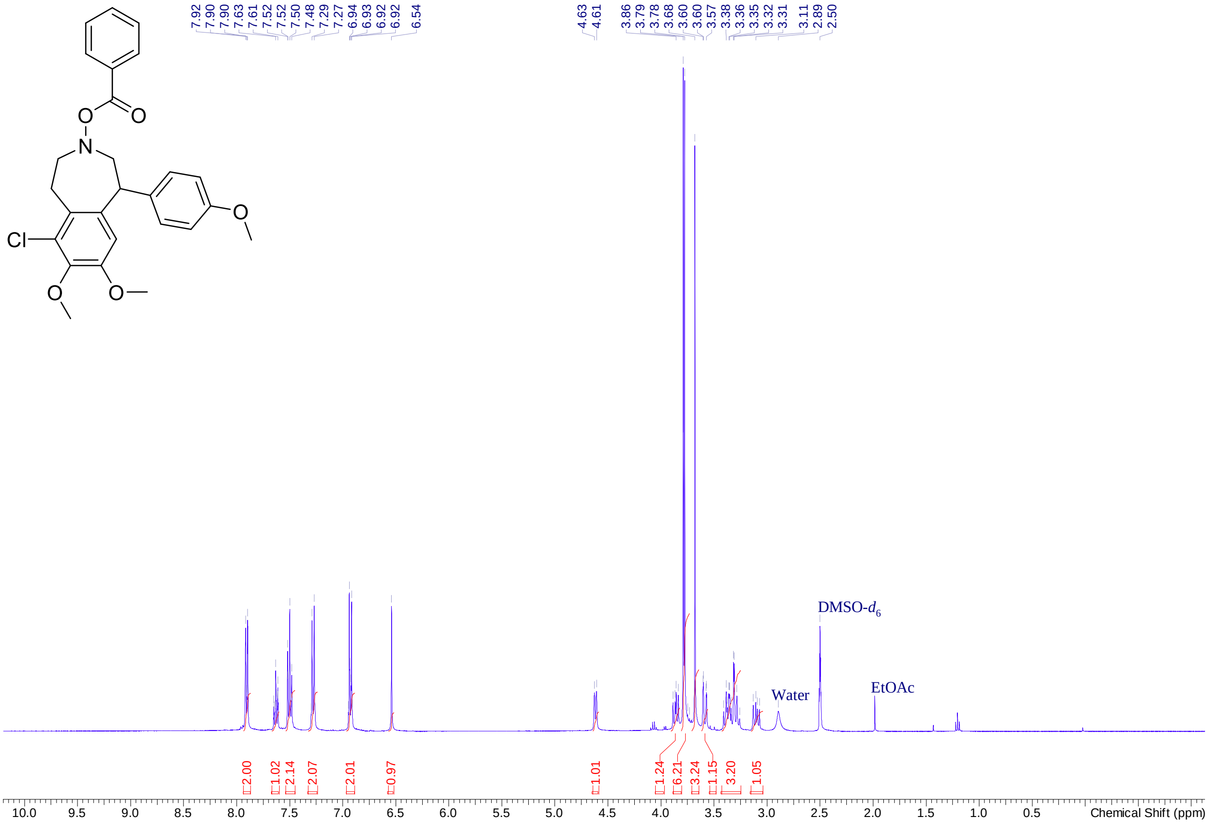

^{13}C NMR (101 MHz, DMSO-d_6) of Compound **1k**

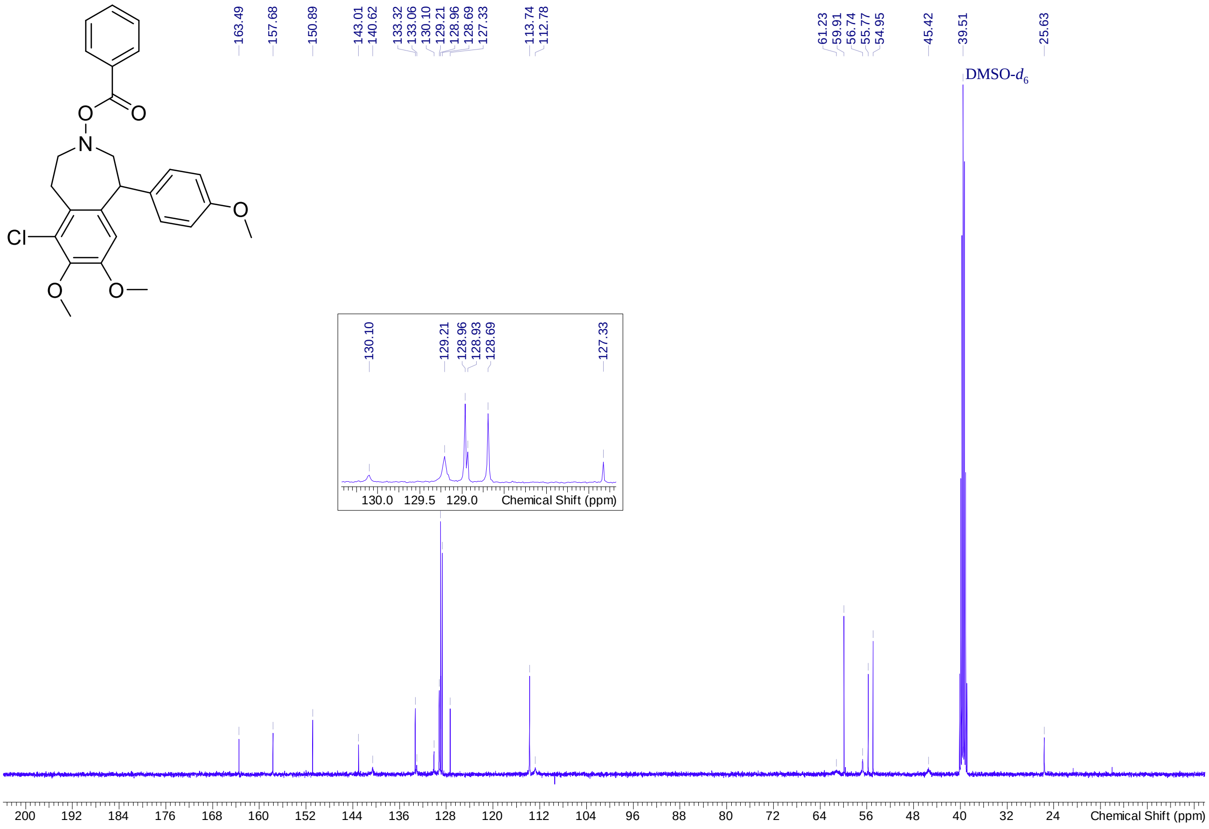

^{1}H NMR (400 MHz, DMSO-d_6) of Compound **1l**

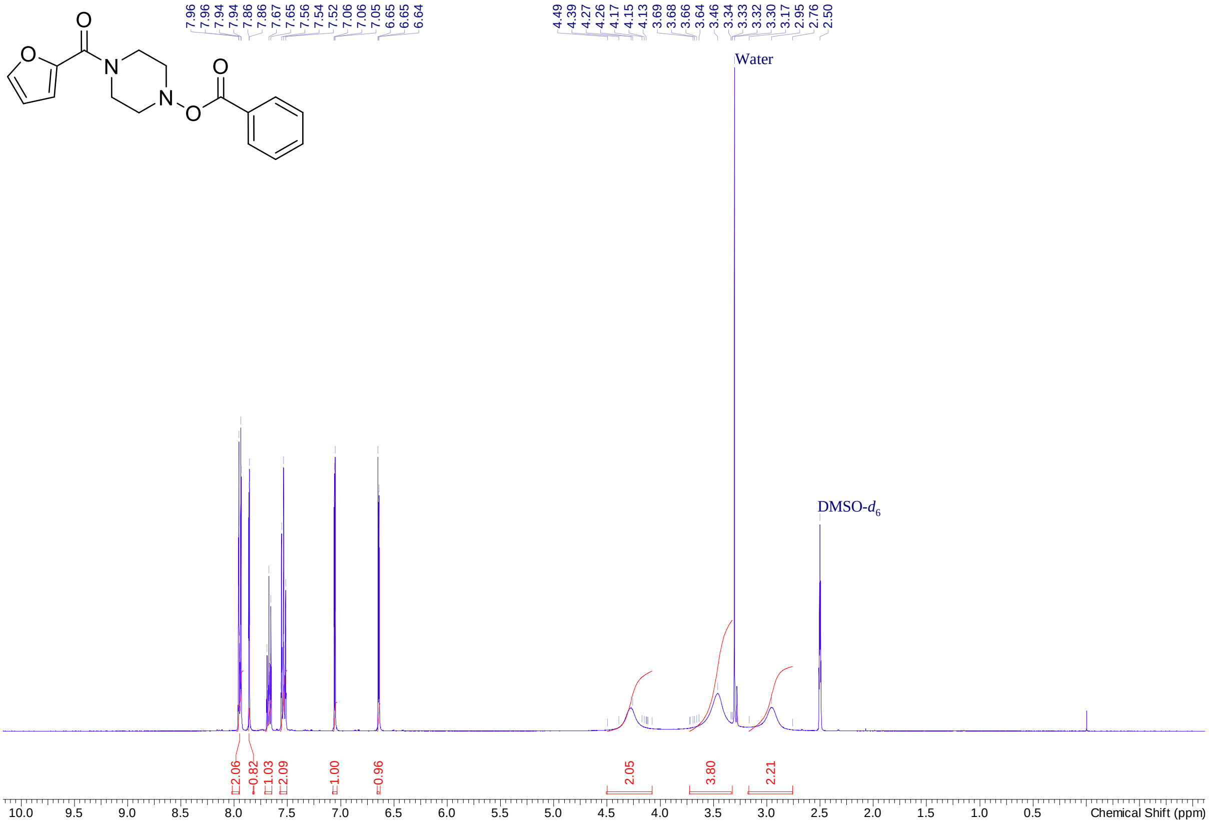

^{13}C NMR (151 MHz, DMSO-d_6) of Compound **1l**

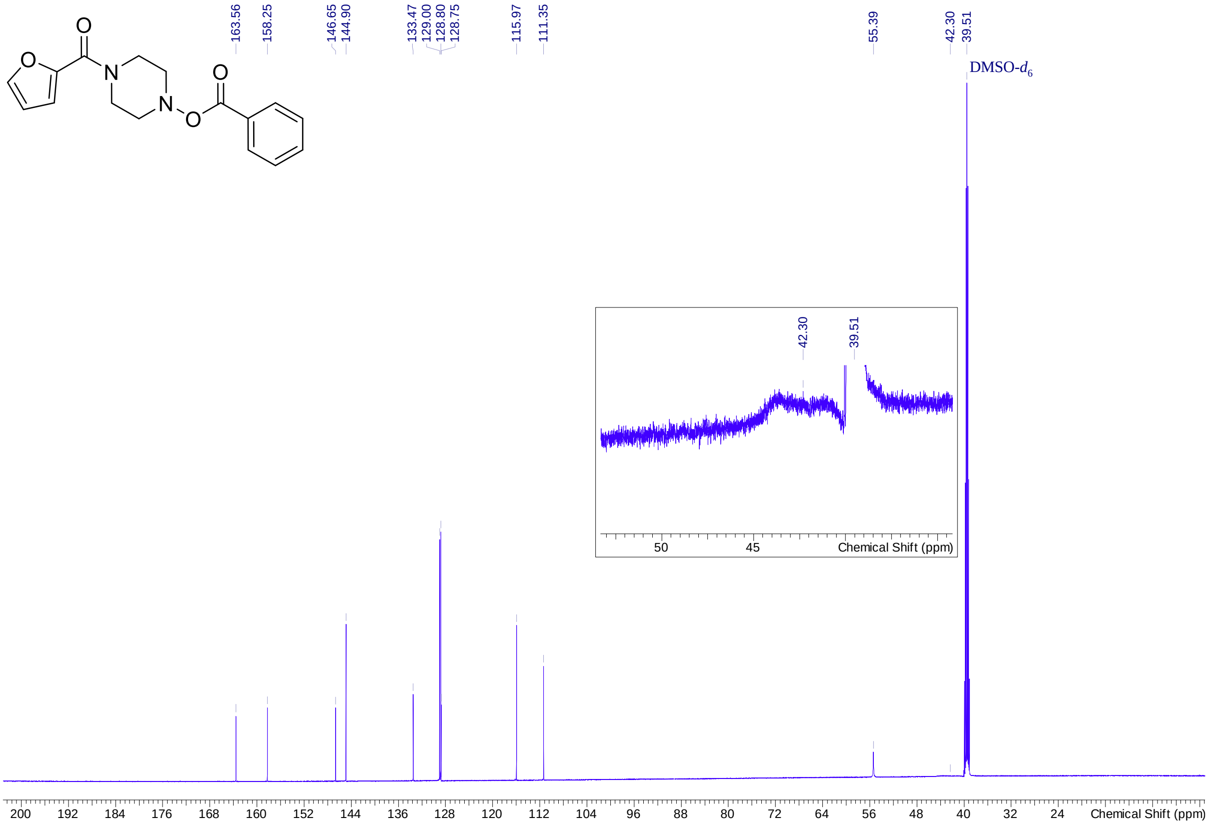

^{1}H NMR (400 MHz, DMSO-d_6) of Compound **1m**

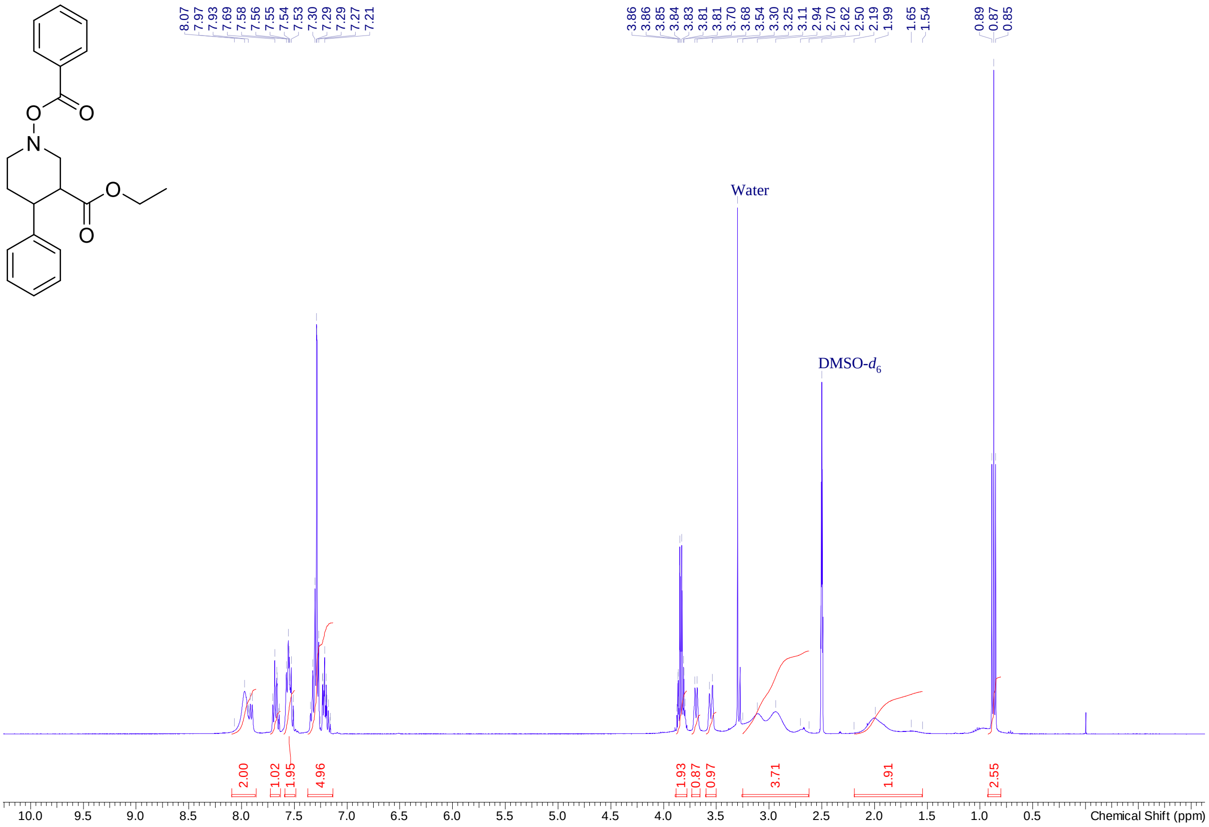

^{13}C NMR (151 MHz, DMSO-d_6) of Compound **1m**

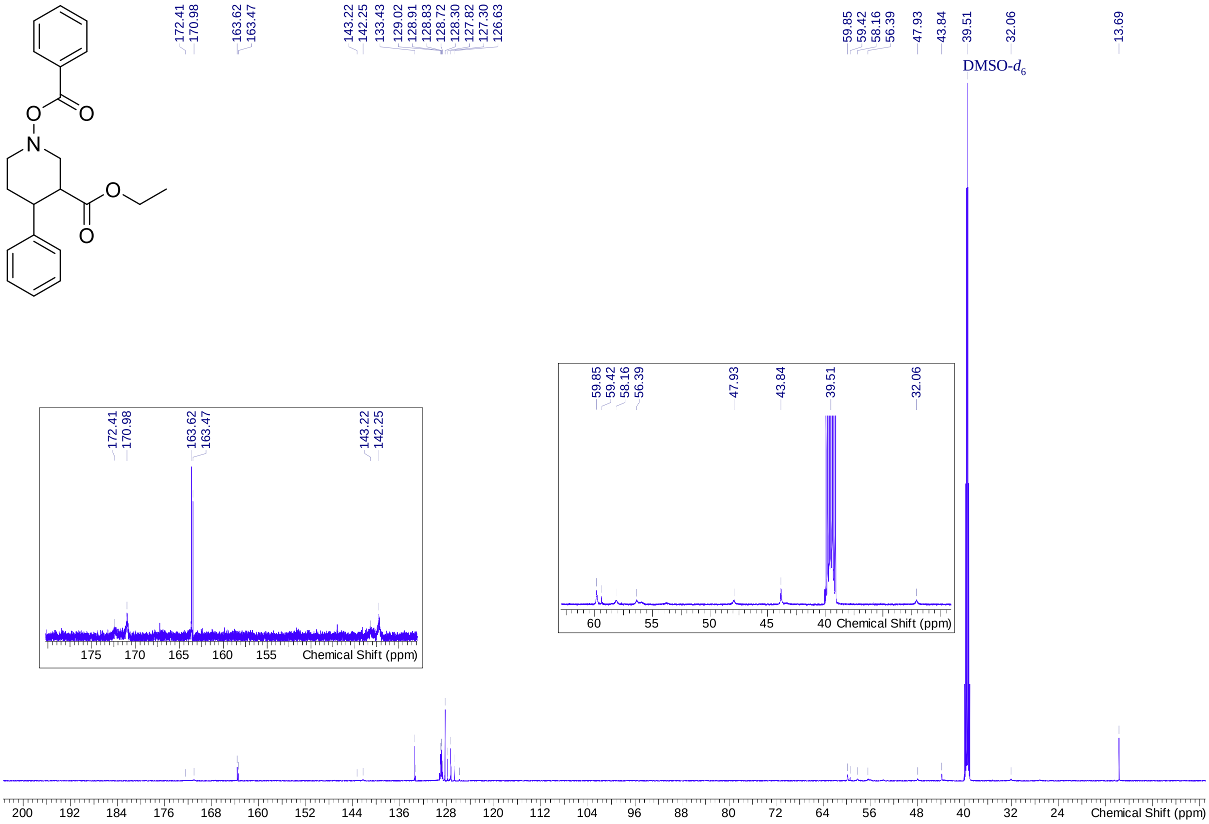

^{1}H NMR (400 MHz, 393 K, DMSO-d_6) of Compound **1m** (for *trans*:*cis* ratio)

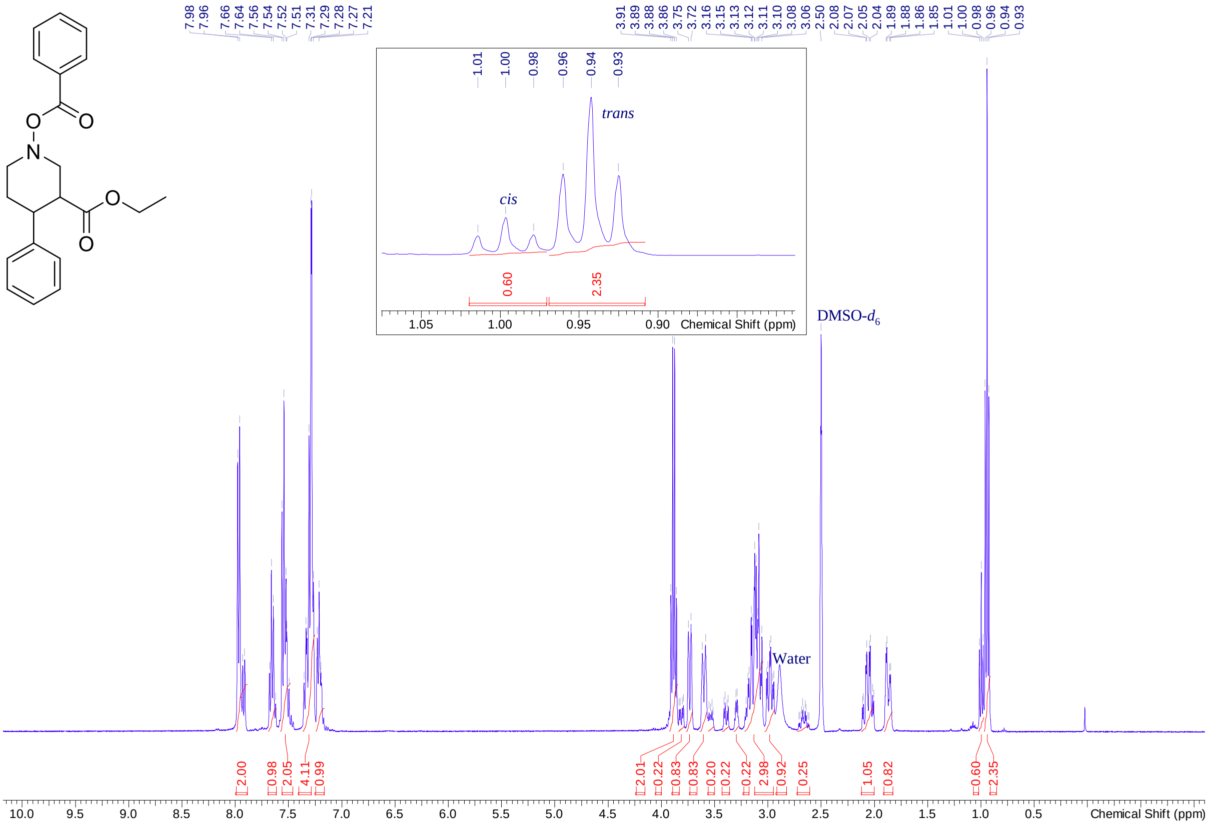

^{1}H NMR (600 MHz, DMSO-d_6) of Compound **1n**

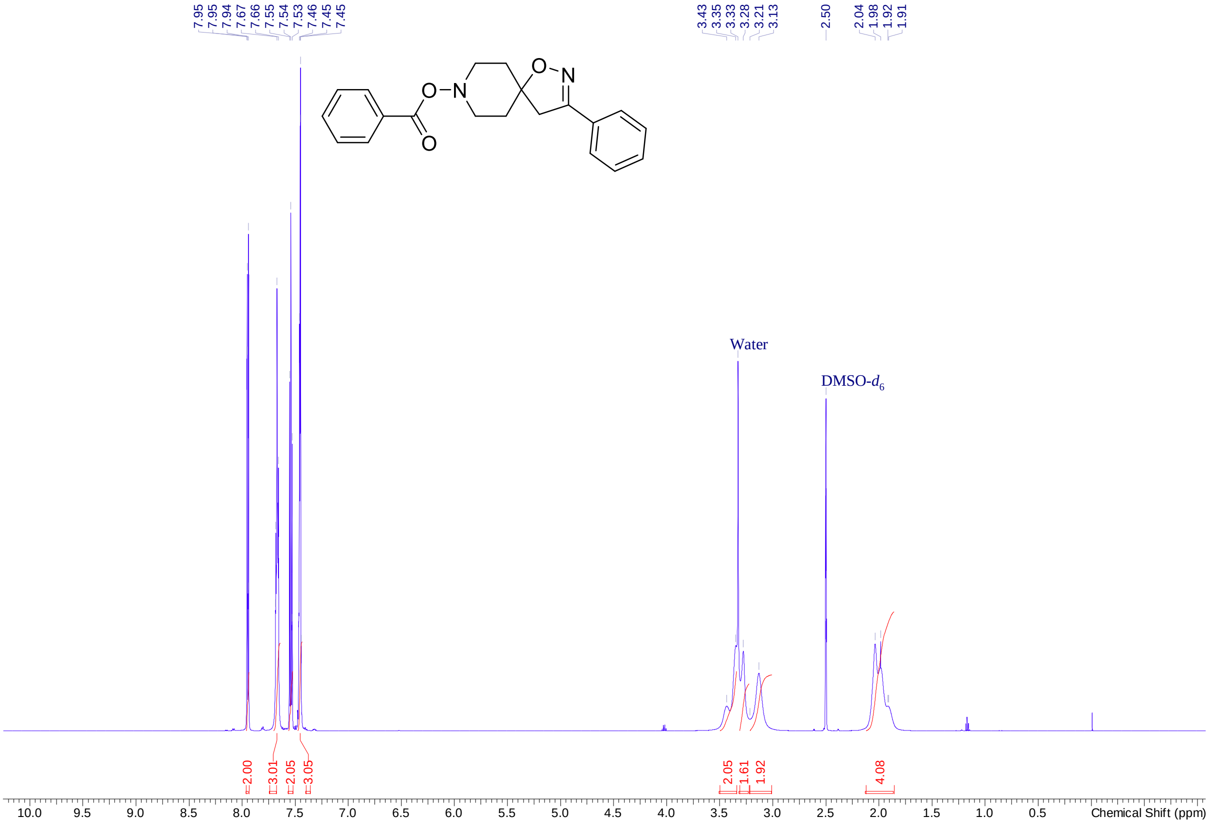

^{13}C NMR (151 MHz, DMSO-d_6) of Compound **1n**

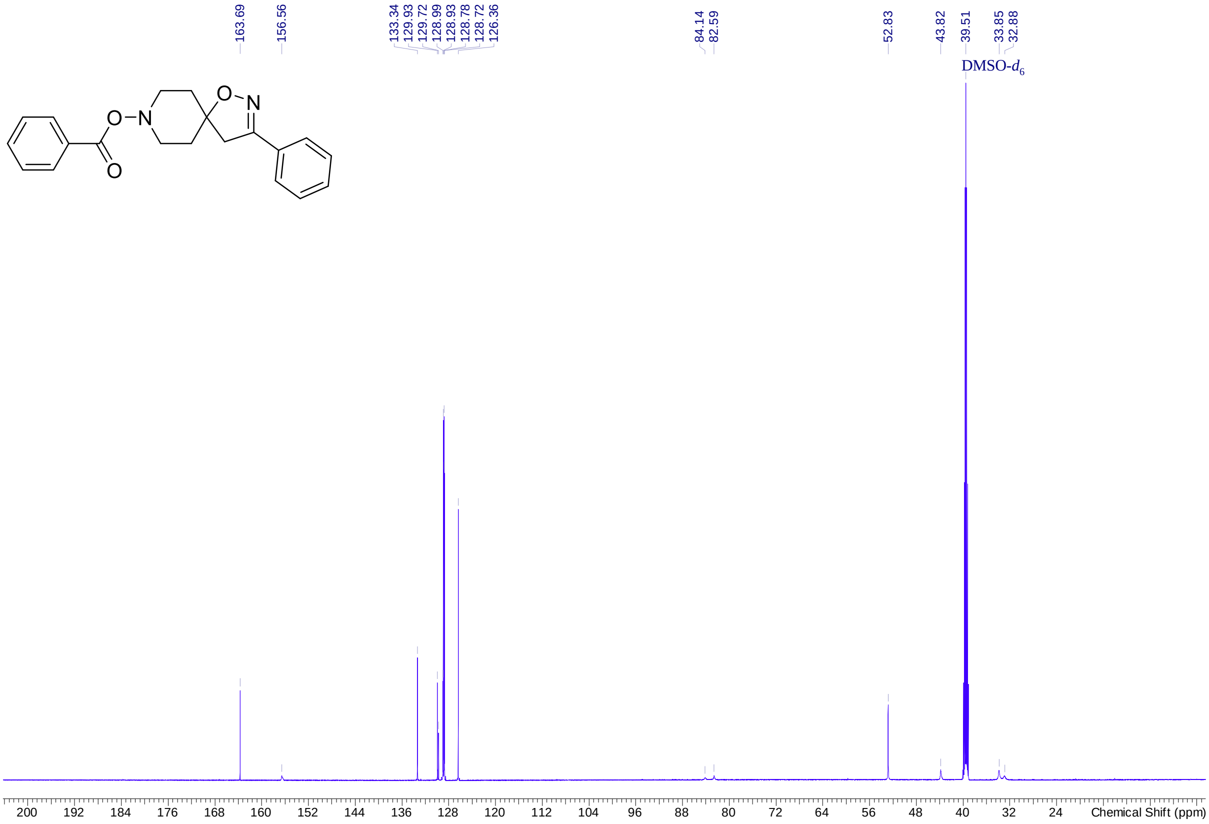

^{1}H NMR (400 MHz, 393 K, DMSO-d_6) of Compound **1o**

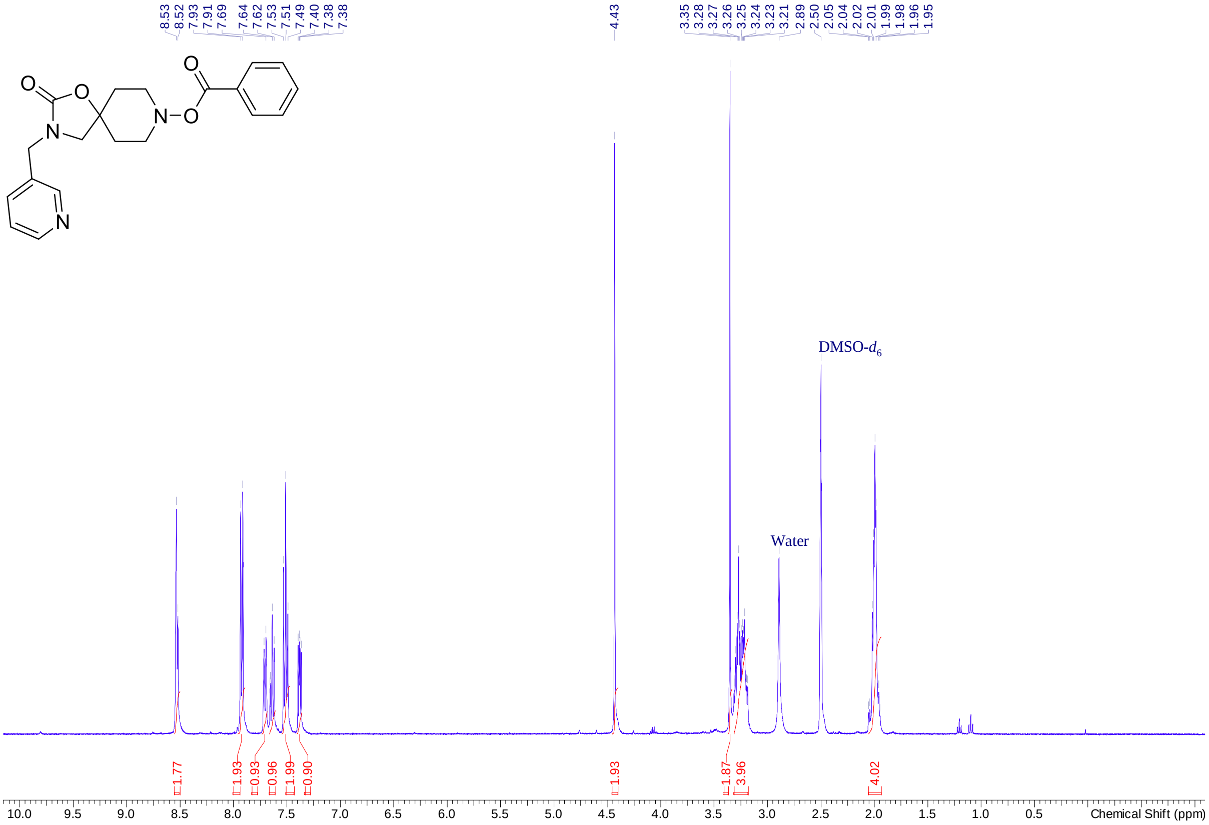

^{13}C NMR (101 MHz, DMSO-d_6) of Compound **1o**

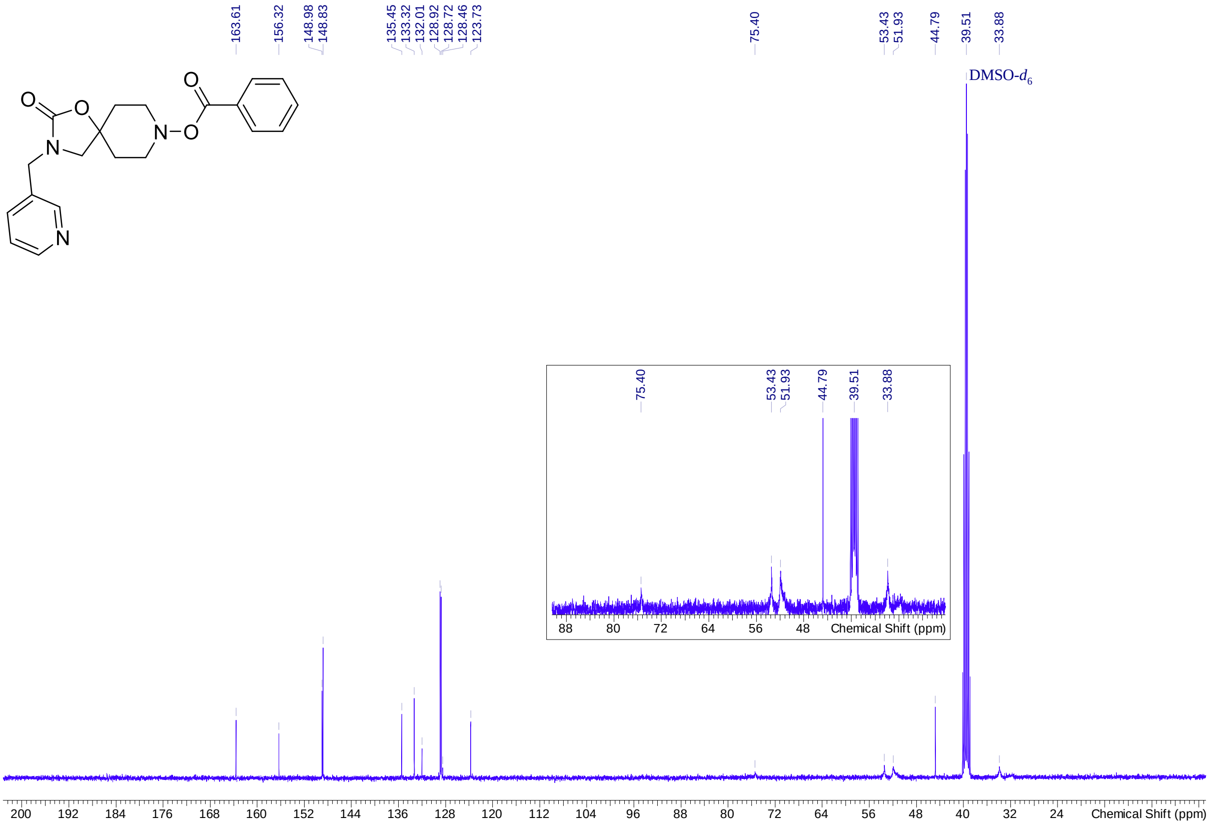

^{1}H NMR (400 MHz, DMSO-d_6) of Compound **1p**

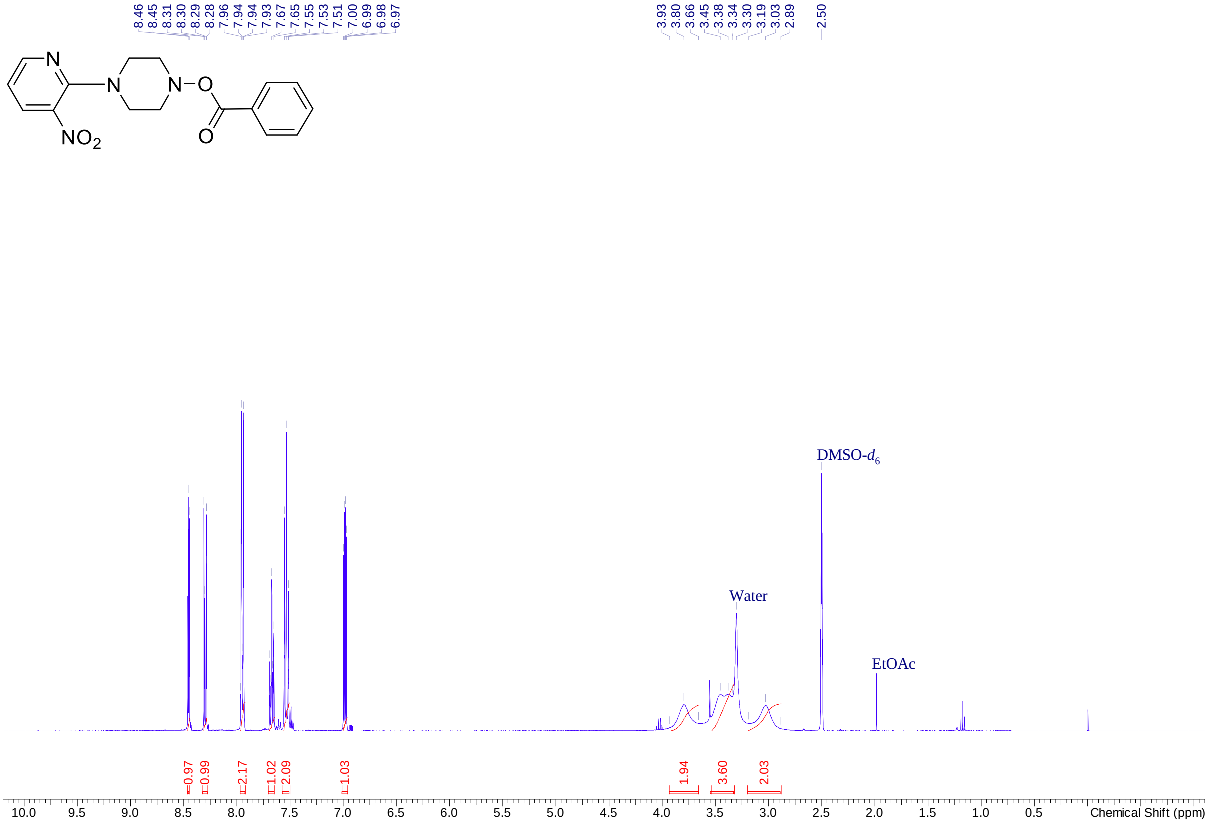

^{13}C NMR (101 MHz, DMSO-d_6) of Compound **1p**

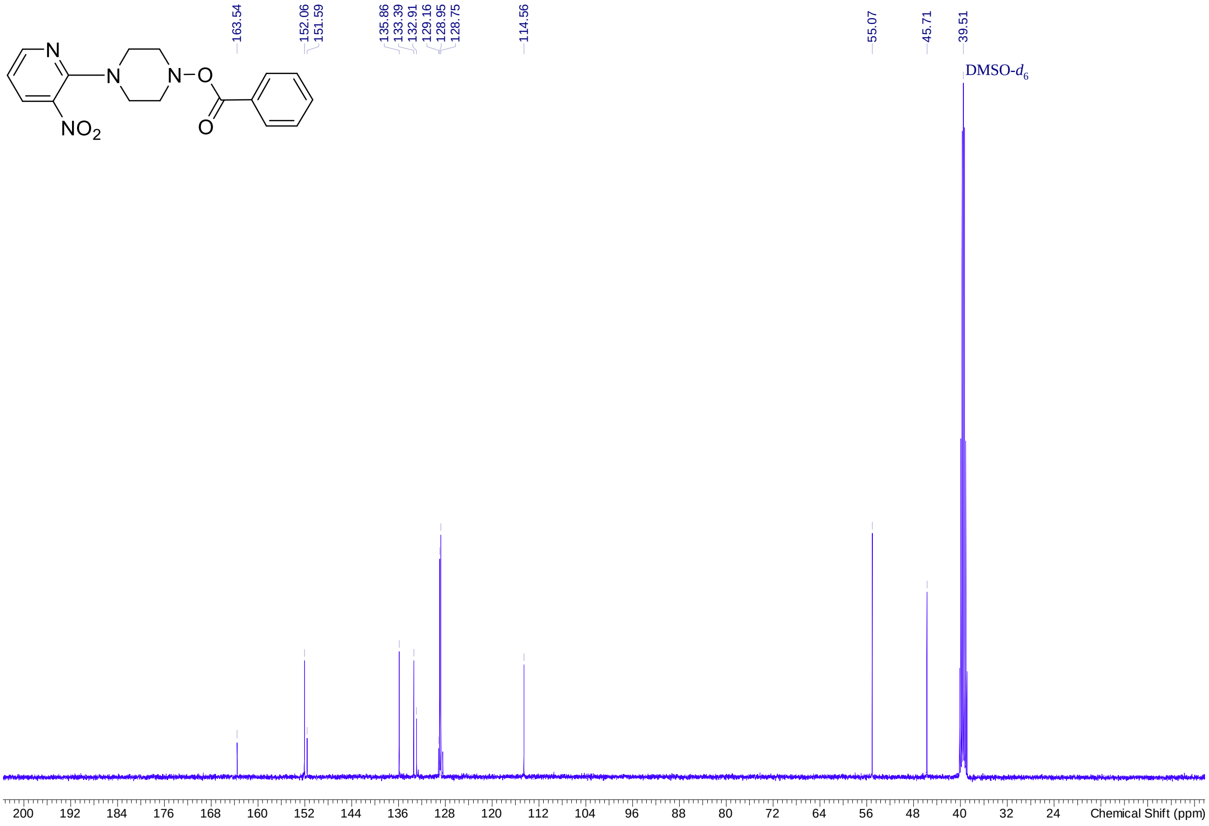

^{1}H NMR (400 MHz, DMSO-d_6) of Compound **1q**

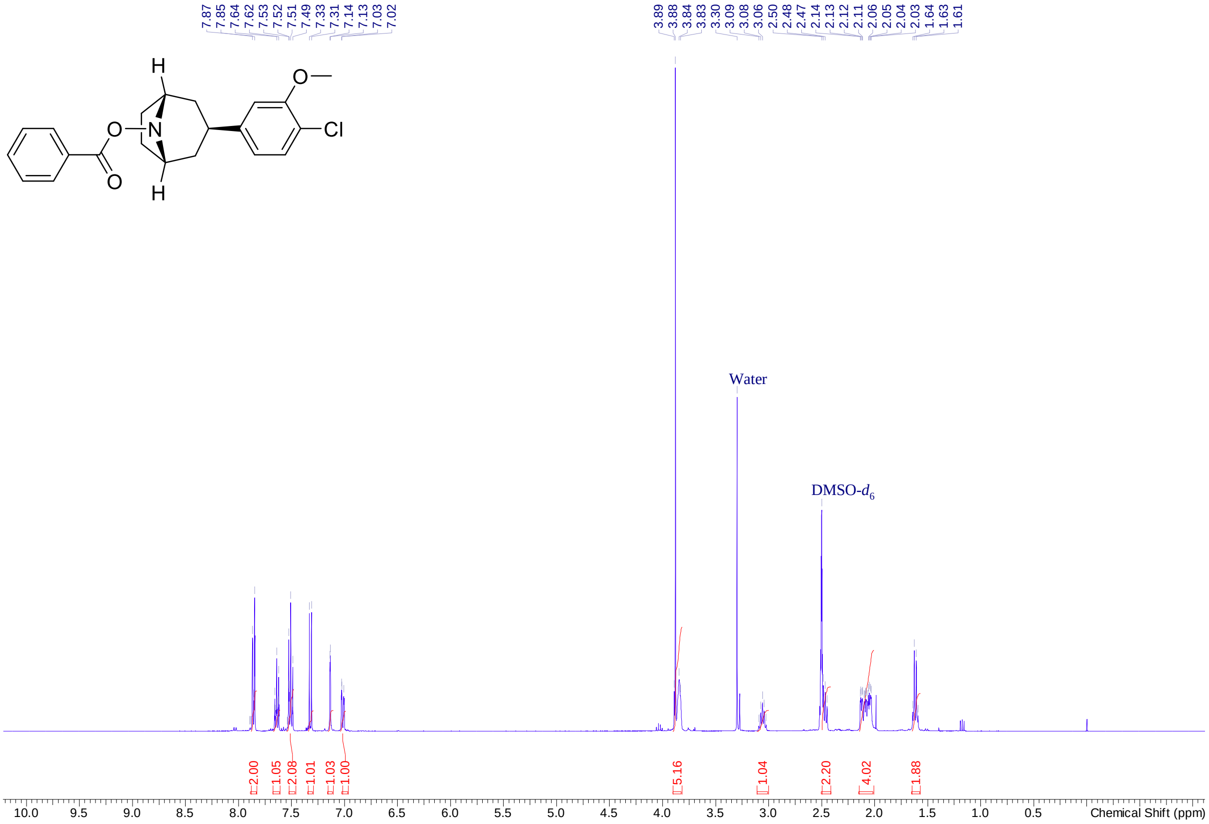

^{13}C NMR (101 MHz, DMSO-d_6) of Compound **1q**

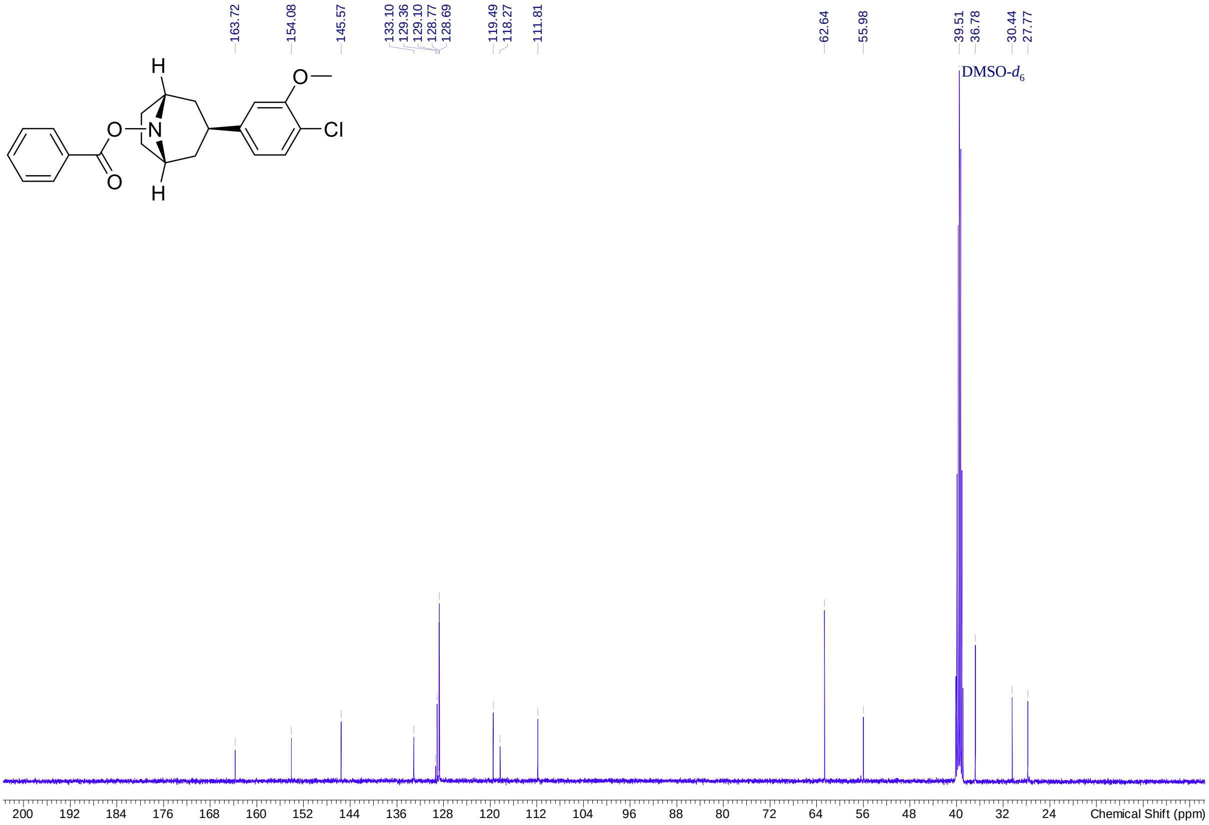

^{1}H NMR (400 MHz, DMSO-d_6) of Compound **1r**

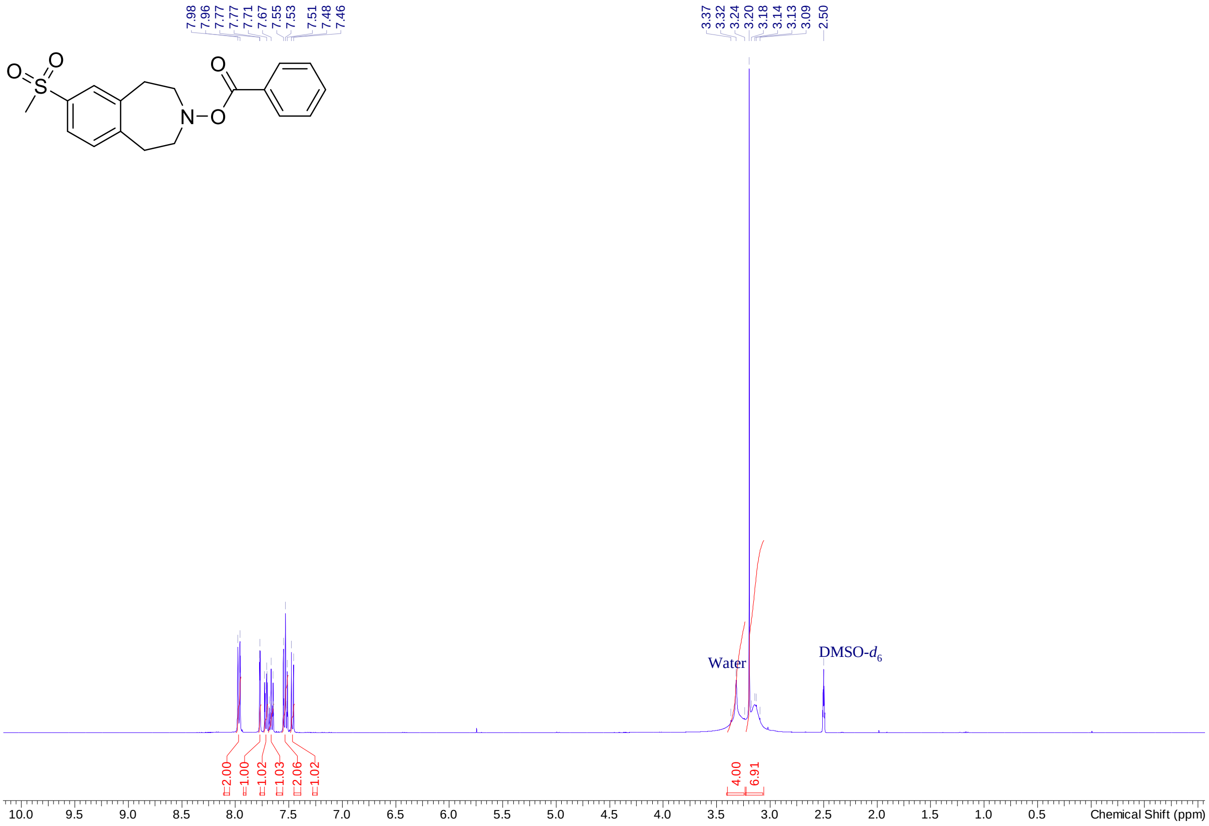

^{13}C NMR (101 MHz, DMSO-d_6) of Compound **1r**

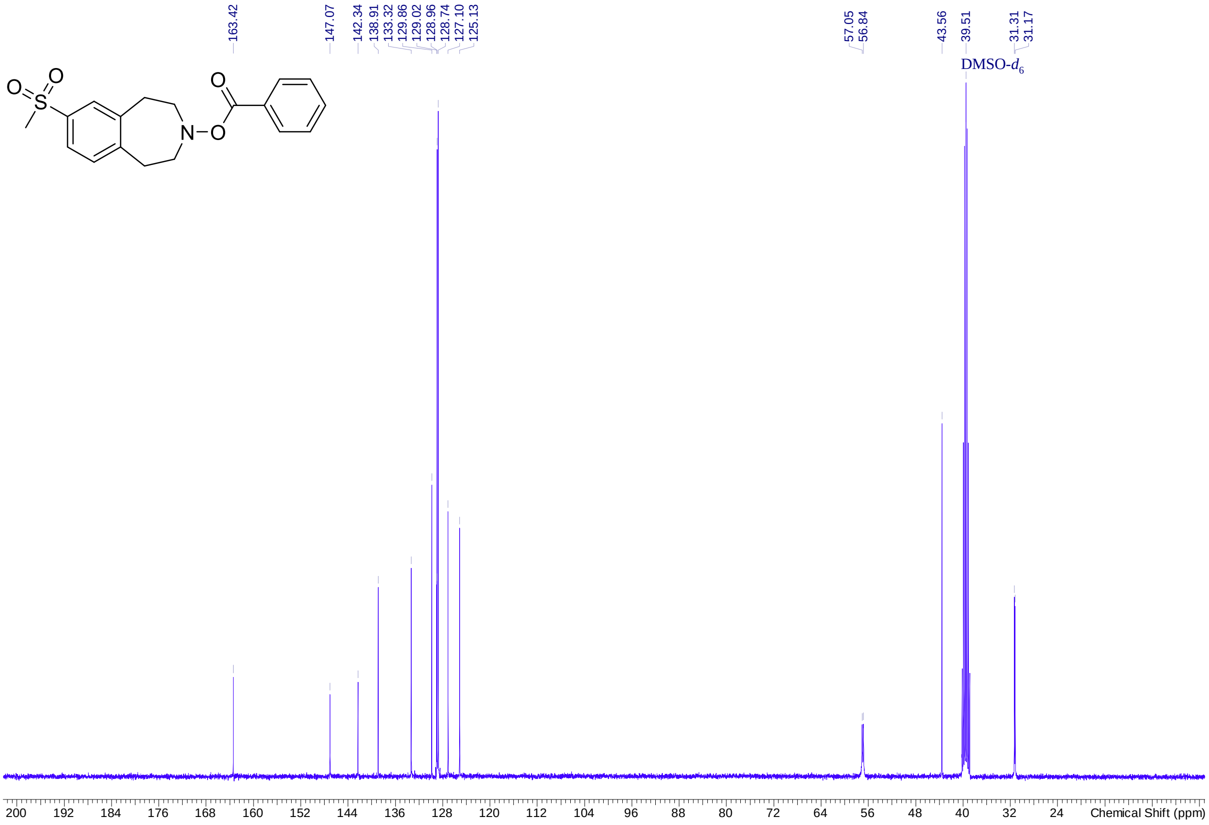

^{1}H NMR (400 MHz, DMSO-d_6) of Compound **1s**

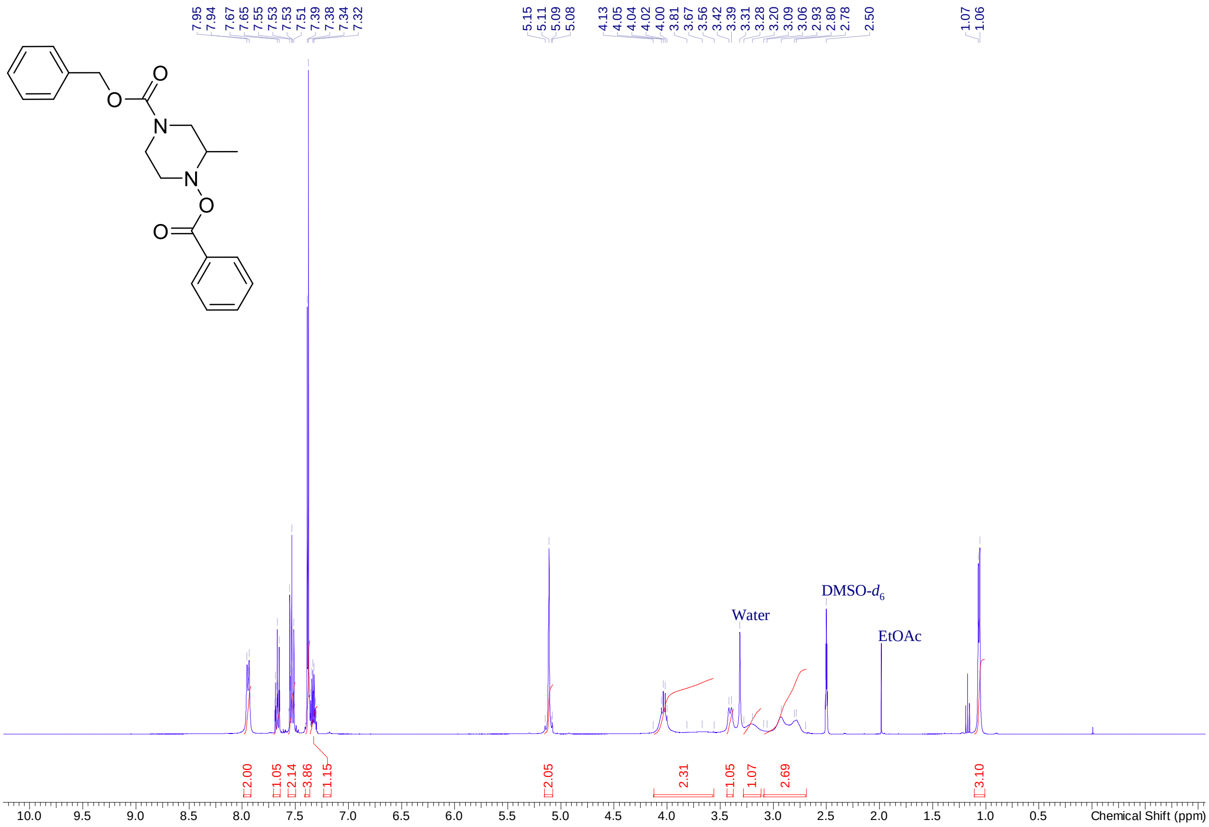

^{13}C NMR (101 MHz, DMSO-d_6) of Compound **1s**

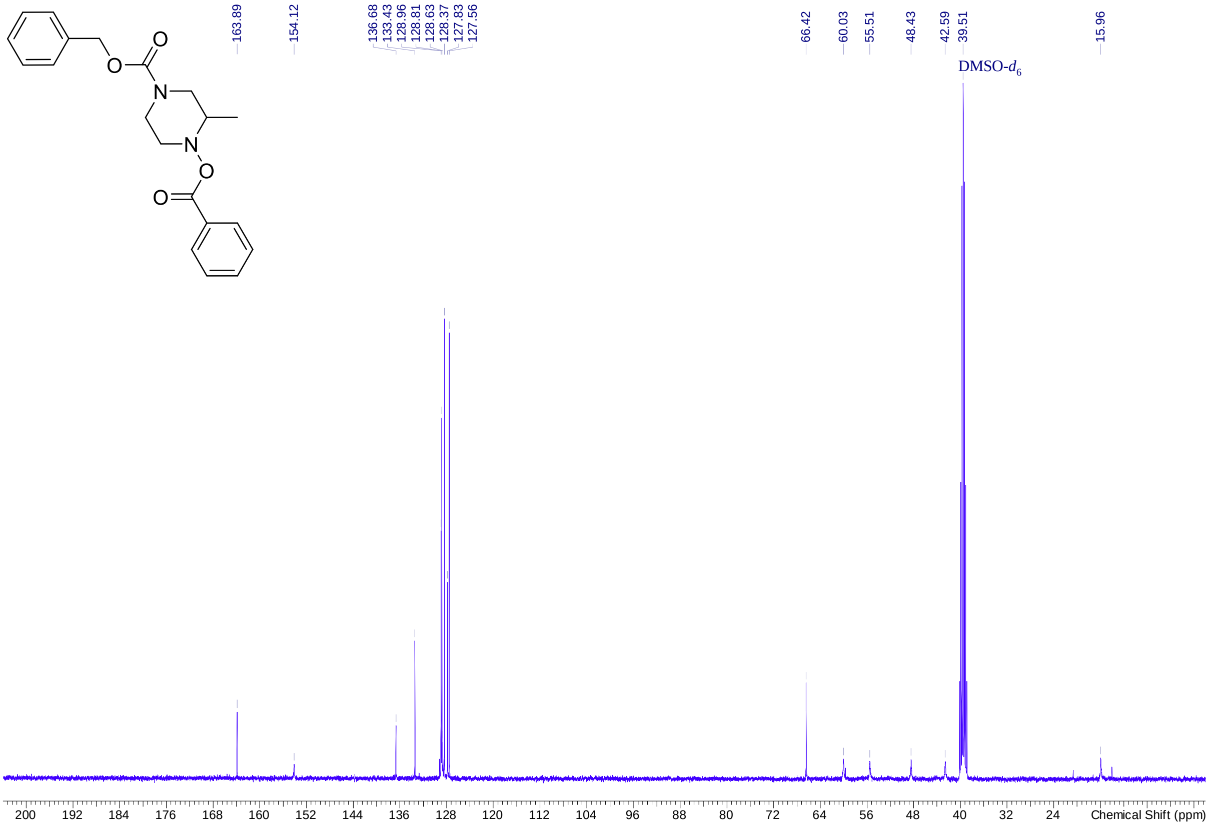

^{1}H NMR (400 MHz, DMSO-d_6) of Compound **1t**

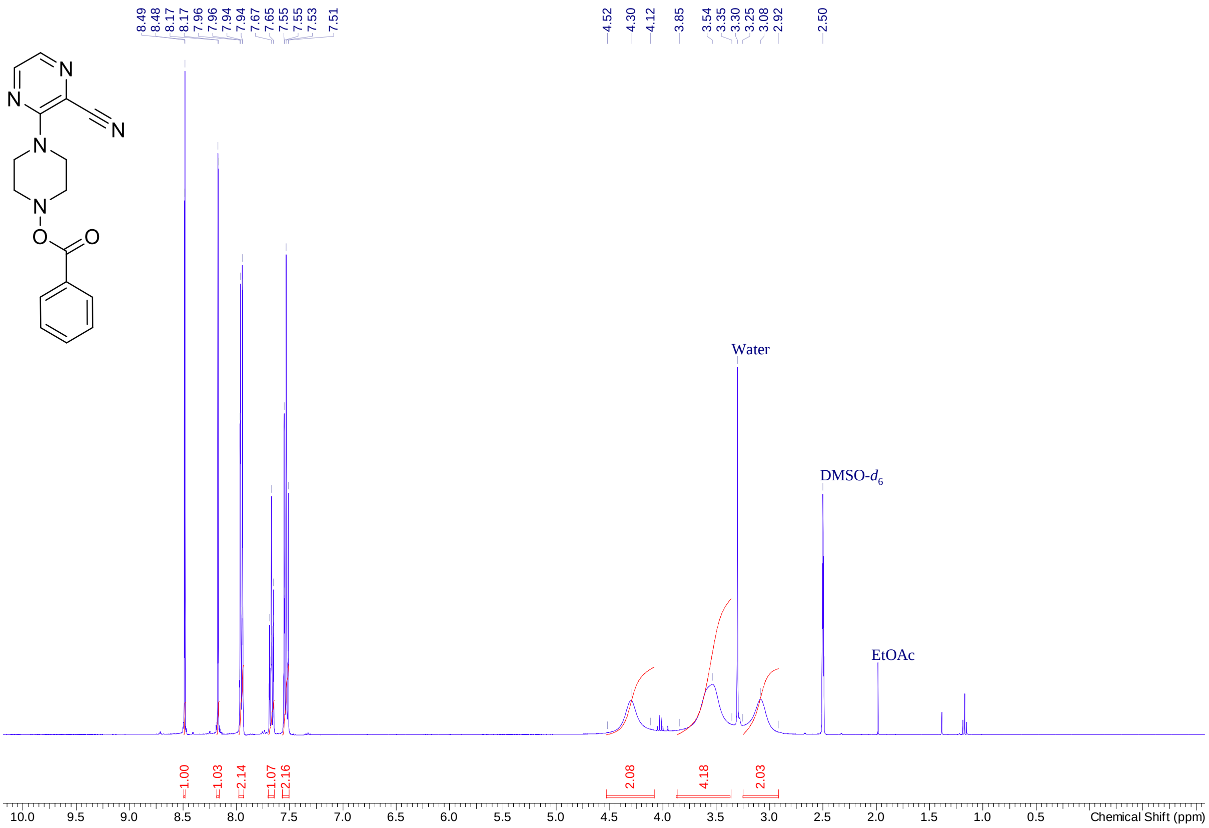

^{13}C NMR (151 MHz, DMSO-d_6) of Compound **1t**

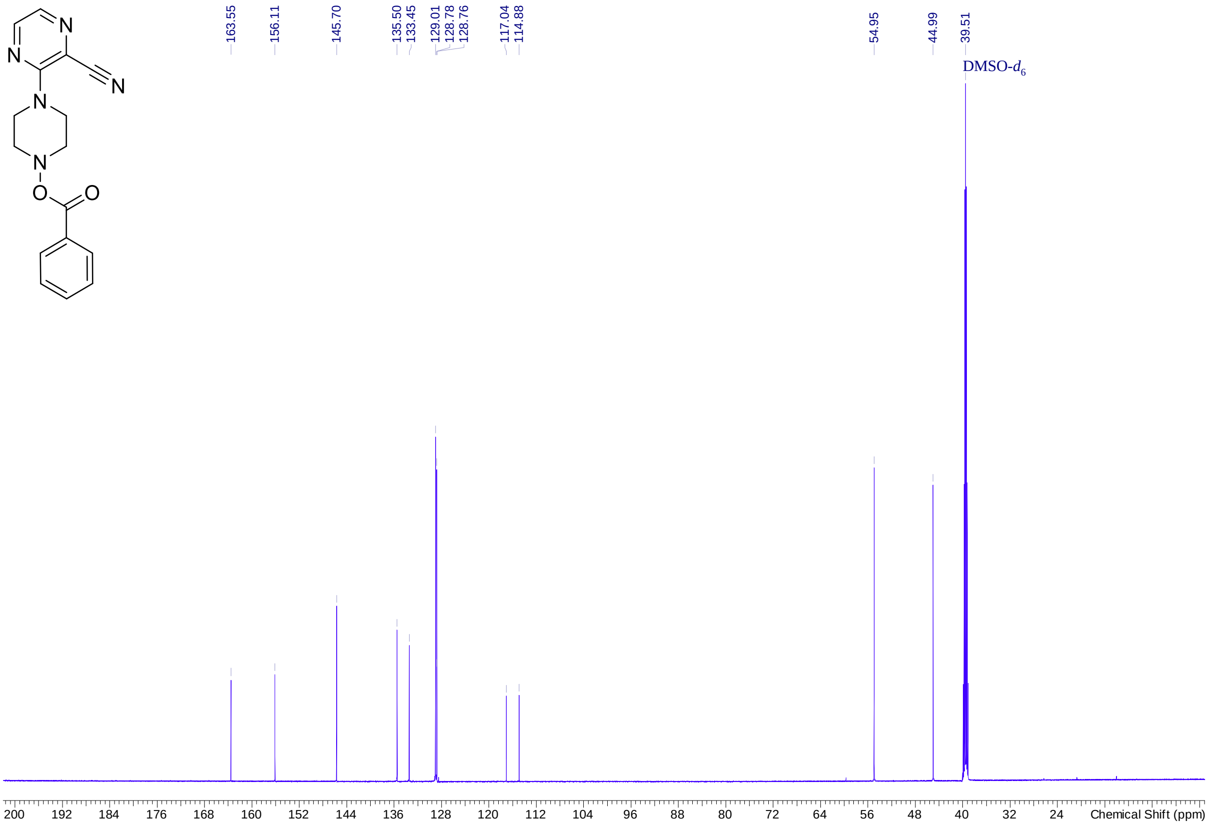

^{1}H NMR (400 MHz, DMSO-d_6) of Compound **1u**

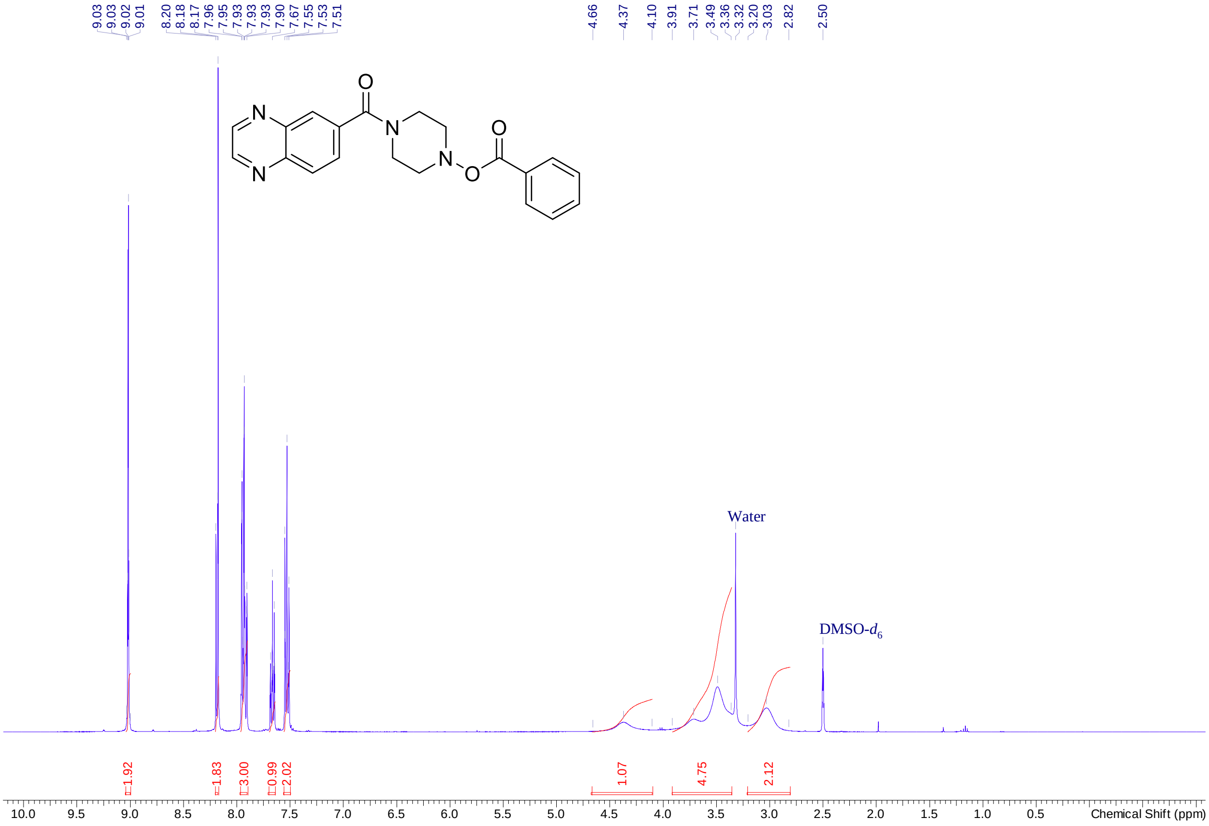

^{13}C NMR (151 MHz, DMSO-d_6) of Compound **1u**

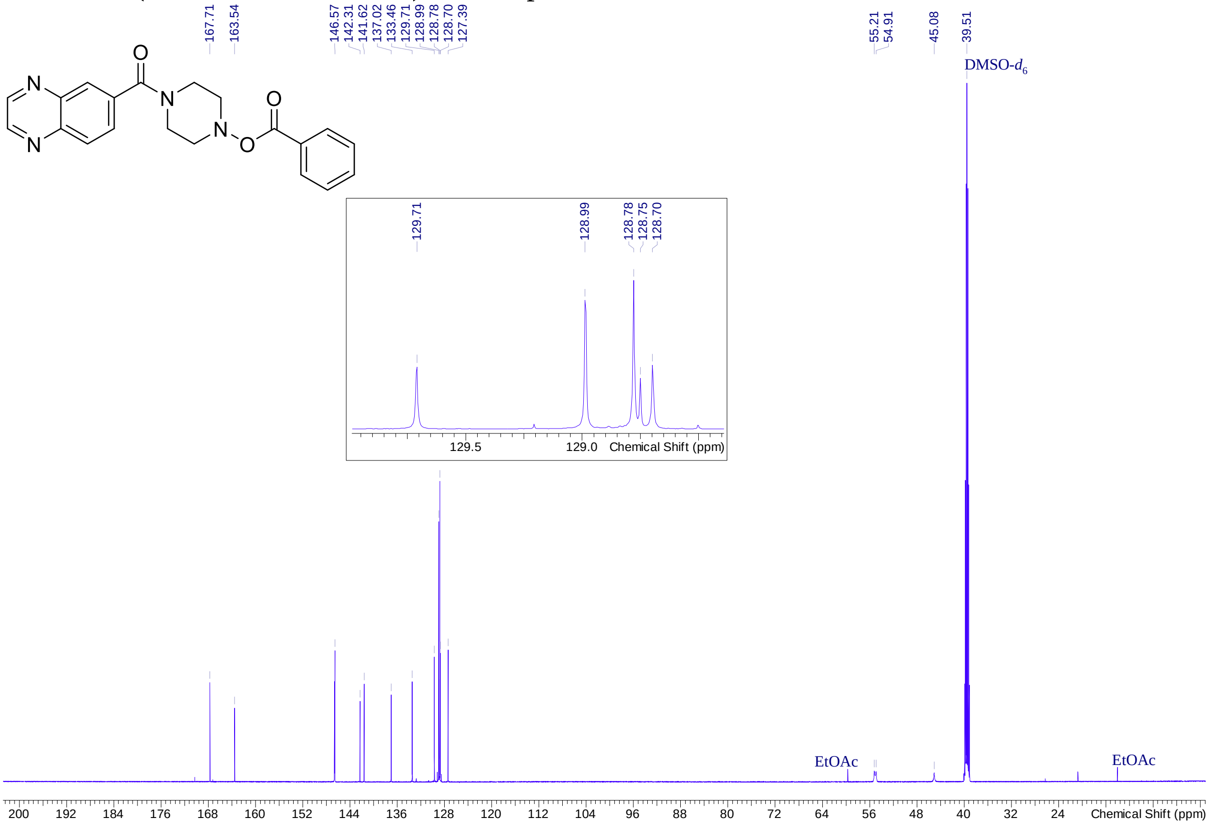

^{1}H NMR (400 MHz, DMSO-d_6) of Compound **1v**

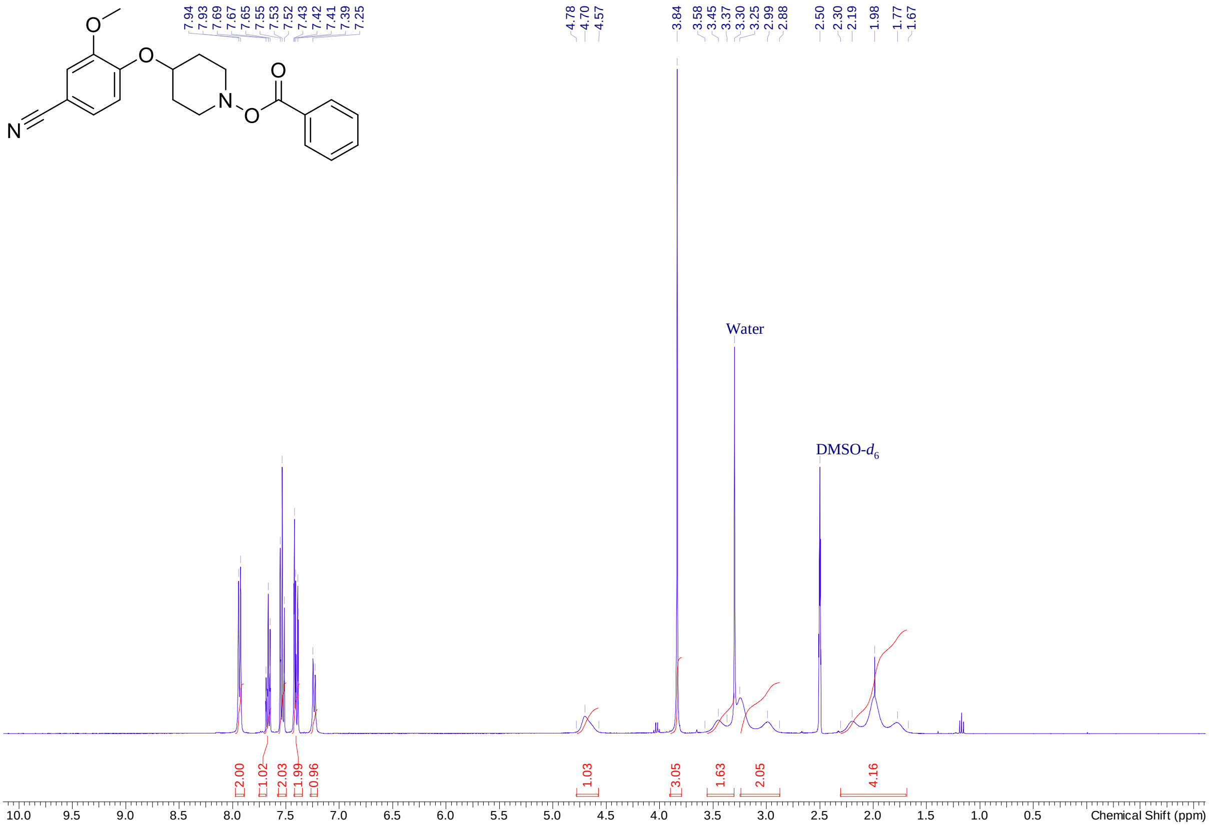

^{13}C NMR (101 MHz, DMSO-d_6) of Compound **1v**

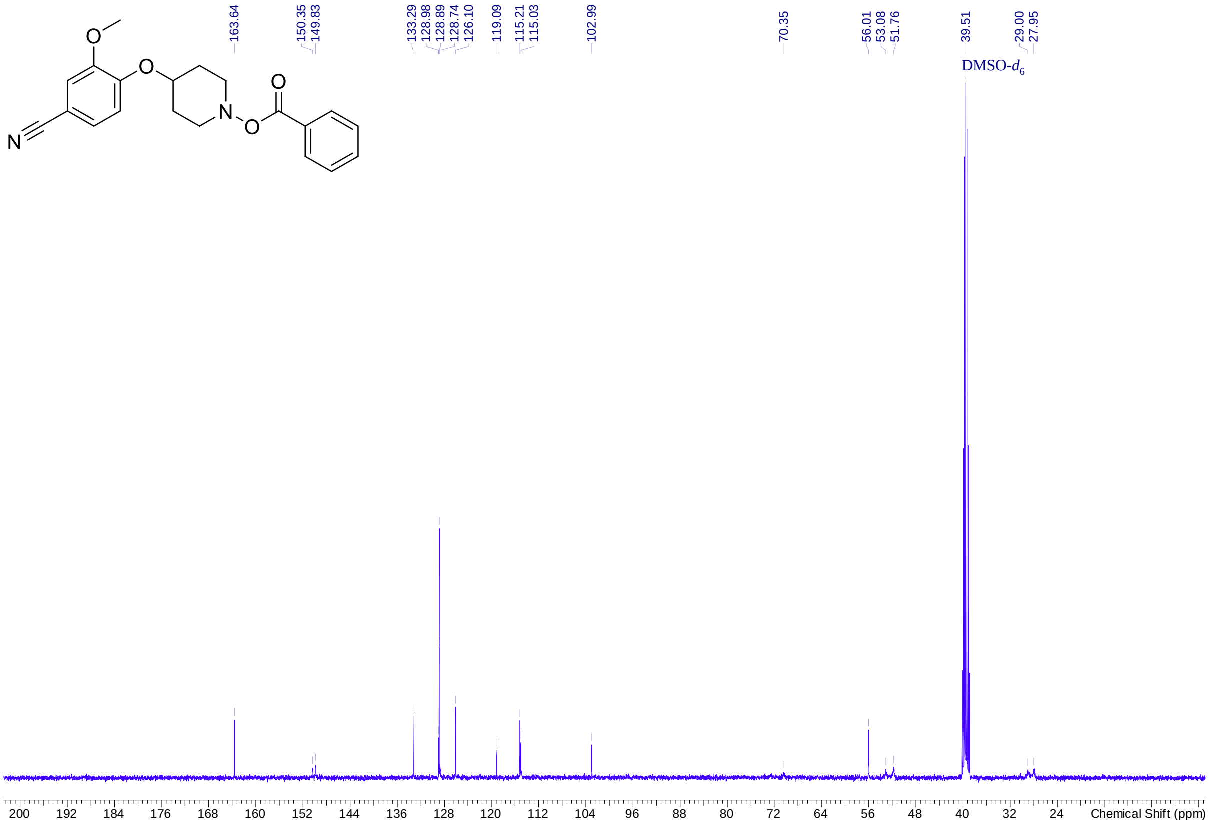

^{1}H NMR (400 MHz, DMSO-d_6) of Compound **1w**

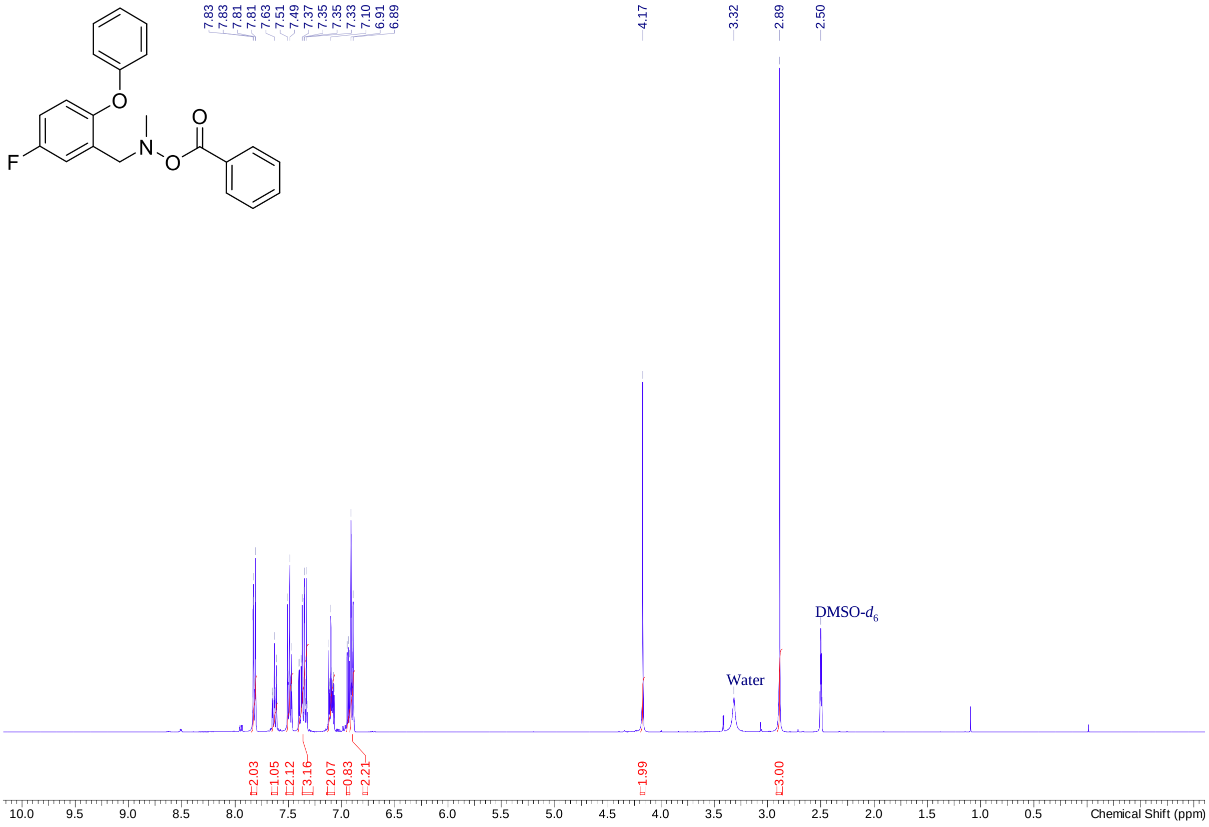

^{19}F{^{1}H} NMR (376 MHz, DMSO-d_6) of Compound **1w**

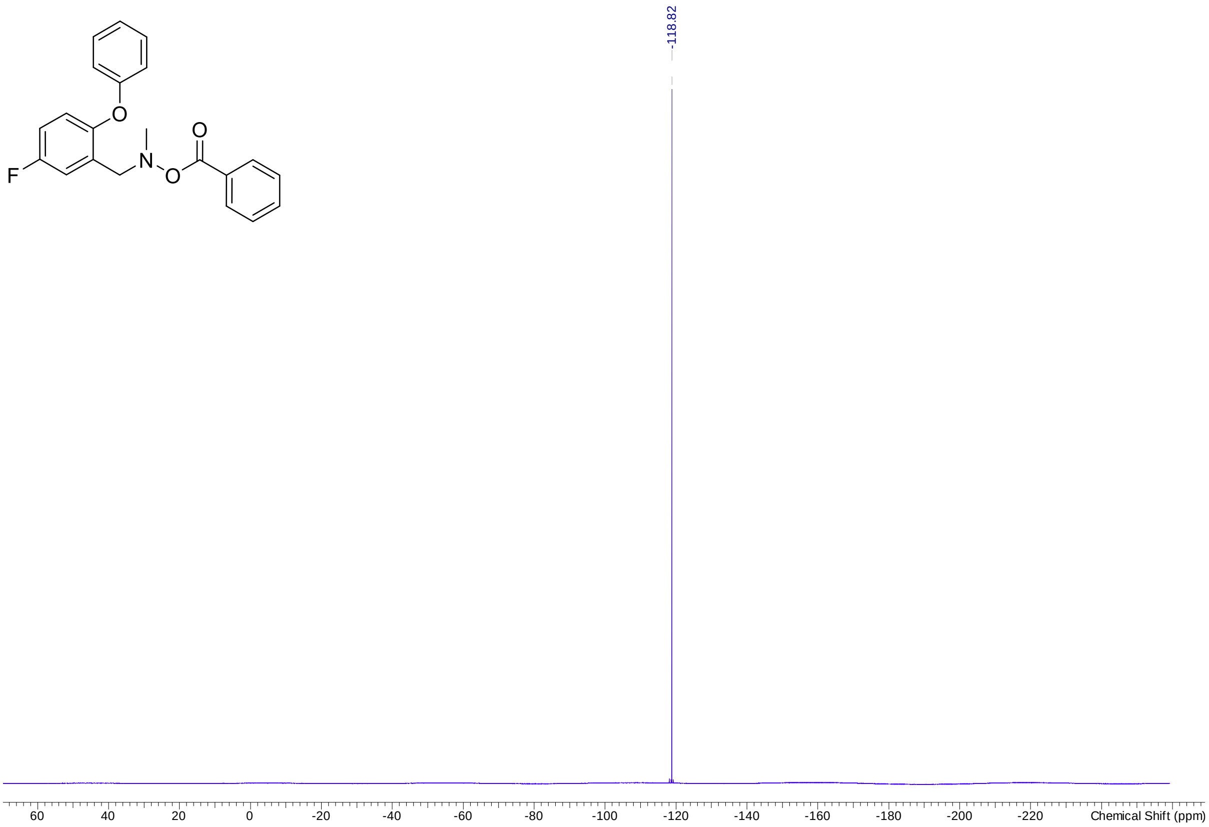

^{13}C NMR (101 MHz, DMSO-d_6) of Compound **1w**

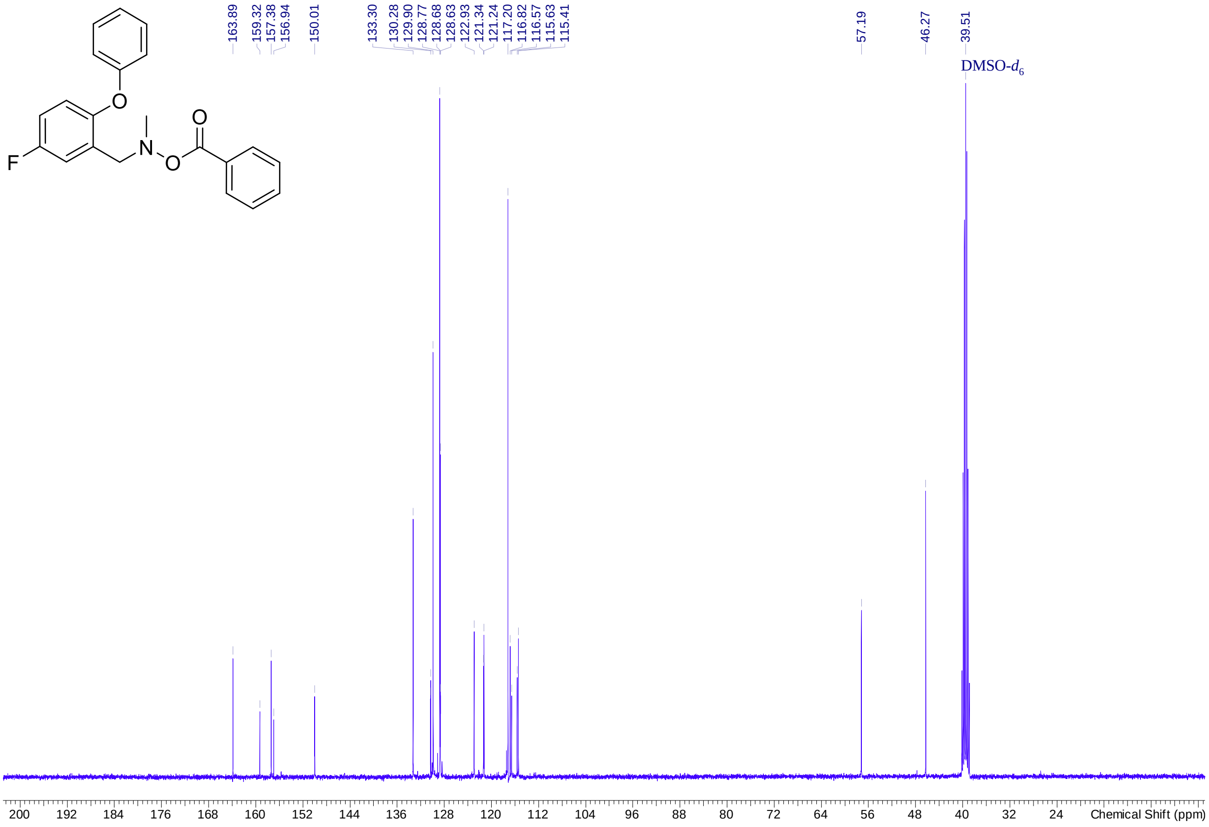

^{1}H NMR (400 MHz, DMSO-d_6) of Compound **1x**

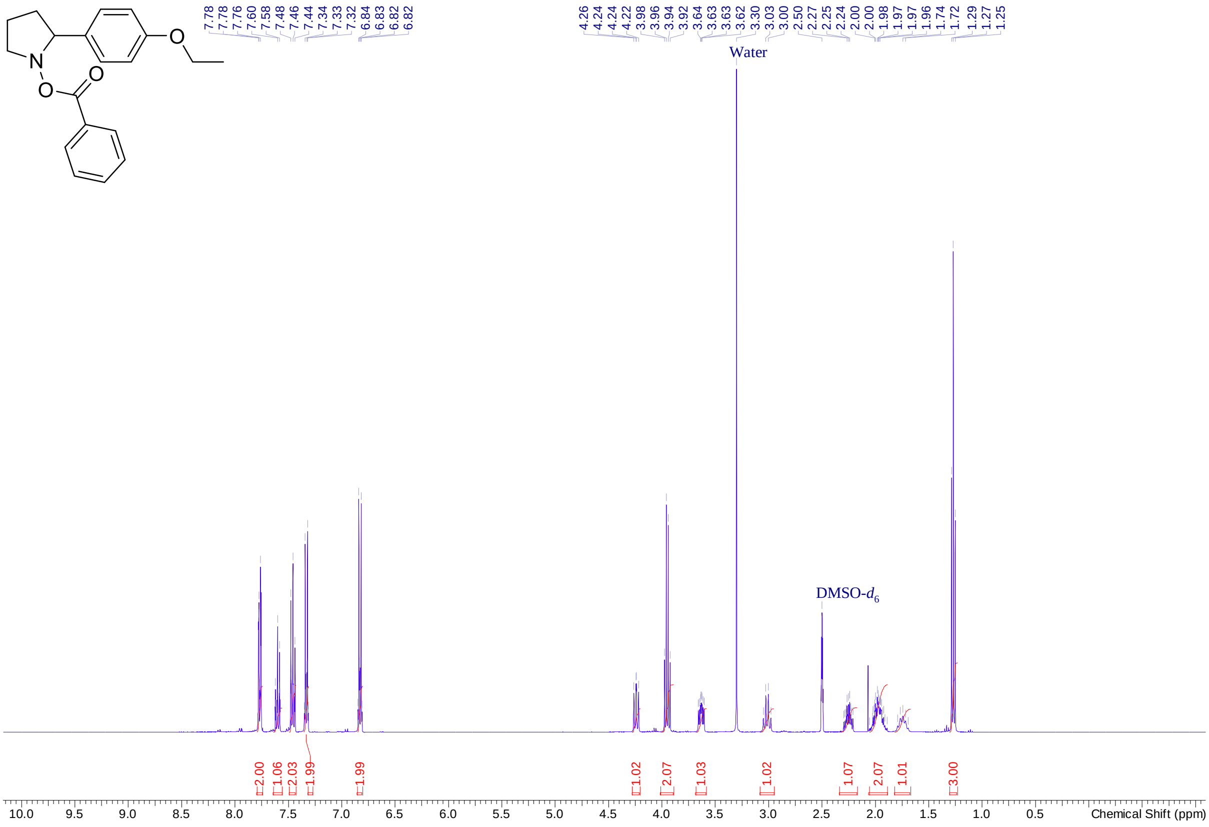

^{13}C NMR (101 MHz, DMSO-d_6) of Compound **1x**

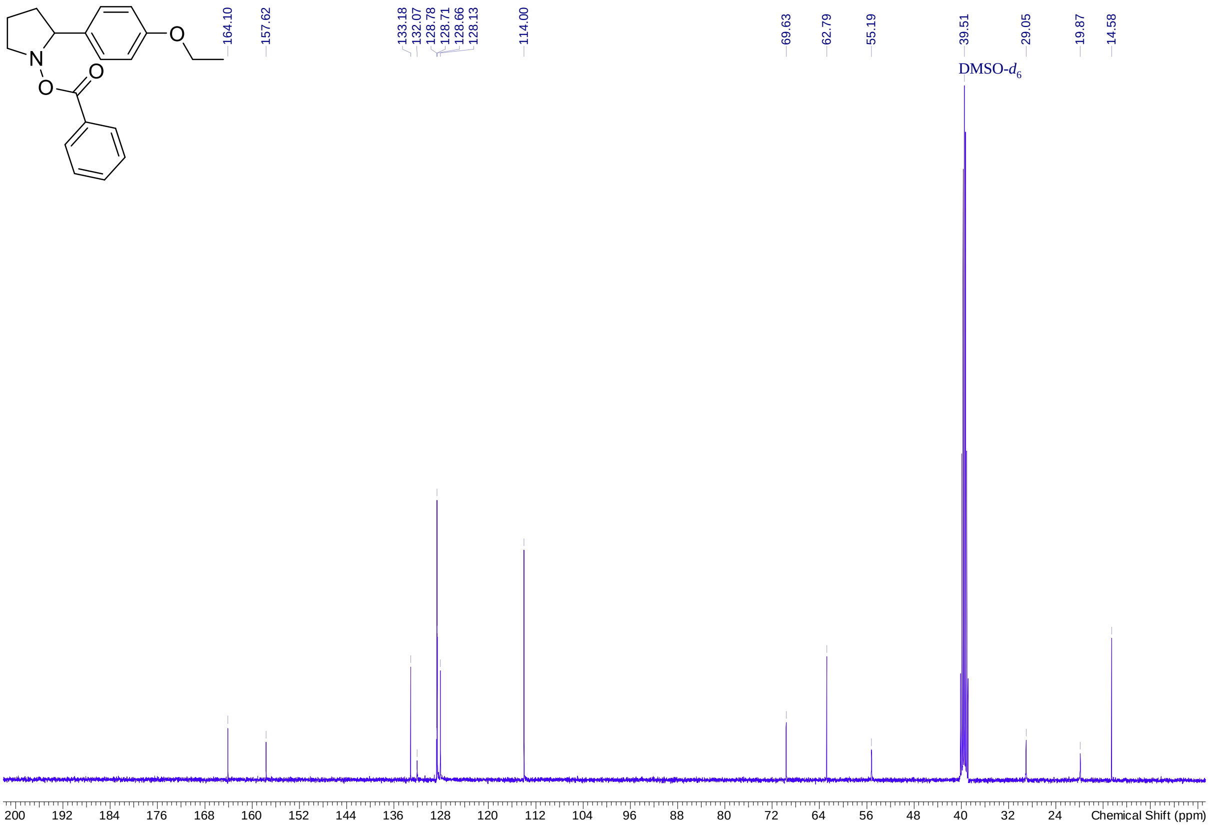

^{1}H NMR (600 MHz, DMSO-d_6) of Compound **S1a**

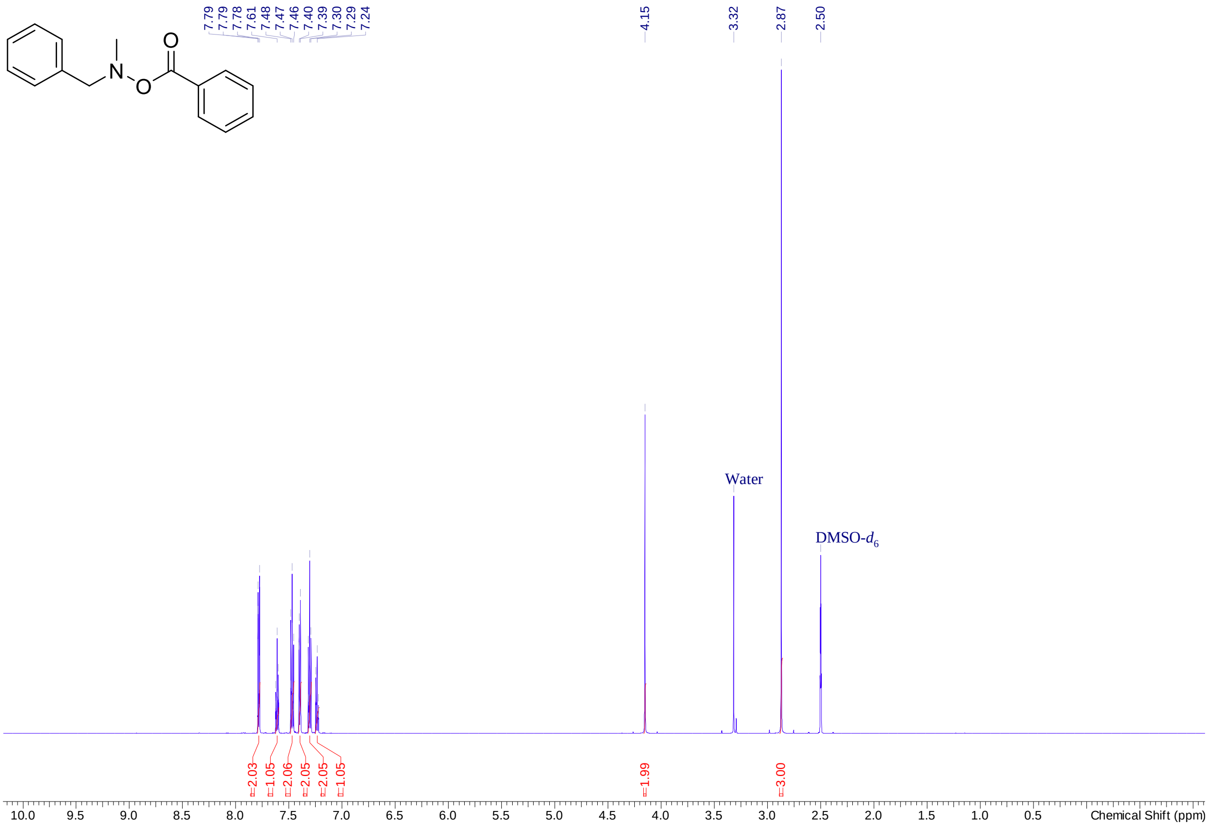

^{13}C NMR (151 MHz, DMSO-d_6) of Compound **S1a**

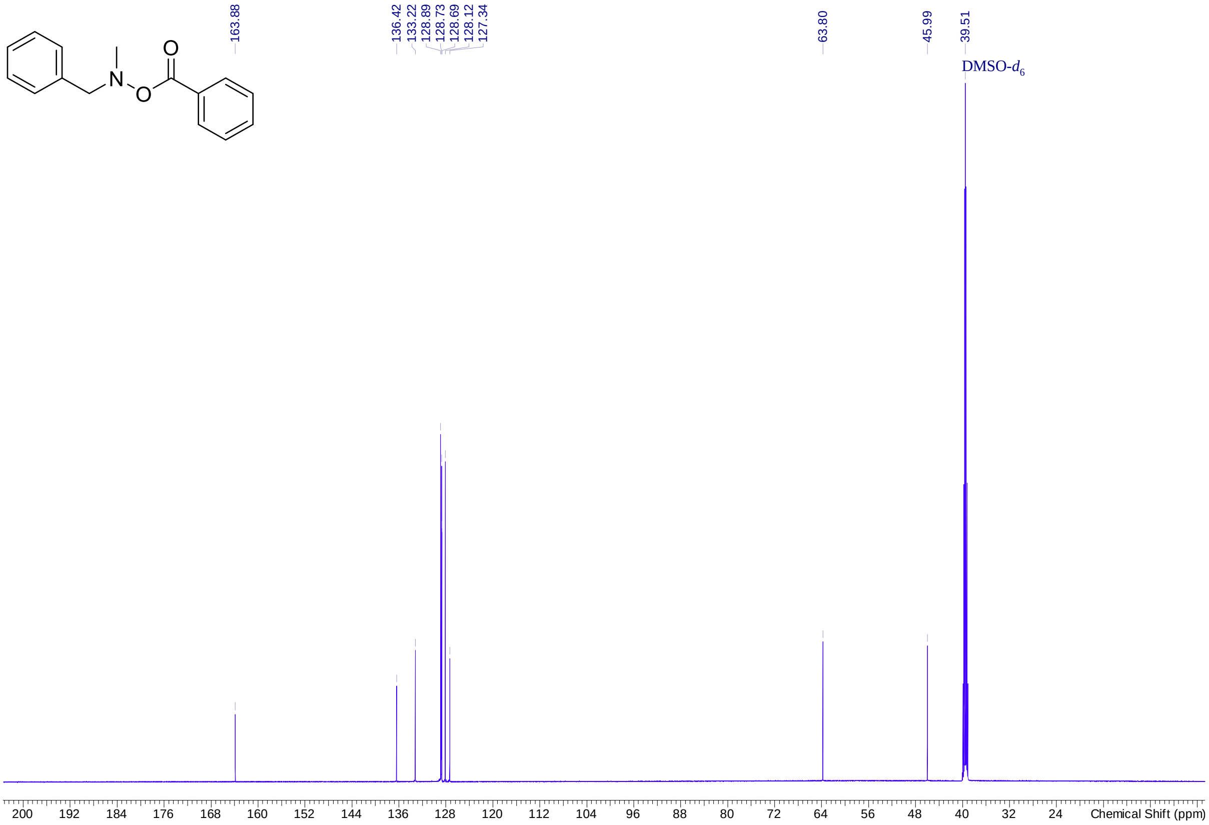

^{1}H NMR (400 MHz, DMSO-d_6) of Compound **S1b**

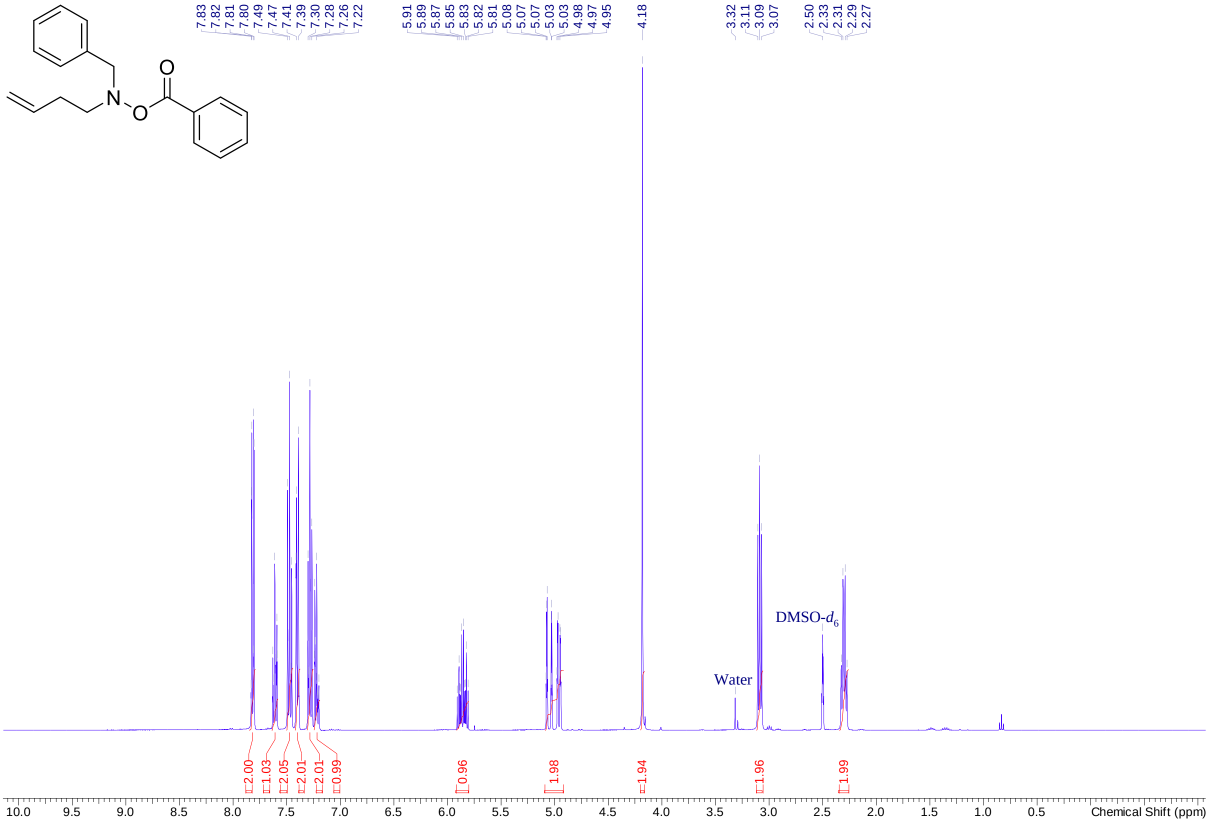

^{13}C NMR (101 MHz, DMSO-d_6) of Compound **S1b**

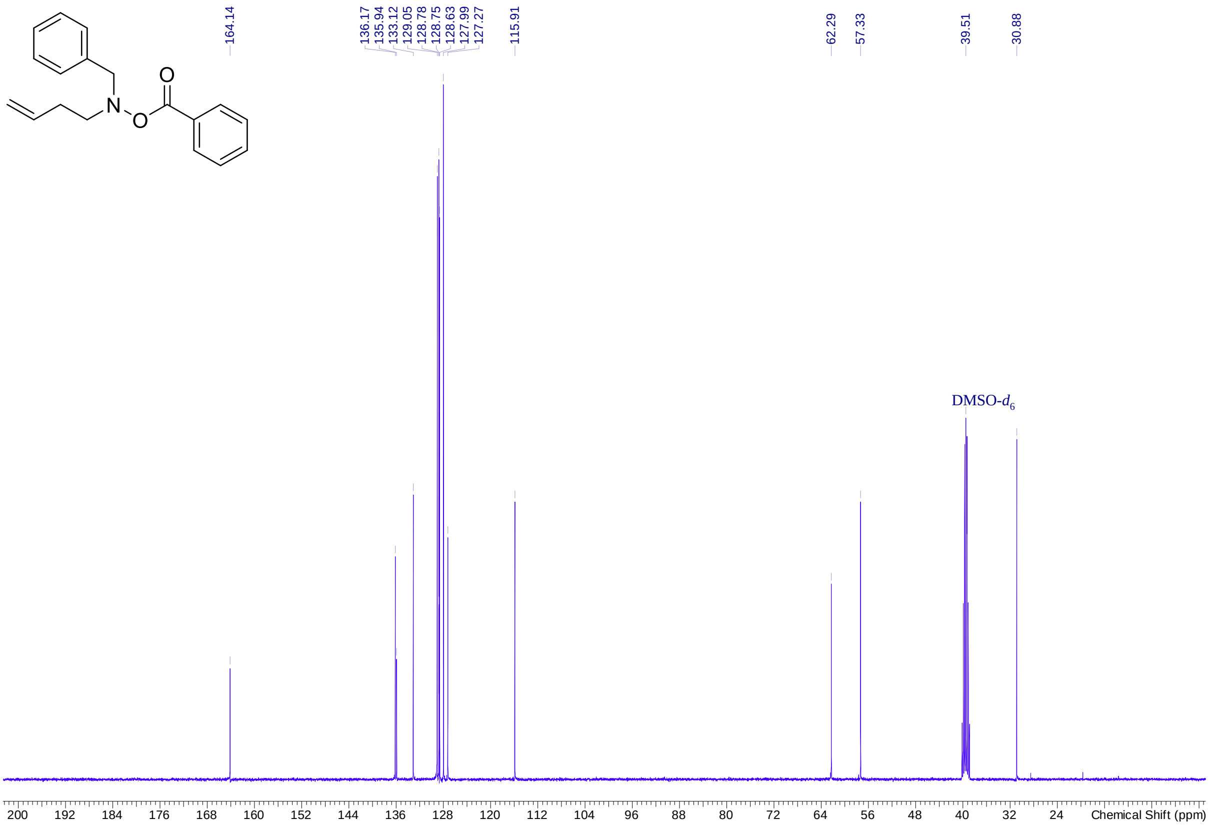

^{1}H NMR (400 MHz, DMSO-d_6) of Compound **S1c**

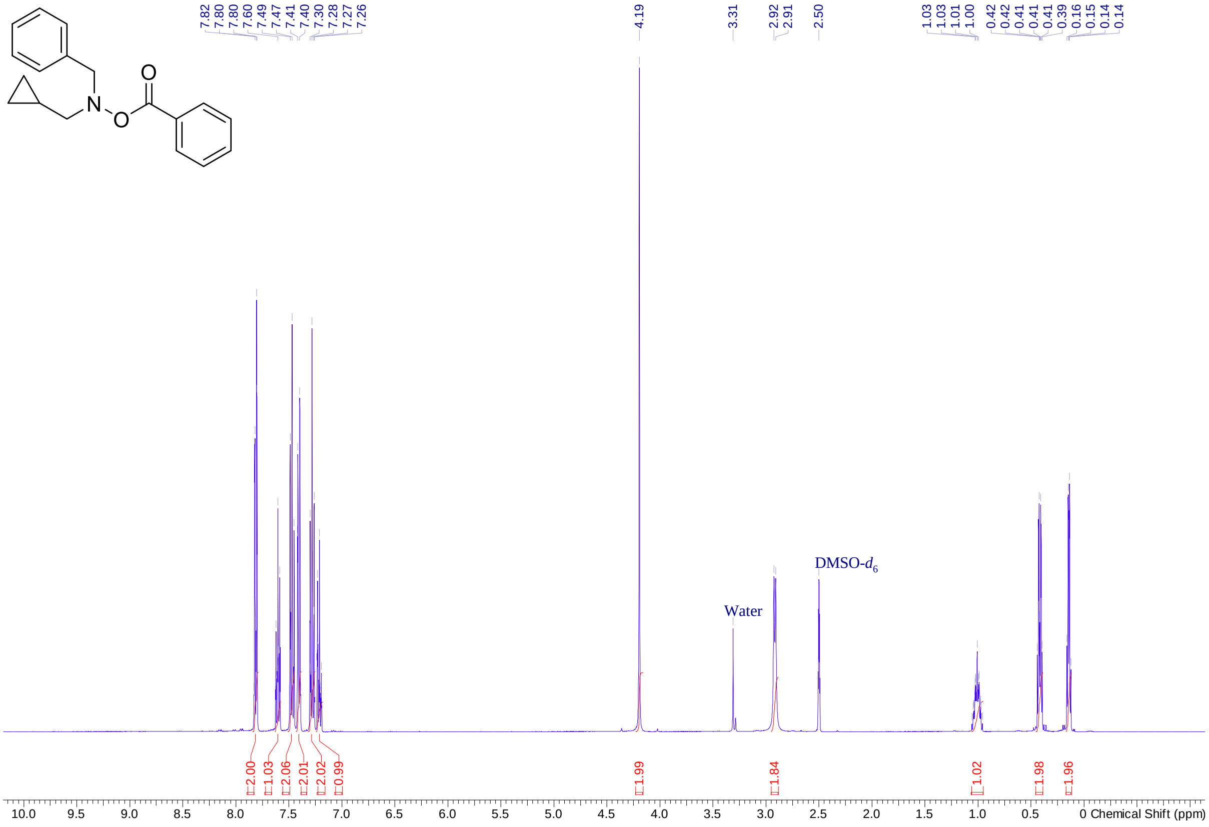

^{13}C NMR (101 MHz, DMSO-d_6) of Compound **S1c**

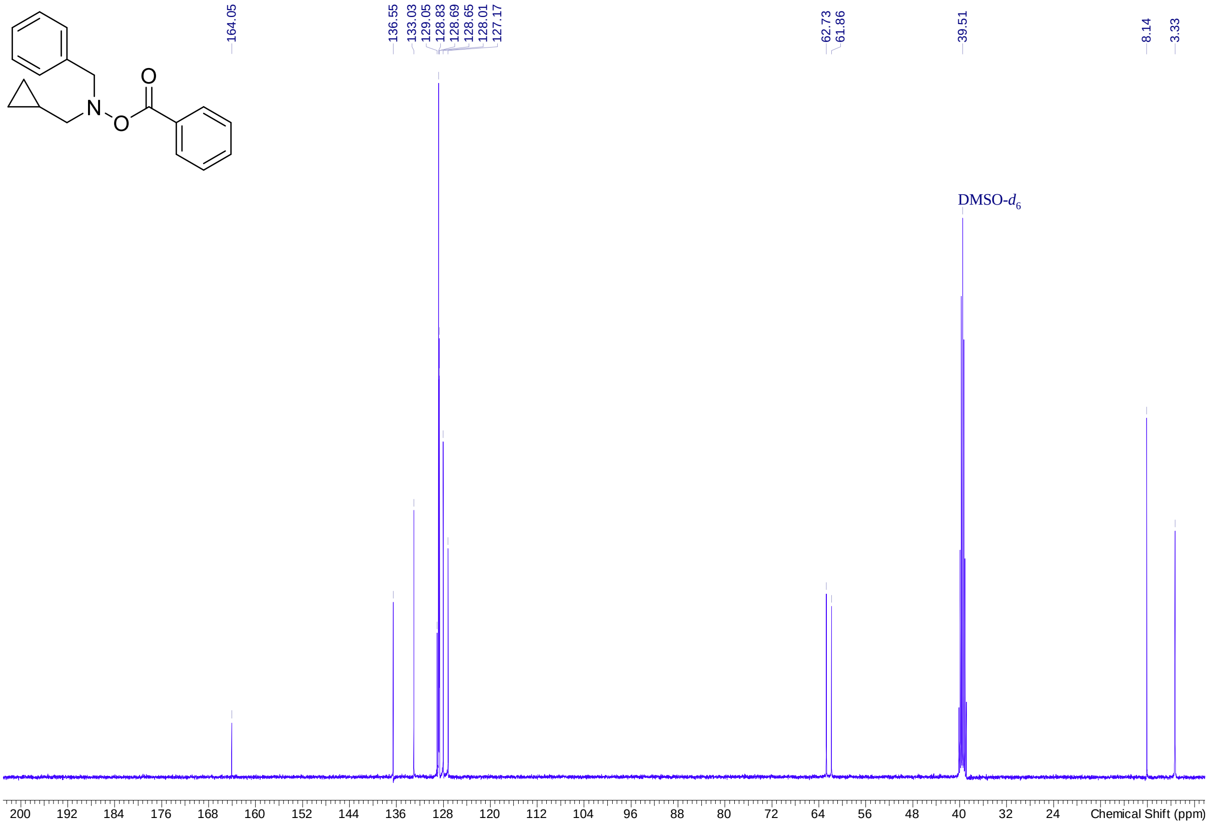

^{1}H NMR (400 MHz, DMSO-d_6) of Compound **S1d**

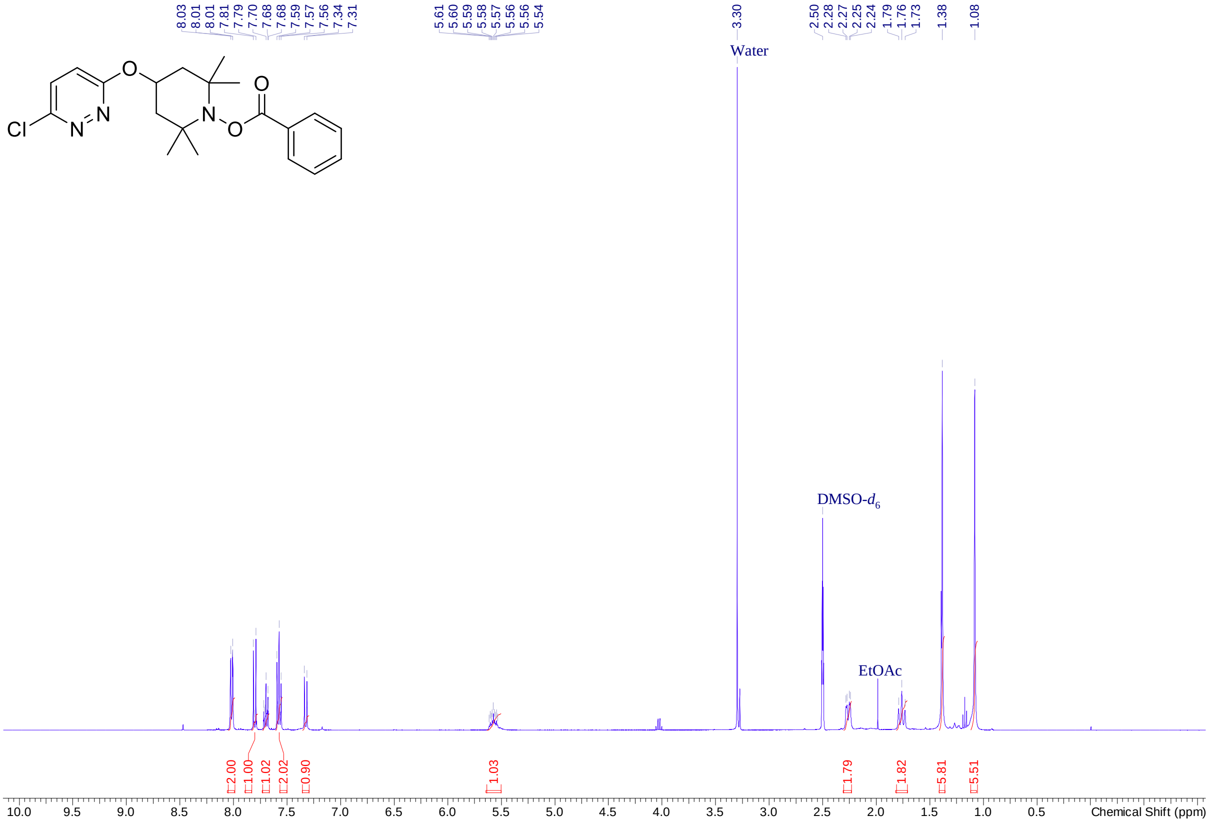

^{13}C NMR (101 MHz, DMSO-d_6) of Compound **S1d**

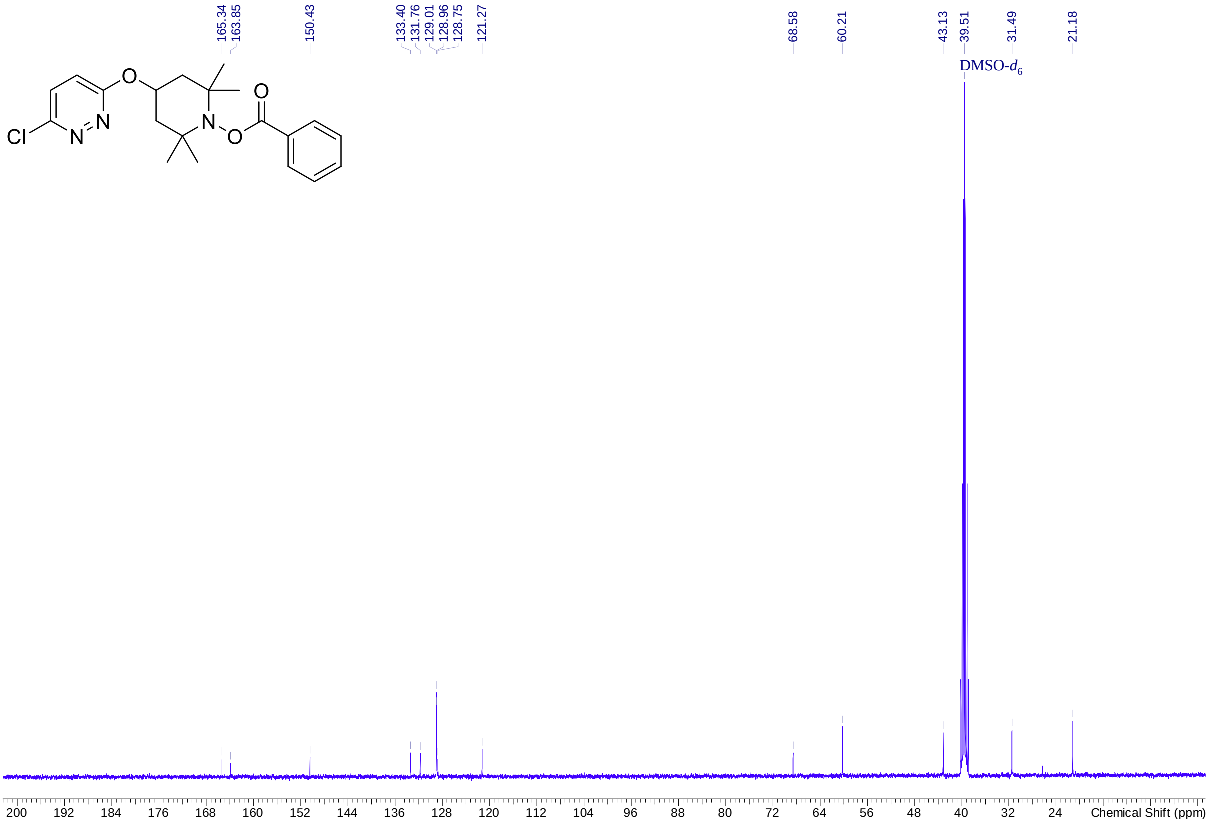

^{1}H NMR (600 MHz, DMSO-d_6) of Compound **S1e**

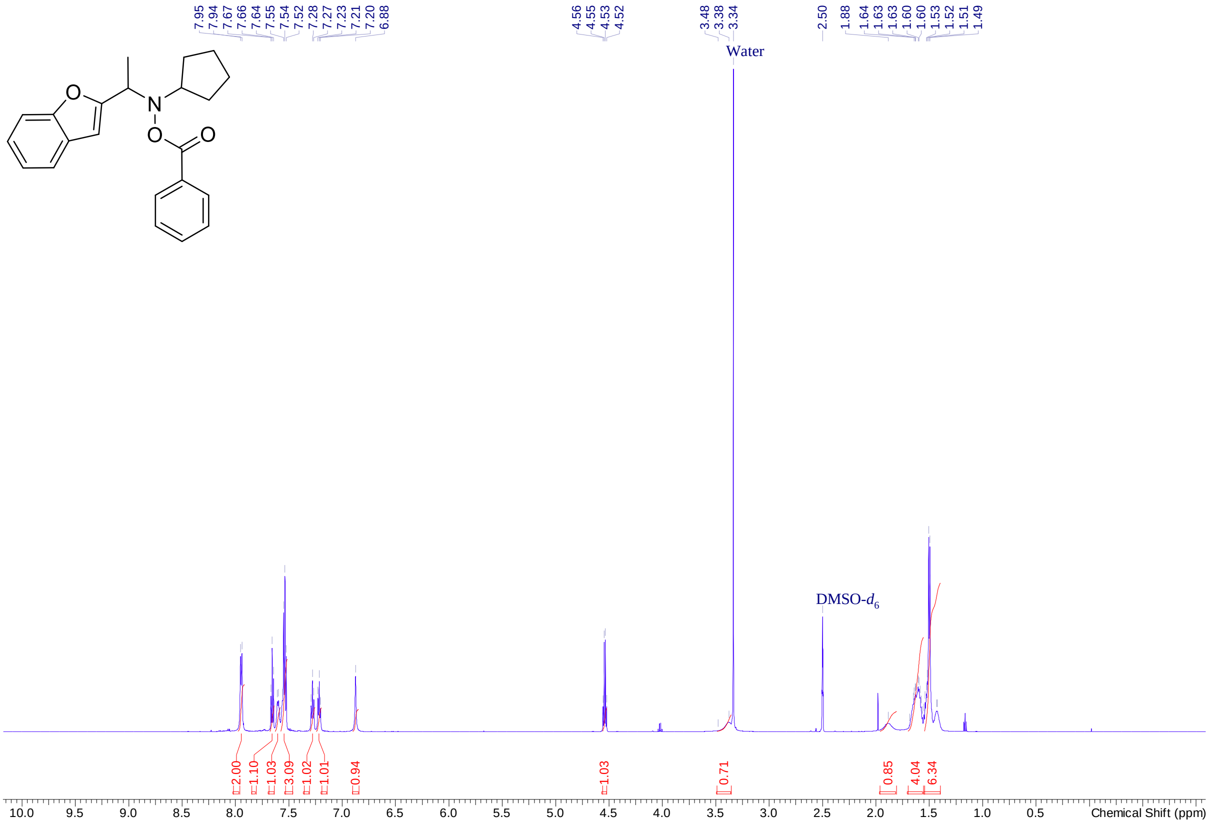

^{13}C NMR (151 MHz, DMSO-d_6) of Compound **S1e**

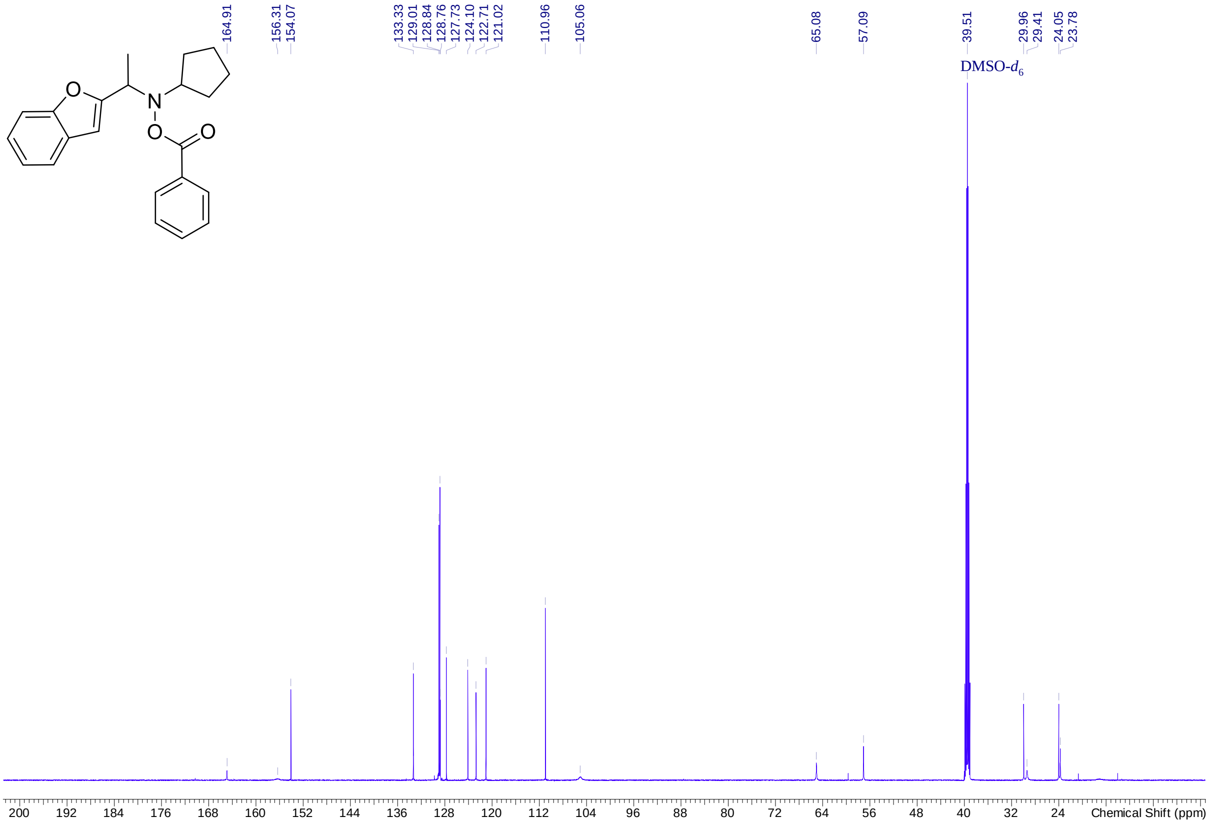

^{1}H NMR (400 MHz, DMSO-d_6) of Compound **S1f**

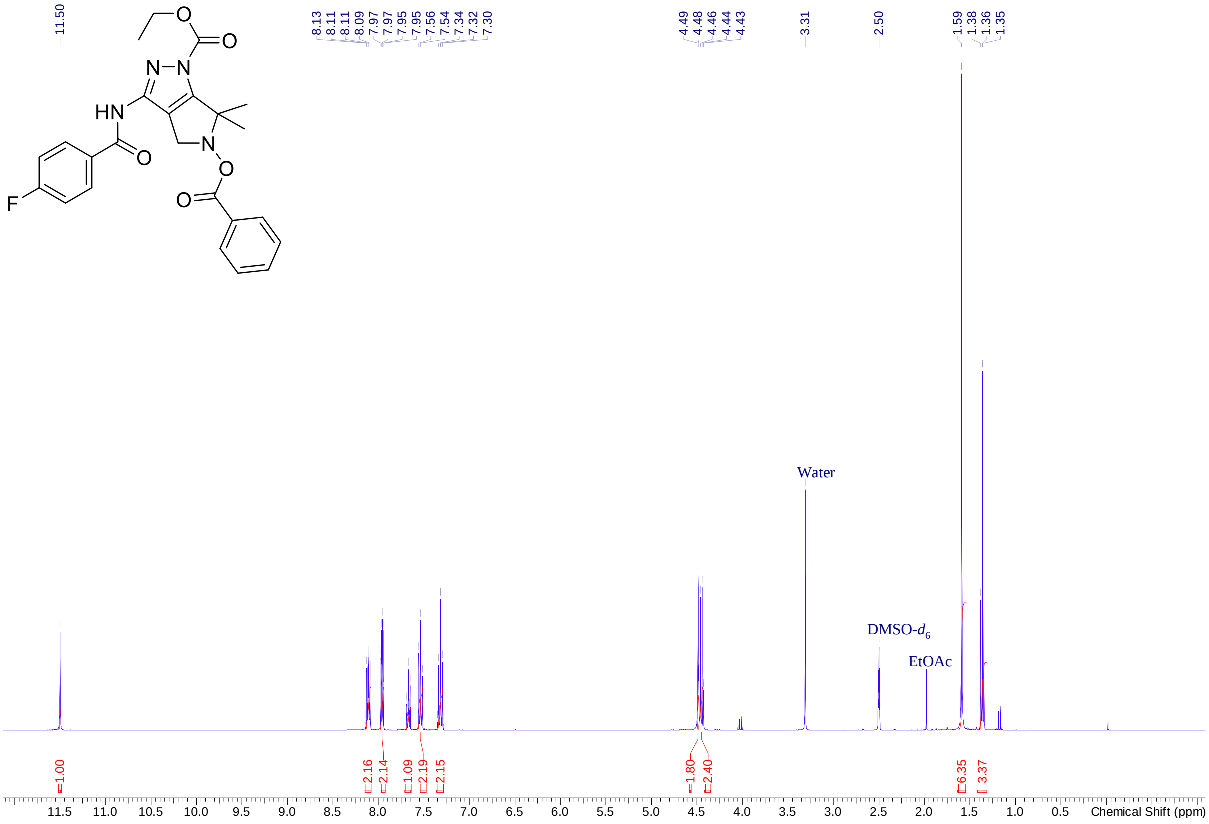

$^{19}F\{^1H\}$ NMR (376 MHz, DMSO-d_6) of Compound **S1f**

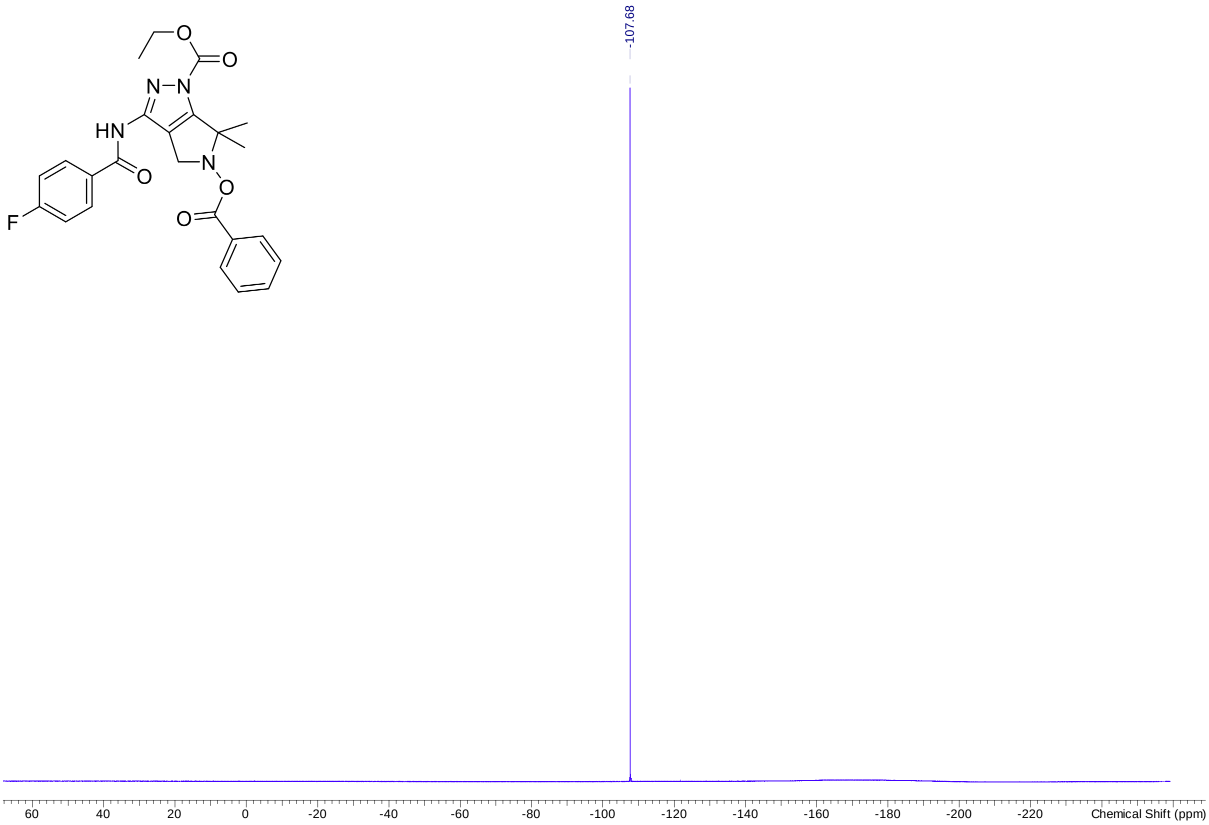

^{13}C NMR (101 MHz, DMSO-d_6) of Compound **S1f**

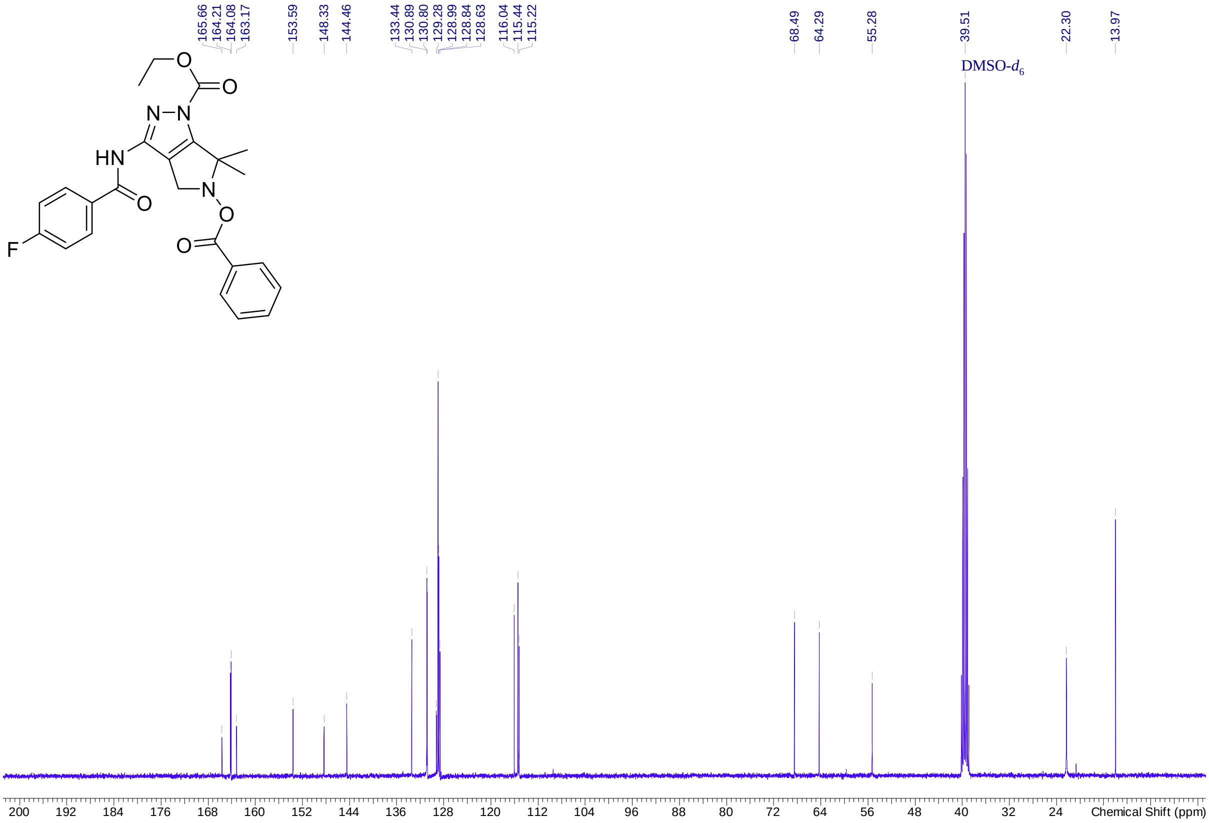

^{1}H NMR (600 MHz, DMSO-d_6) of Compound **S2a**

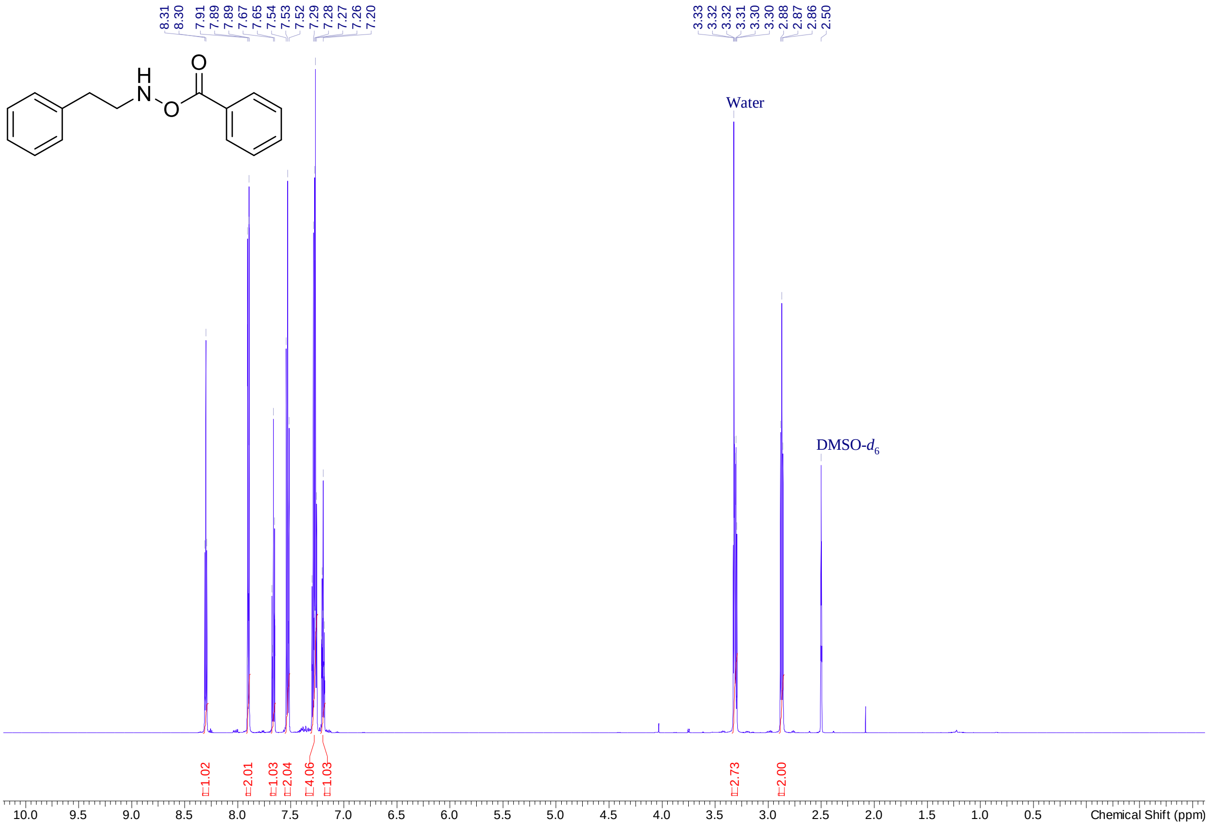

^{13}C NMR (151 MHz, DMSO-d_6) of Compound **S2a**

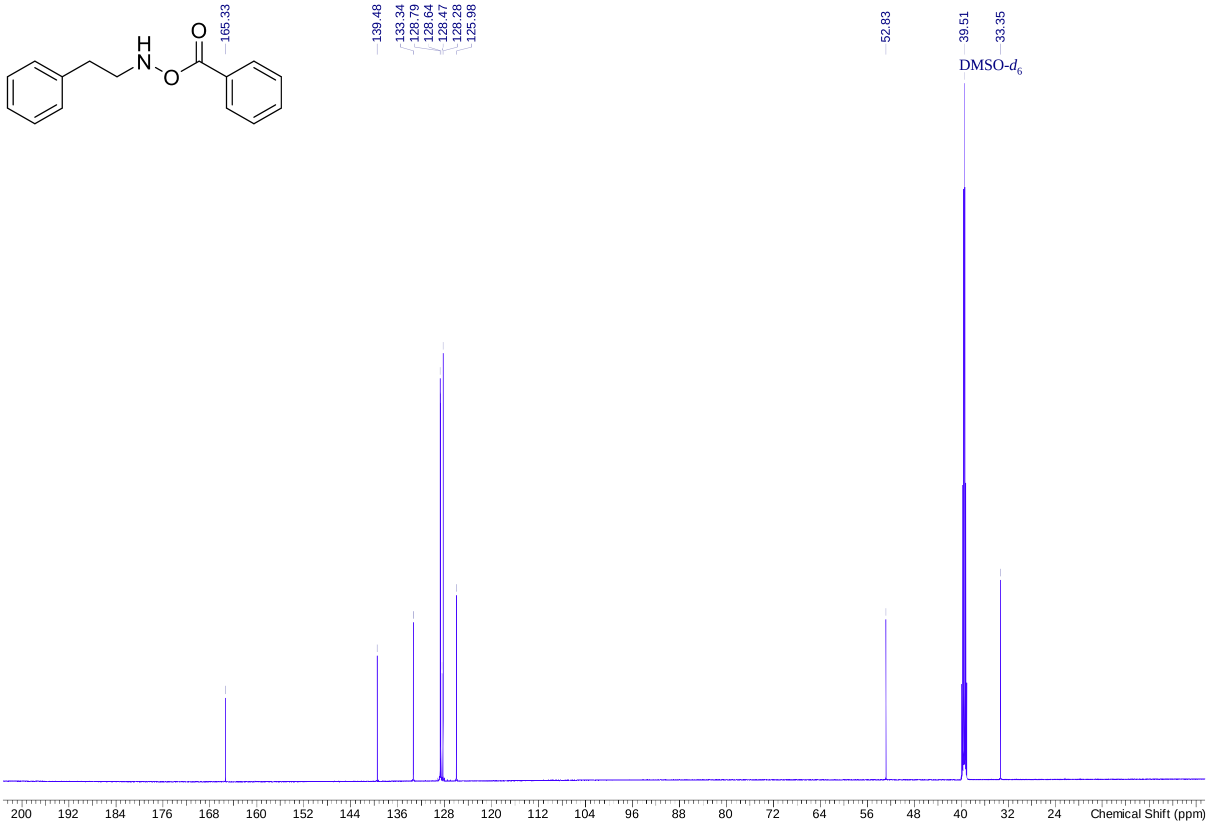

^{1}H NMR (600 MHz, DMSO-d_6) of Compound **S2b**

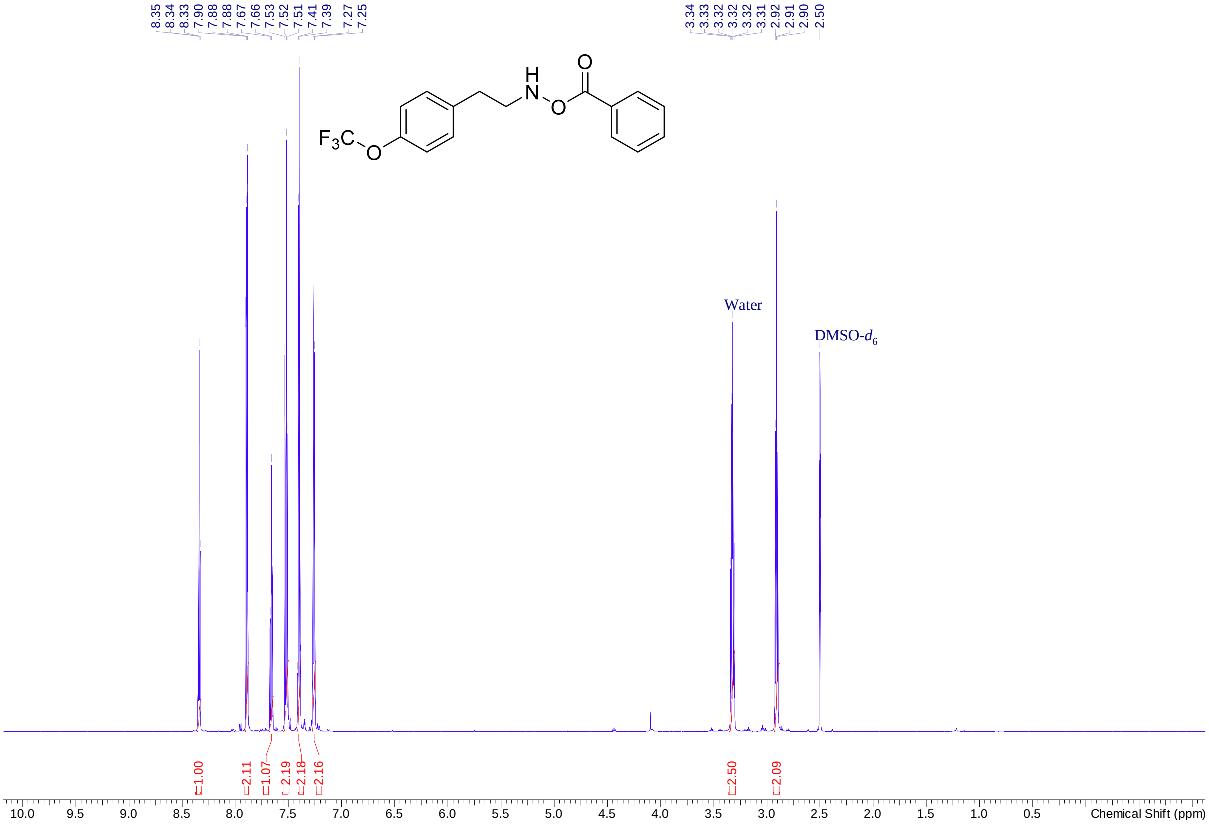

^{19}F NMR (376 MHz, DMSO-d_6) of Compound **S2b**

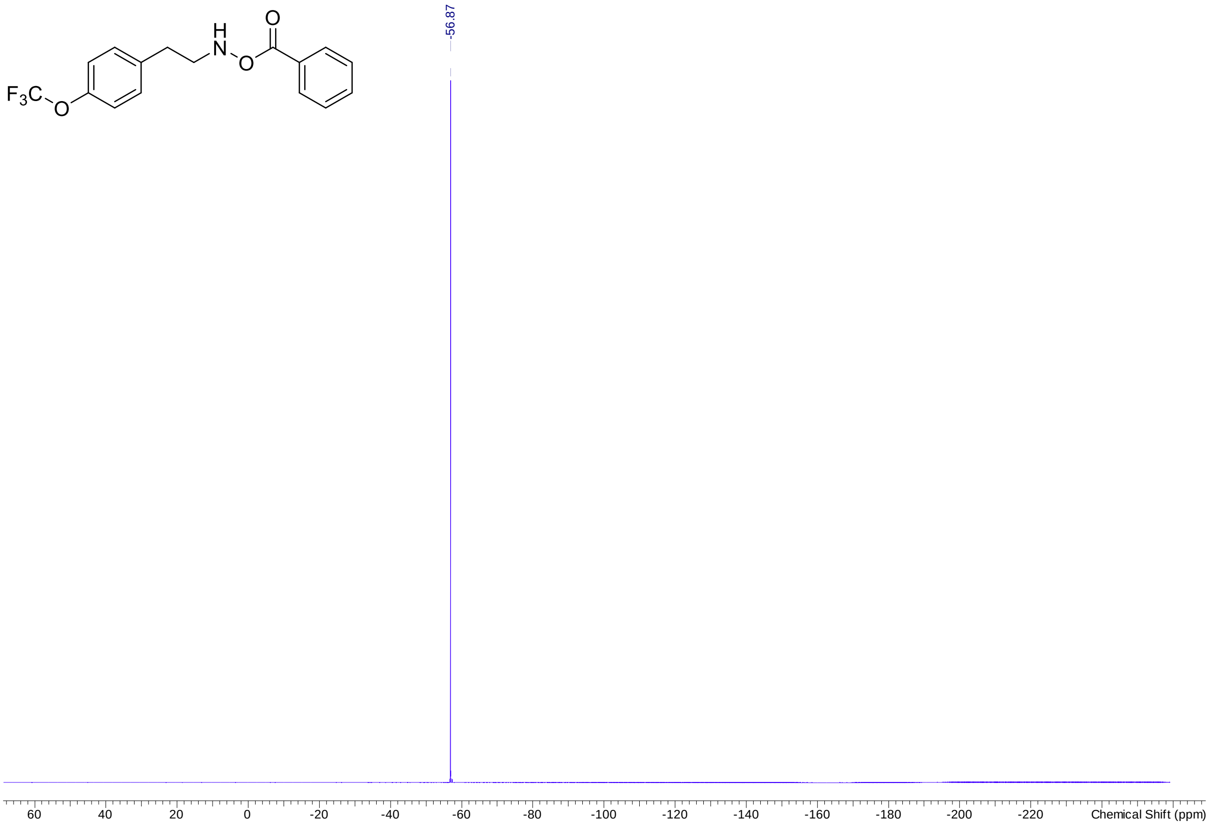

^{13}C NMR (151 MHz, DMSO-d_6) of Compound **S2b**

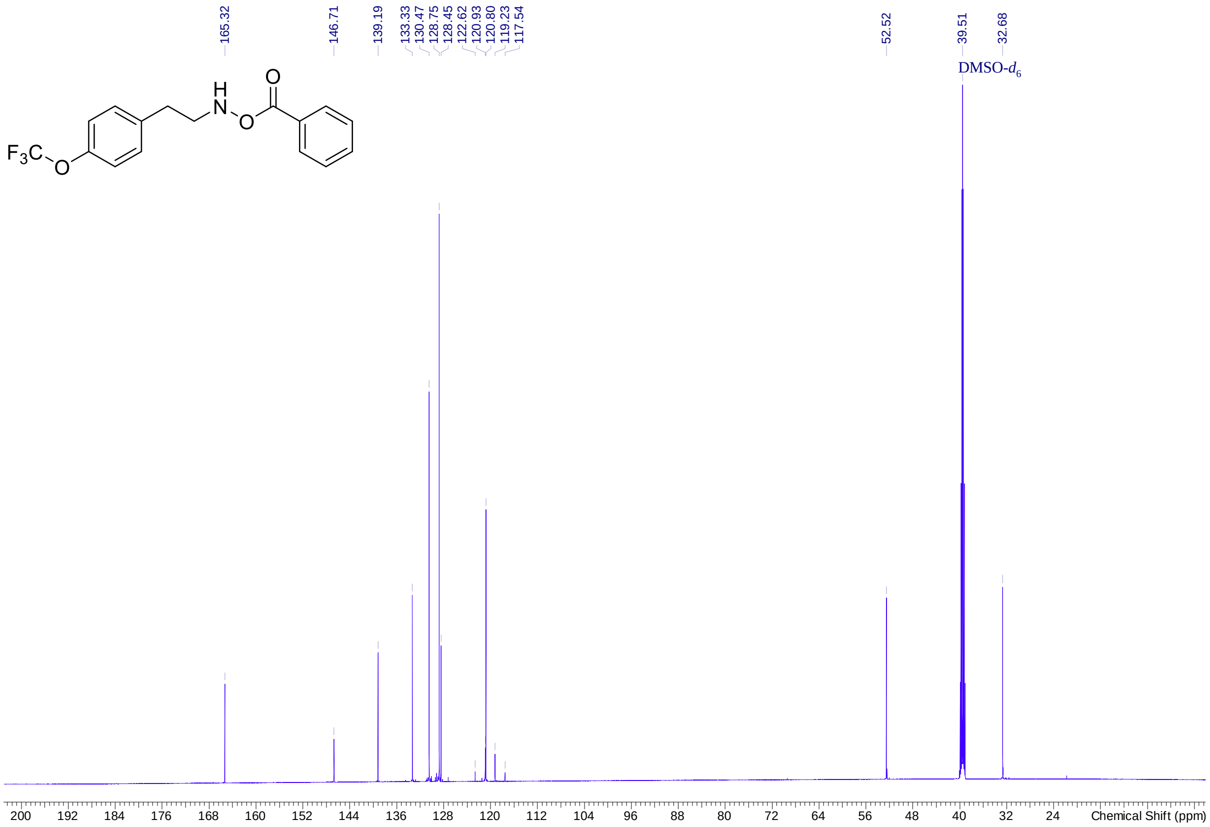

^1H NMR (400 MHz, DMSO-d_6) of Compound **S2c**

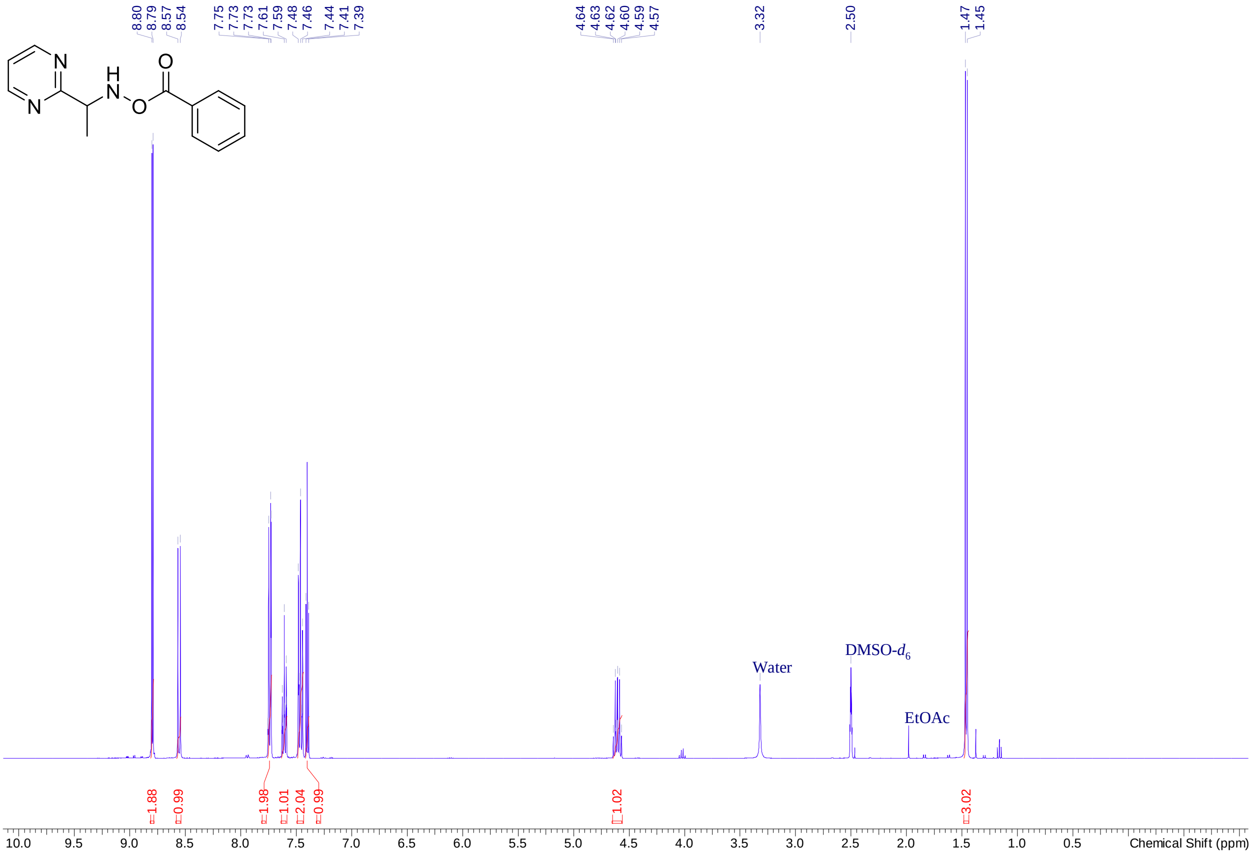

^{13}C NMR (101 MHz, DMSO-d_6) of Compound **S2c**

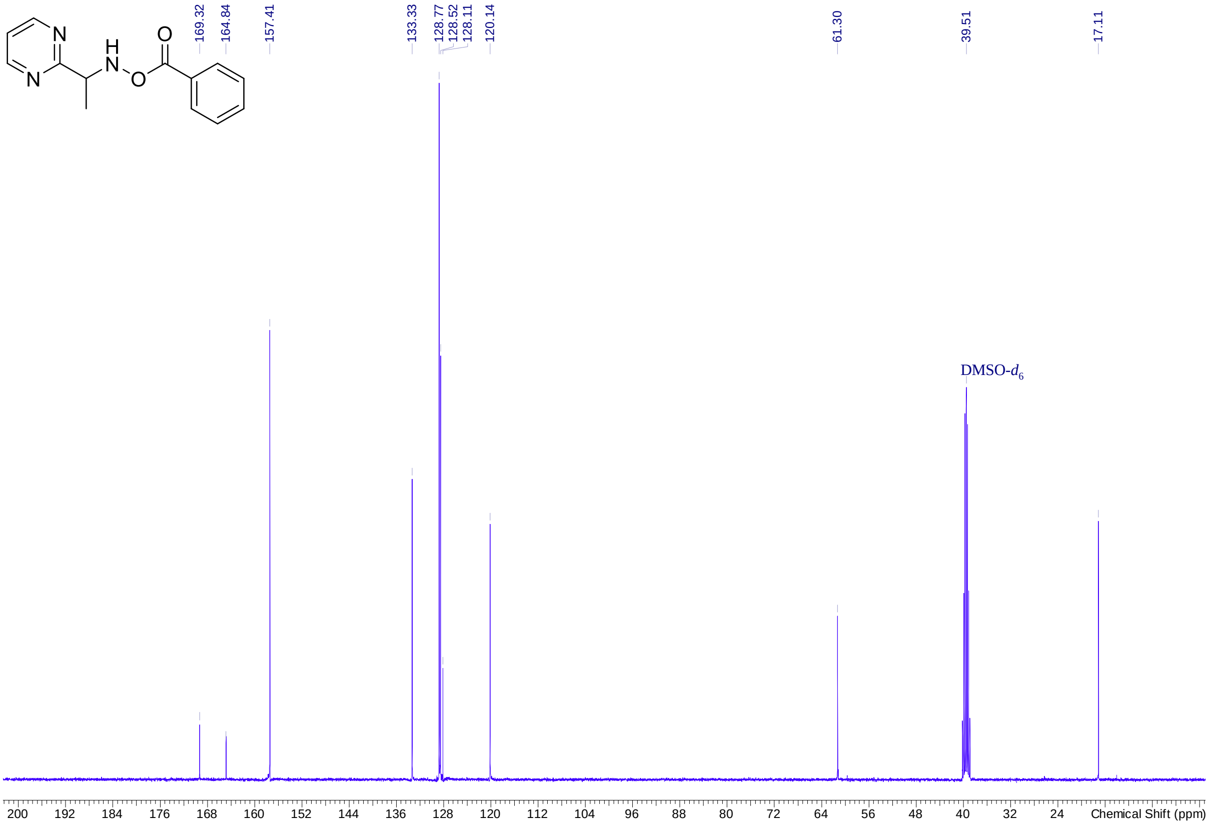

^{1}H NMR (600 MHz, DMSO-d_6) of Compound **S2d**

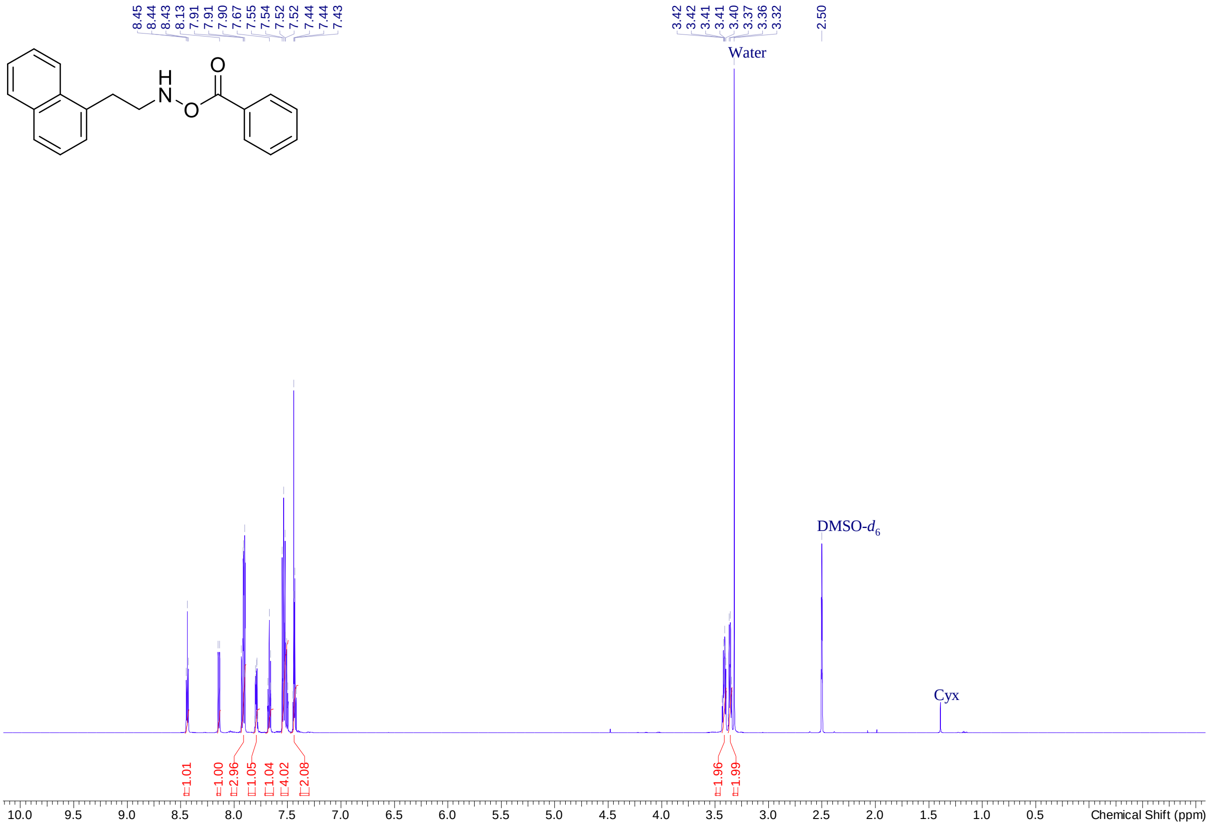

^{13}C NMR (151 MHz, DMSO-d_6) of Compound **S2d**

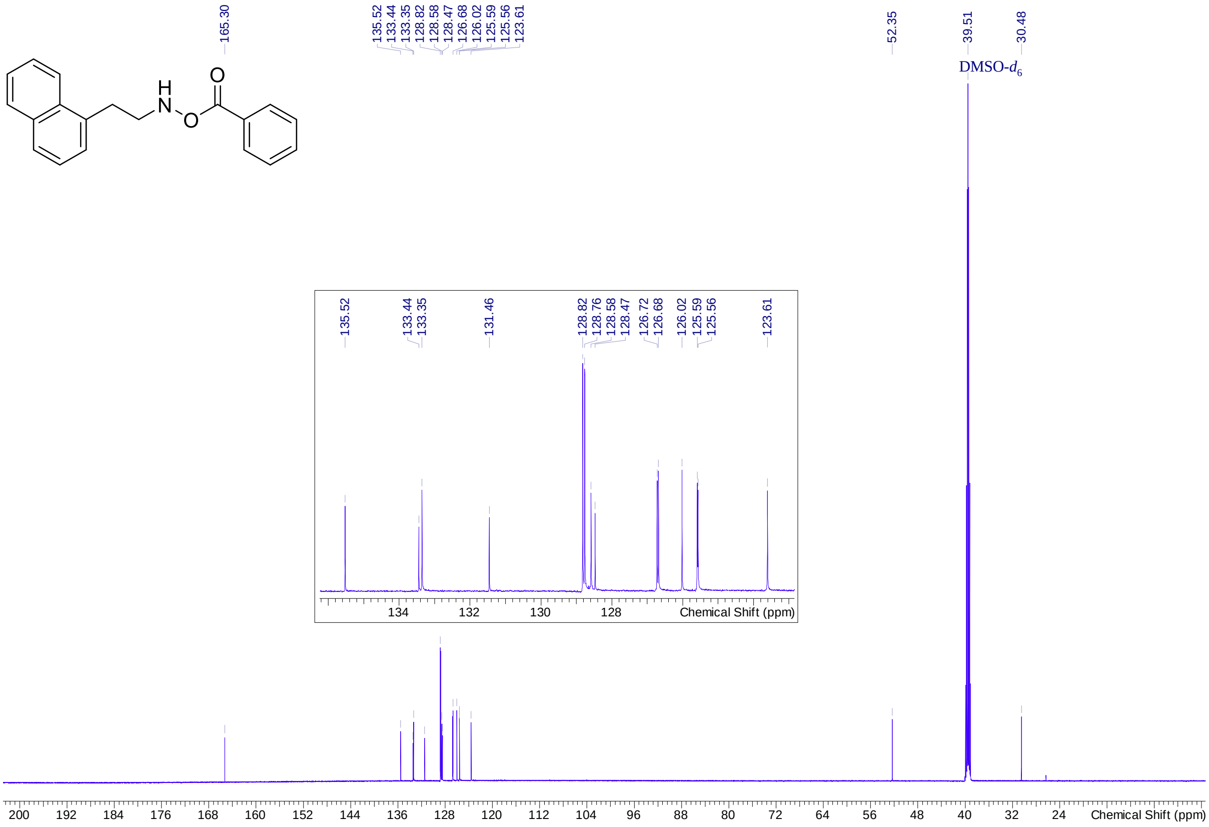

^{1}H NMR (400 MHz, DMSO-d_6) of Compound **S2e**

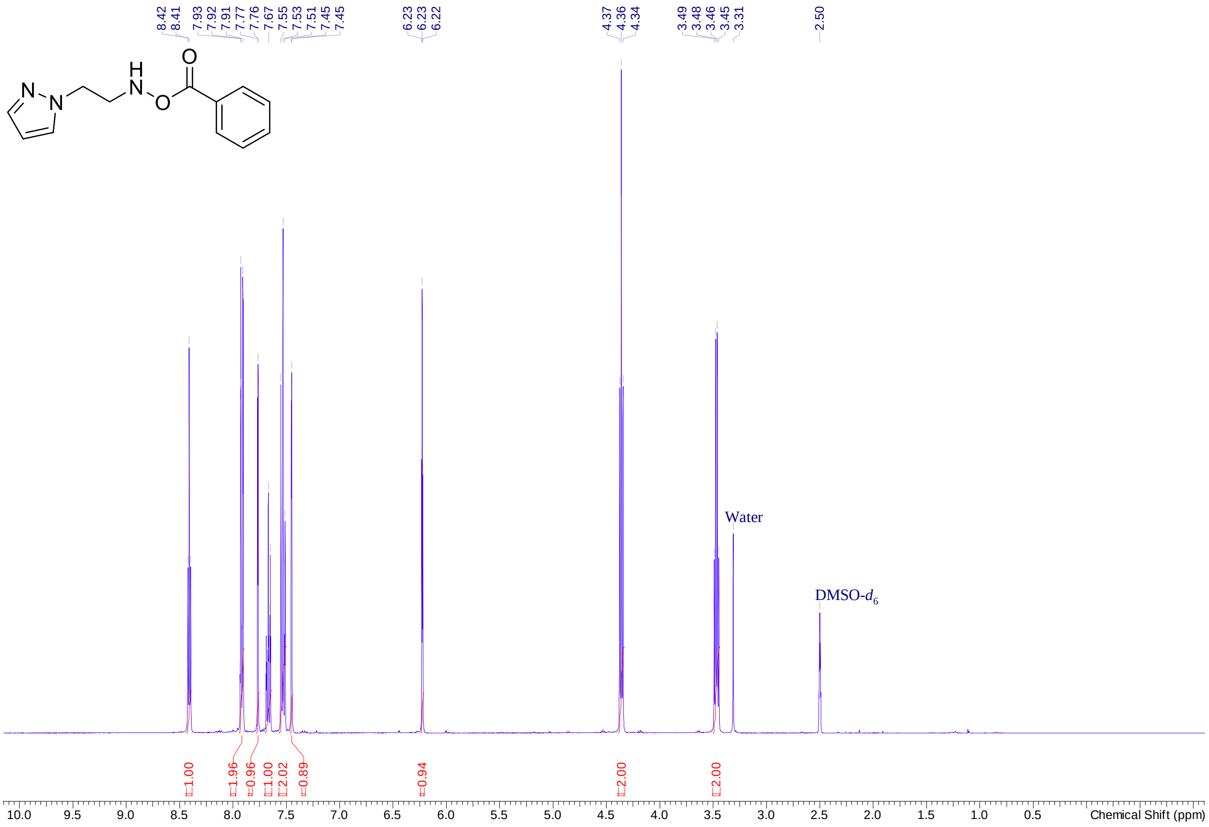

^{13}C NMR (101 MHz, DMSO-d_6) of Compound **S2e**

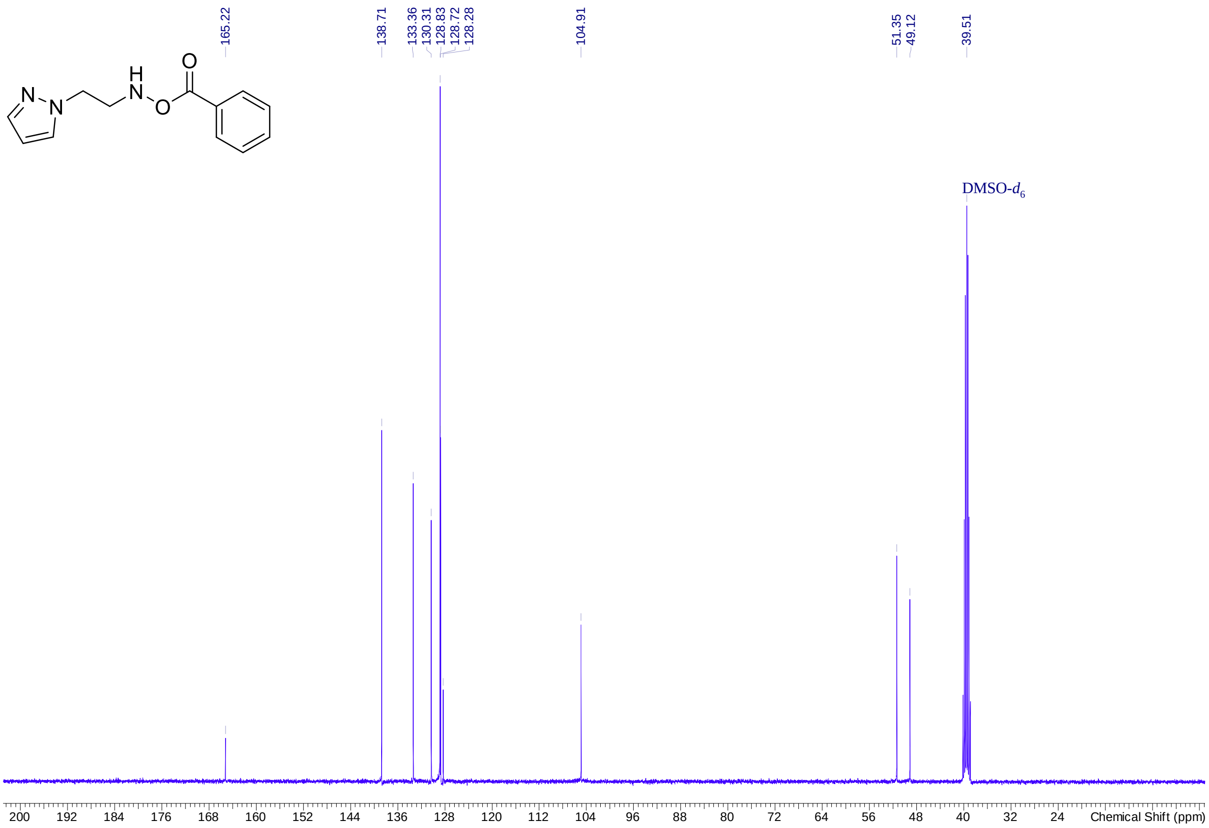

^{1}H NMR (600 MHz, DMSO-d_6) of Compound **S3a**

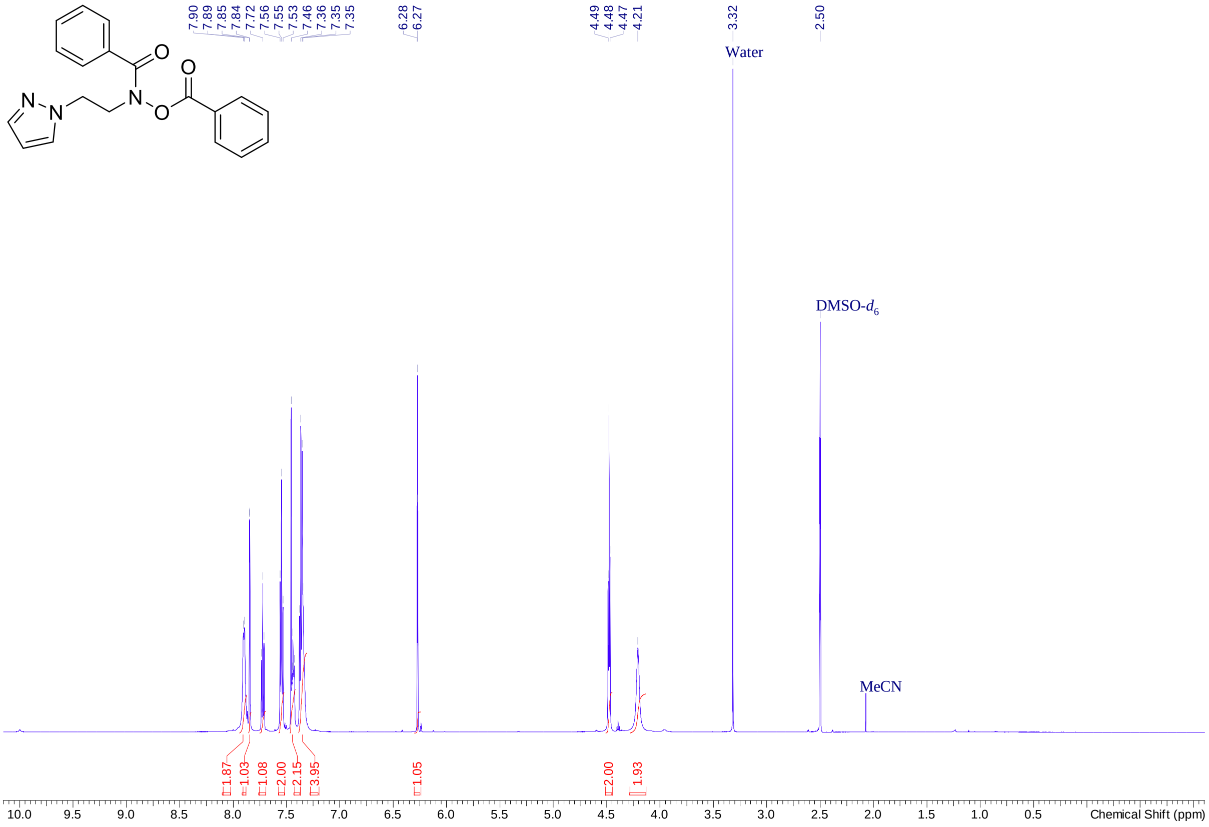

^{13}C NMR (151 MHz, DMSO-d_6) of Compound **S3a**

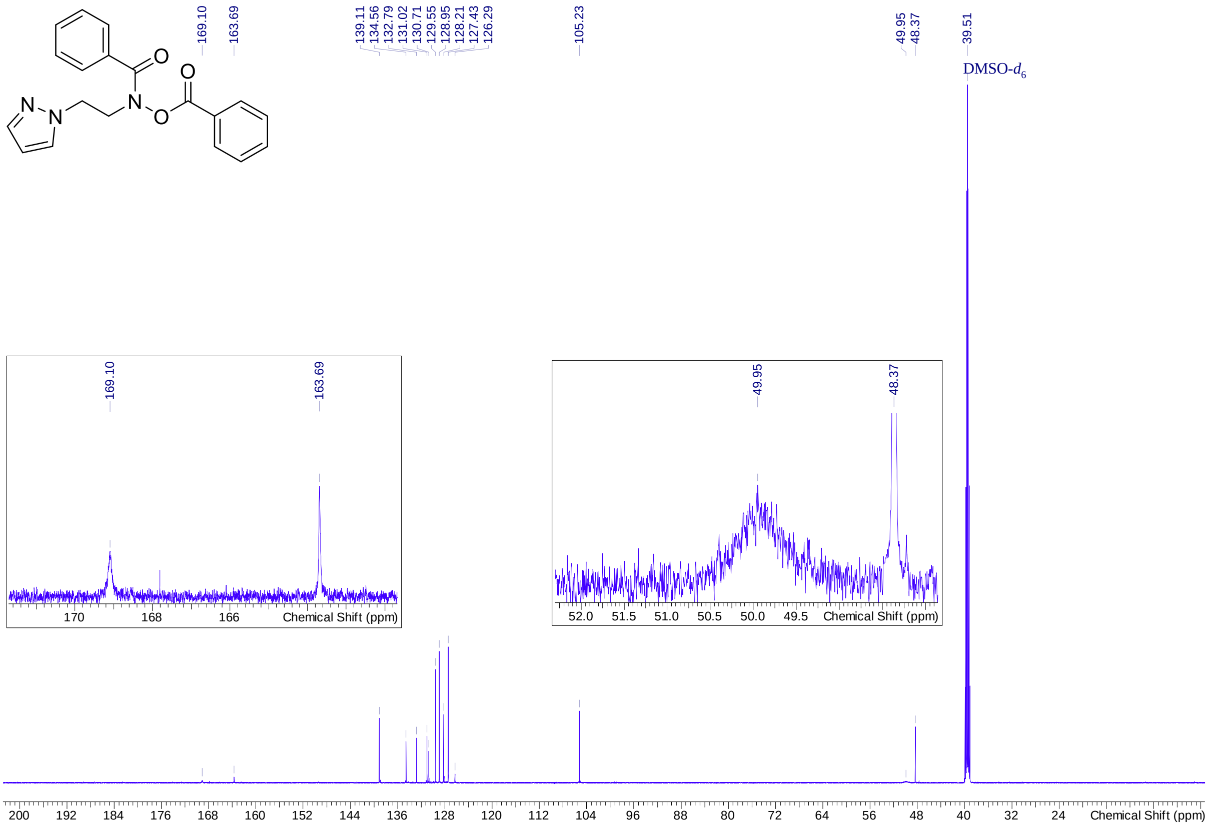

^{1}H NMR (400 MHz, DMSO-d_6) of Compound **S3b**

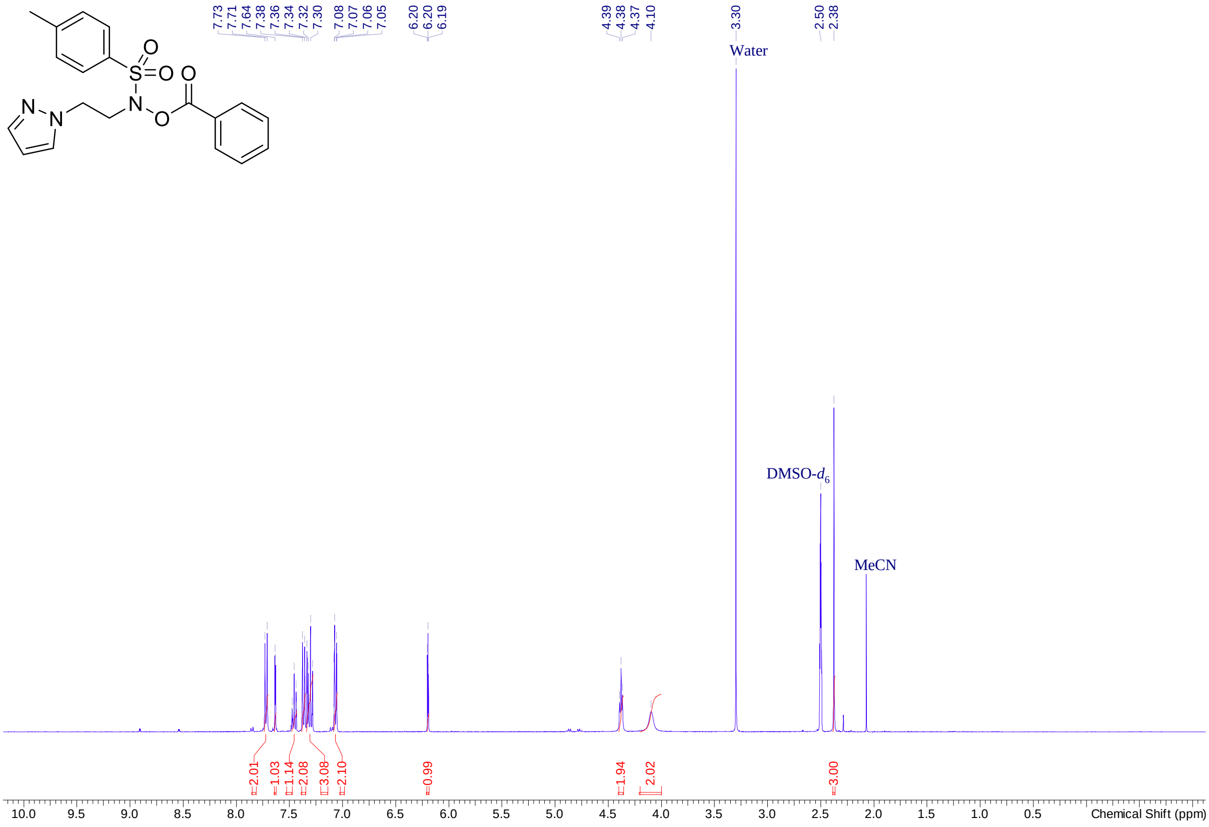

^{13}C NMR (101 MHz, DMSO-d_6) of Compound **S3b**

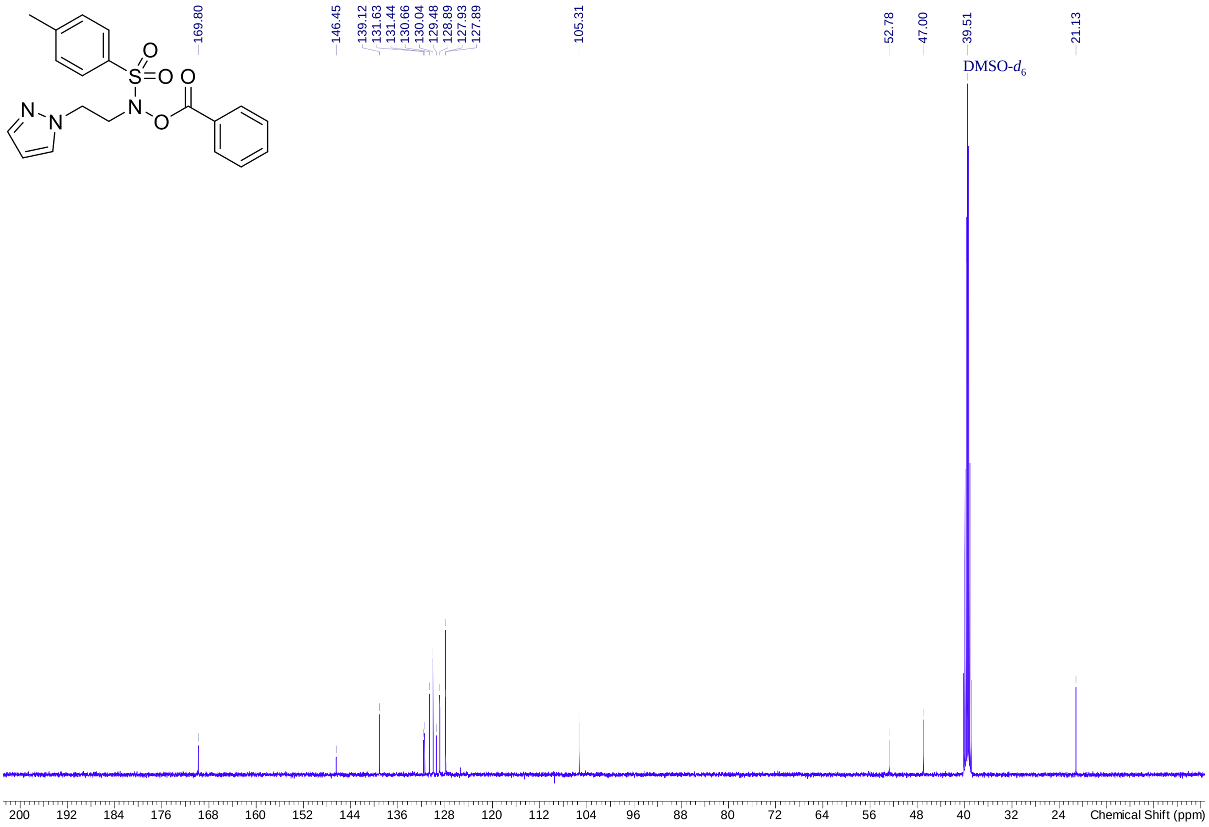

^{1}H NMR (400 MHz, DMSO-d_6) of Compound **S3c**

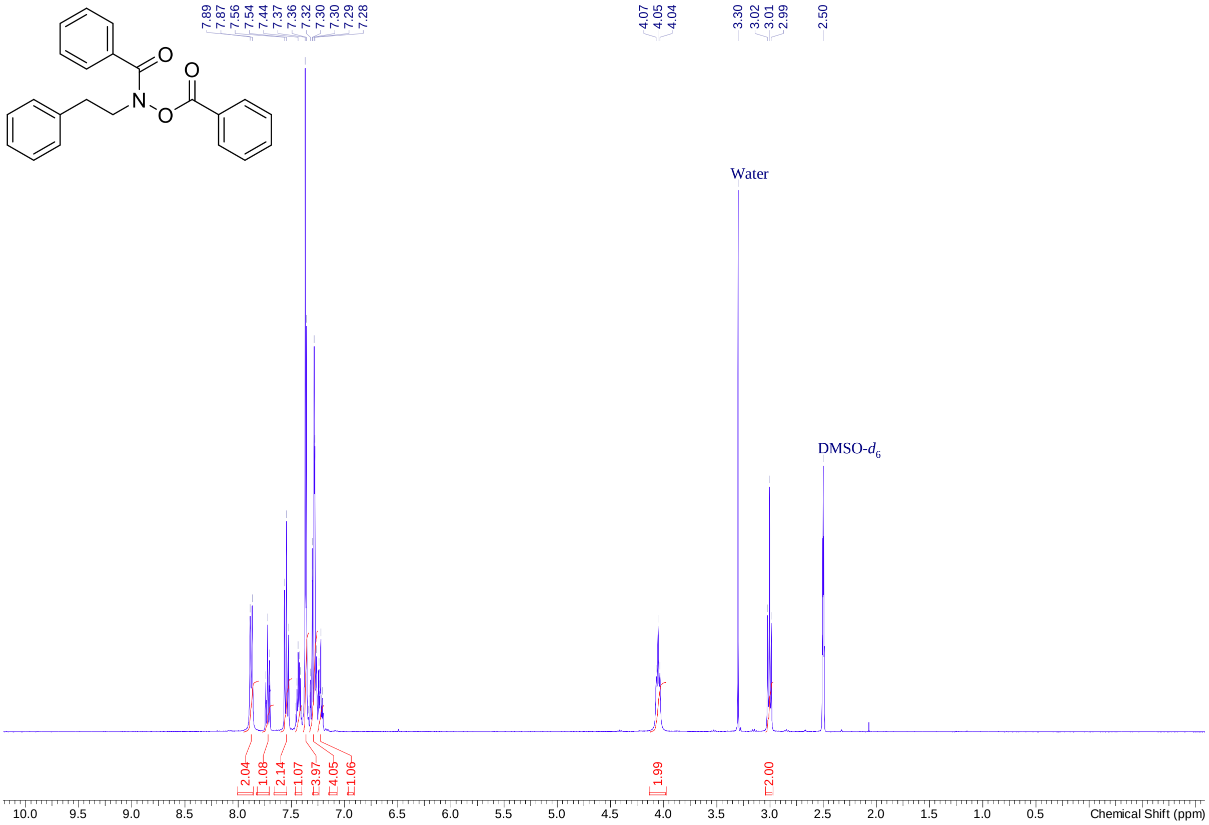

^{13}C NMR (101 MHz, DMSO-d_6) of Compound **S3c**

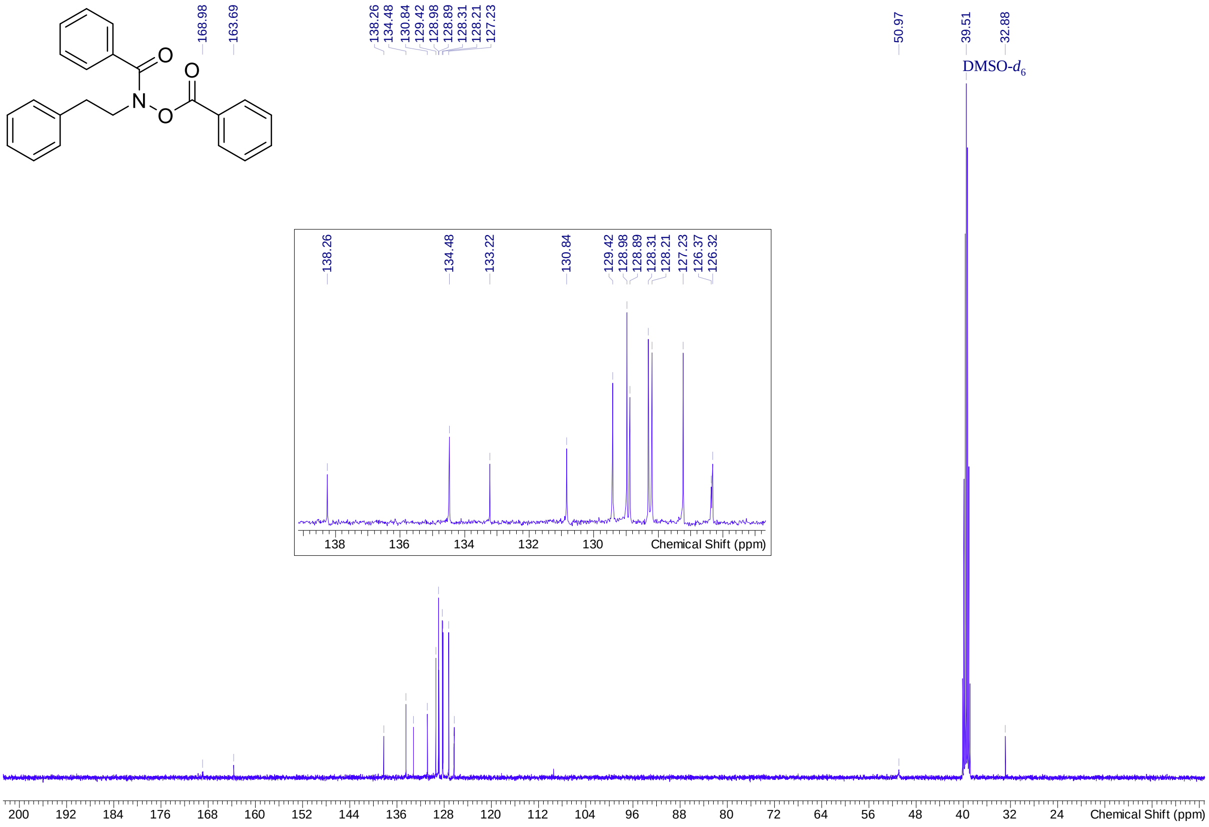

10. NMR Spectra of *N*-Trifluoromethylamines

Note: In the ^{13}C NMR the CF_3 quartets are expanded for clarity where appropriate.

^{1}H NMR (400 MHz, DMSO-d_6) of Compound **2a**

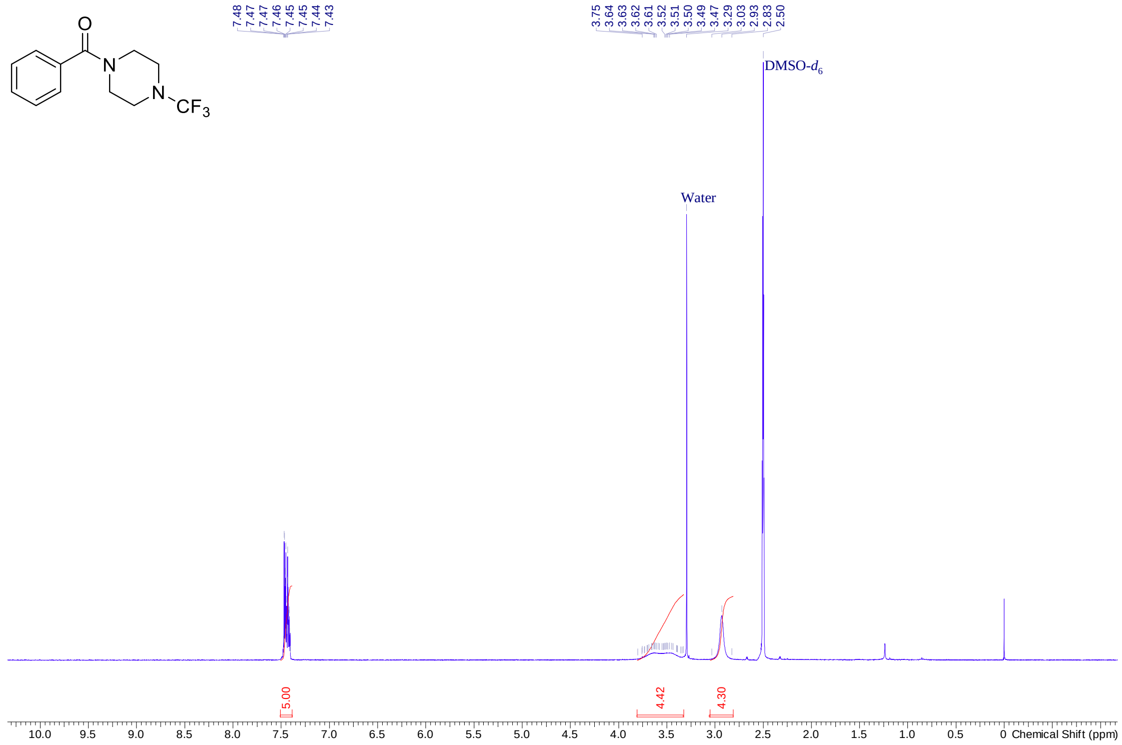

^{19}F NMR (376 MHz, DMSO-d_6) of Compound **2a**

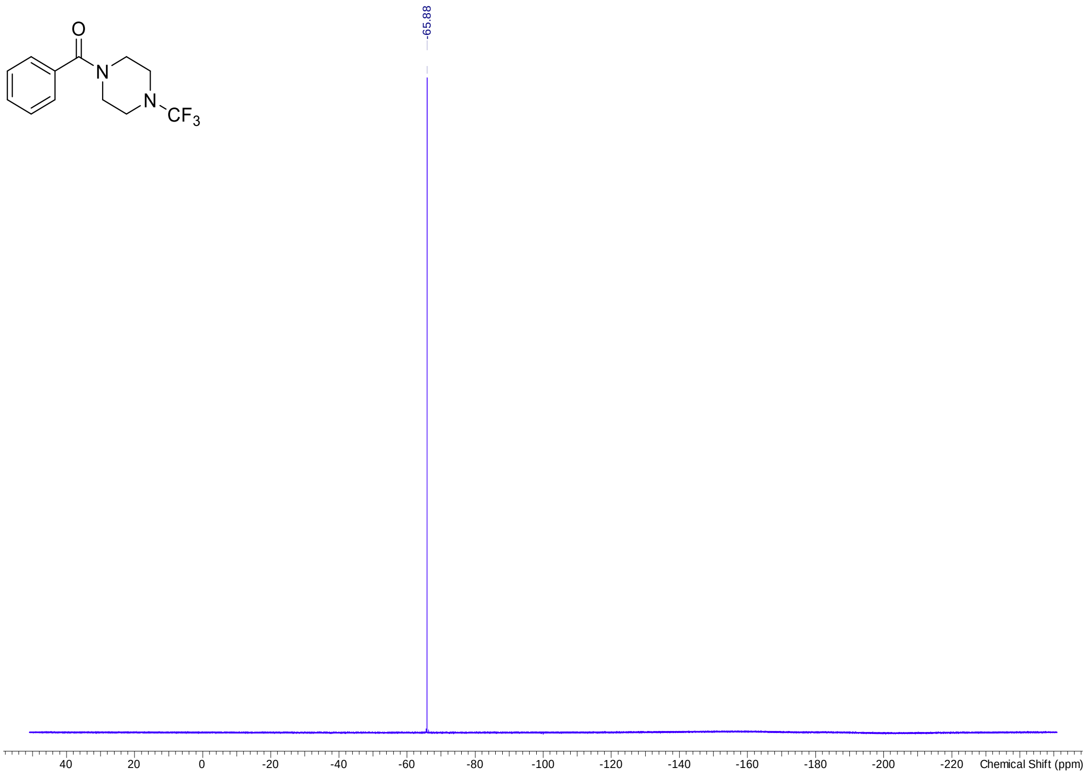

^{13}C NMR (151 MHz, DMSO-d_6) of Compound **2a**

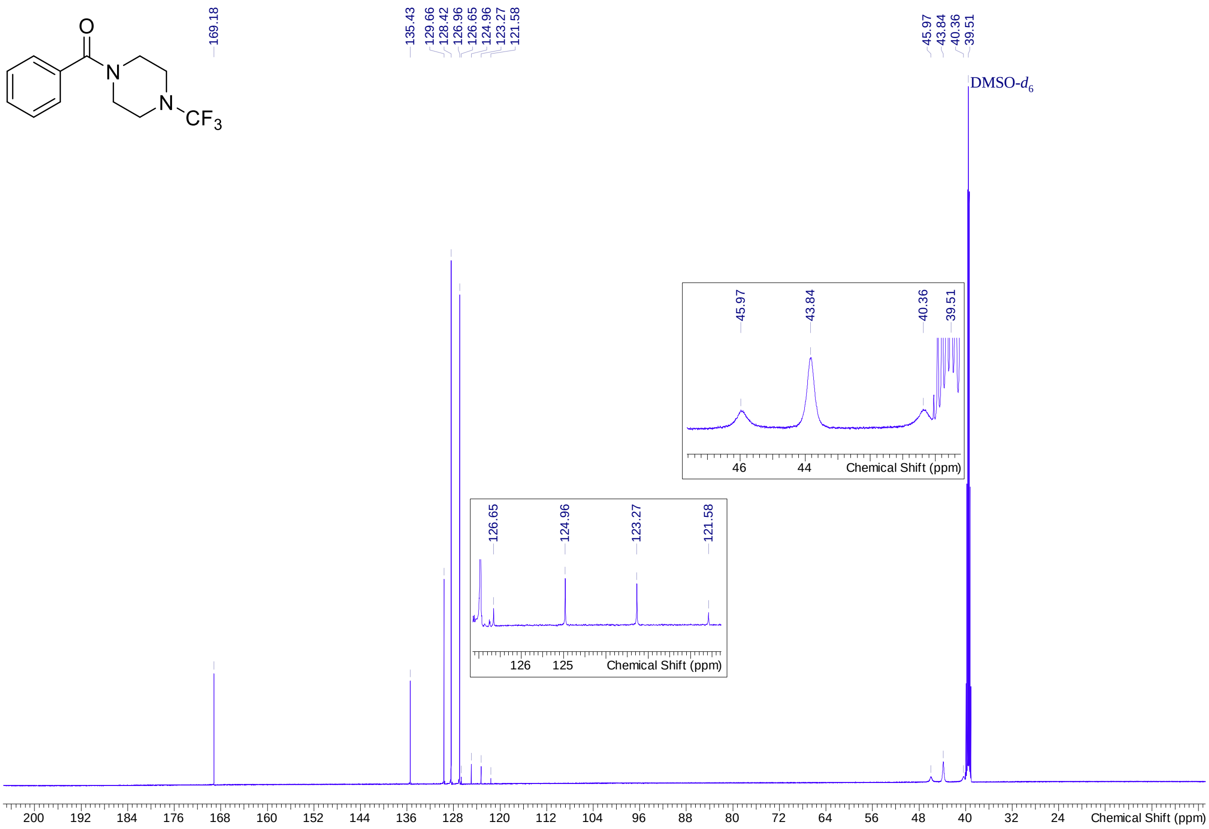

^{1}H NMR (600 MHz, DMSO-d_6) of Compound **2b**

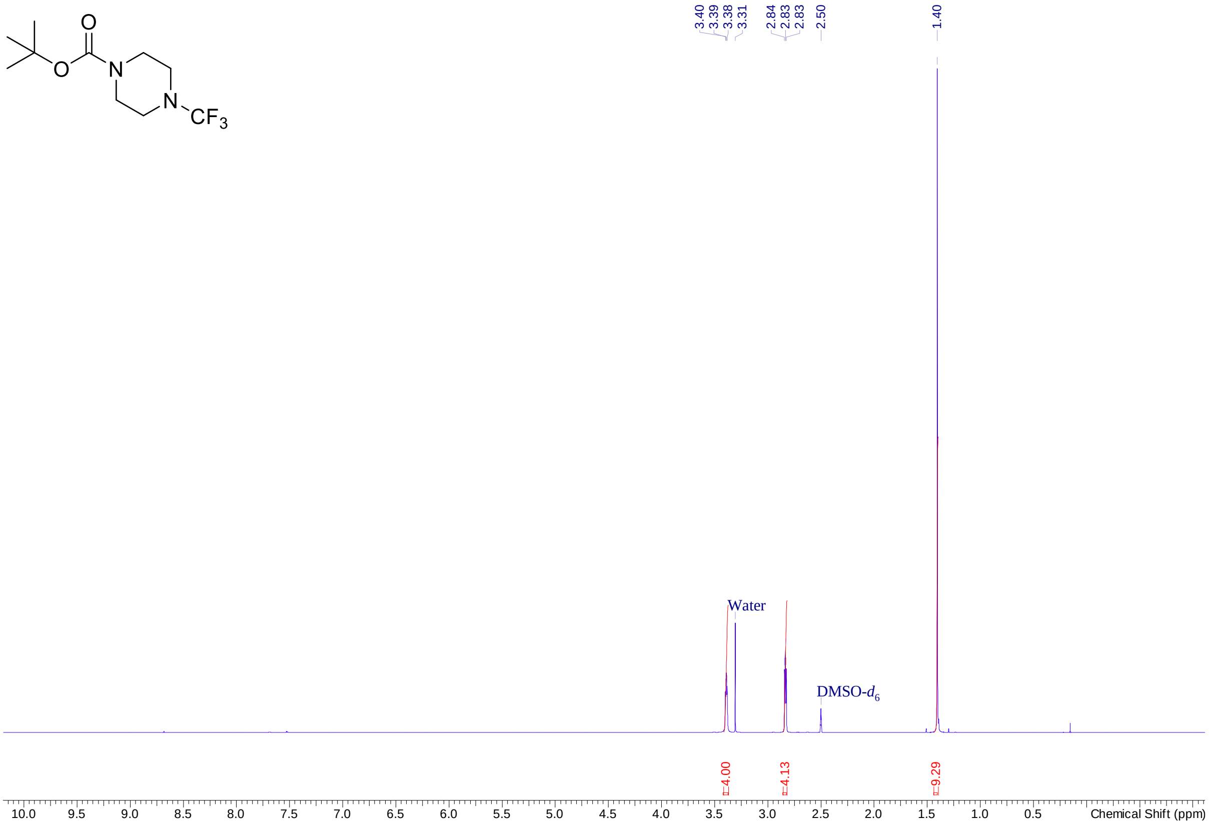

^{19}F NMR (376 MHz, DMSO-d_6) of Compound **2b**

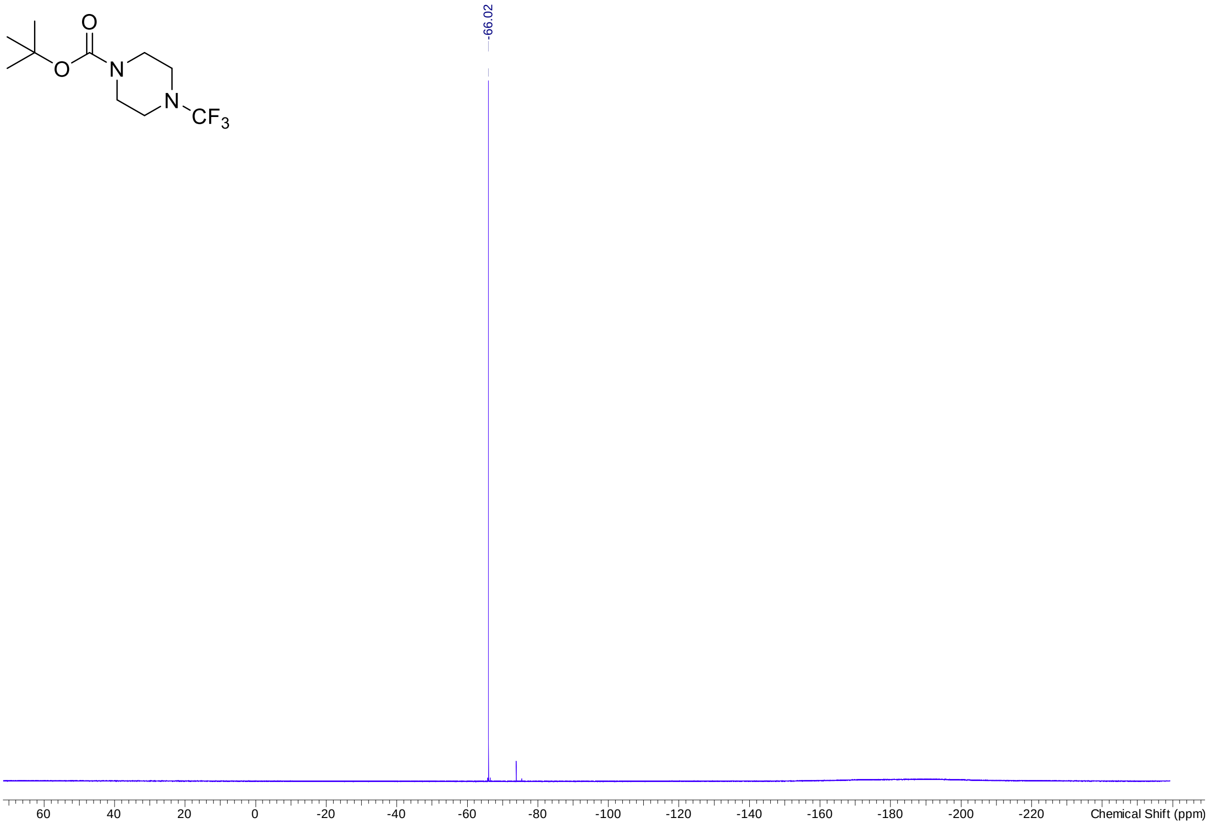

^{13}C NMR (151 MHz, DMSO-d_6) of Compound **2b**

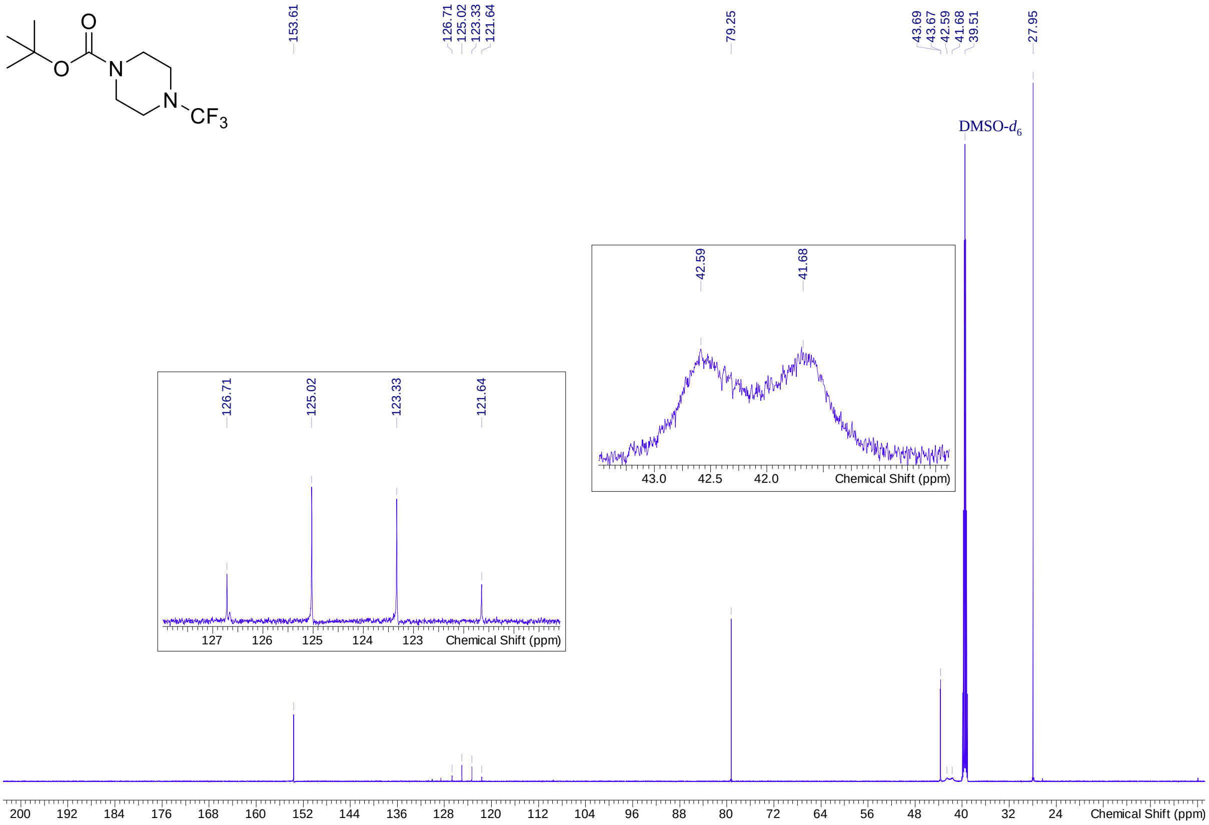

^{1}H NMR (400 MHz, DMSO-d_6) of Compound **2c**

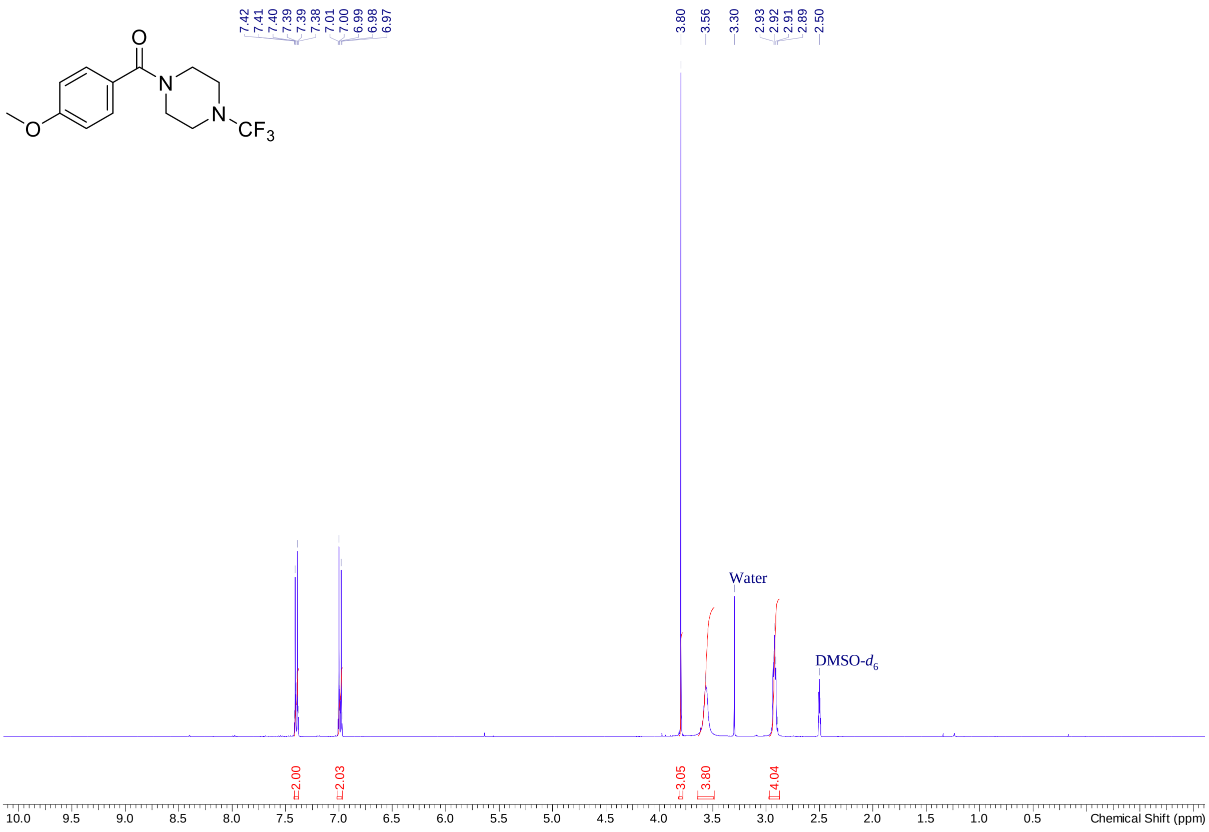

^{19}F NMR (376 MHz, DMSO-d_6) of Compound **2c**

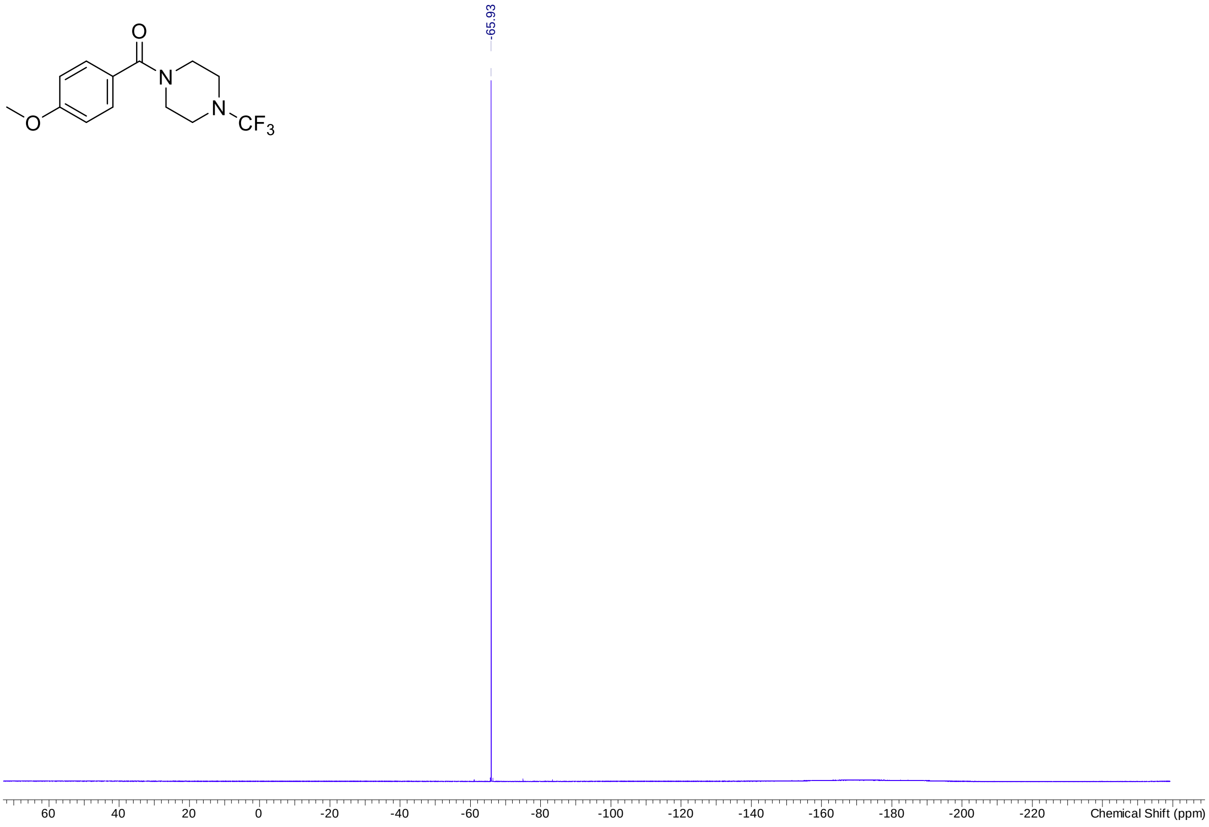

^{13}C NMR (151 MHz, DMSO-d_6) of Compound **2c**

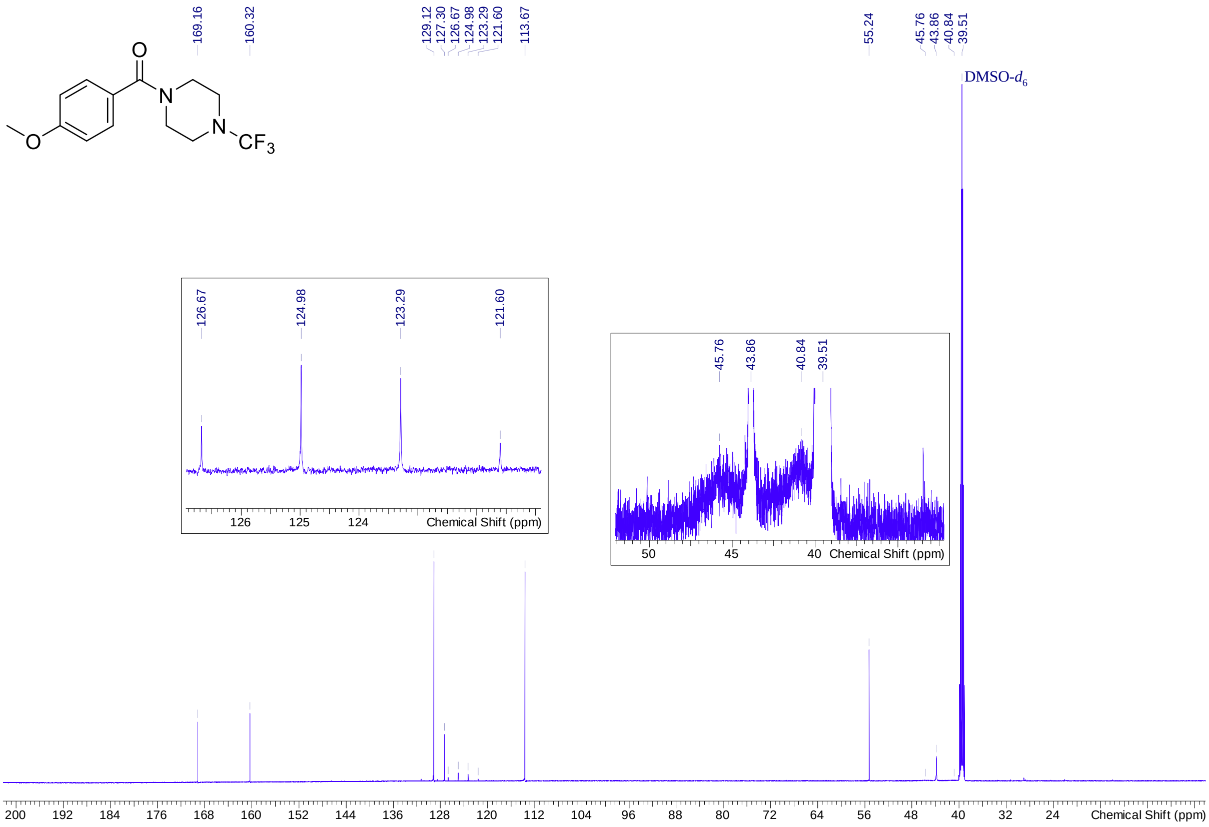

^{1}H NMR (400 MHz, $CDCl_3$) of Compound **2d**

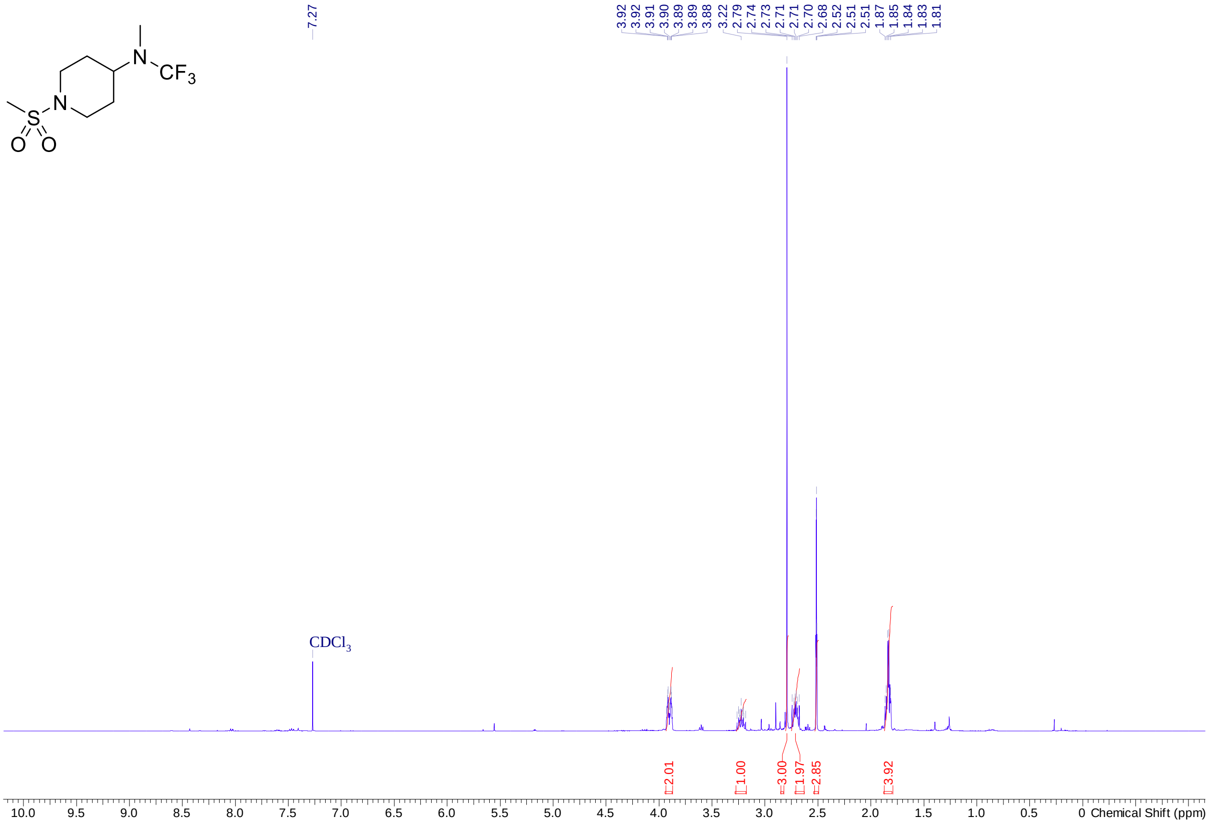

^{19}F NMR (376 MHz, $CDCl_3$) of Compound **2d**

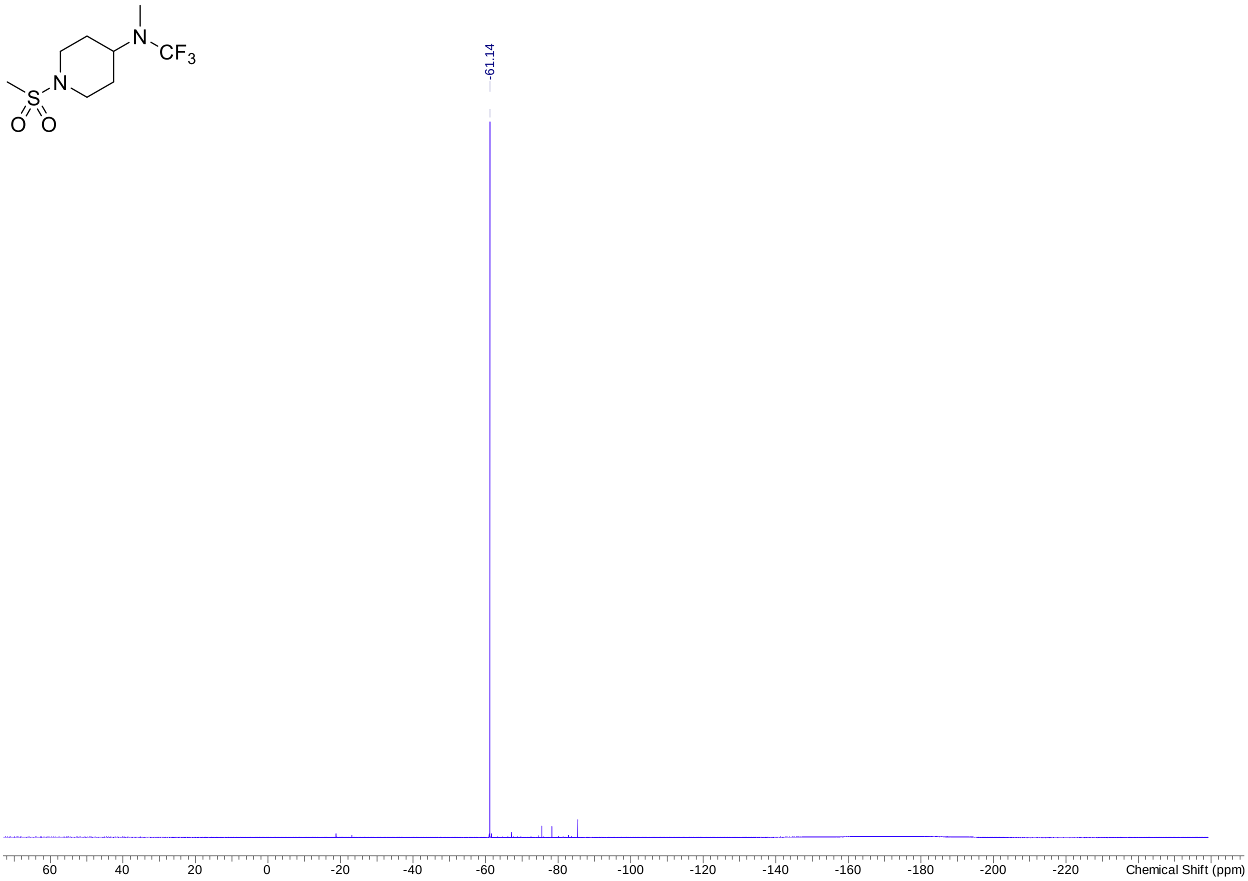

^{13}C NMR (101 MHz, $CDCl_3$) of Compound **2d**

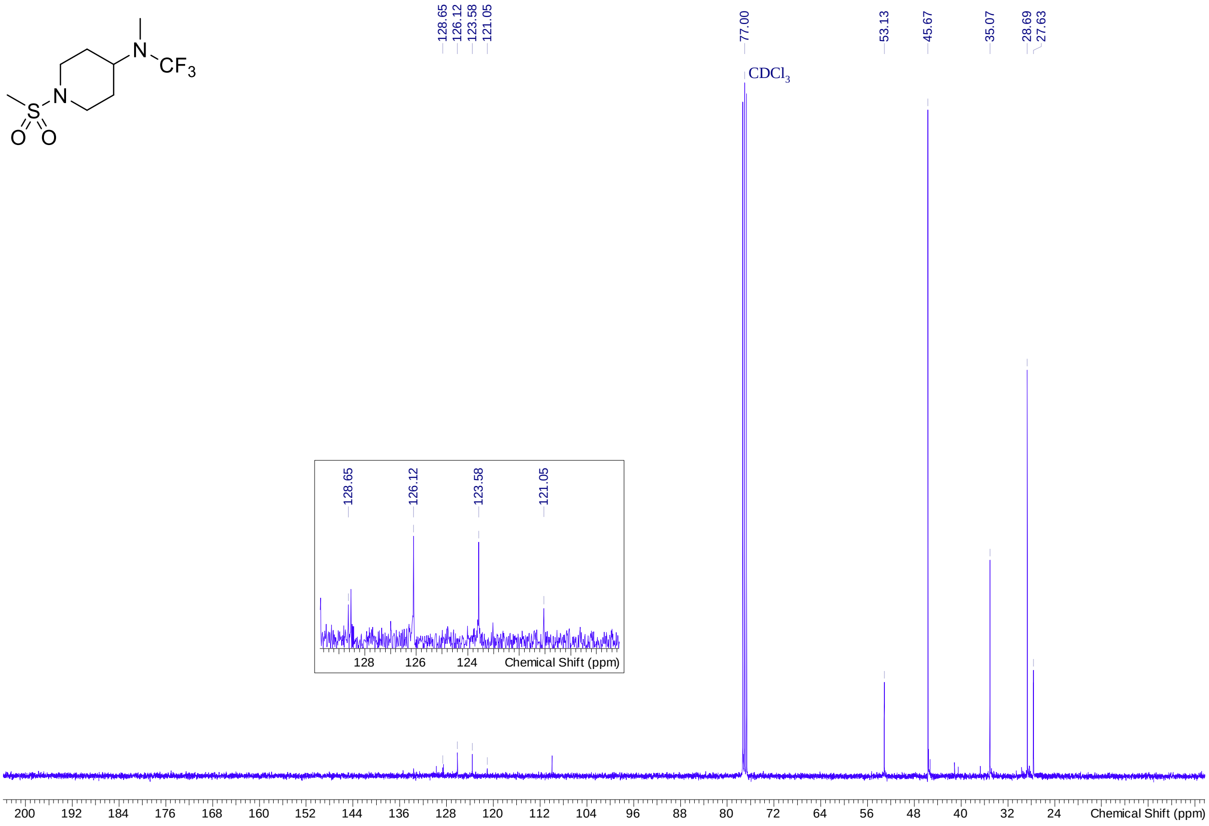

^1H NMR (400 MHz, DMSO-d_6) of Compound **2e**

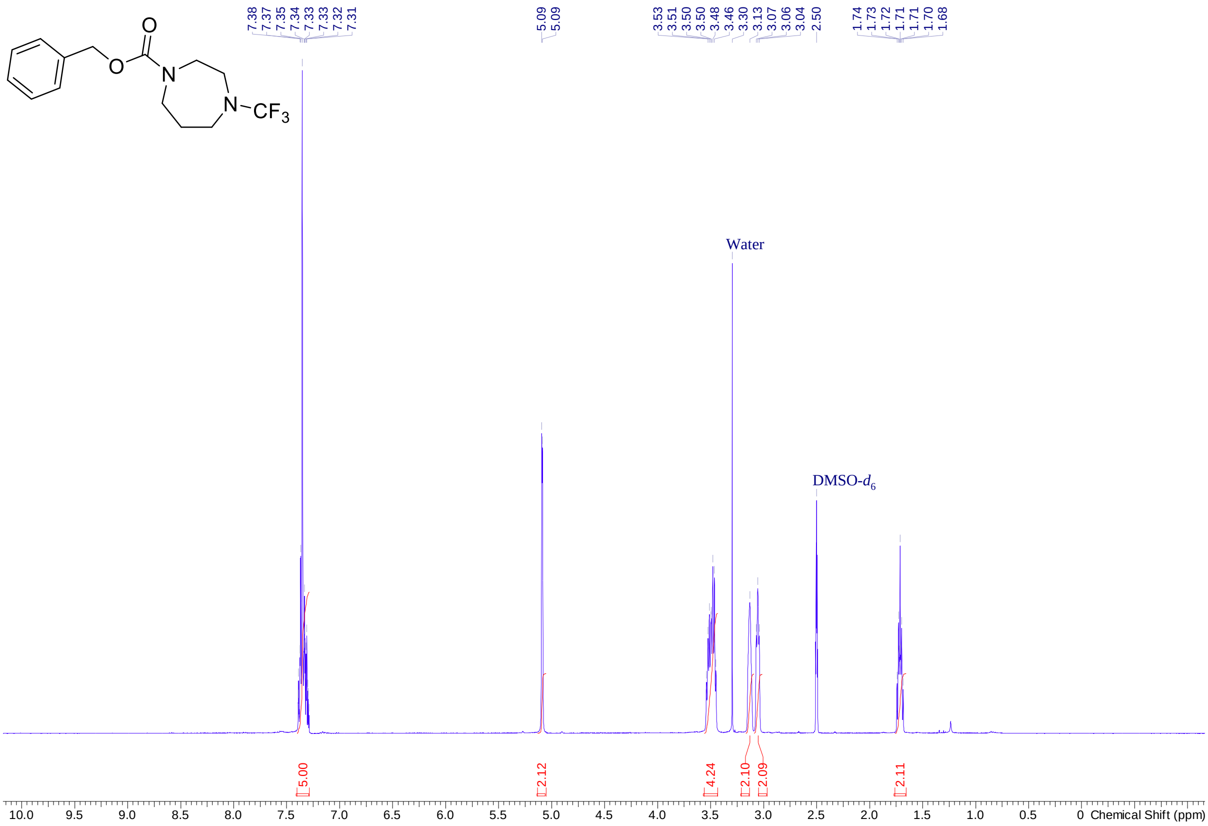

^{19}F NMR (376 MHz, DMSO-d_6) of Compound **2e**

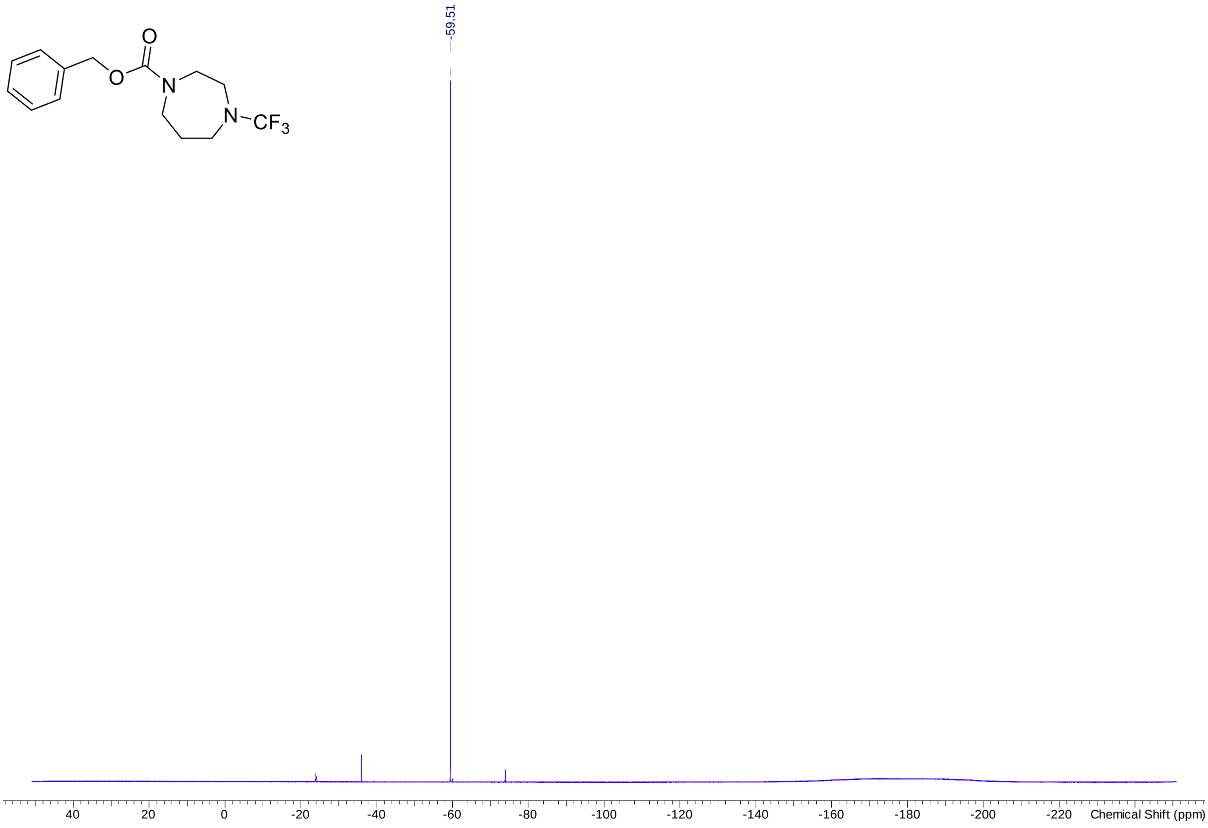

^{13}C NMR (151 MHz, DMSO-d_6) of Compound **2e**

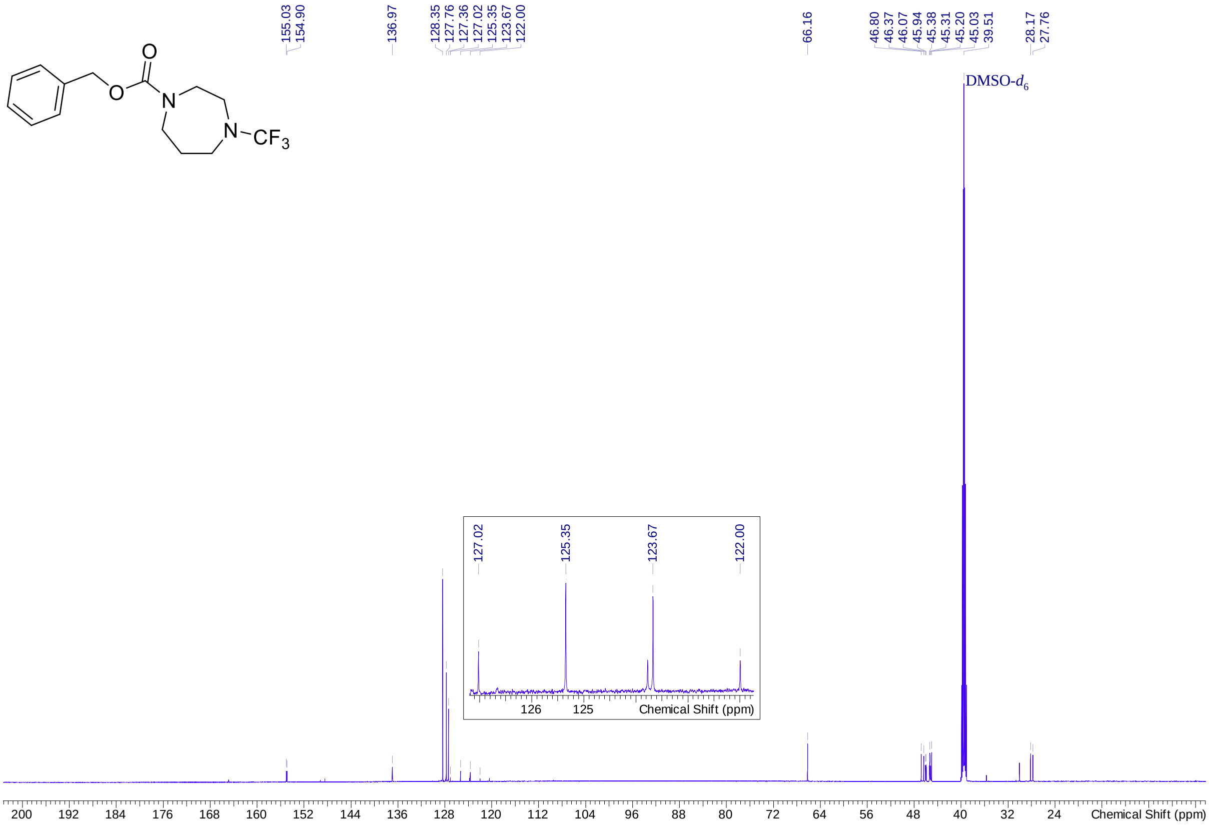

^{1}H NMR (400 MHz, DMSO-d_6) of Compound **2f**

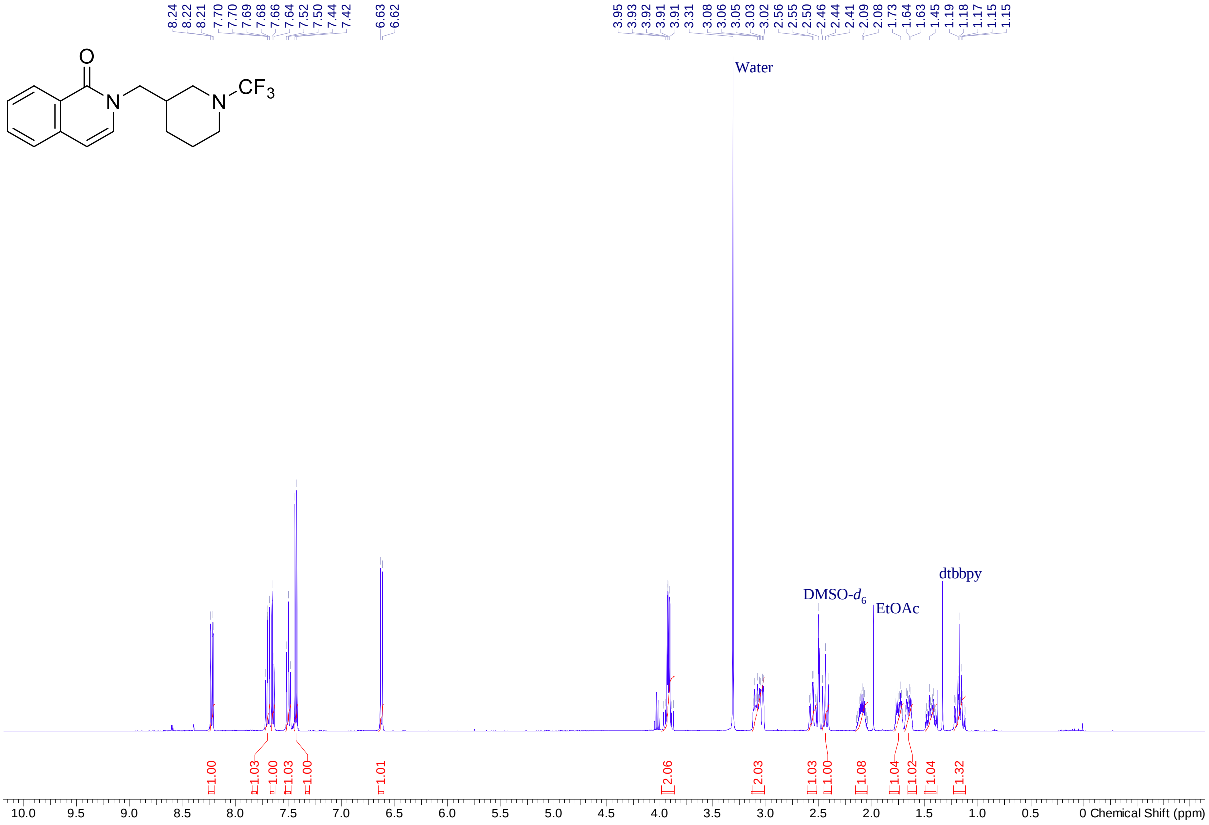

^{19}F NMR (376 MHz, DMSO-d_6) of Compound **2f**

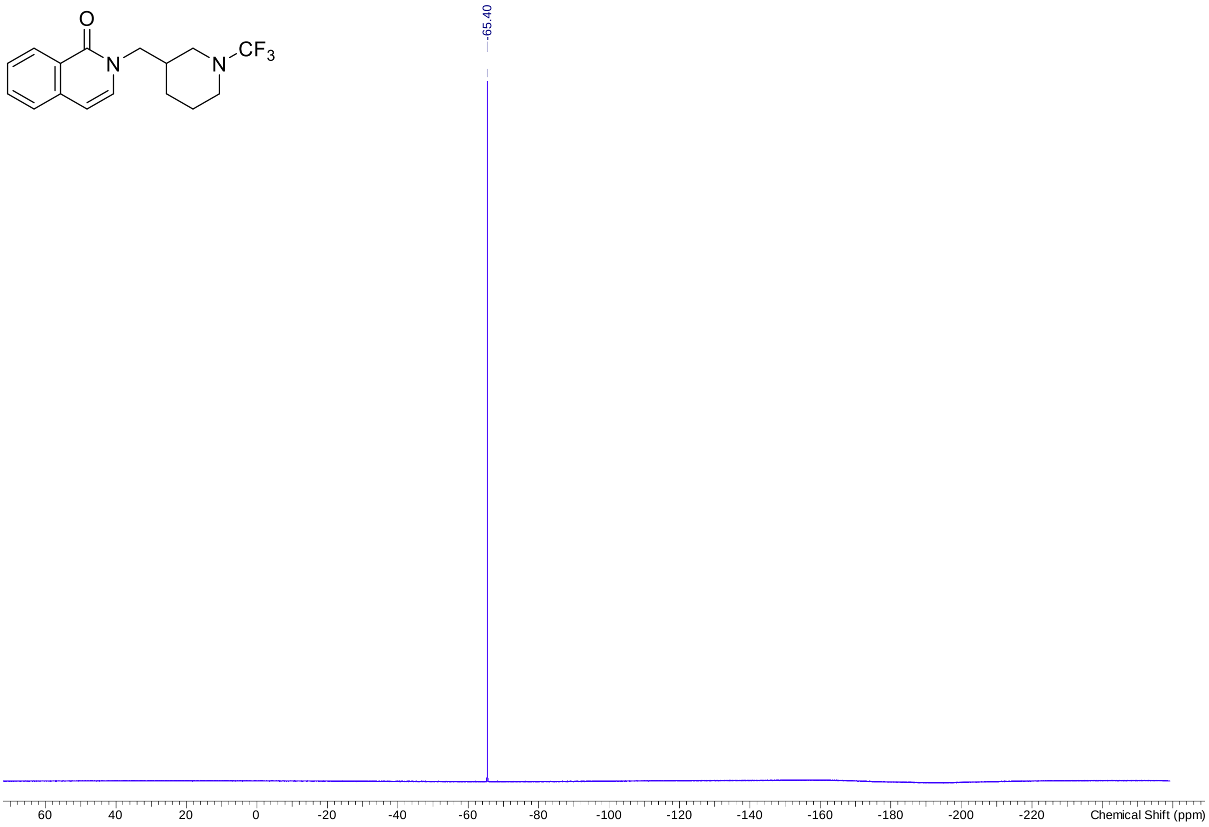

^{13}C NMR (101 MHz, DMSO-d_6) of Compound **2f**

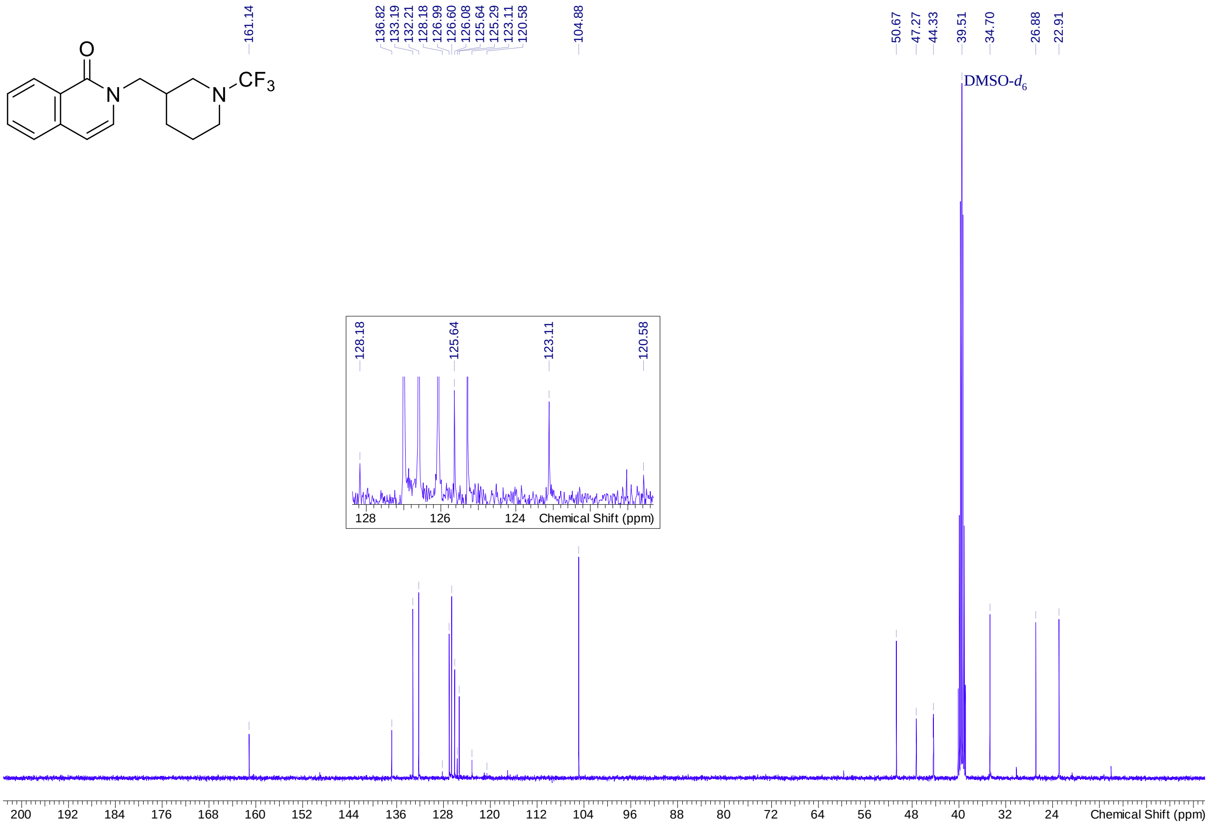

^{1}H NMR (400 MHz, DMSO-d_6) of Compound **2g**

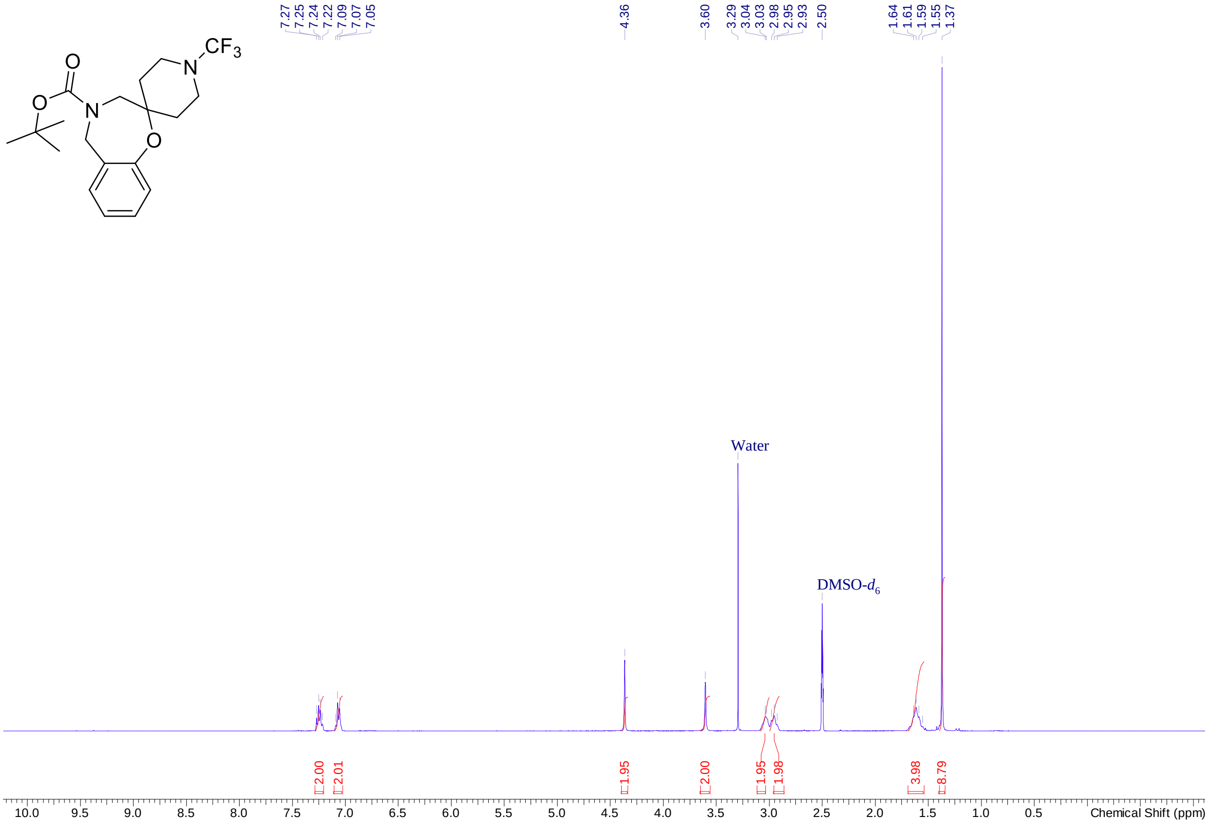

^{19}F NMR (376 MHz, DMSO-d_6) of Compound **2g**

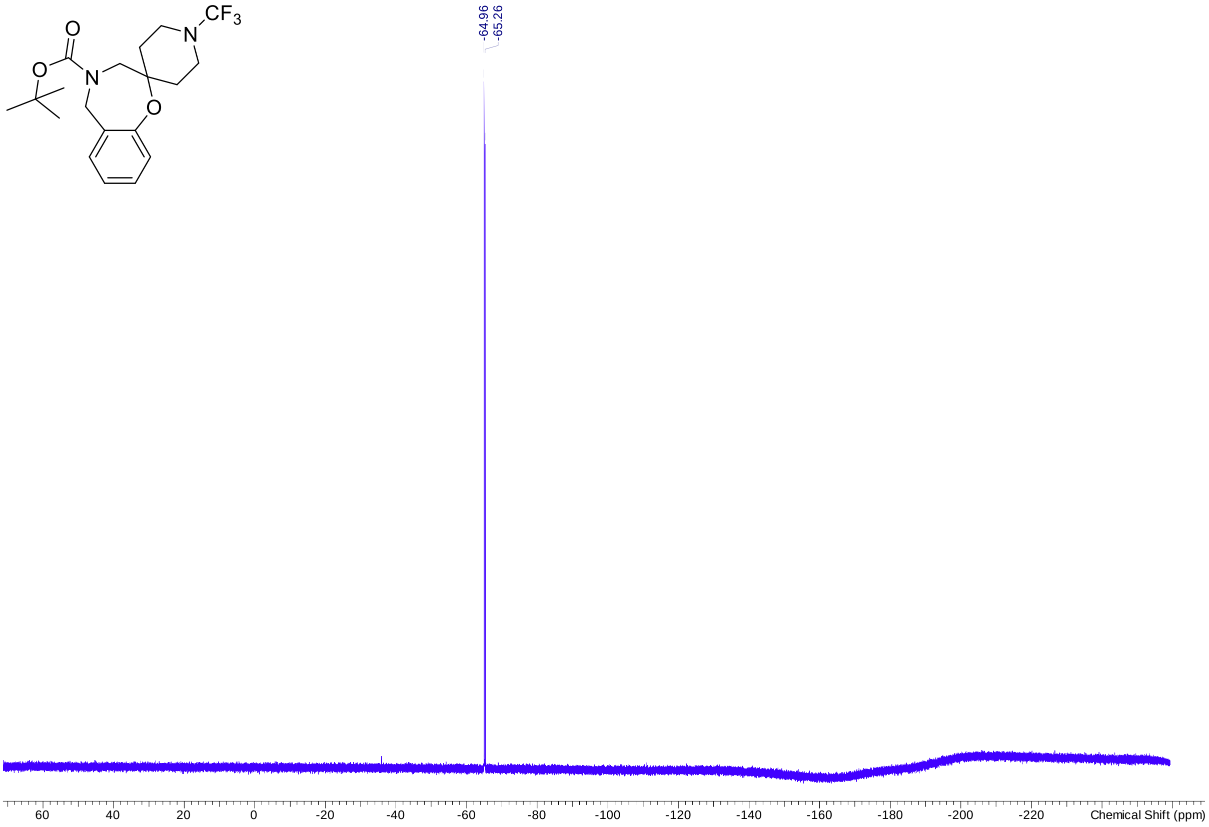

^{13}C NMR (151 MHz, DMSO-d_6) of Compound **2g**

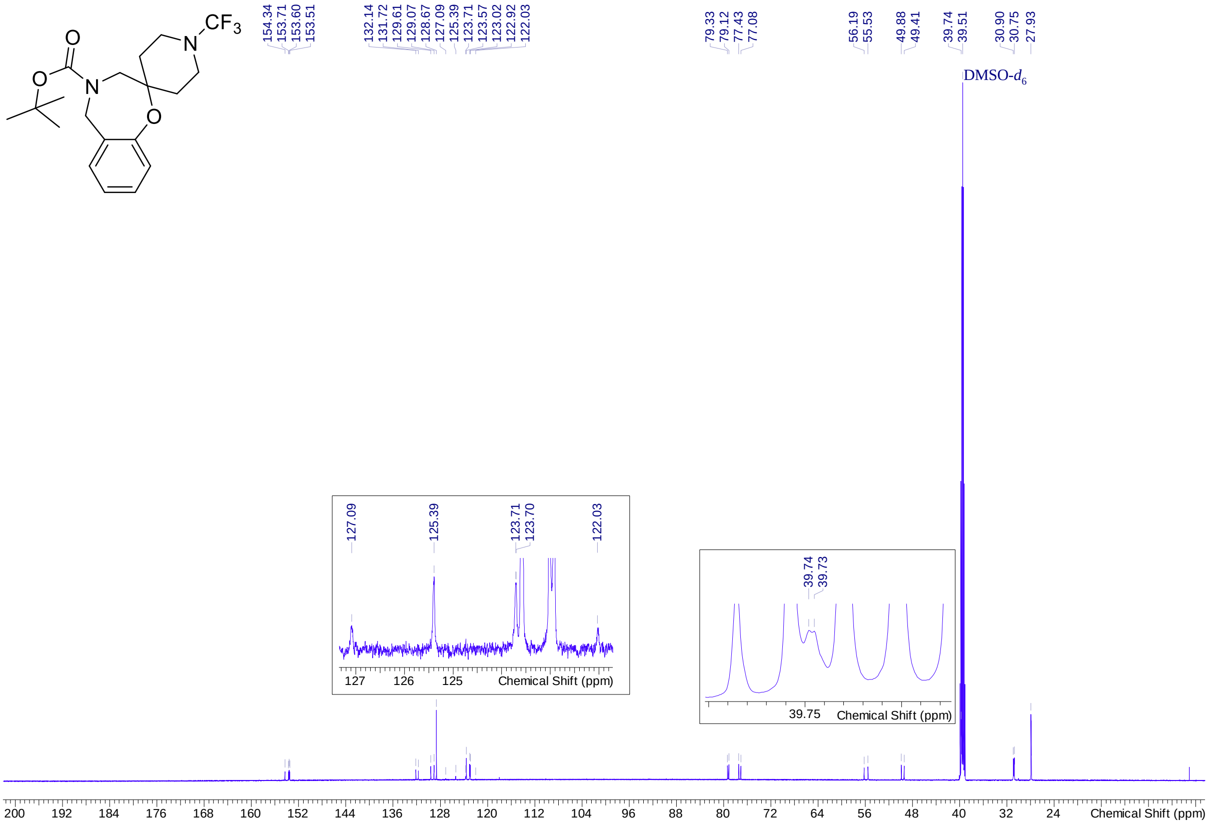

^{1}H NMR (600 MHz, DMSO-d_6) of Compound **2h**

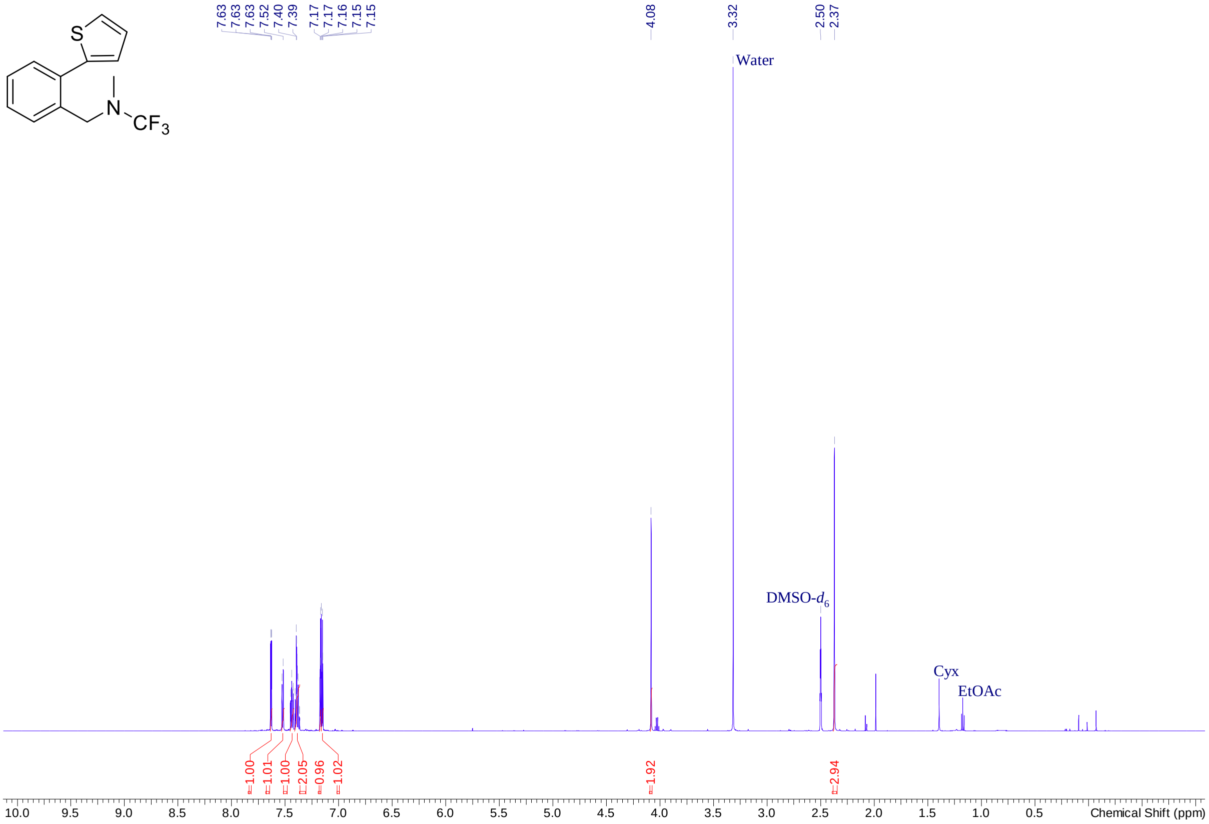

^{19}F NMR (376 MHz, DMSO-d_6) of Compound **2h**

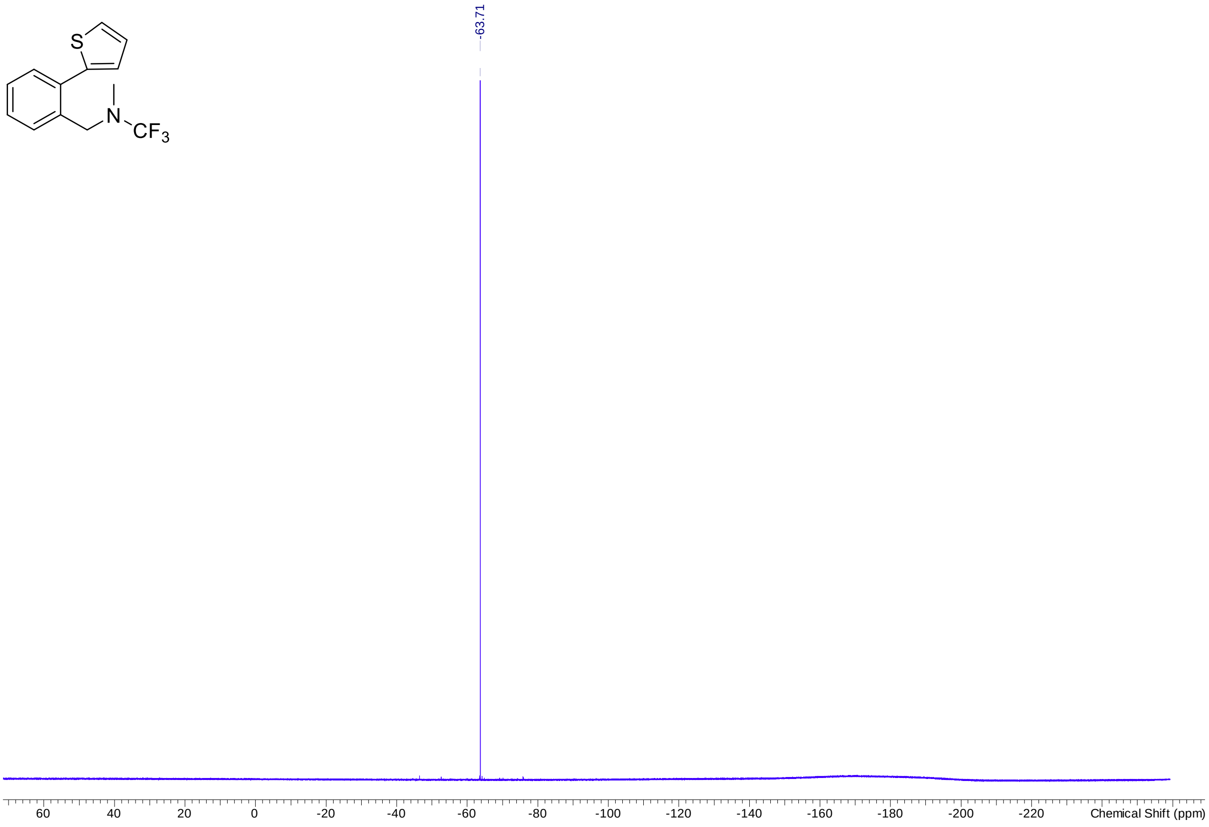

^{13}C NMR (151 MHz, DMSO-d_6) of Compound **2h**

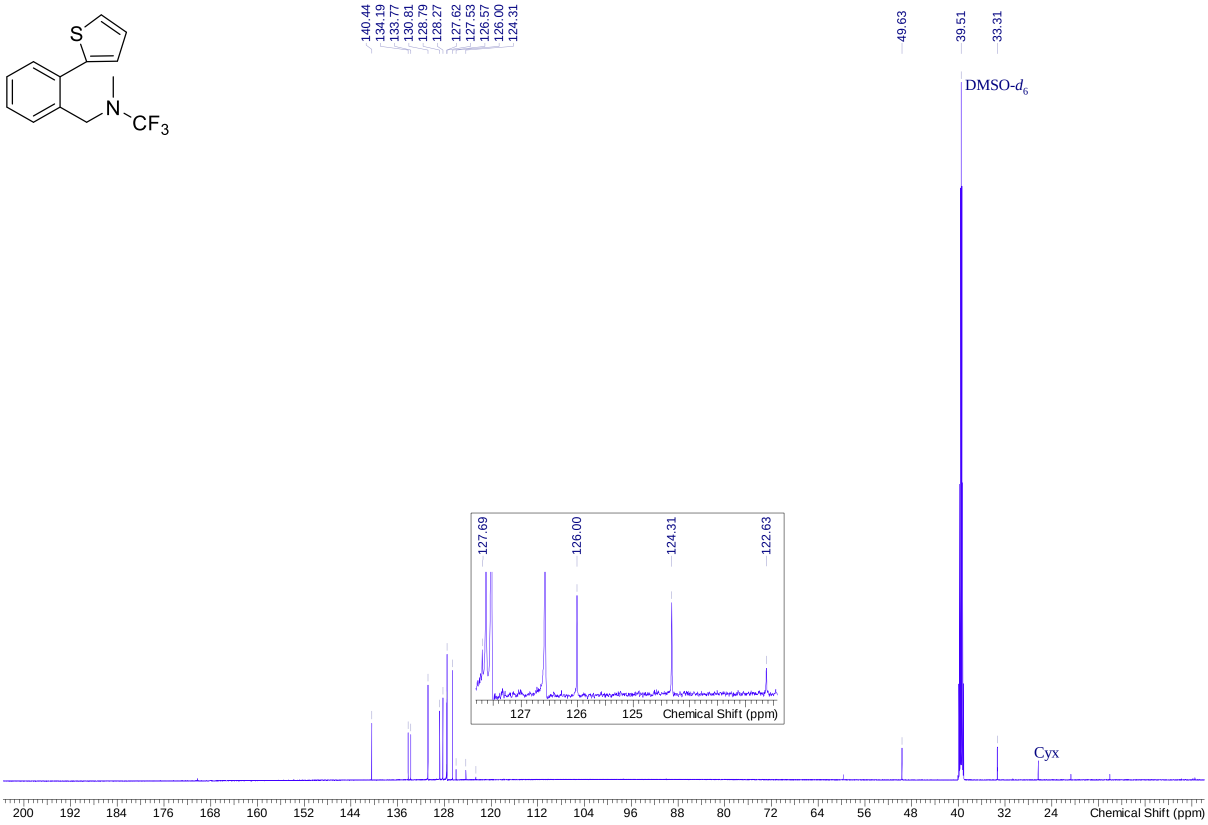

^{1}H NMR (600 MHz, DMSO-d_6) of Compound **2i**

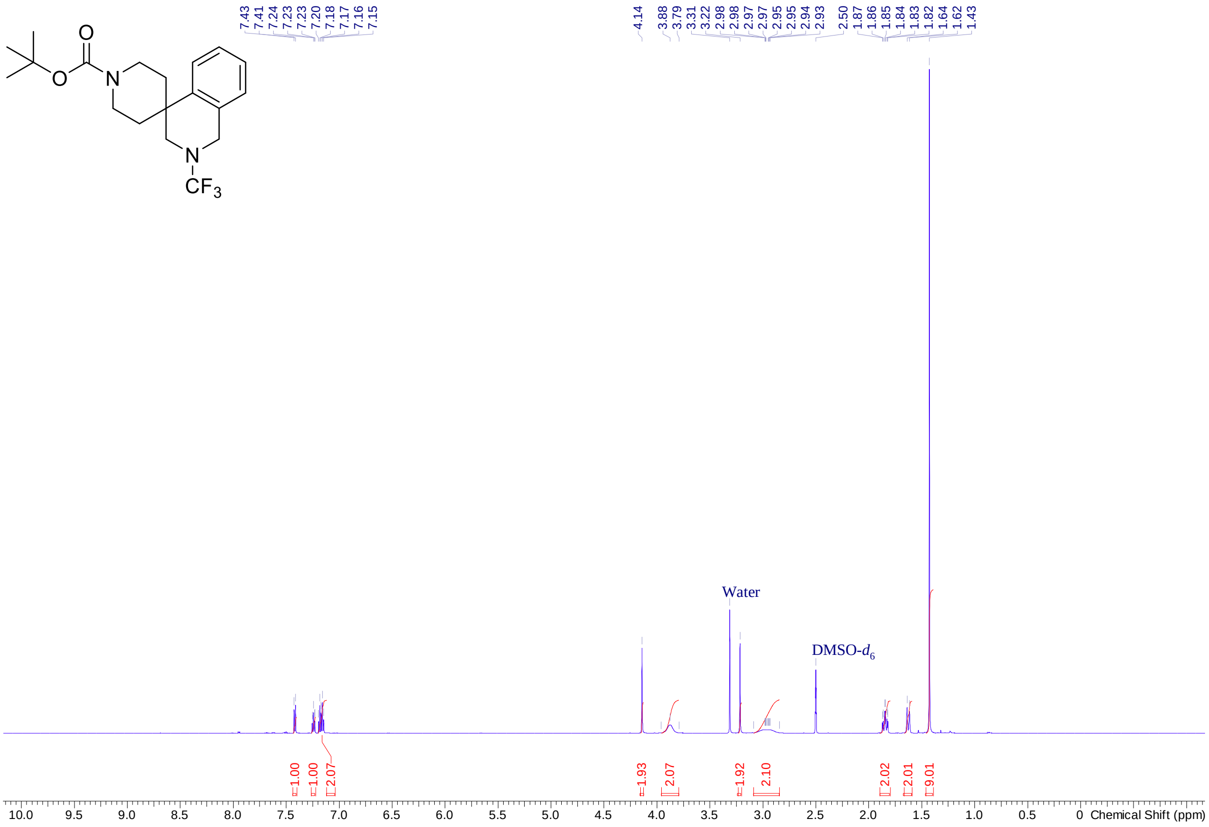

^{19}F NMR (376 MHz, DMSO-d_6) of Compound **2i**

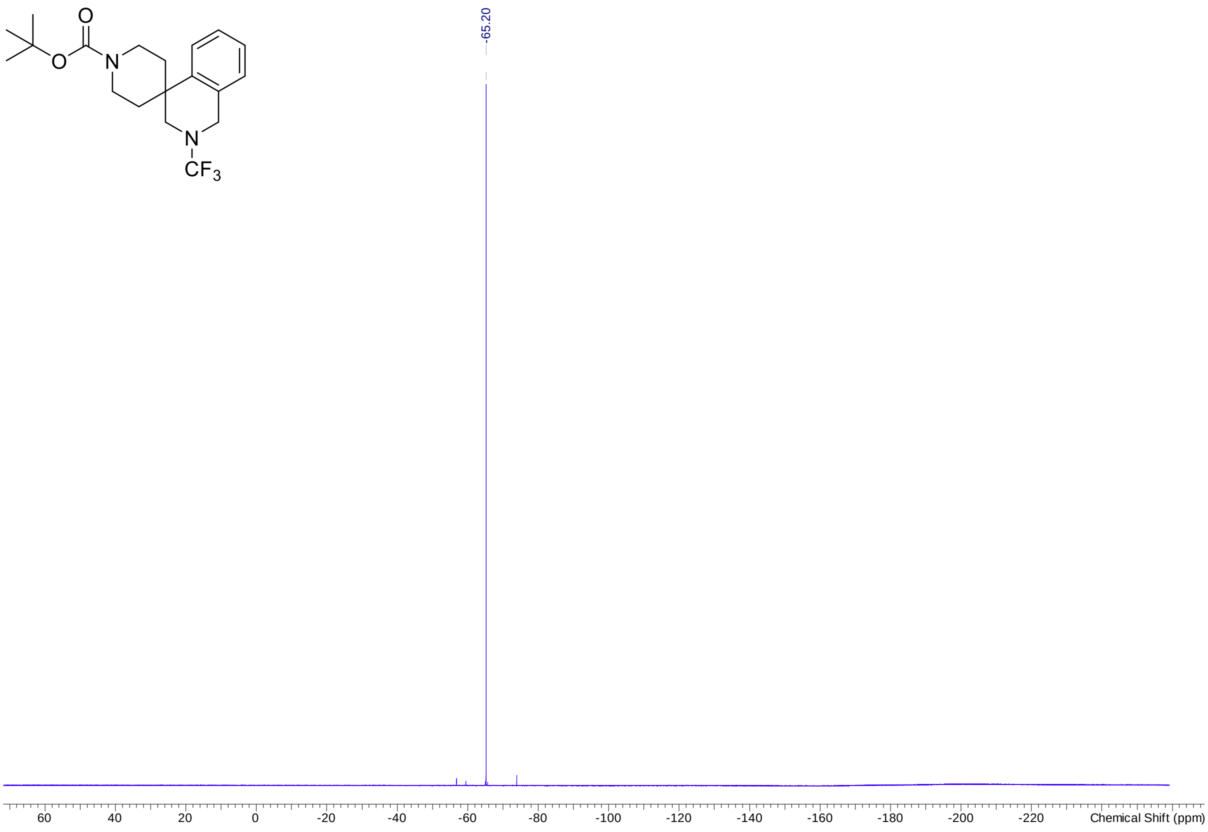

^{13}C NMR (151 MHz, DMSO-d_6) of Compound **2i**

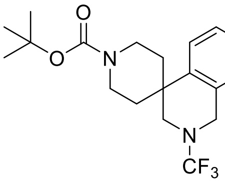

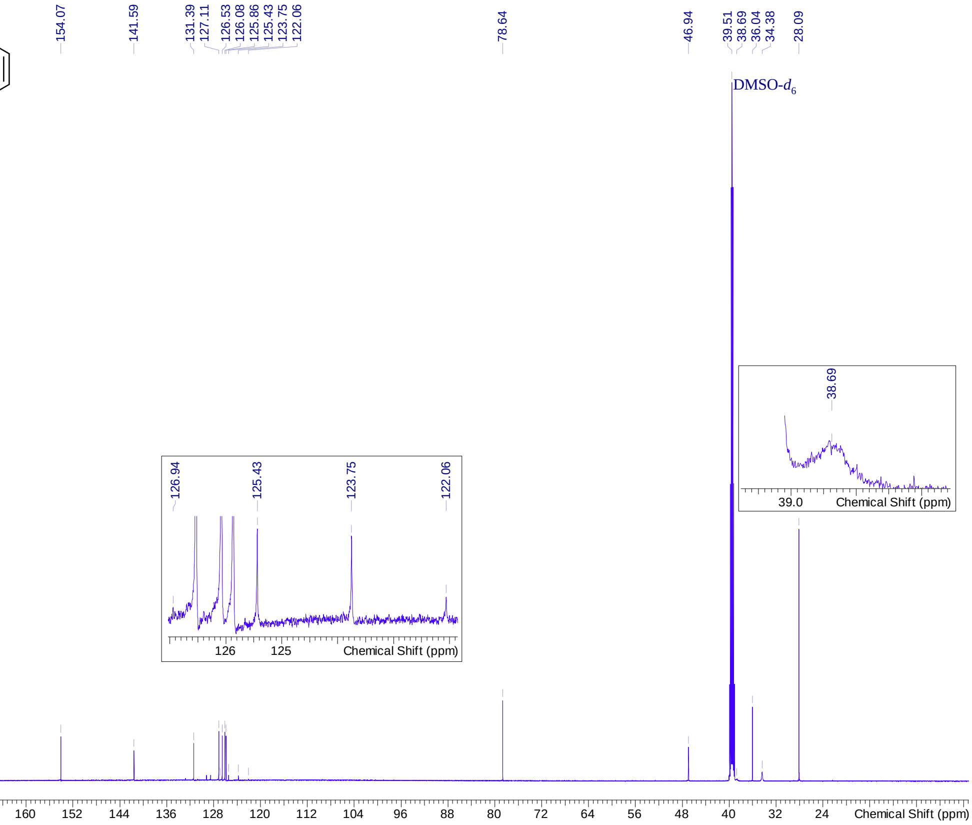

Partial HSQC-DEPT (^{1}H, 600 MHz, DMSO-d_6, ^{13}C, 151 MHz, DMSO-d_6) of Compound **2i**.
Red = **C**H or **C**H_3; Blue = **C**H_2

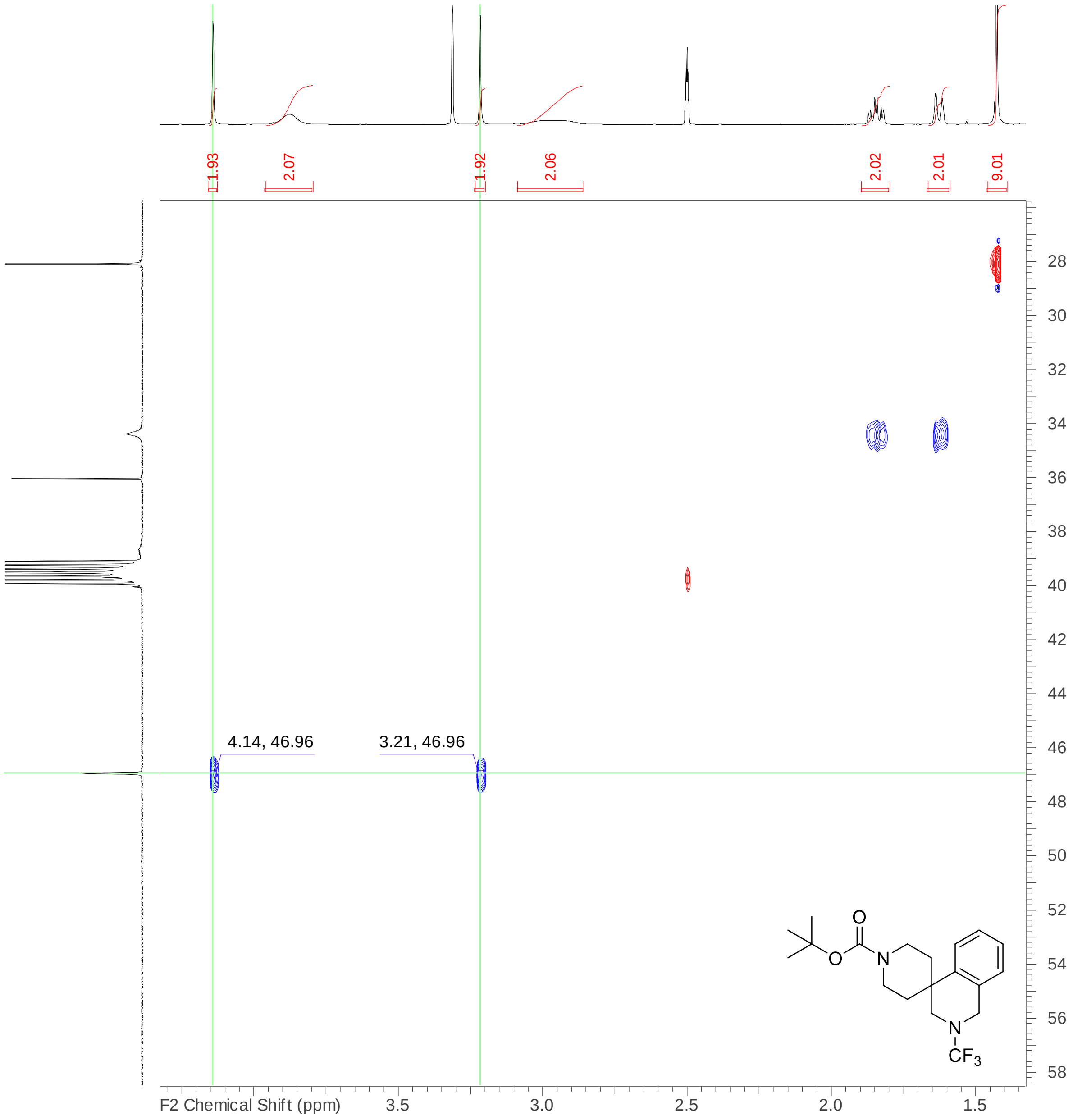

^{1}H NMR (600 MHz, $CDCl_3$) of Compound **2j**

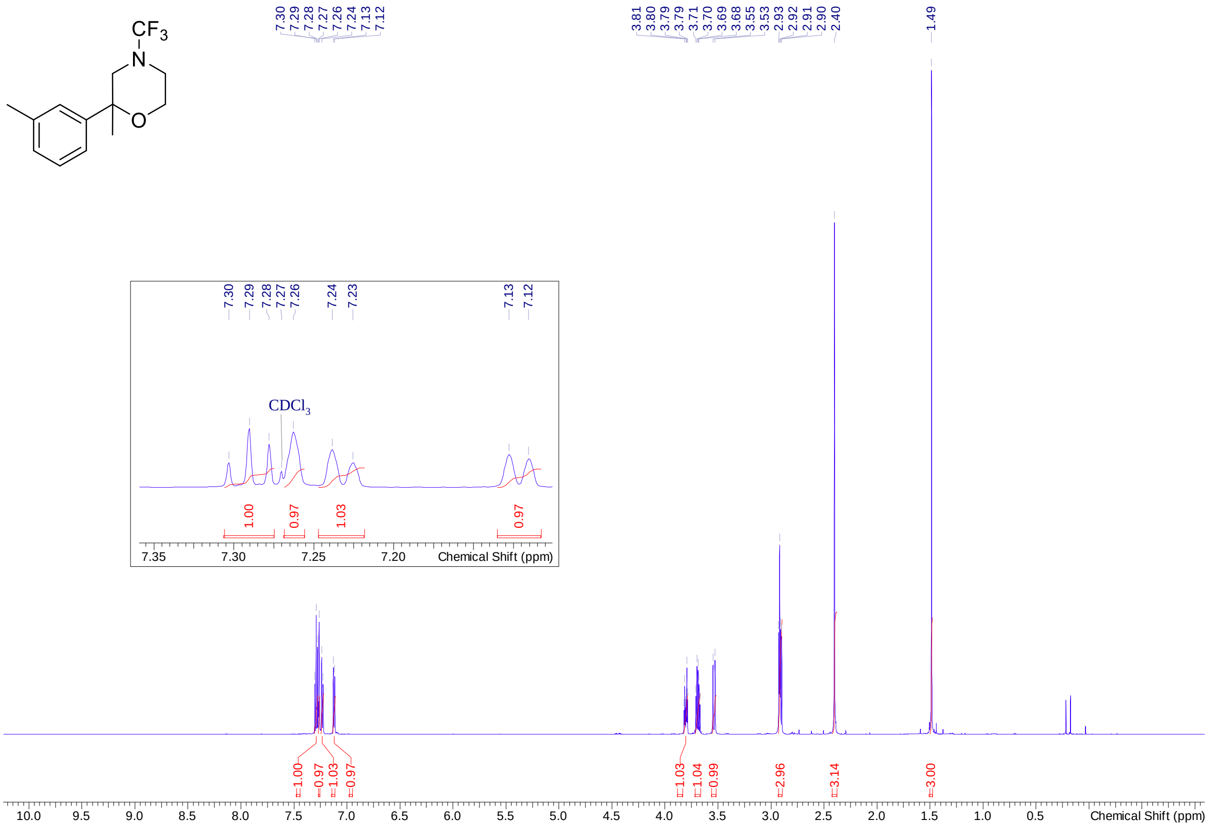

^{19}F NMR (376 MHz, $CDCl_3$) of Compound **2j**

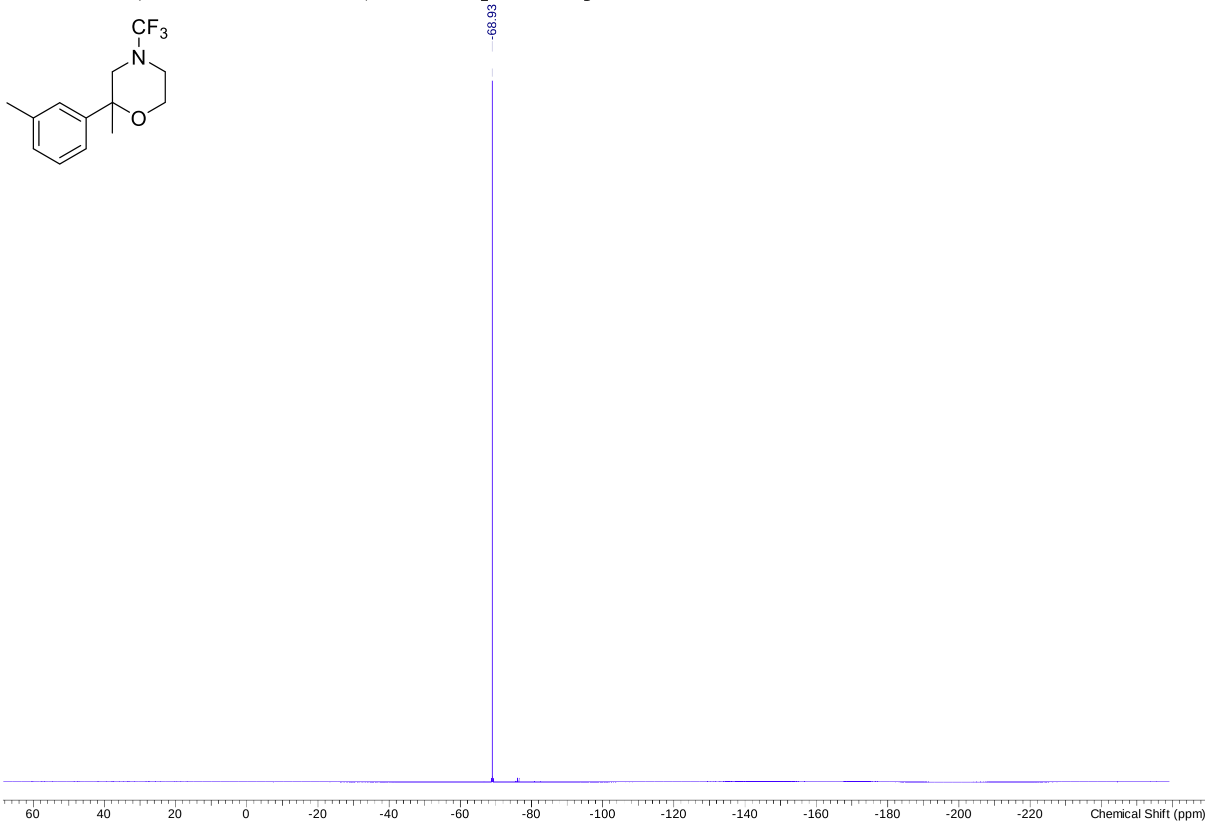

^{13}C NMR (151 MHz, $CDCl_3$) of Compound **2j**

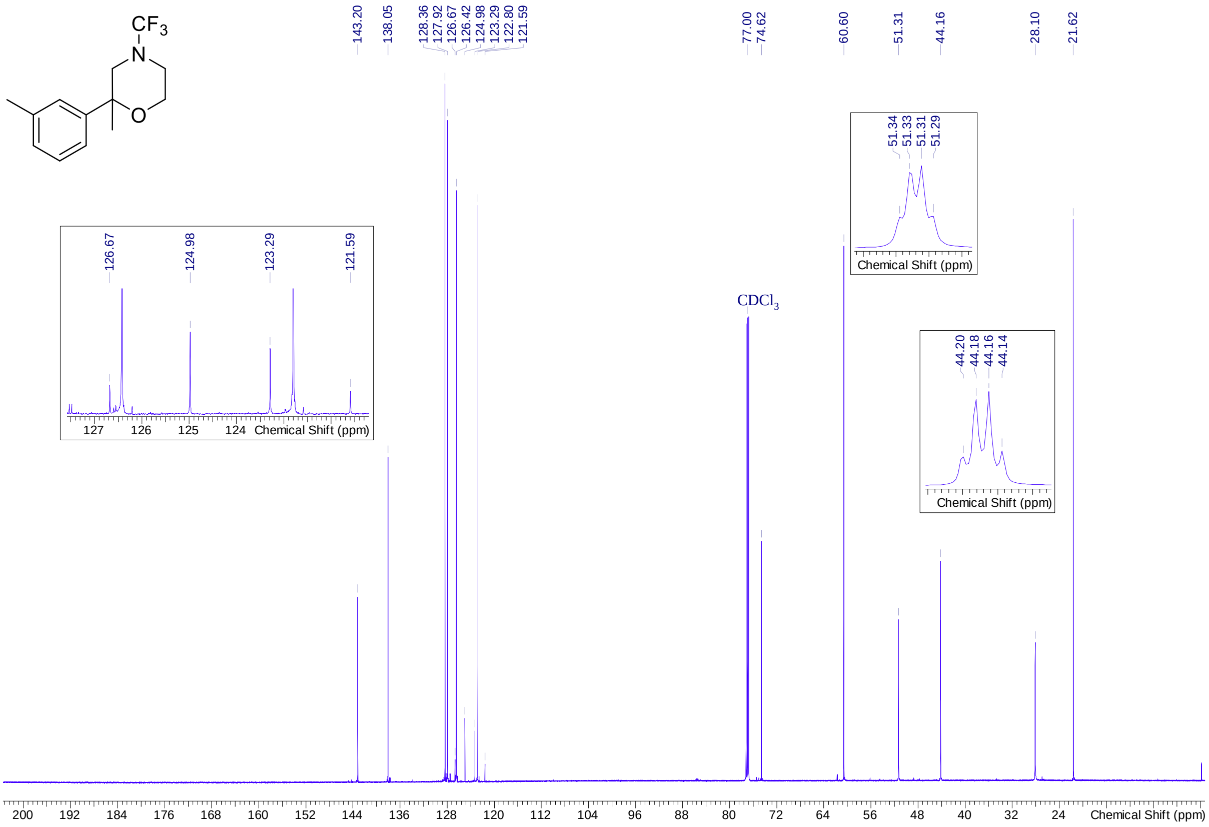

^{1}H NMR (400 MHz, DMSO-d_6) of Compound **2k**

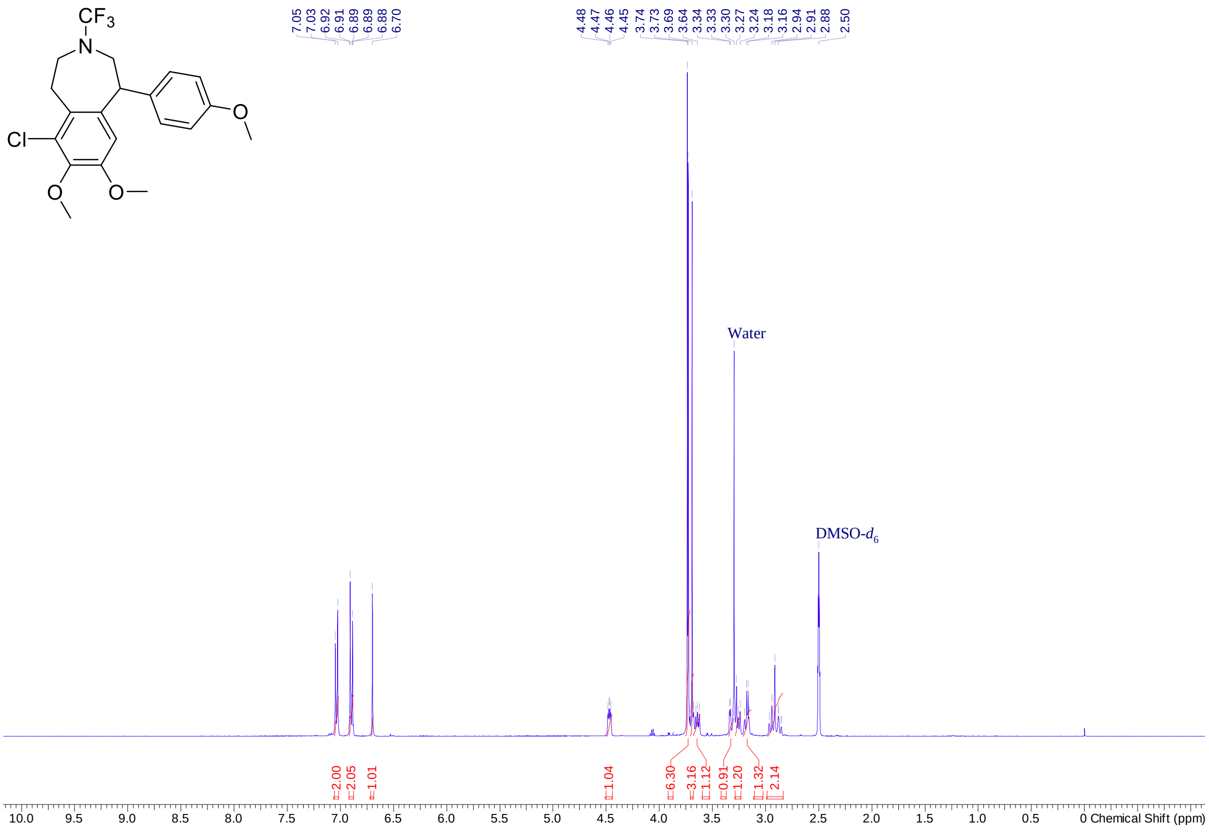

^{19}F NMR (376 MHz, DMSO-d_6) of Compound **2k**

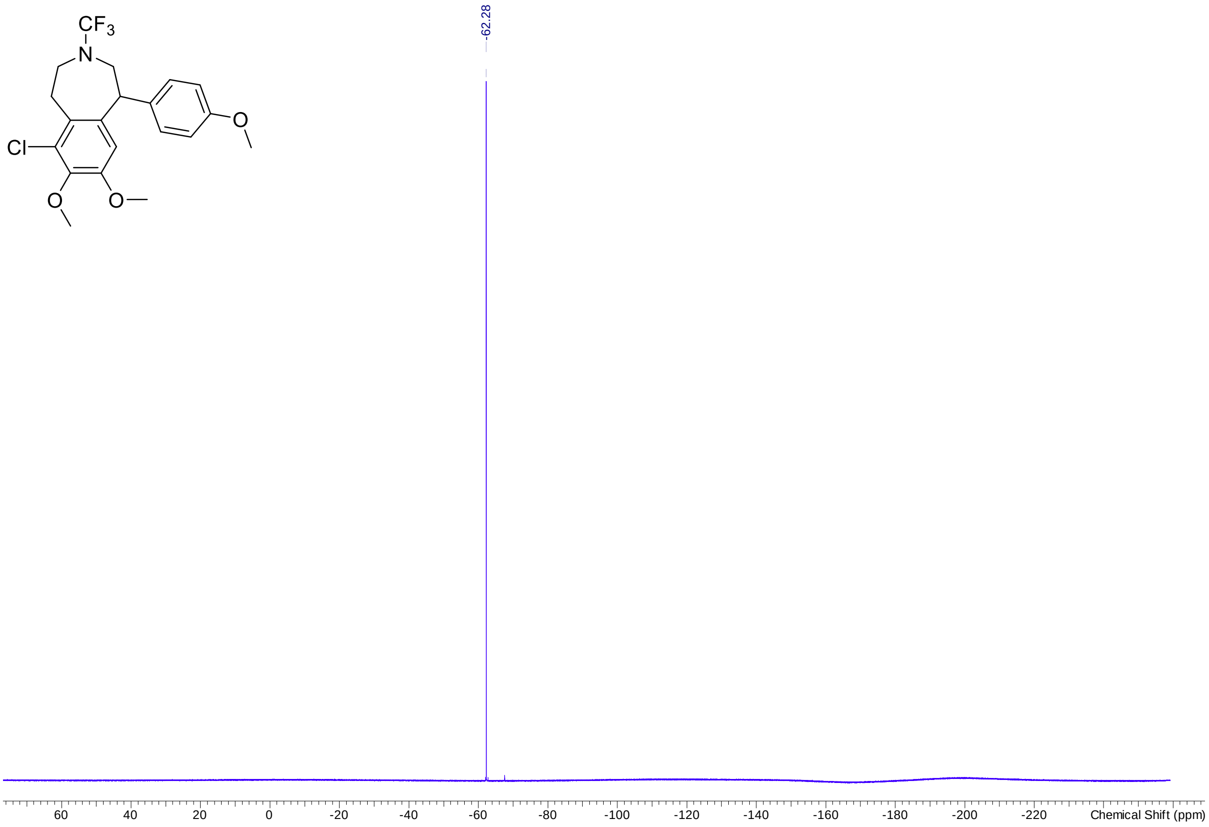

^{13}C NMR (151 MHz, DMSO-d_6) of Compound **2k**

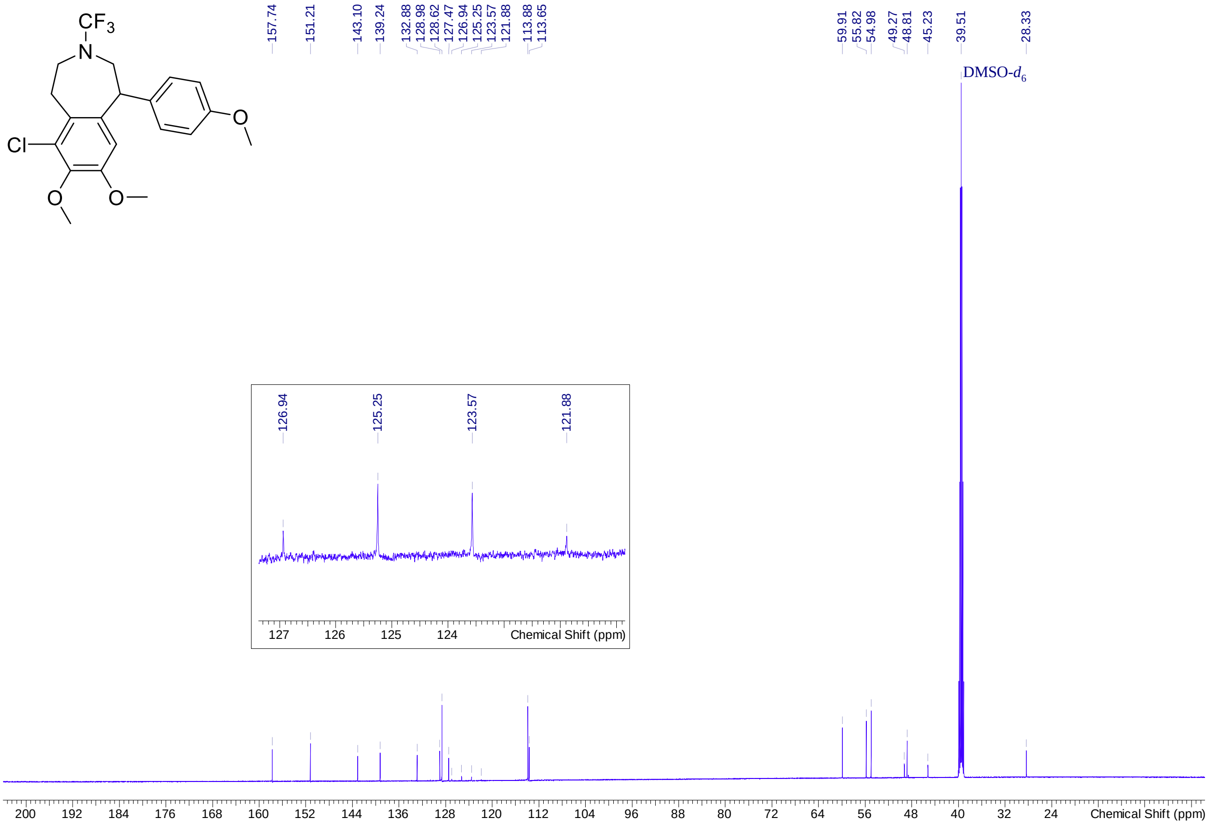

^{1}H NMR (400 MHz, DMSO-d_6) of Compound **2l**

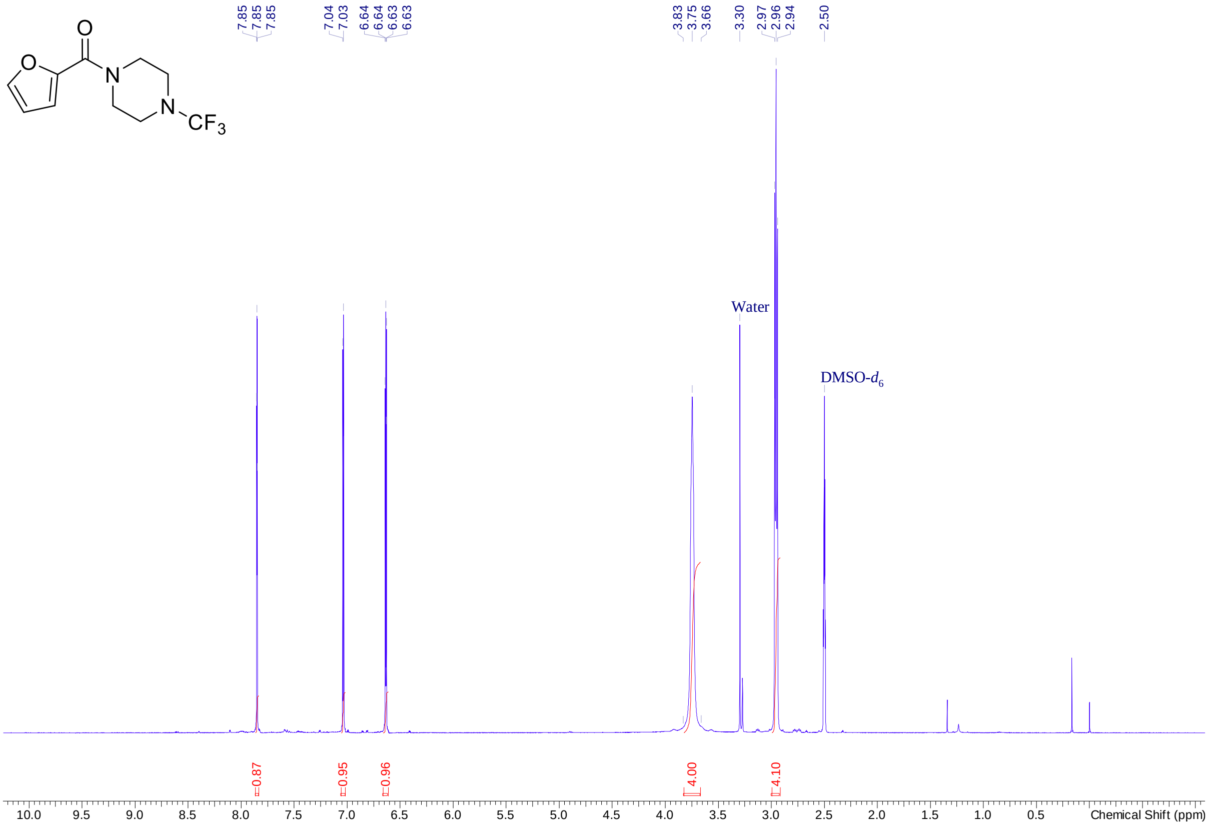

^{19}F NMR (376 MHz, DMSO-d_6) of Compound **2l**

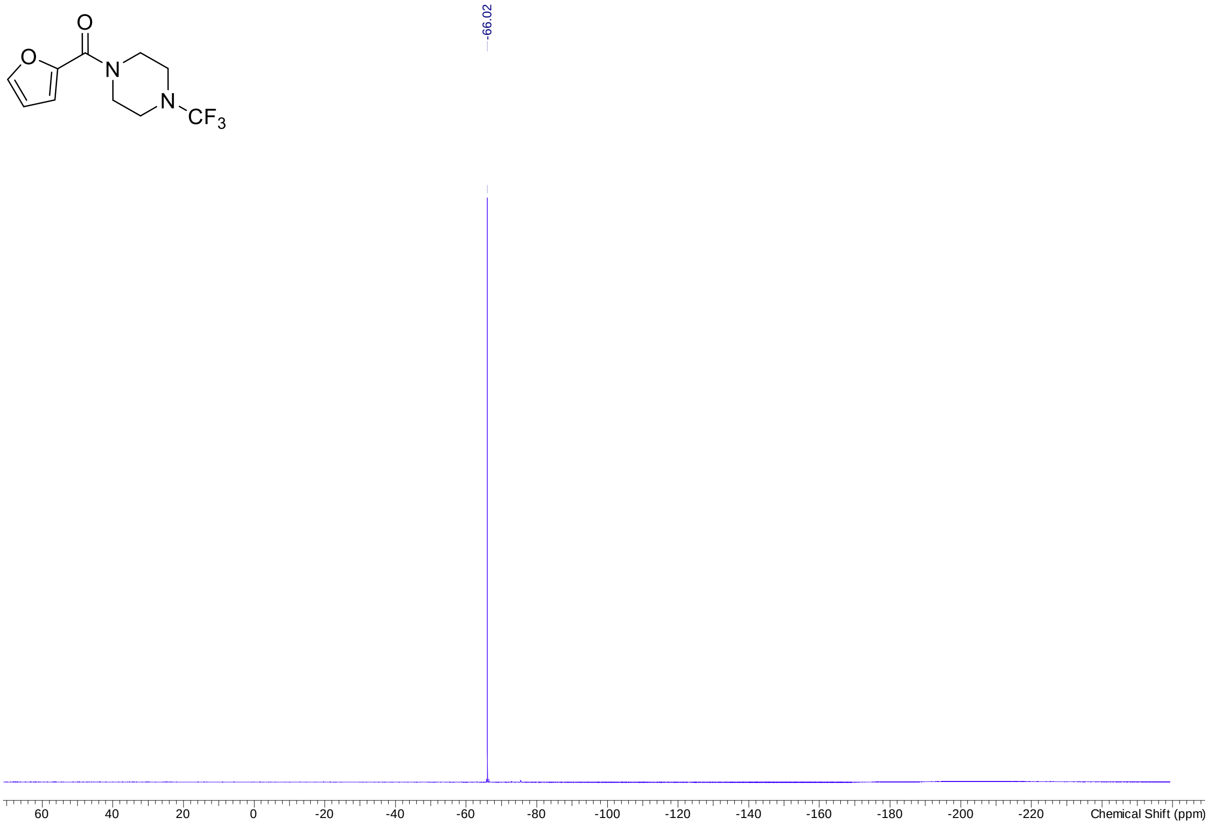

^{13}C NMR (101 MHz, DMSO-d_6) of Compound **2l**

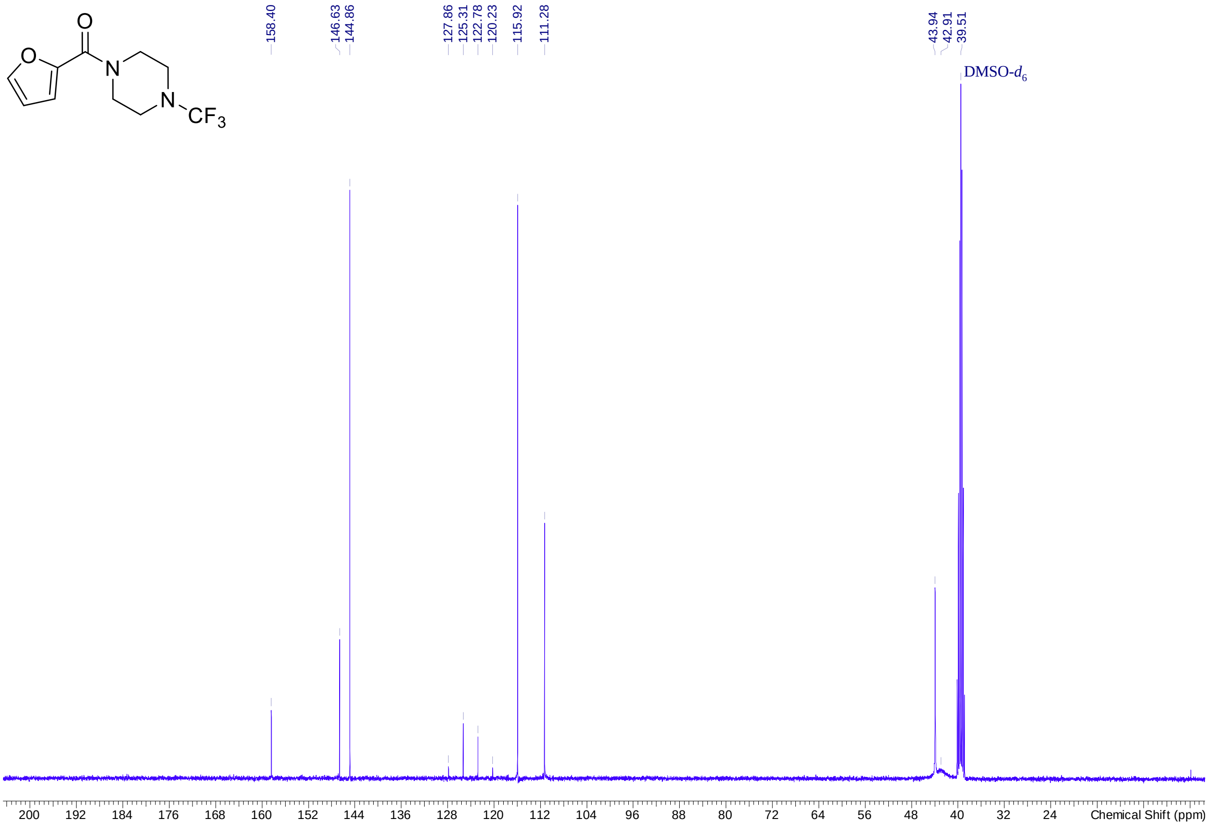

^{1}H NMR (600 MHz, $CDCl_3$) of Compound **2m**

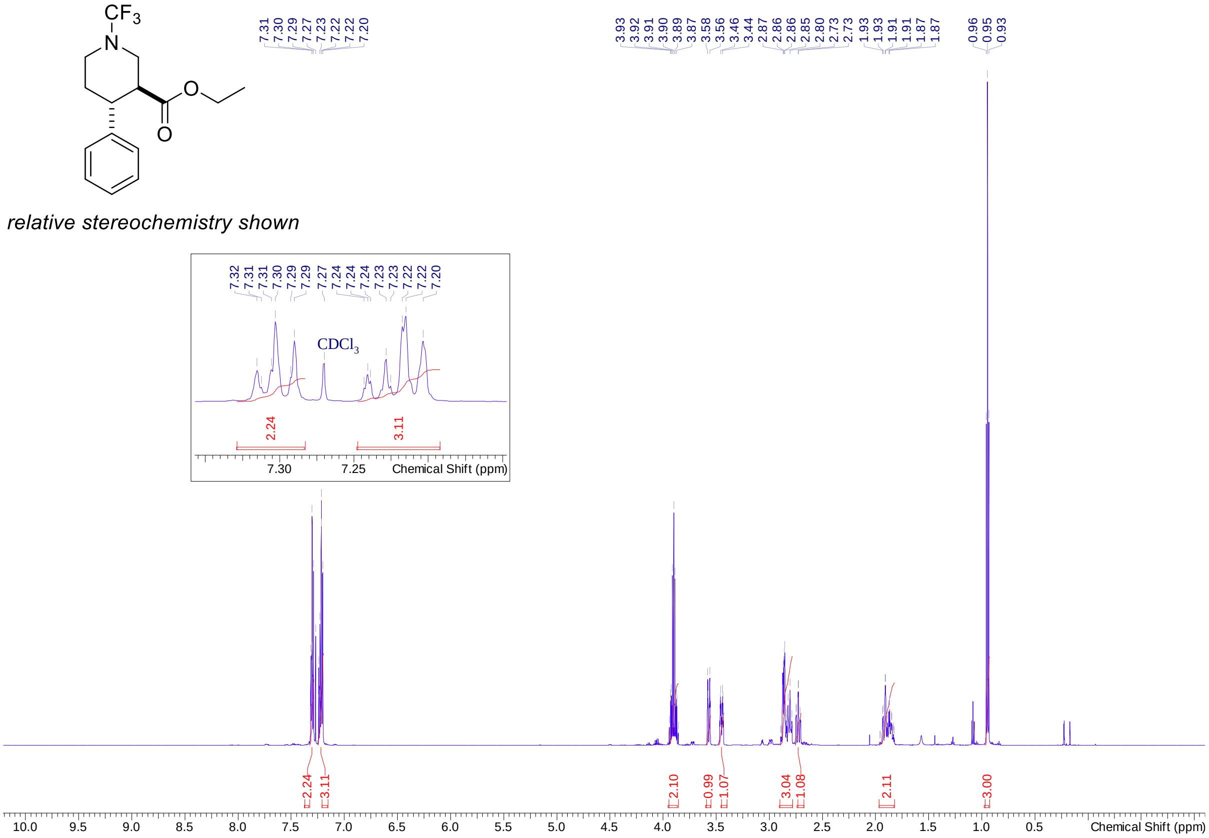

^{19}F NMR (376 MHz, $CDCl_3$) of Compound **2m**

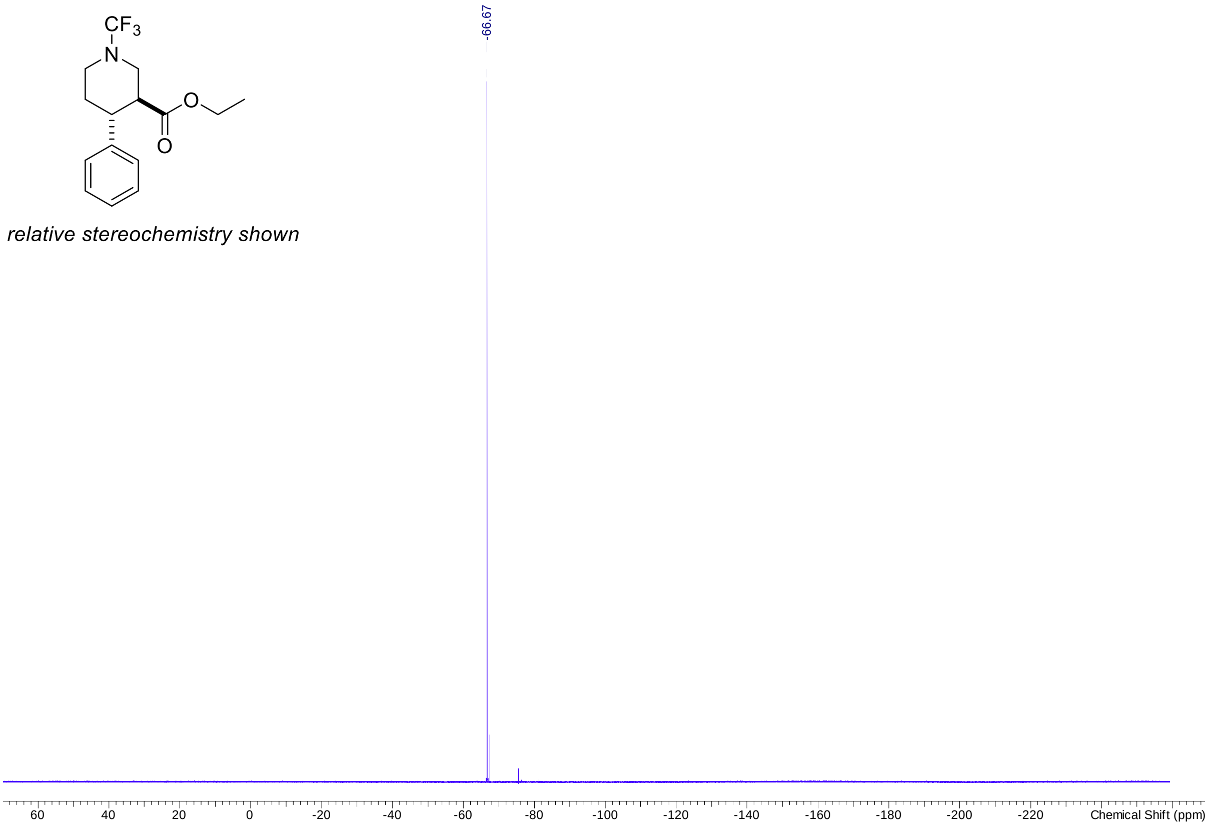

^{13}C NMR (151 MHz, $CDCl_3$) of Compound **2m**

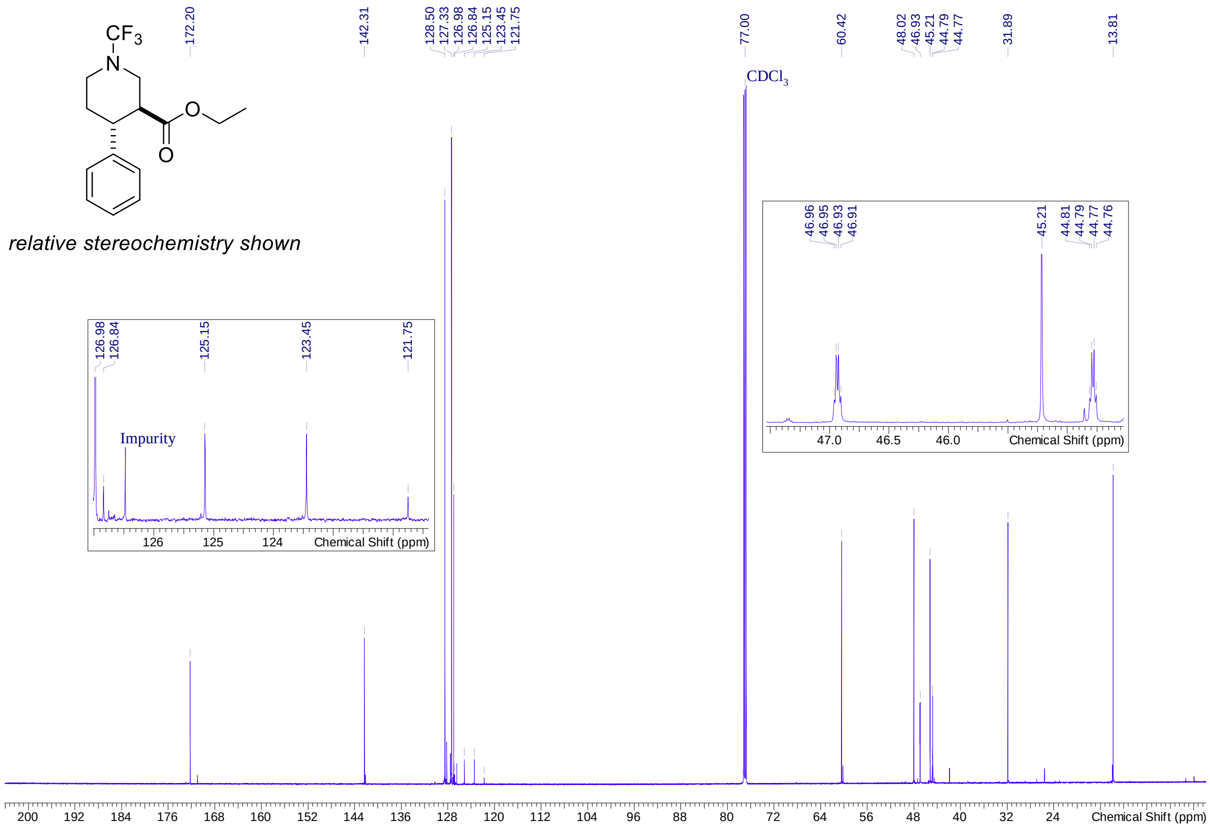

^{1}H NMR (600 MHz, $CDCl_3$) of Compound **2n**

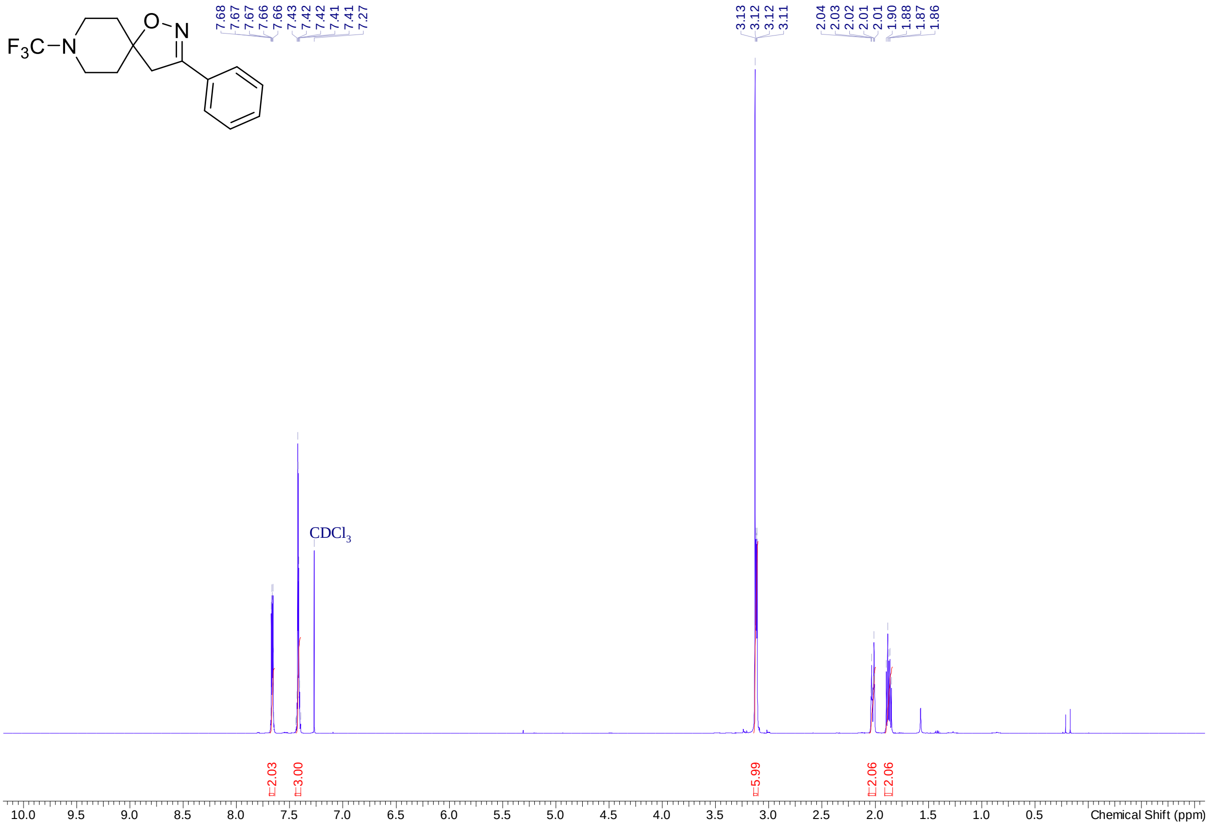

^{19}F NMR (376 MHz, $CDCl_3$) of Compound **2n**

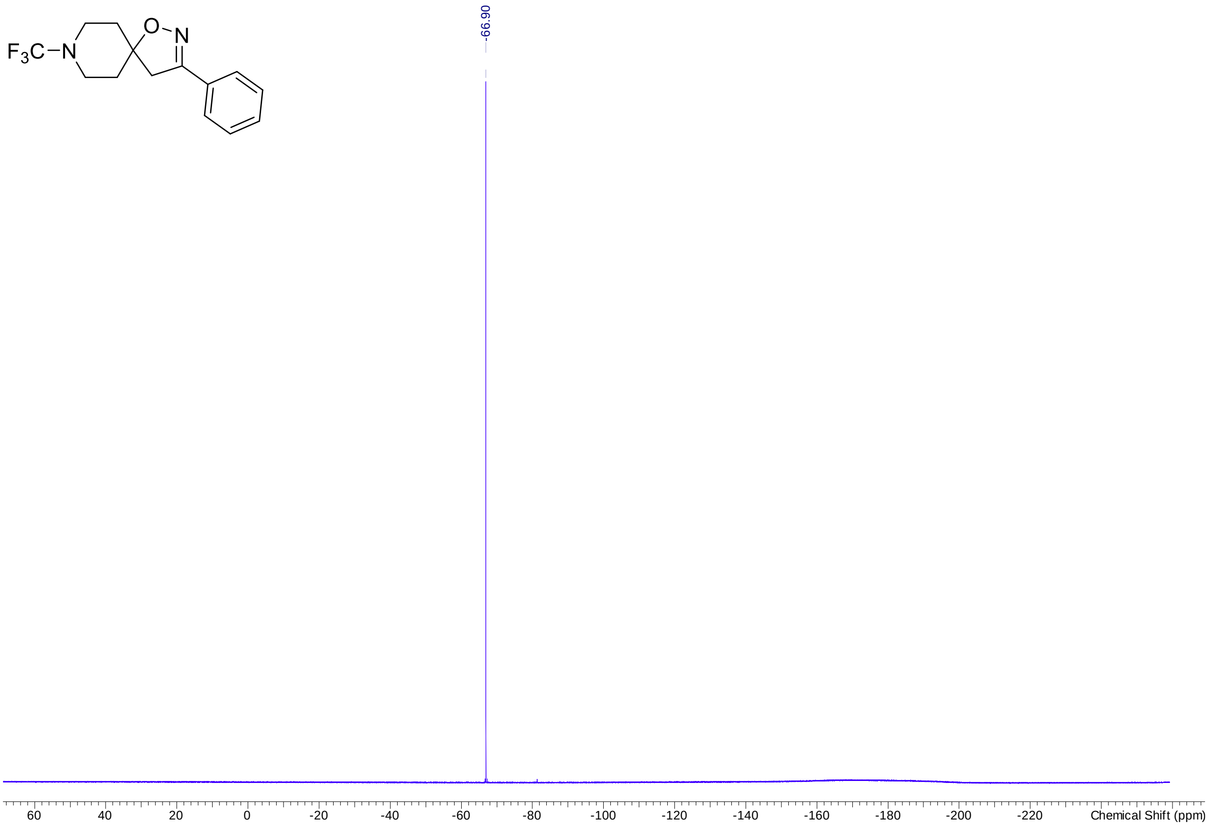

^{13}C NMR (151 MHz, $CDCl_3$) of Compound **2n**

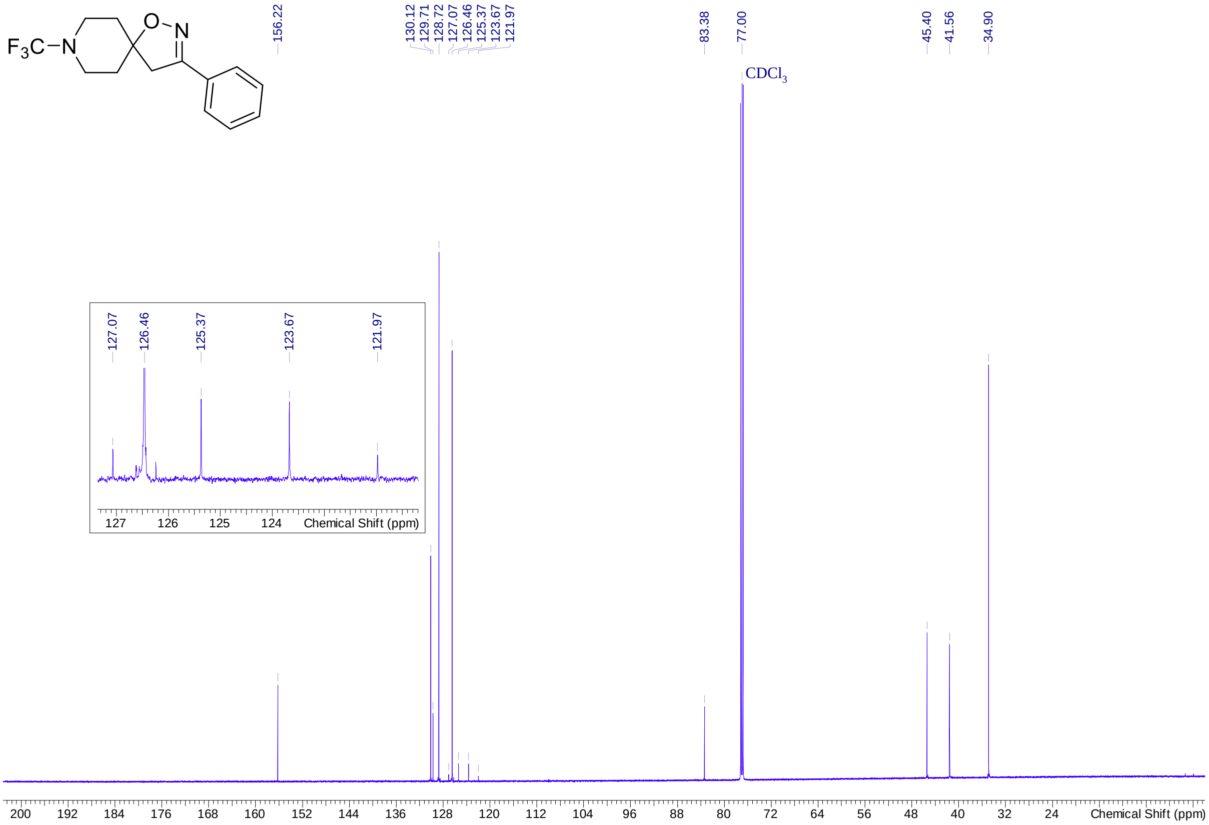

^{1}H NMR (600 MHz, $CDCl_3$) of Compound **2o**

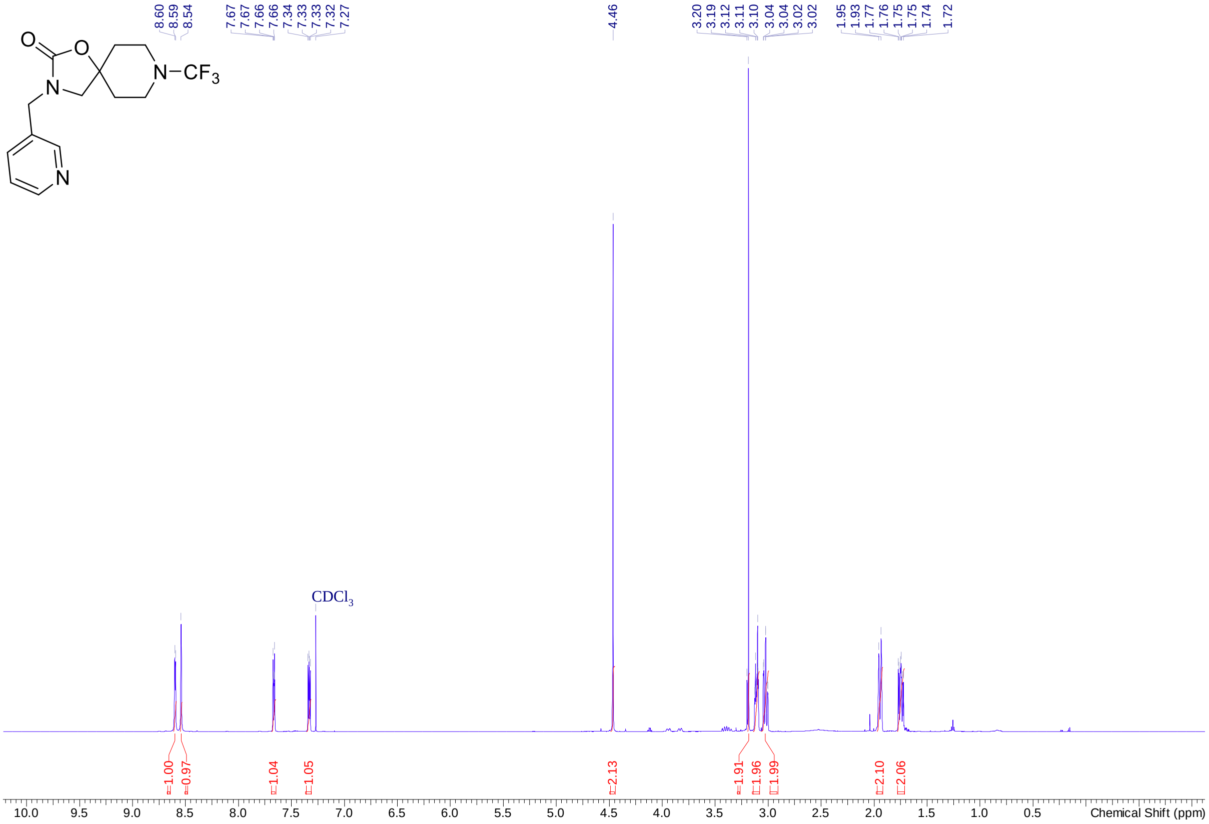

^{19}F NMR (376 MHz, $CDCl_3$) of Compound **2o**

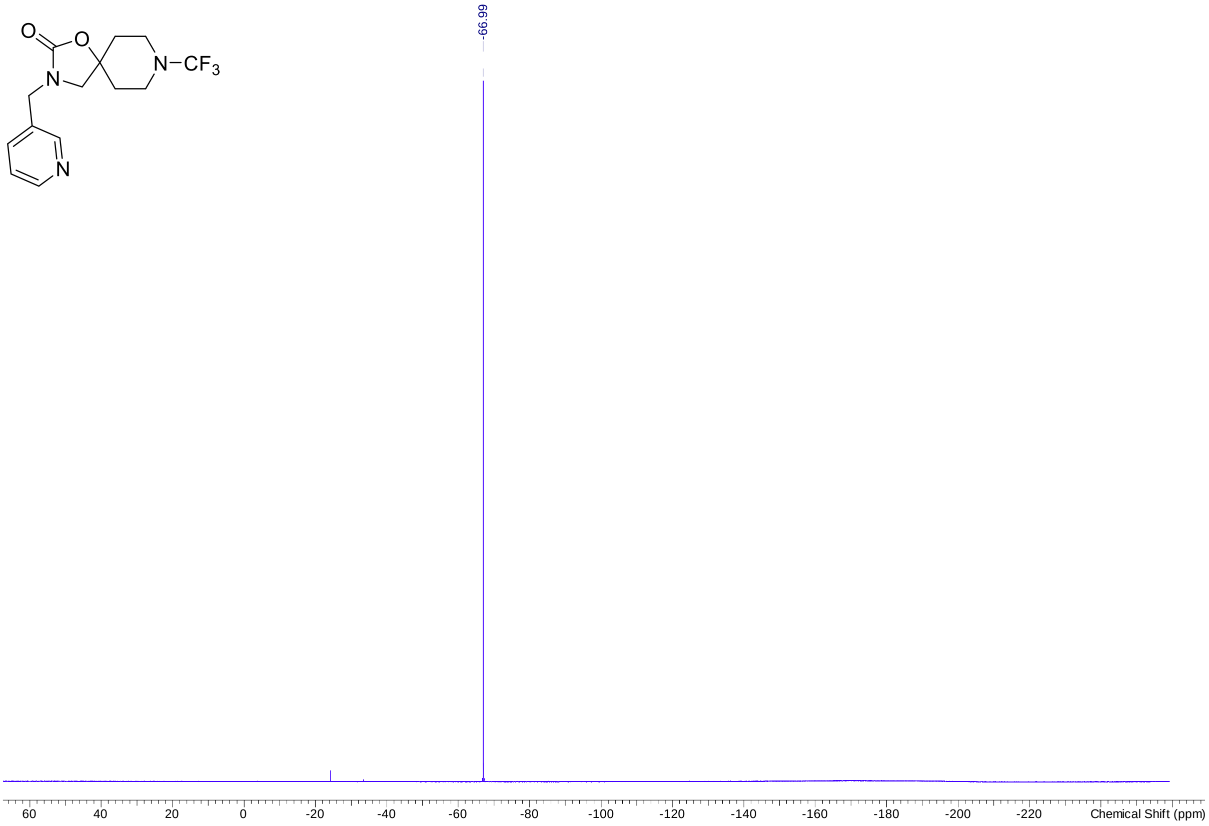

^{13}C NMR (151 MHz, $CDCl_3$) of Compound **2o**

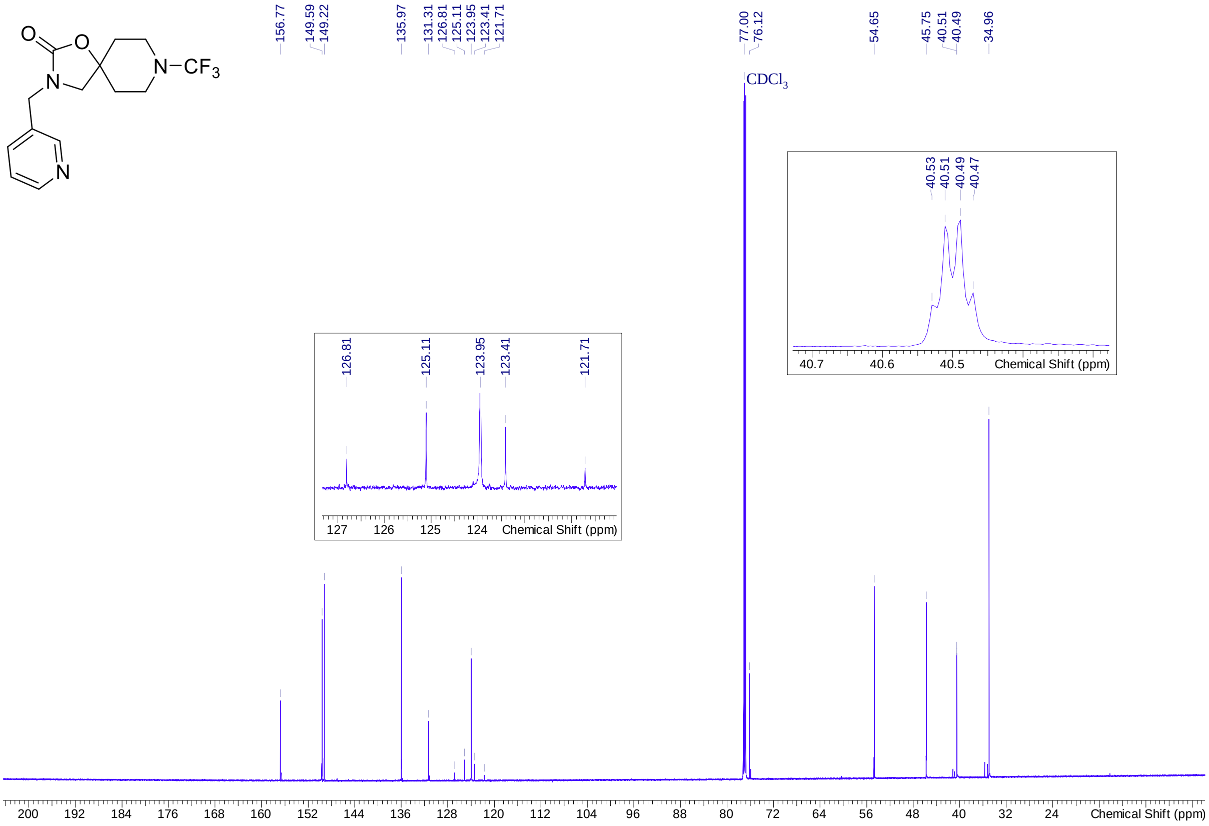

^{1}H NMR (400 MHz, $CDCl_3$) of Compound **2p**

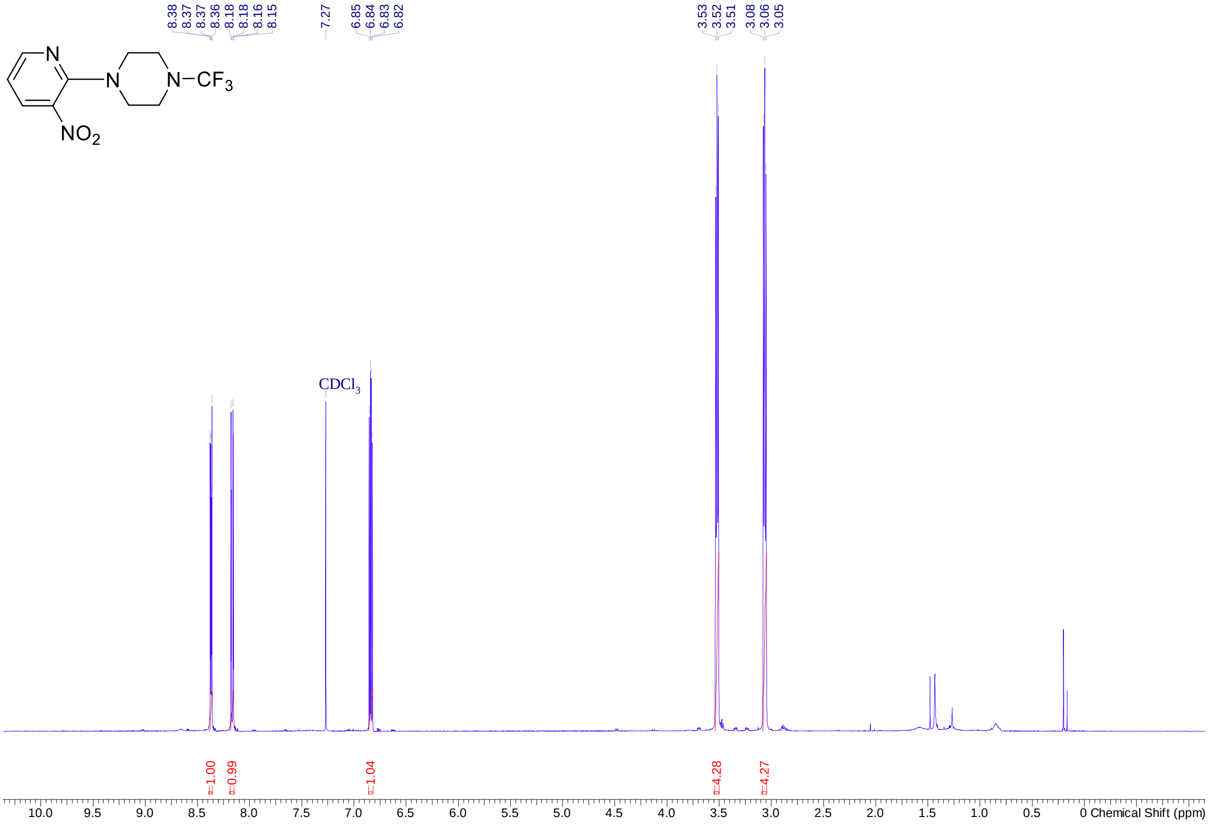

^{19}F NMR (376 MHz, $CDCl_3$) of Compound **2p**

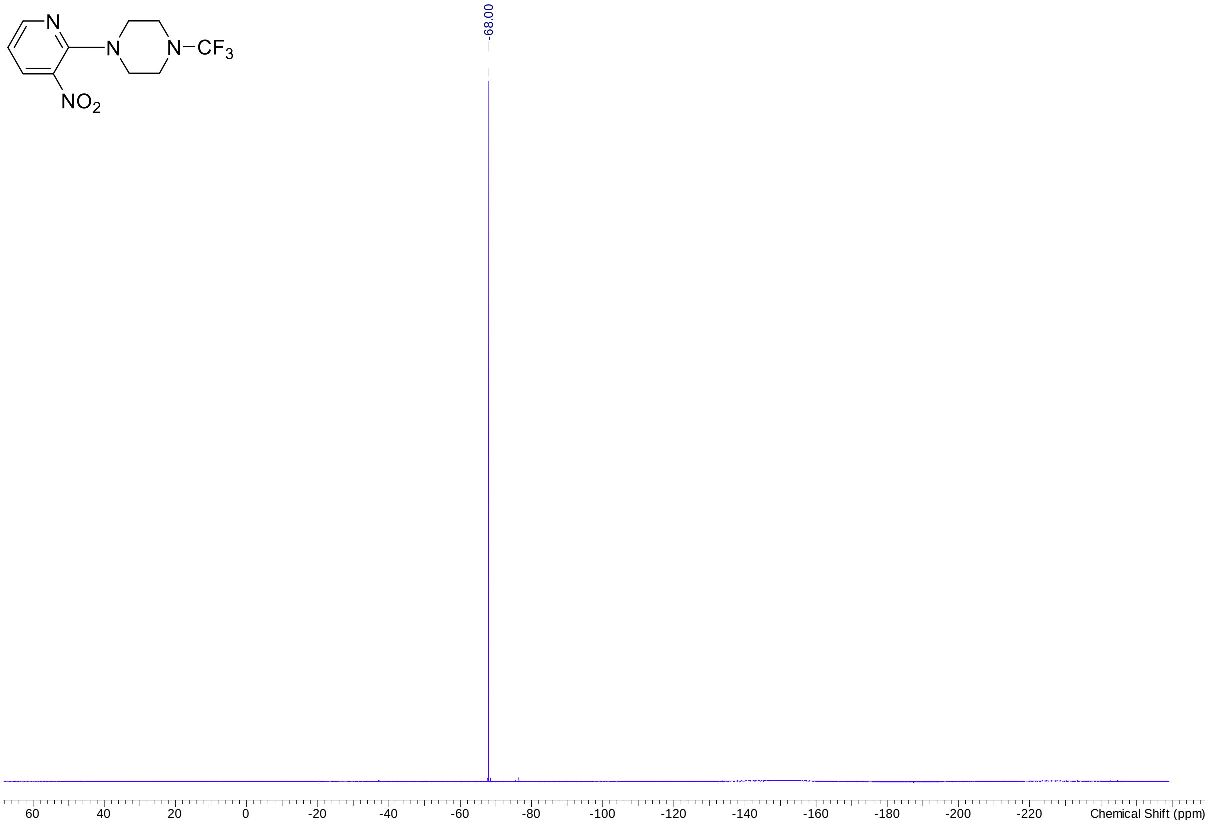

^{13}C NMR (101 MHz, $CDCl_3$) of Compound **2p**

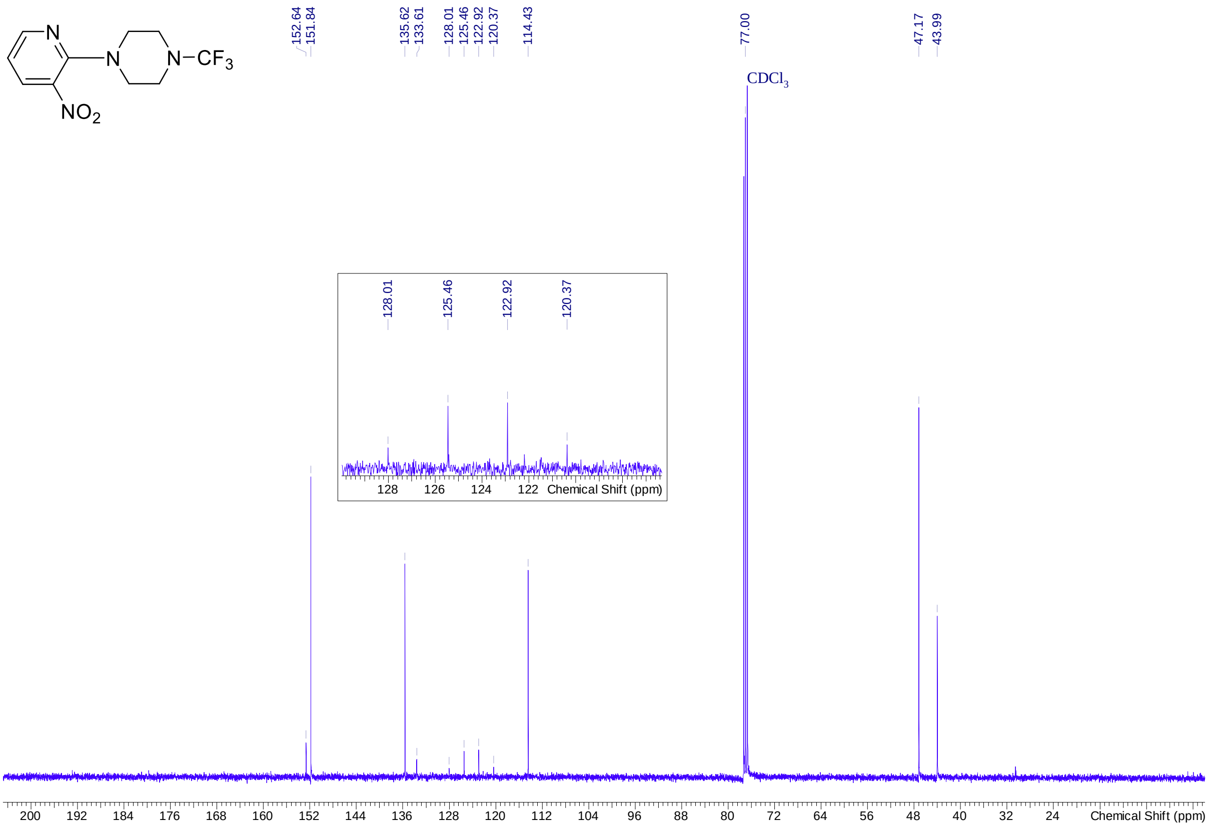

^{1}H NMR (400 MHz, $CDCl_3$) of Compound **2q**

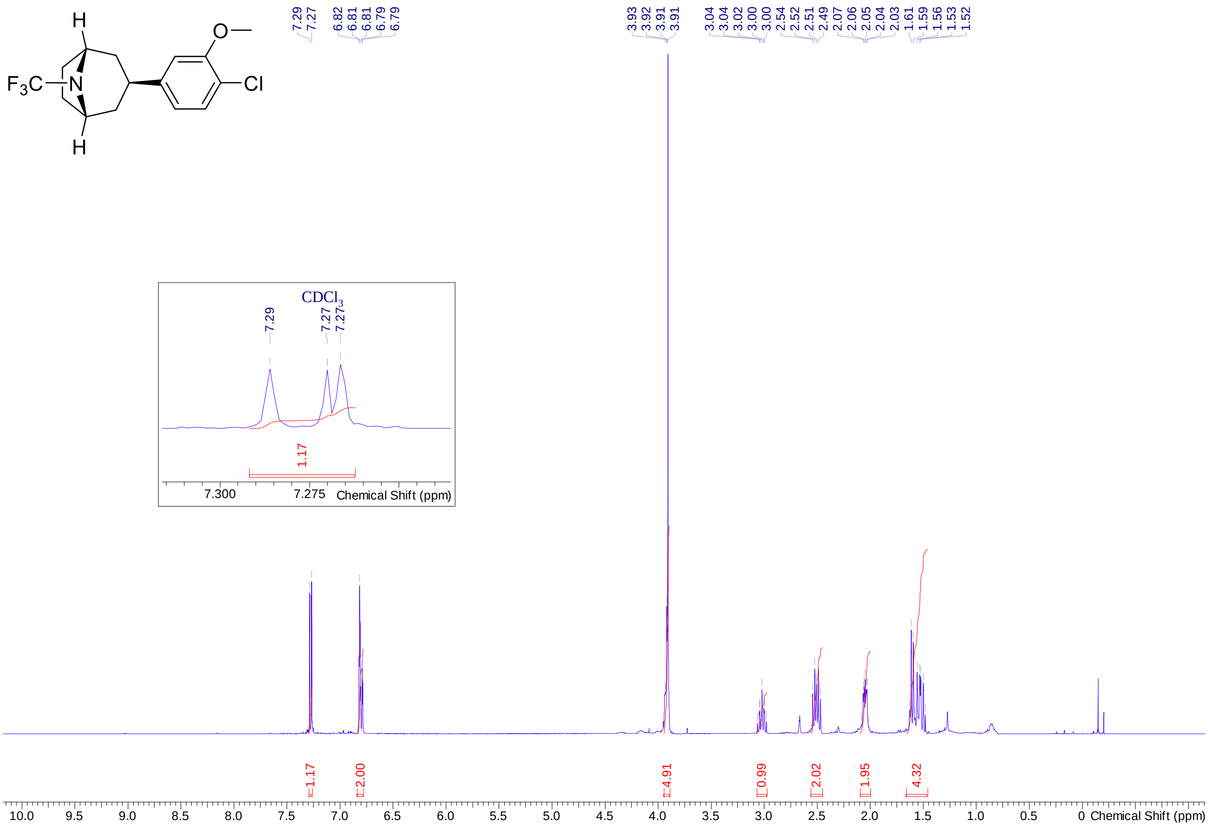

^{19}F NMR (376 MHz, $CDCl_3$) of Compound **2q**

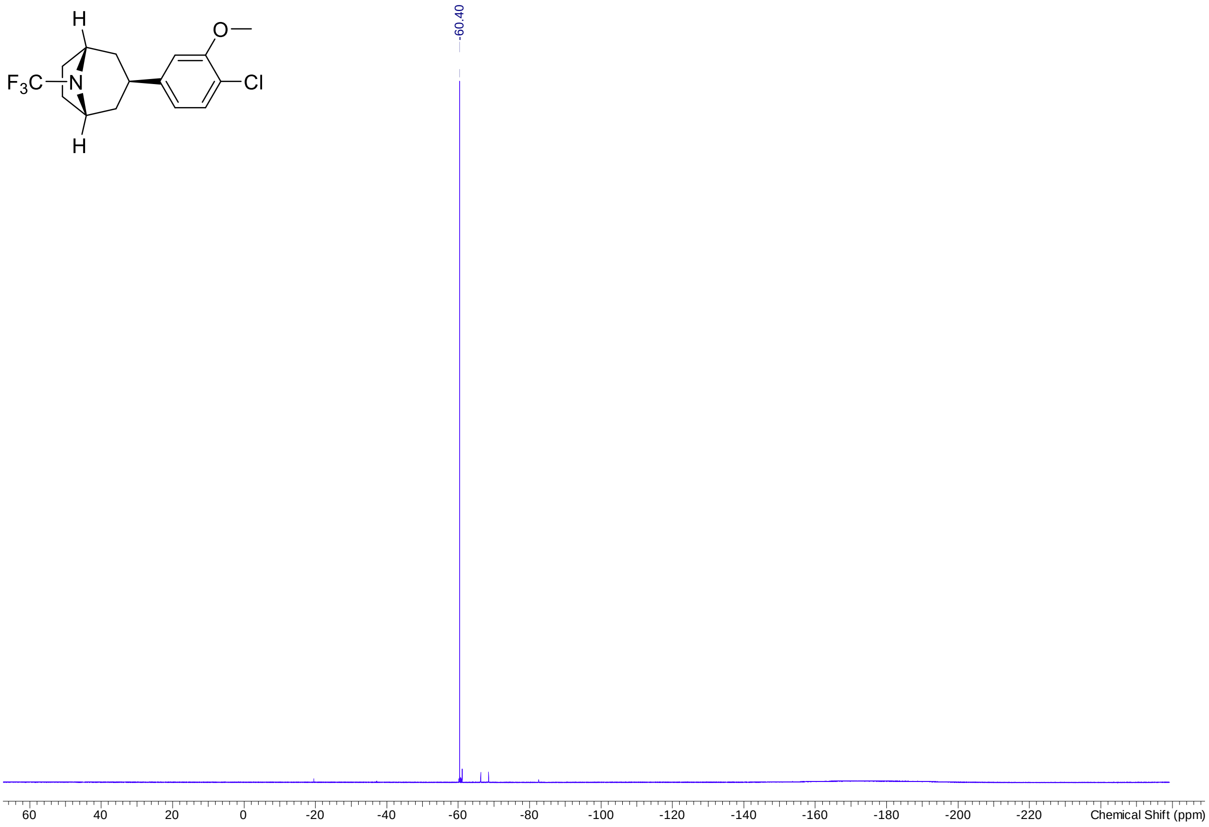

^{13}C NMR (151 MHz, $CDCl_3$) of Compound **2q**

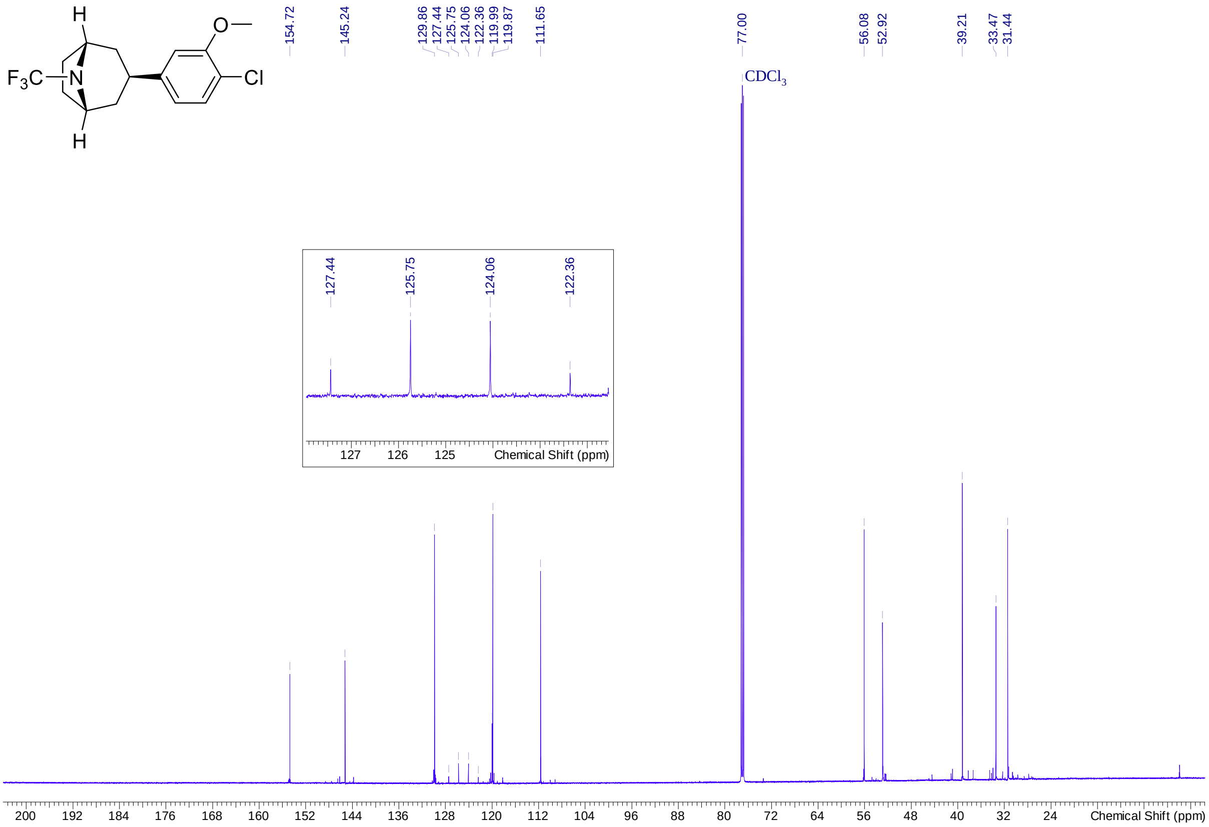

^{1}H NMR (400 MHz, $CDCl_3$) of Compound **2r**

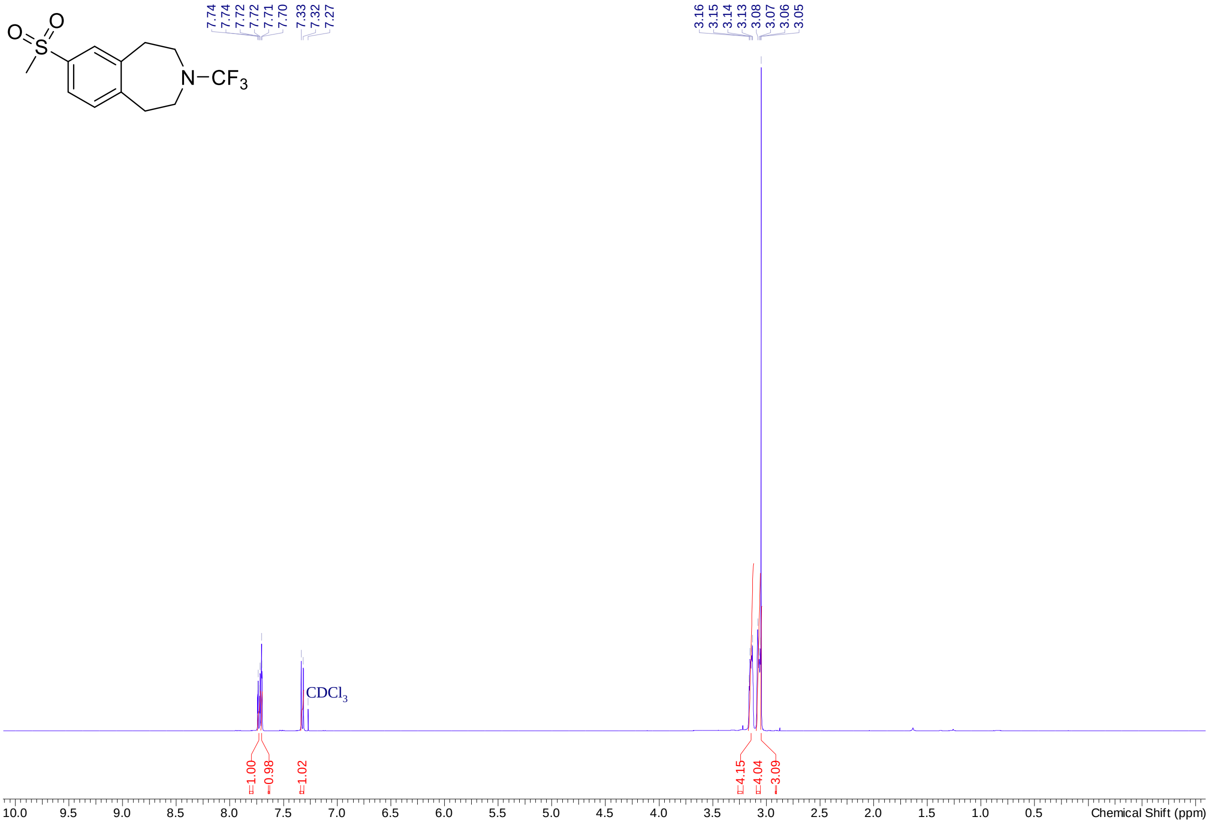

^{19}F NMR (376 MHz, $CDCl_3$) of Compound **2r**

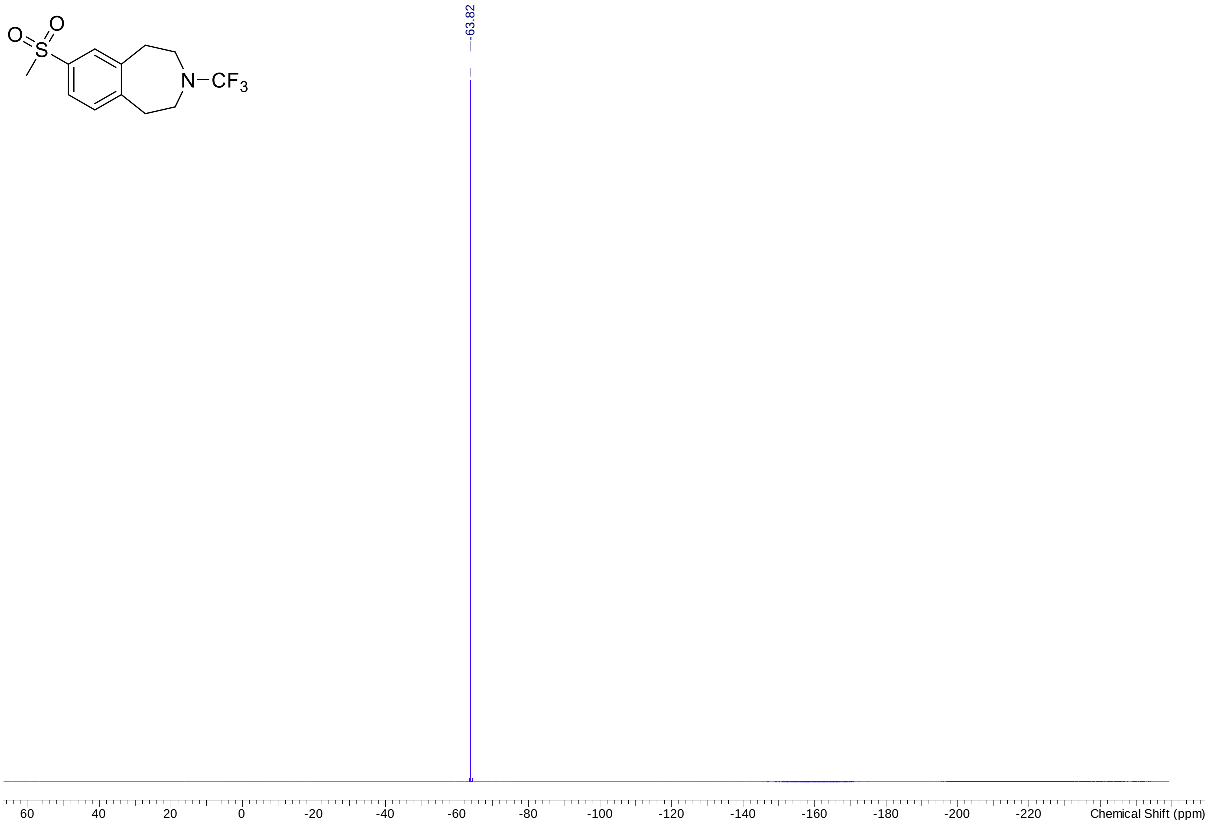

^{13}C NMR (101 MHz, $CDCl_3$) of Compound **2r**

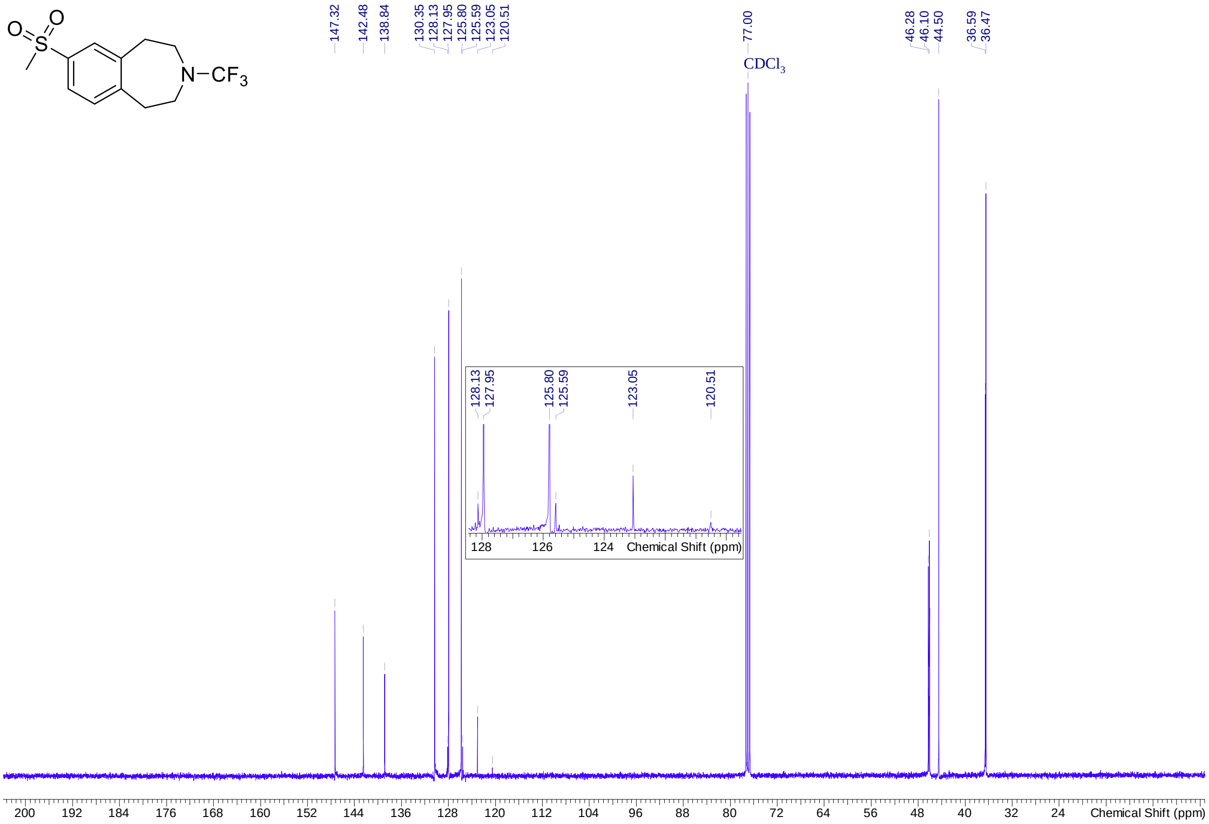

^{1}H NMR (400 MHz, $CDCl_3$) of Compound **2s**

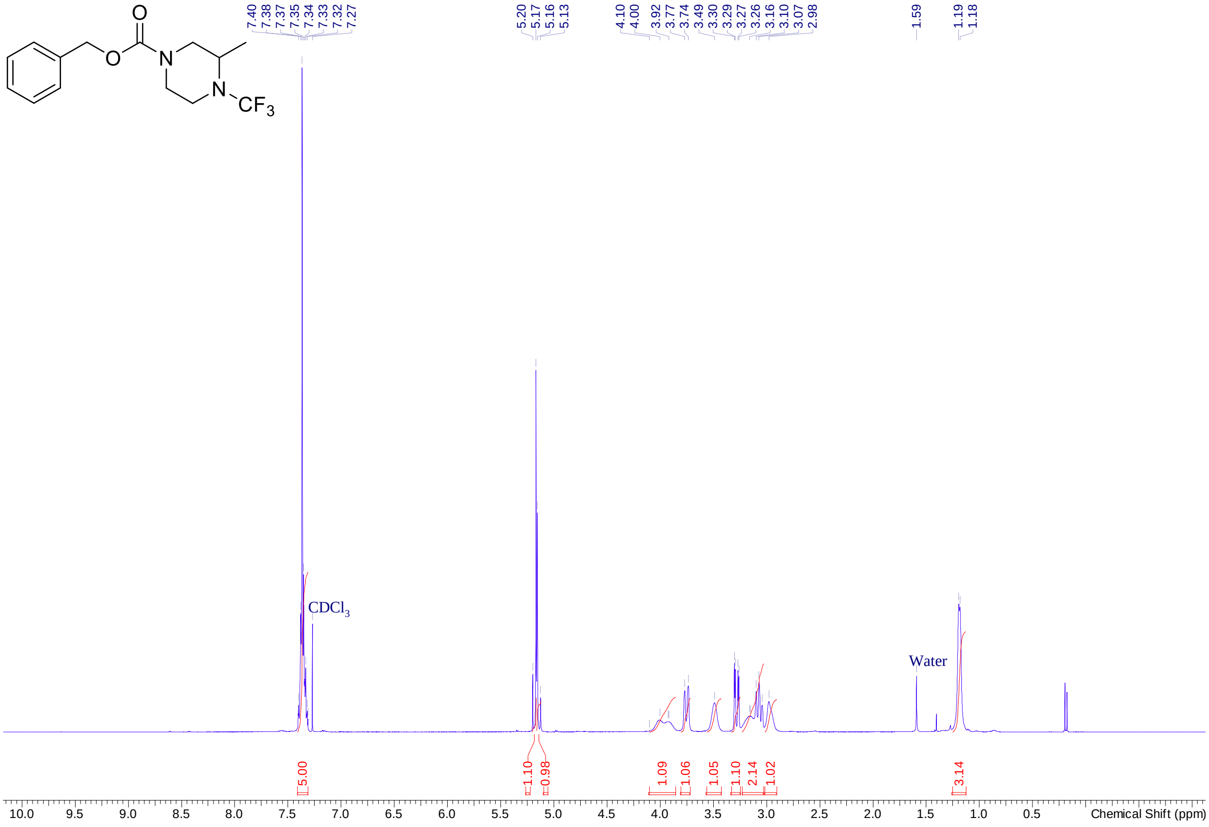

^{19}F NMR (376 MHz, $CDCl_3$) of Compound **2s**

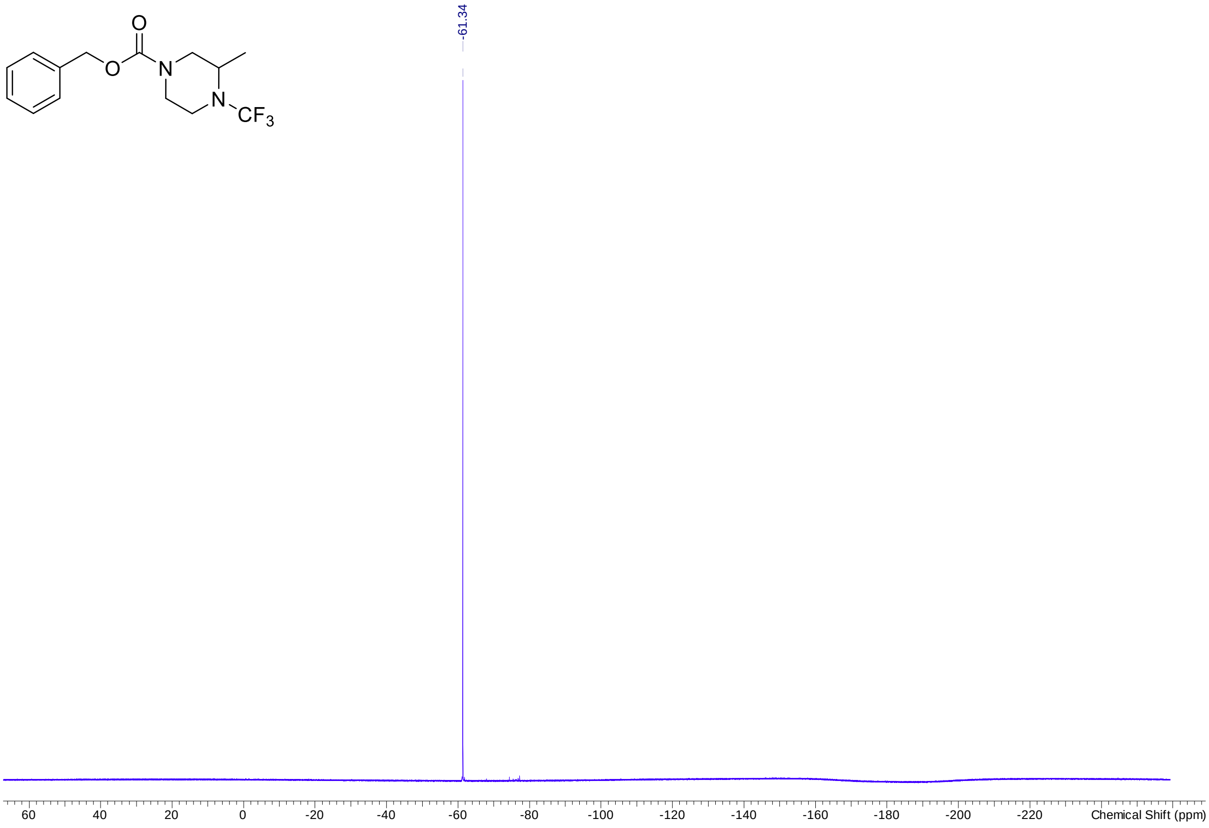

^{13}C NMR (151 MHz, $CDCl_3$) of Compound **2s**

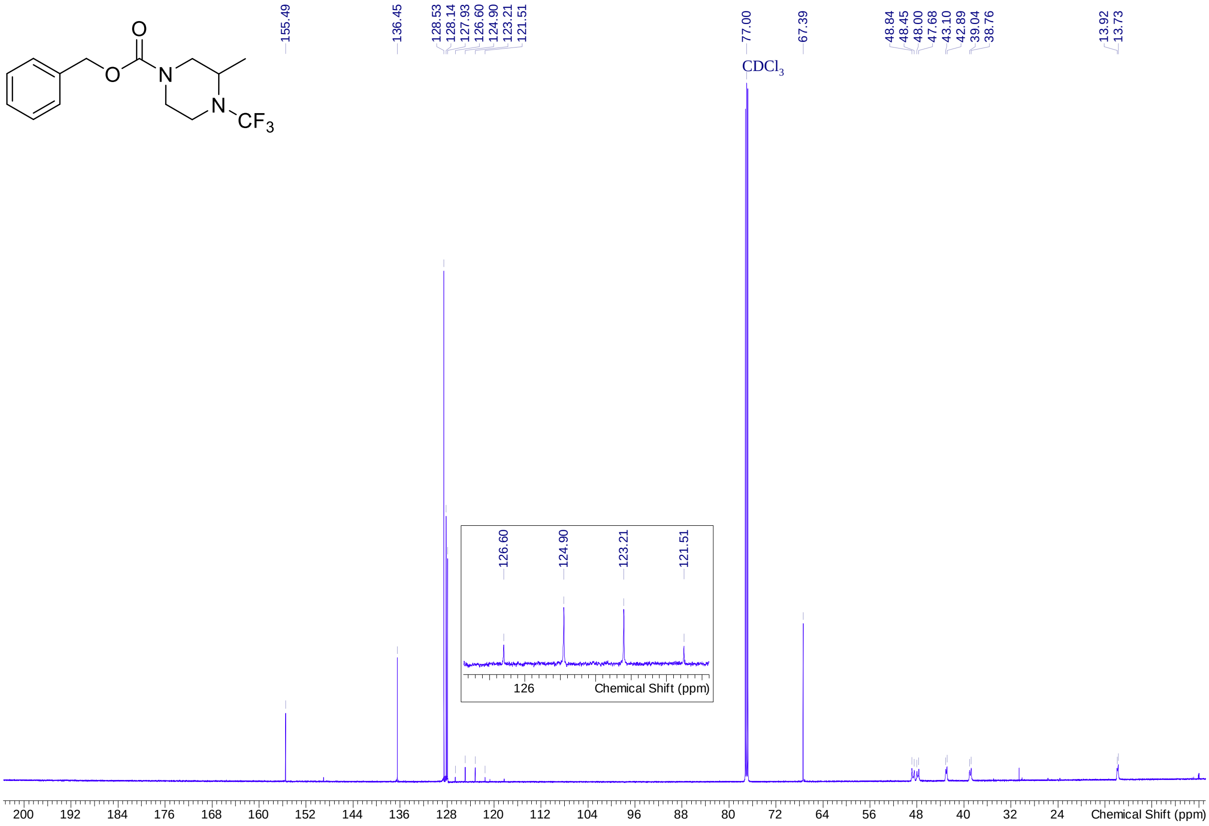

^{1}H NMR (400 MHz, $CDCl_3$) of Compound **2t**

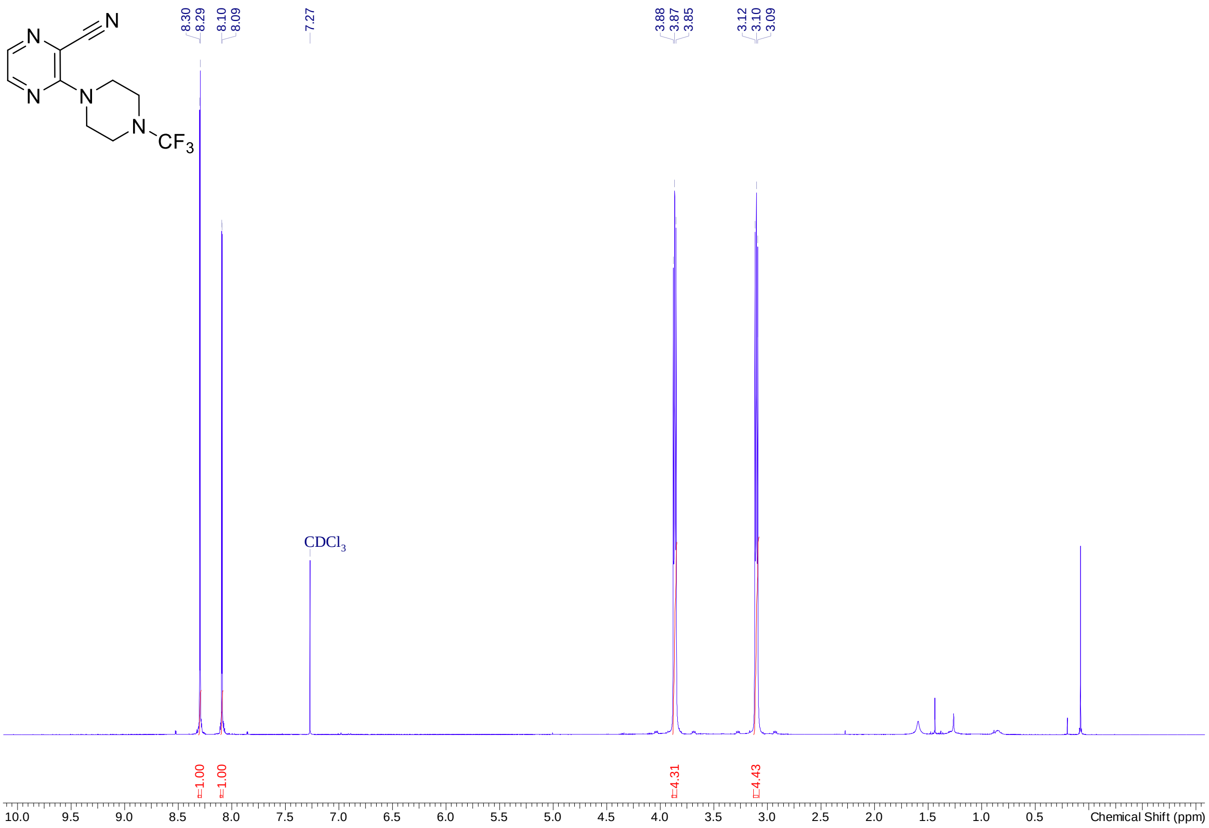

^{19}F NMR (376 MHz, $CDCl_3$) of Compound **2t**

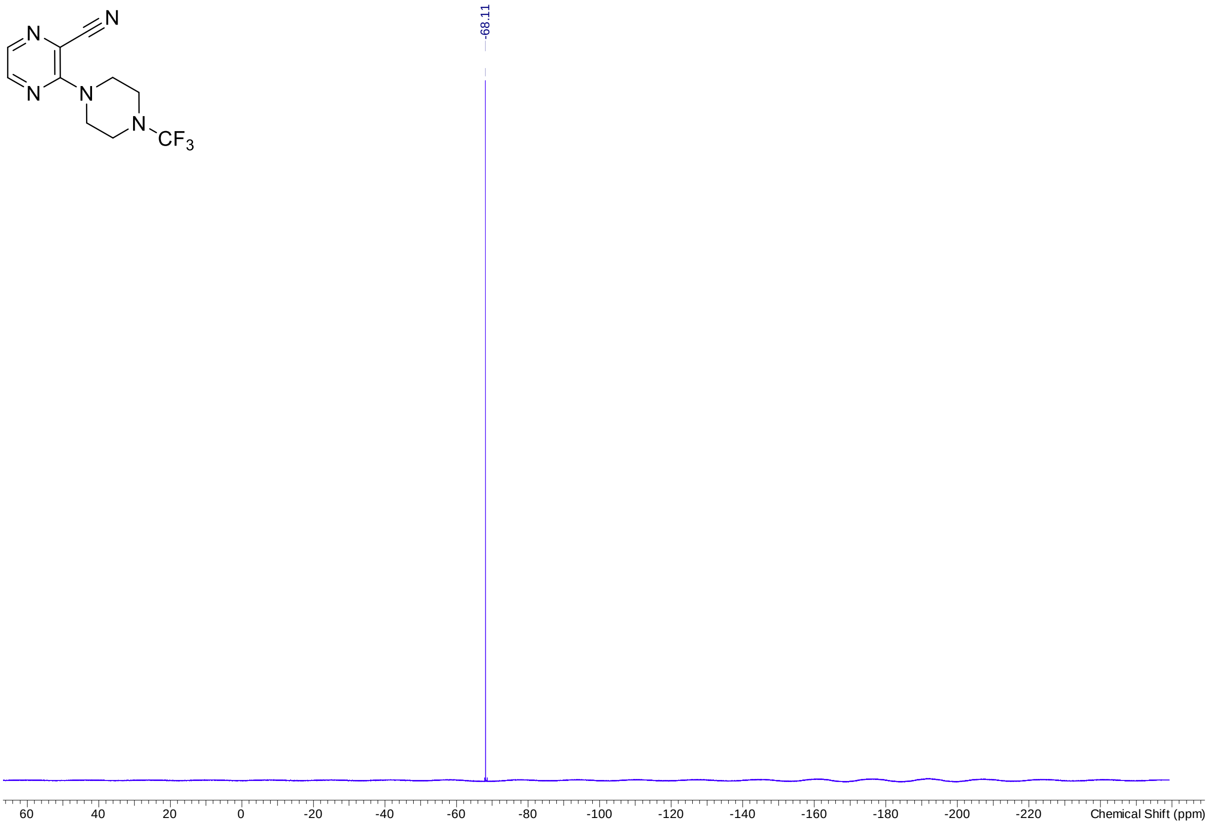

^{13}C NMR (101 MHz, $CDCl_3$) of Compound **2t**

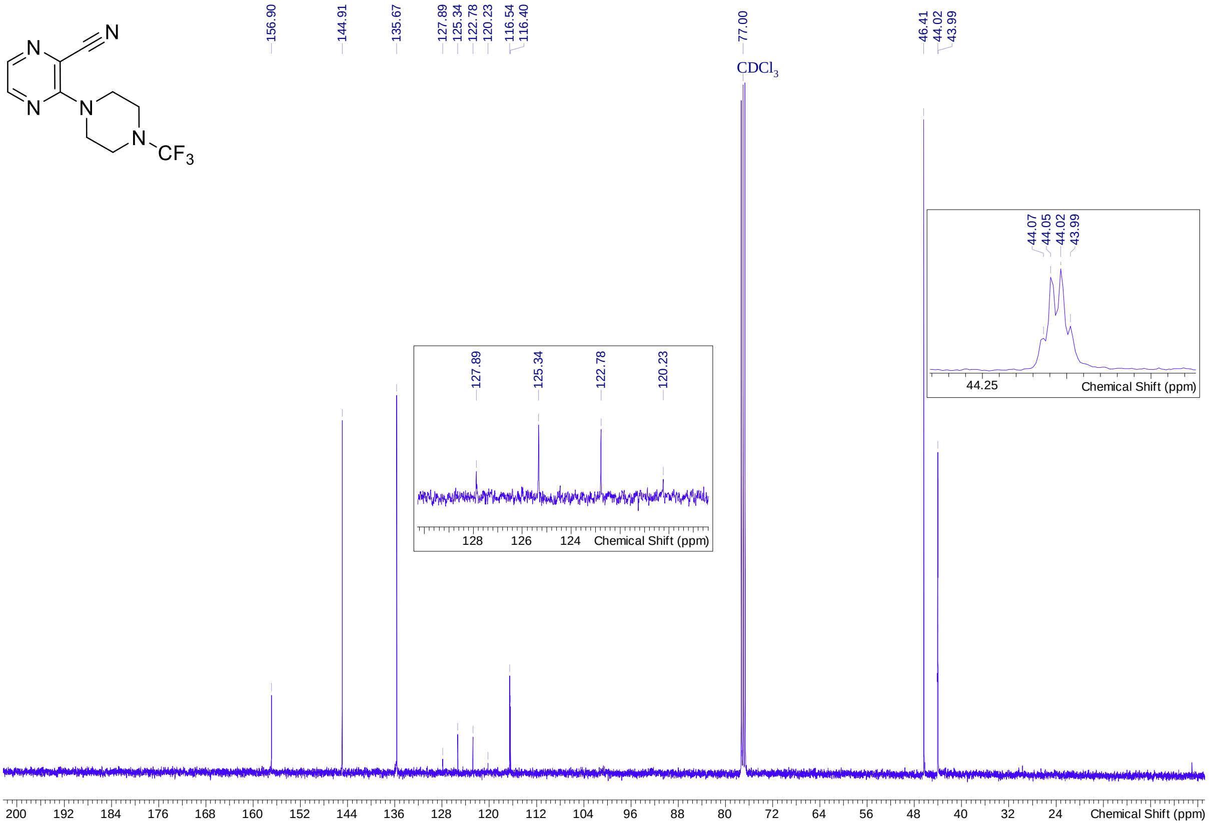

^{1}H NMR (400 MHz, $CDCl_3$) of Compound **2u**

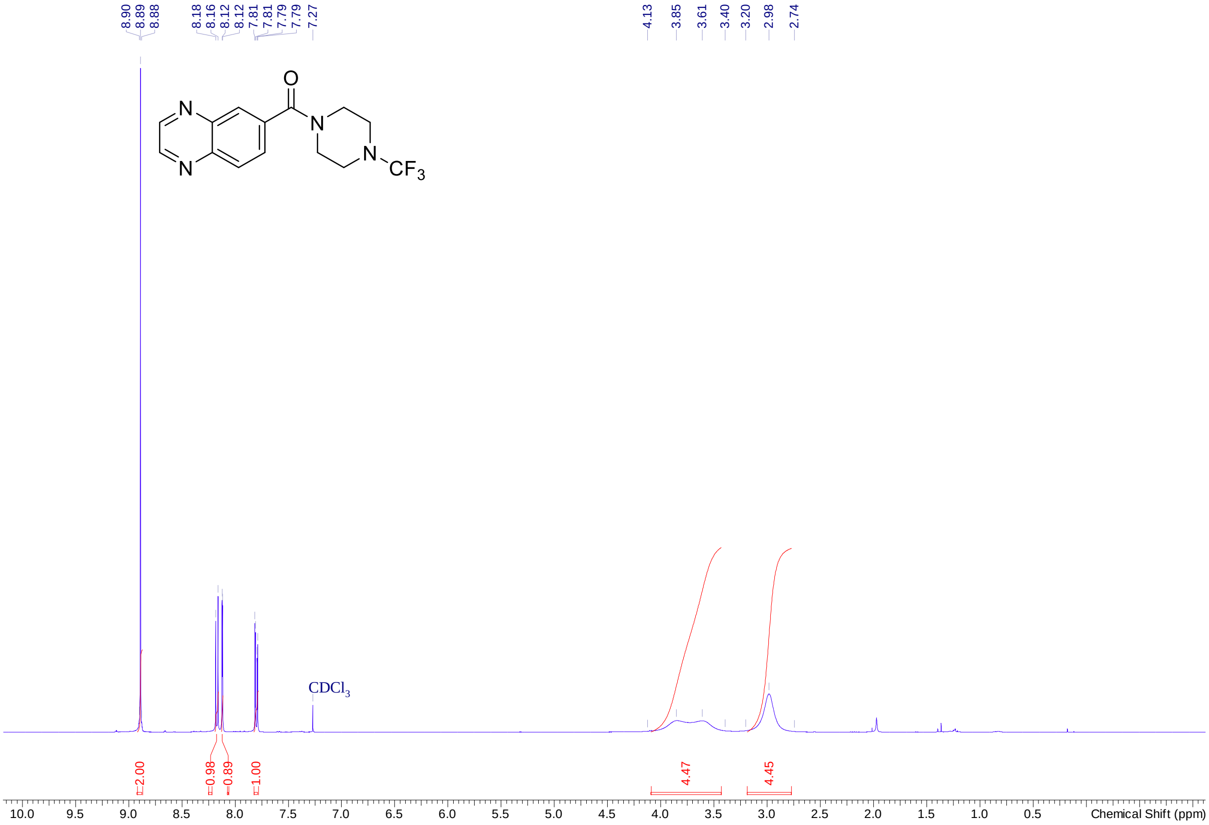

^{19}F NMR (376 MHz, $CDCl_3$) of Compound **2u**

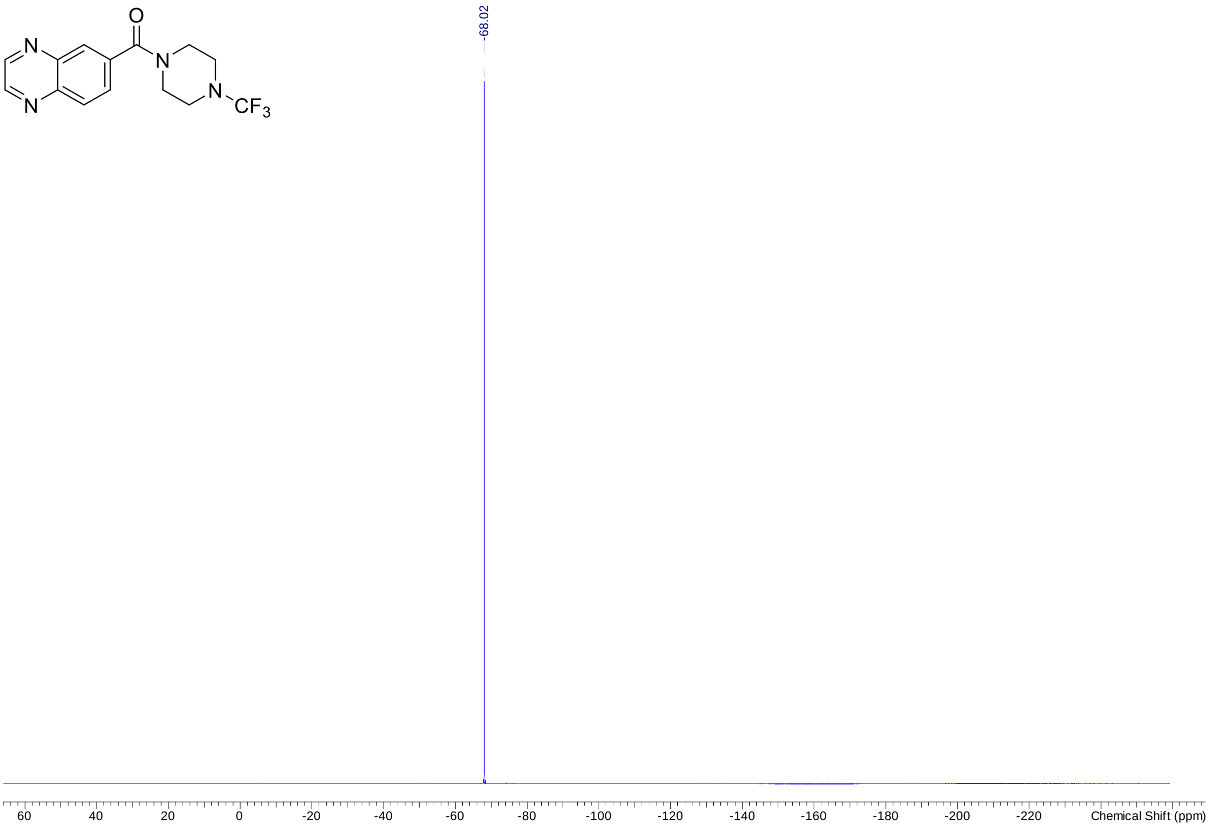

^{13}C NMR (101 MHz, $CDCl_3$) of Compound **2u**

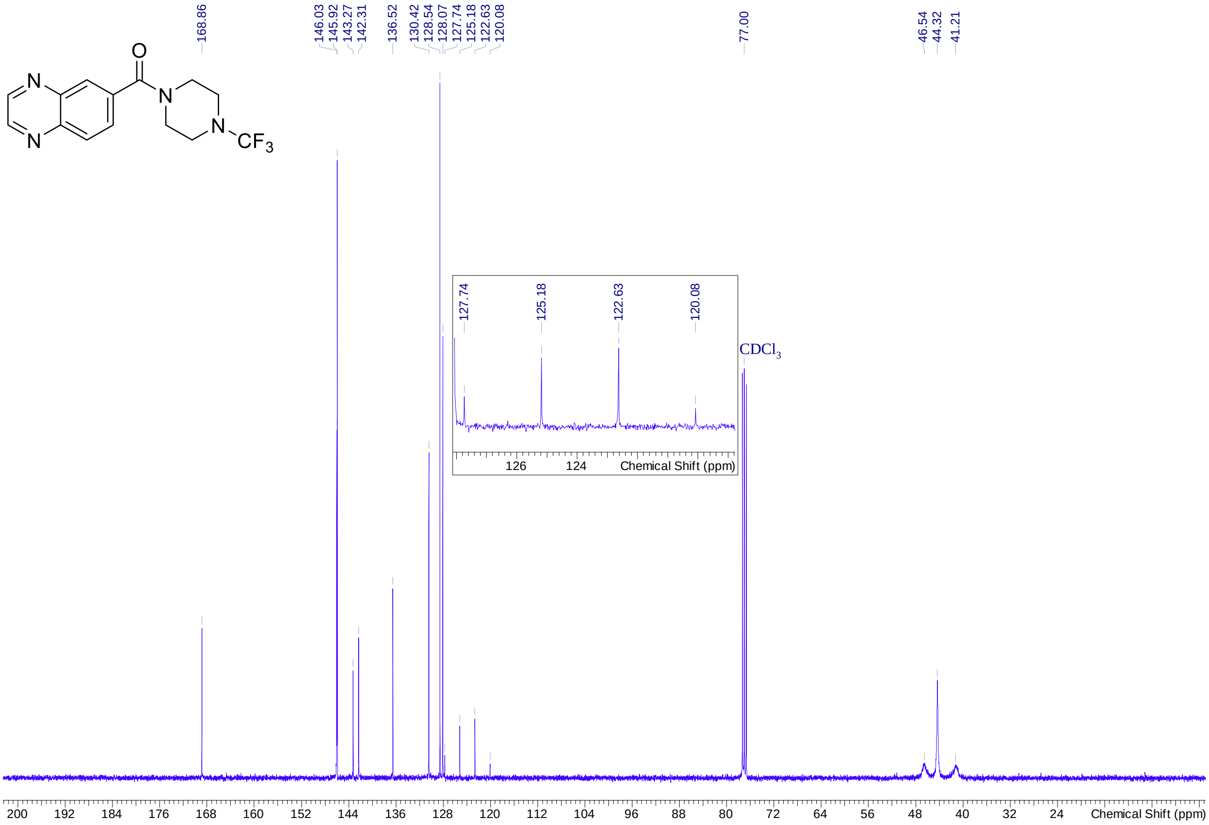

^{1}H NMR (400 MHz, $CDCl_3$) of Compound **2v**

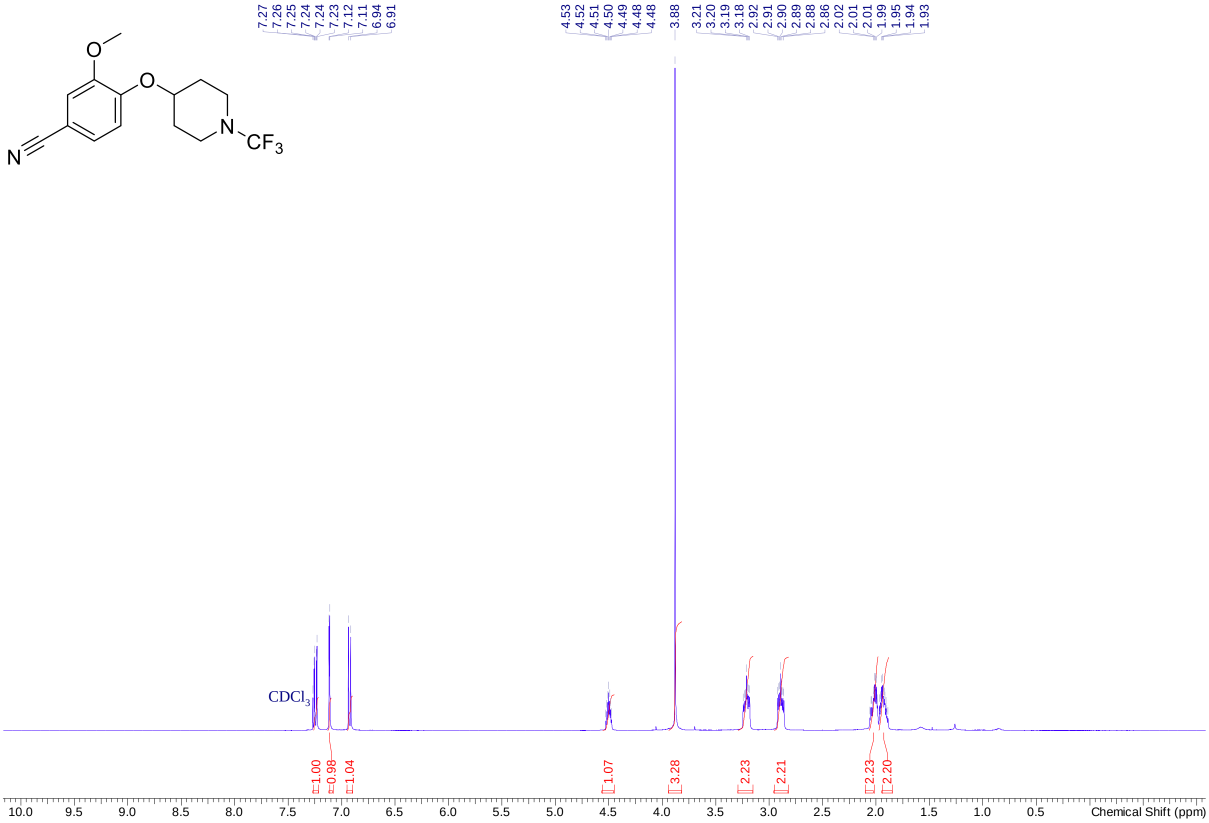

^{19}F NMR (376 MHz, $CDCl_3$) of Compound **2v**

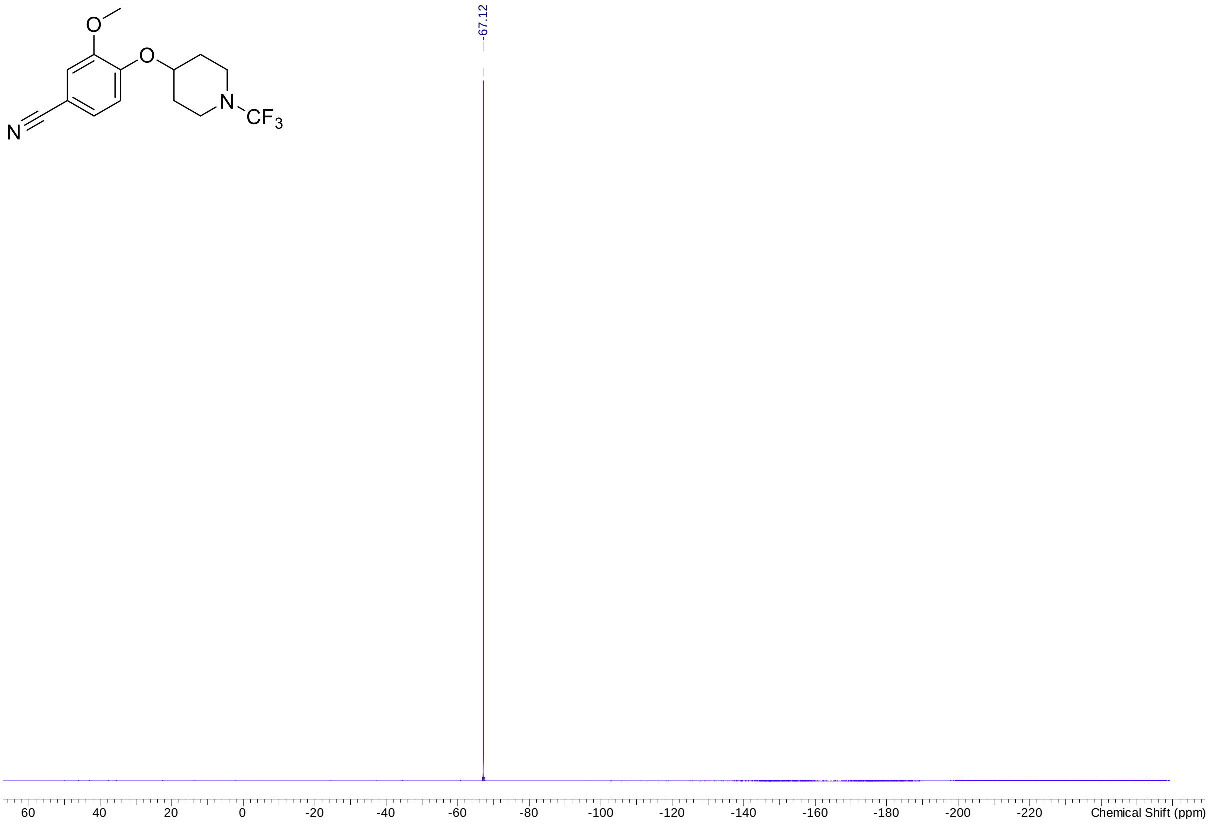

^{13}C NMR (101 MHz, $CDCl_3$) of Compound **2v**

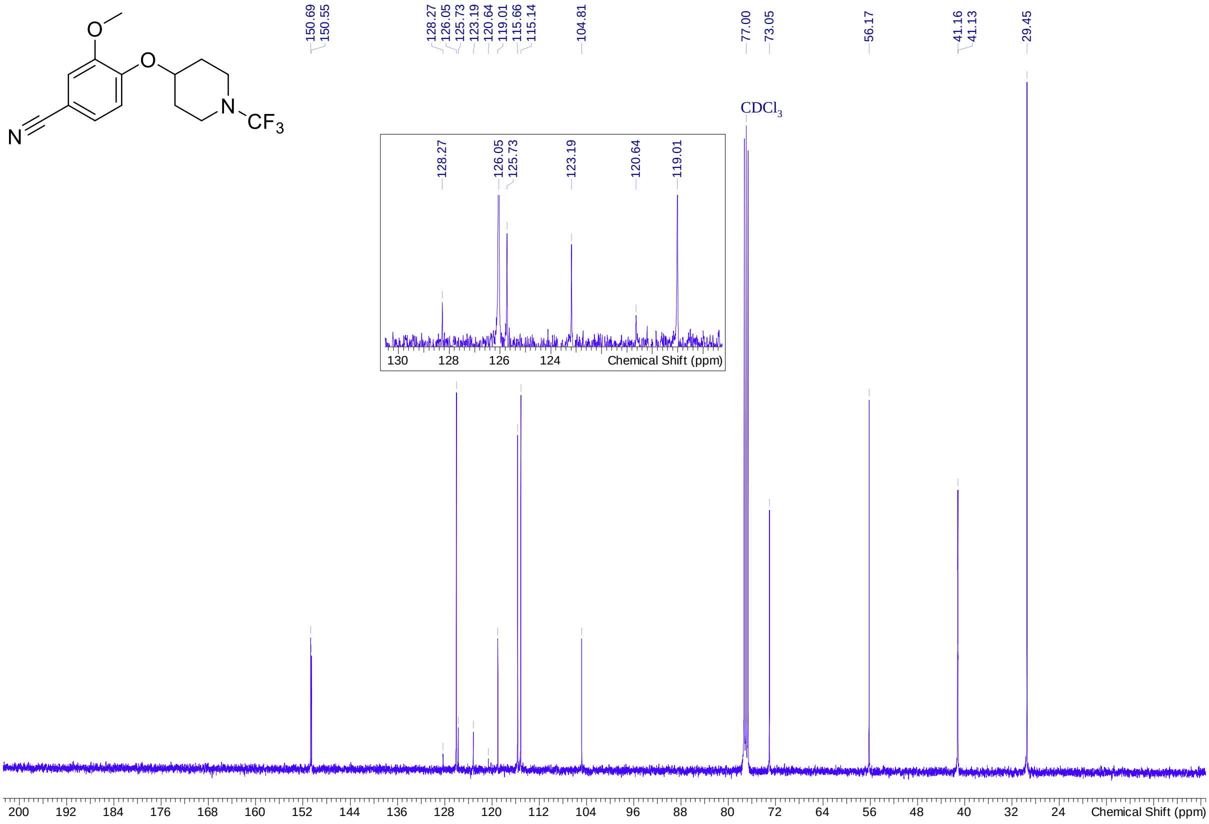

^{1}H NMR (400 MHz, $CDCl_3$) of Compound **2w**

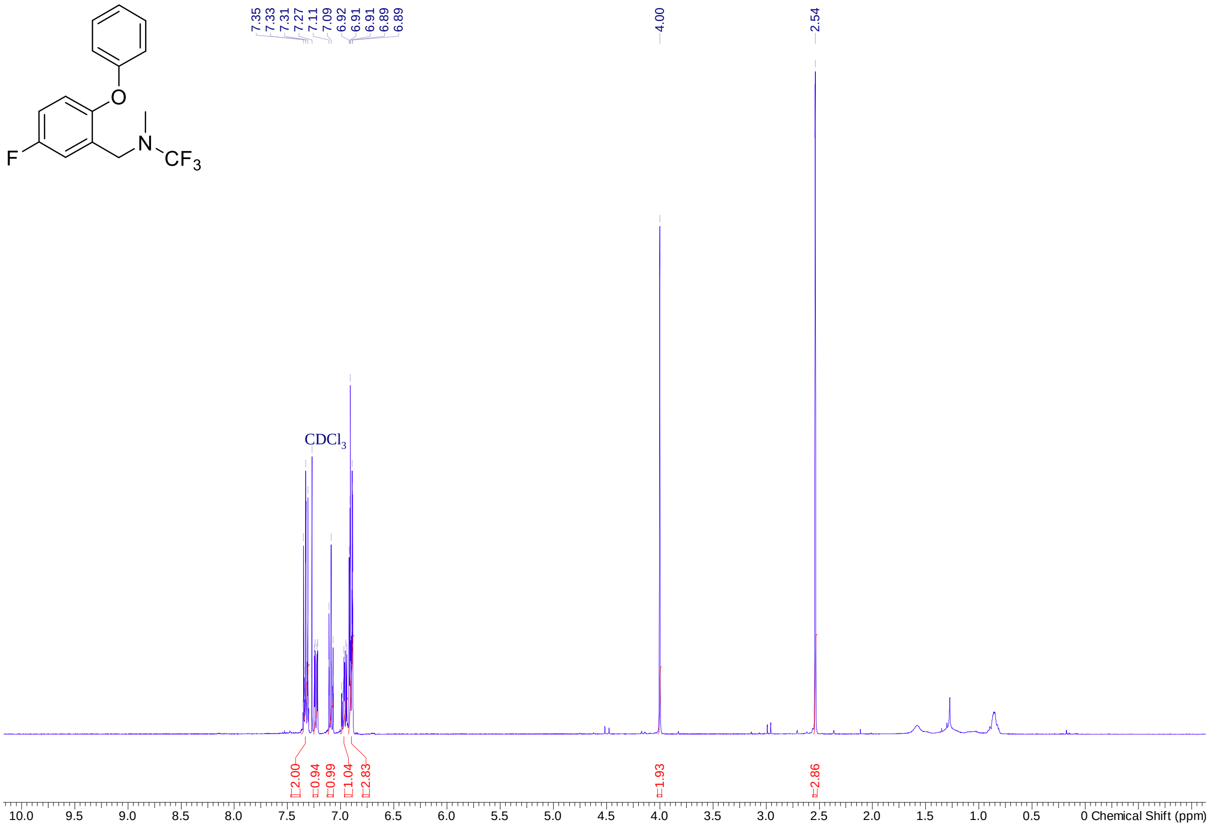

$^{19}F\{^1H\}$ NMR (376 MHz, $CDCl_3$) of Compound **2w**

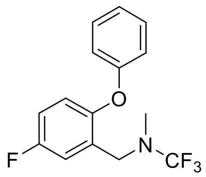

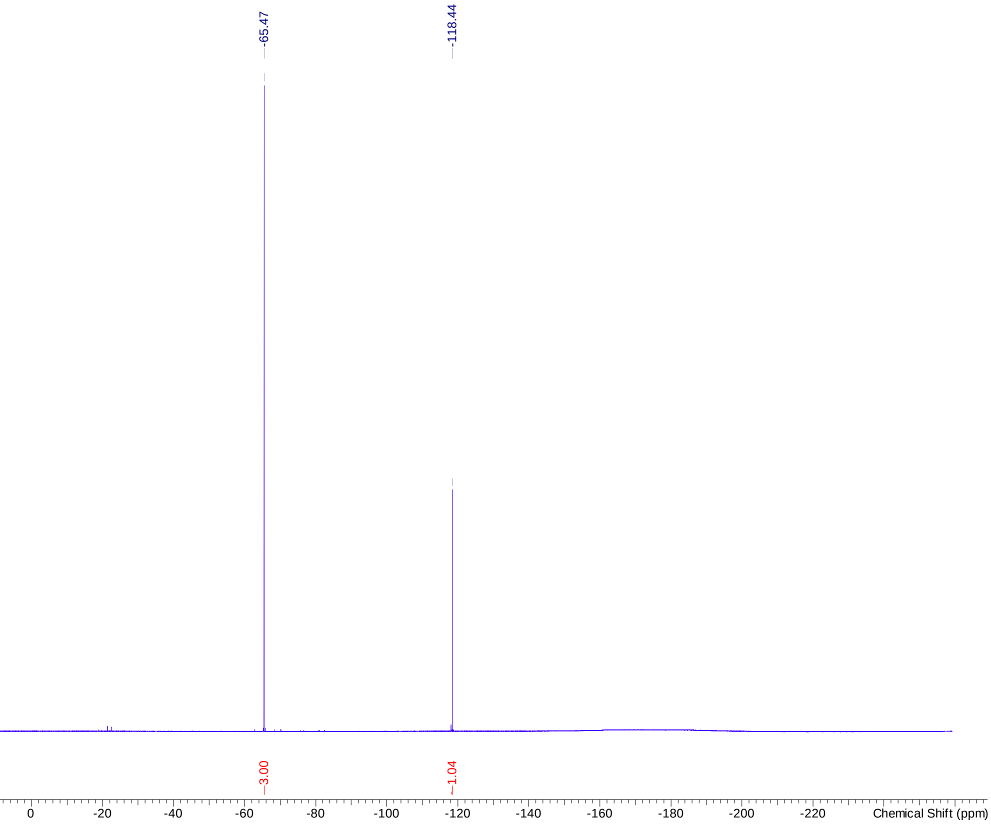

11. NMR Spectra of Isolated Side Products

^{1}H NMR (600 MHz, DMSO-d_6) of Compound **3a**

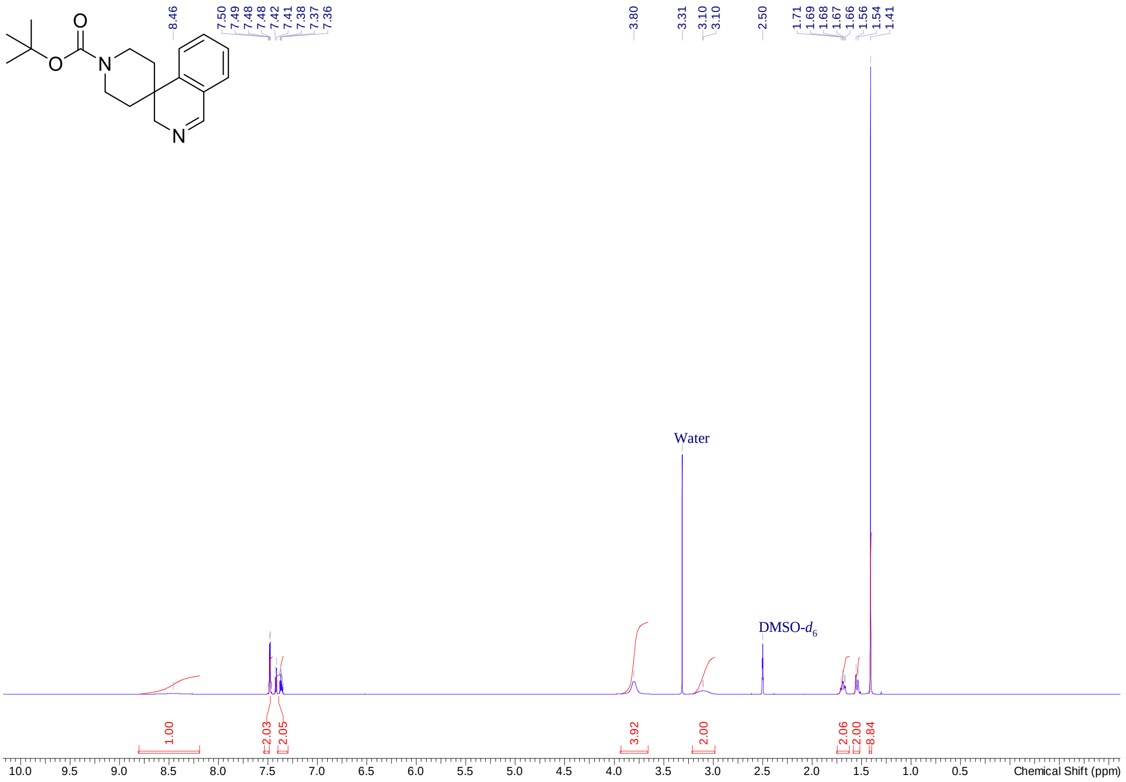

^{13}C NMR (151 MHz, DMSO-d_6) of Compound **3a**

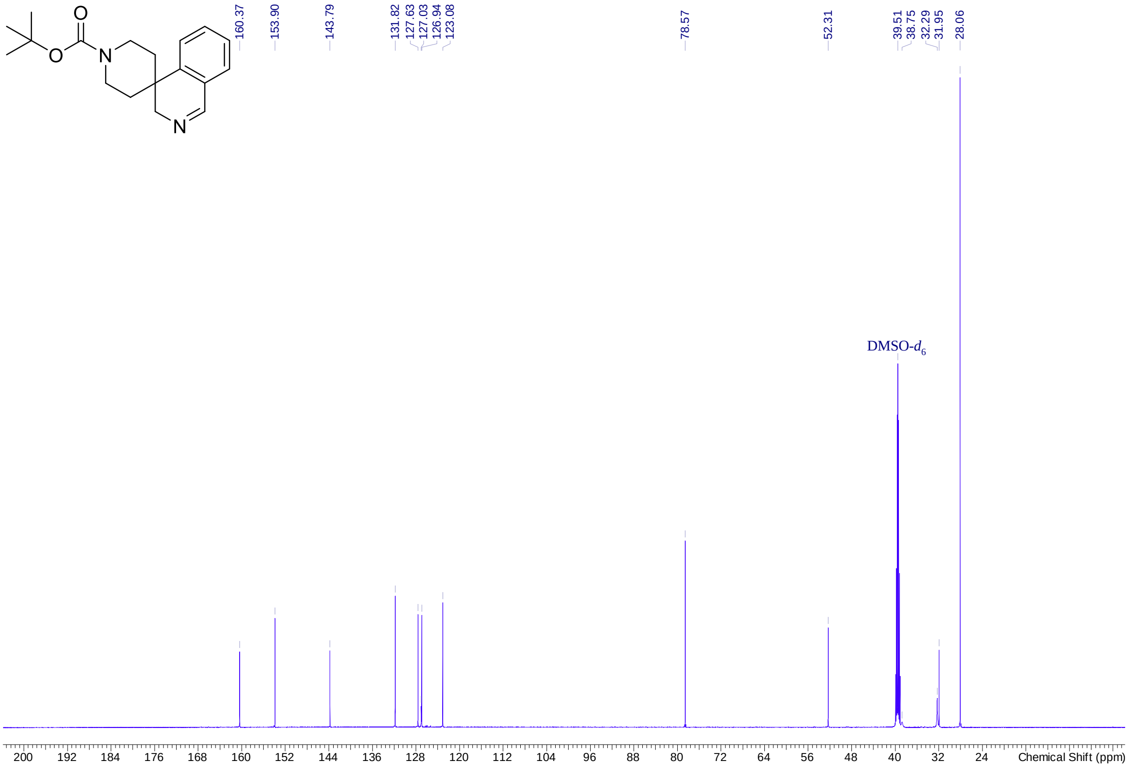

^{1}H NMR (400 MHz, DMSO-d_6) of Compound **3b**

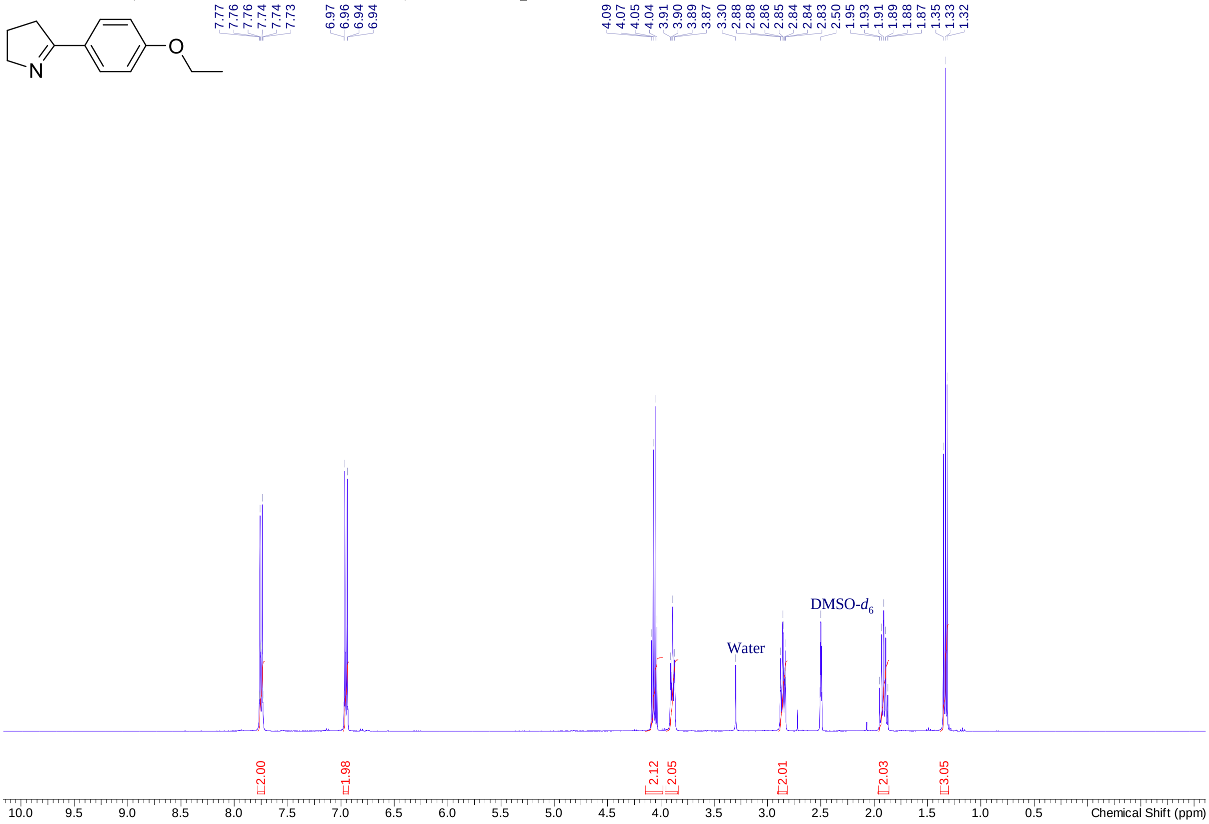

^{13}C NMR (101 MHz, DMSO-d_6) of Compound **3b**

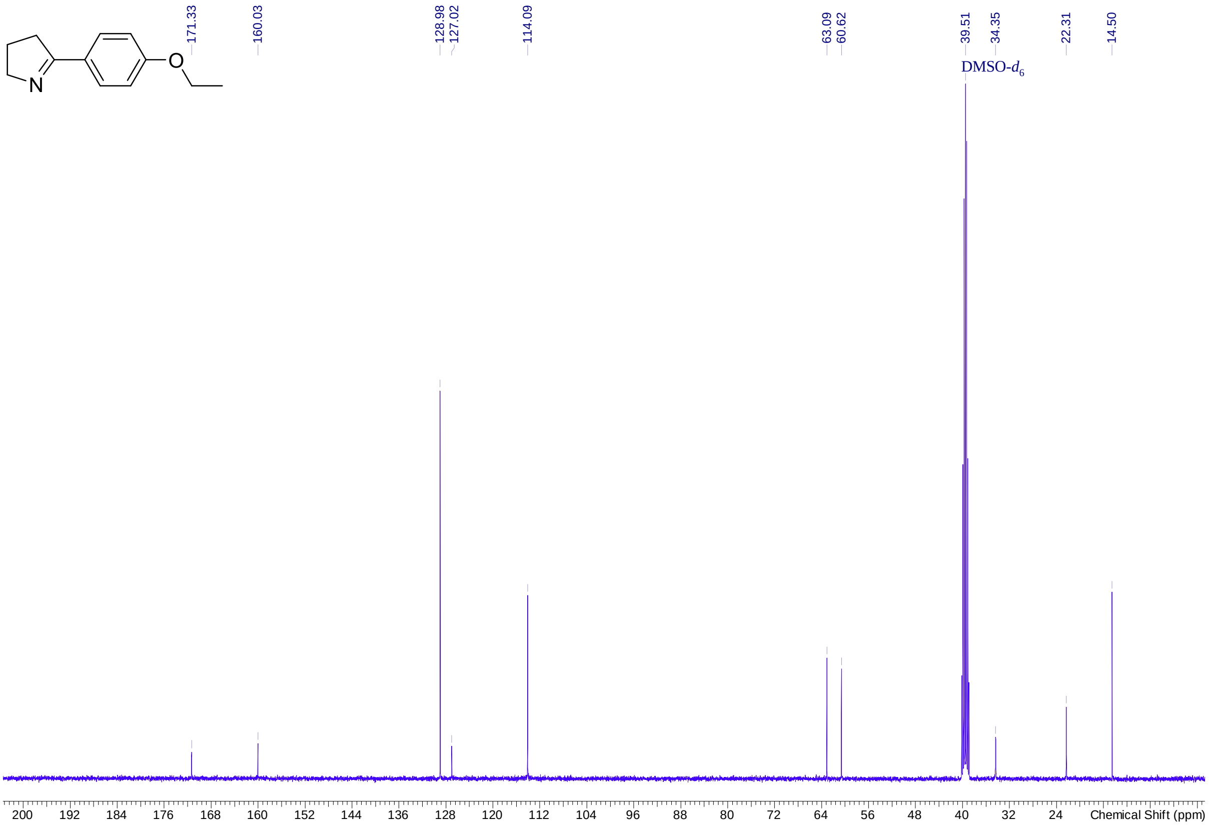

^{1}H NMR (400 MHz, DMSO-d_6) of Compound **S5a**

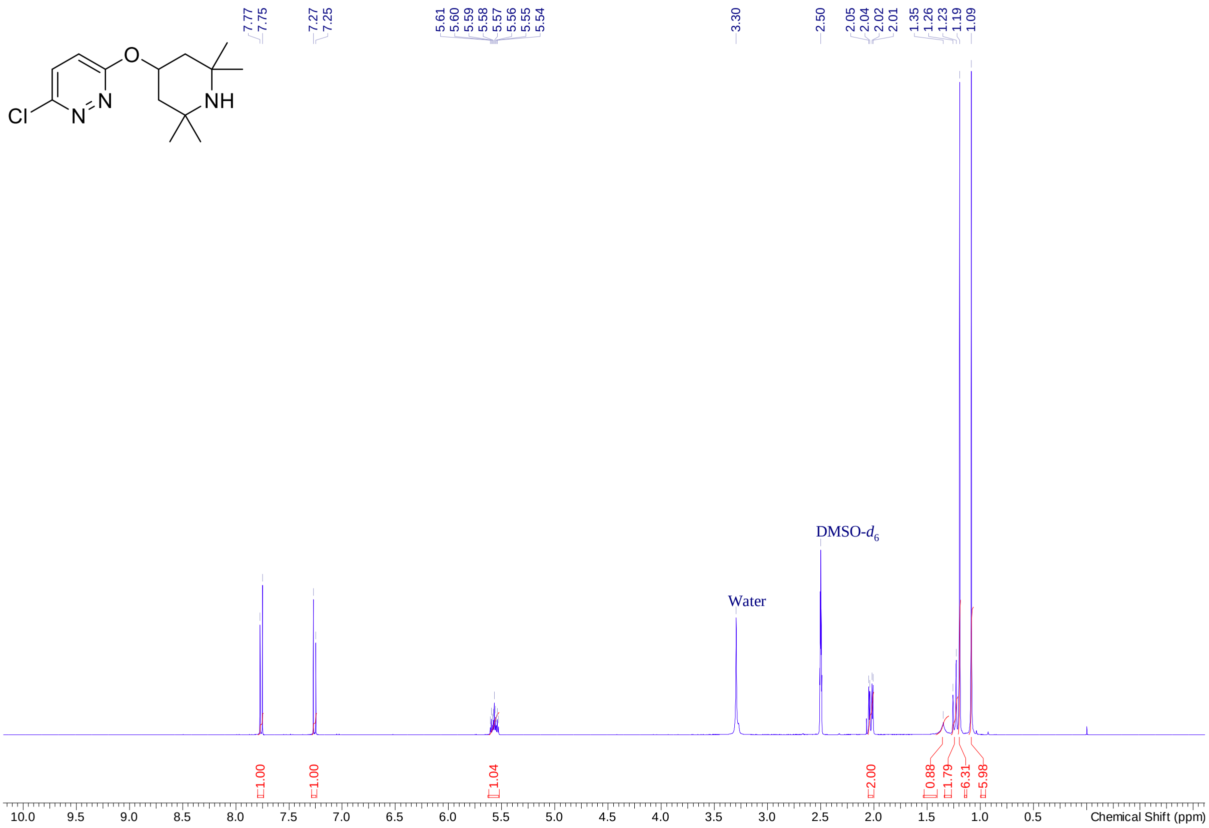

^{13}C NMR (151 MHz, DMSO-d_6) of Compound **S5a**

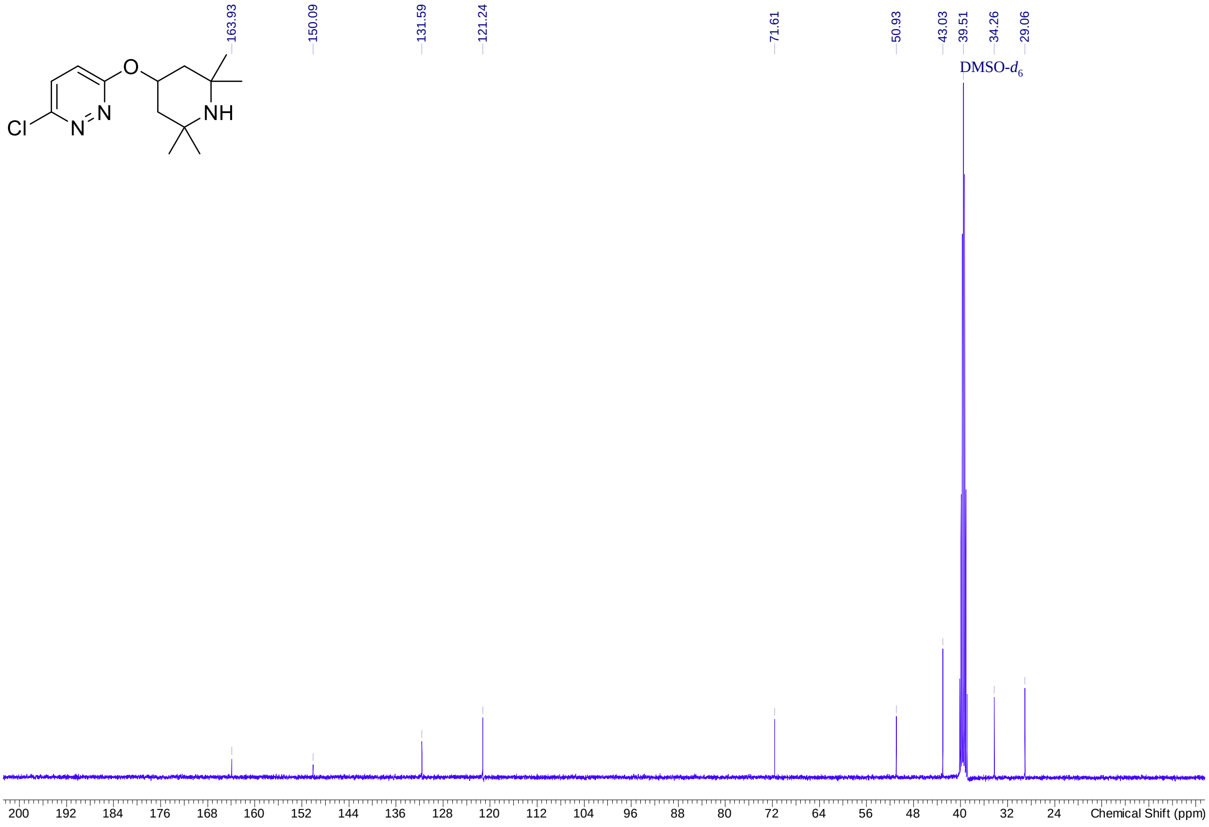

^{1}H NMR (400 MHz, DMSO-d_6) of Compound **6a**

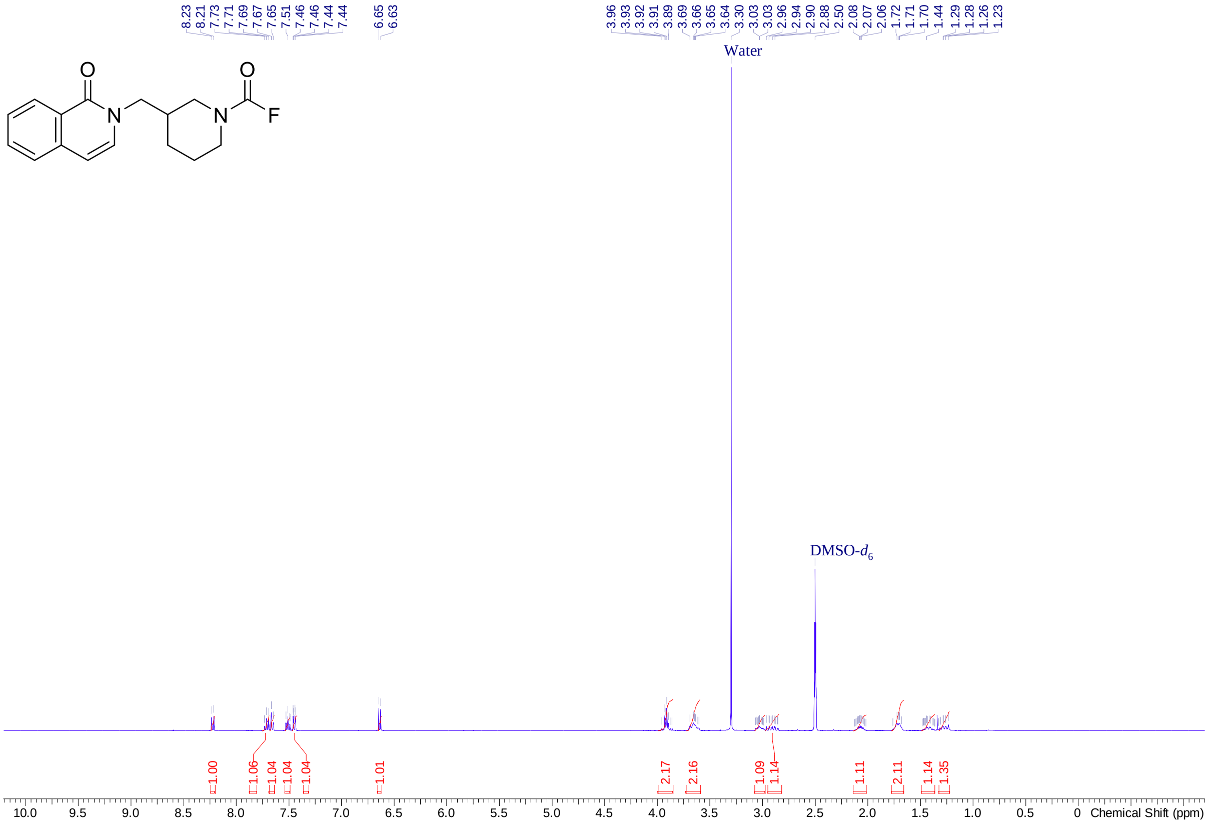

^{19}F NMR (376 MHz, DMSO-d_6) of Compound **6a**

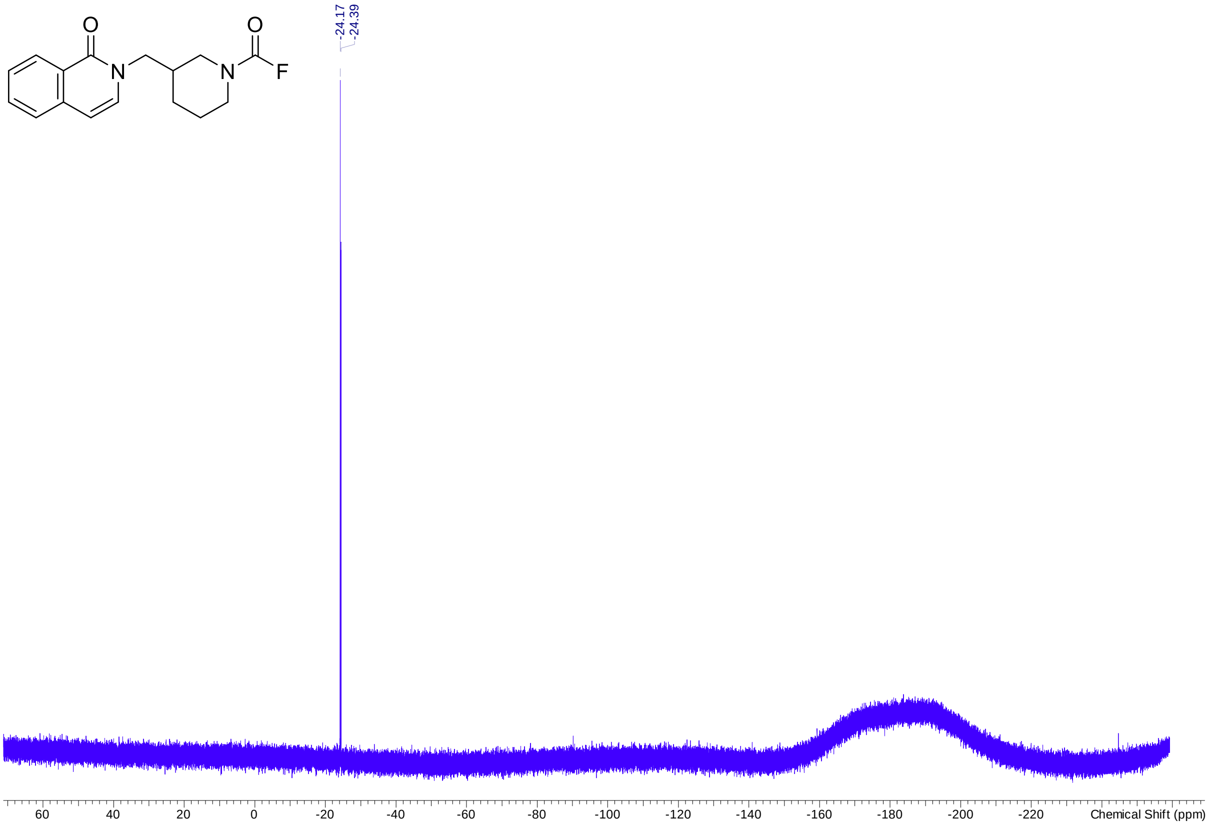

^{13}C NMR (151 MHz, DMSO-d_6) of Compound **6a**

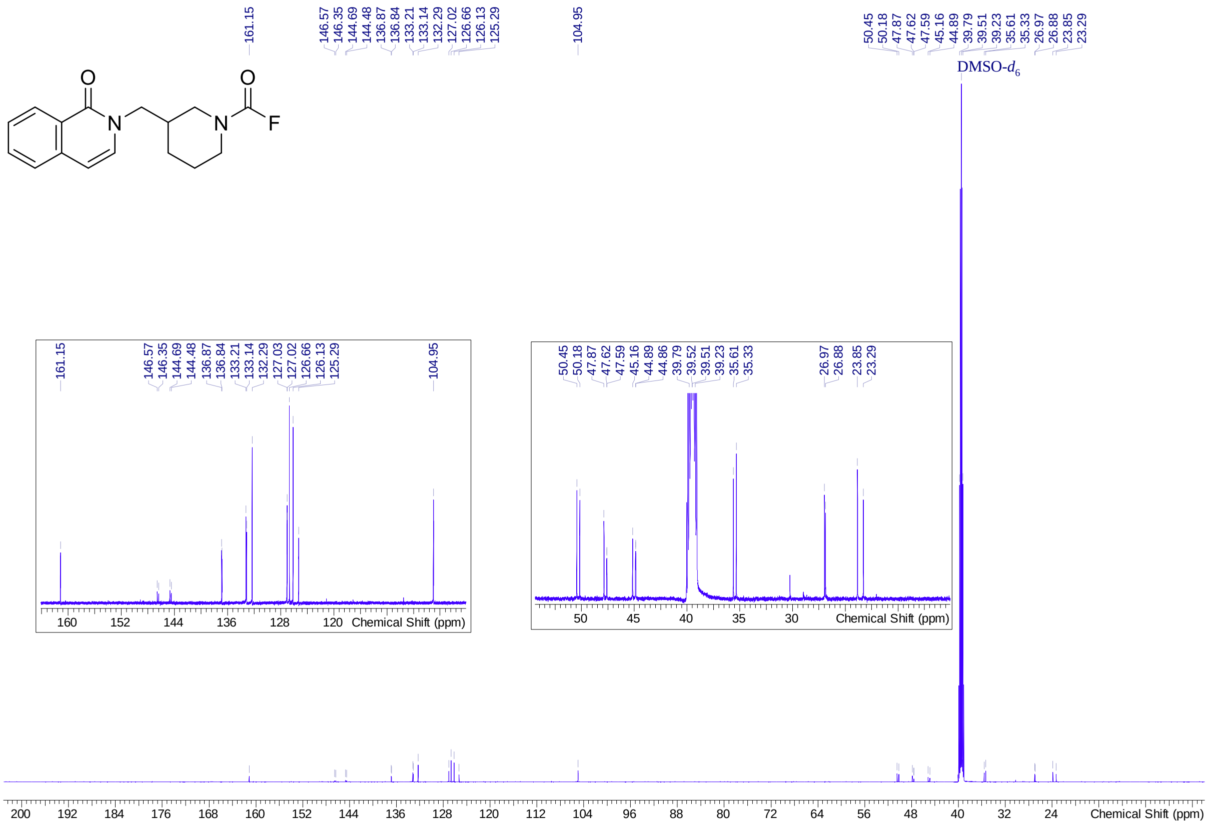

Supplement: Supplementary file 1 — Supporting Information [file CHEM-30-0-s001.pdf]
